# Curing hemophilia A by NHEJ-mediated ectopic F8 insertion in the mouse

**DOI:** 10.1186/s13059-019-1907-9

**Published:** 2019-12-16

**Authors:** Jian-Ping Zhang, Xin-Xin Cheng, Mei Zhao, Guo-Hua Li, Jing Xu, Feng Zhang, Meng-Di Yin, Fei-Ying Meng, Xin-Yue Dai, Ya-Wen Fu, Zhi-Xue Yang, Cameron Arakaki, Ruijun Jeanna Su, Wei Wen, Wen-Tian Wang, Wanqiu Chen, Hannah Choi, Charles Wang, Guangping Gao, Lei Zhang, Tao Cheng, Xiao-Bing Zhang

**Affiliations:** 1 State Key Laboratory of Experimental Hematology, Tianjin, 300020 China; 2 Institute of Hematology and Blood Disease Hospital, Chinese Academy of Medical Sciences and Peking Union Medical College, Tianjin, 300020 China; 3 School of Medicine, Loma Linda University, Loma Linda, CA 92354 USA; 4 CAMS Key Laboratory of Gene Therapy for Blood Diseases, Tianjin, 300020 China; 5 Center for Genomics, School of Medicine, Loma Linda University, Loma Linda, CA 92350 USA; 6 Horae Gene Therapy Center, University of Massachusetts Medical School, Worcester, MA 01655 USA; 7 National Clinical Research Center for Blood Diseases, Tianjin, 300020 China; 8 Tianjin Key Laboratory of Gene Therapy for Blood Diseases, Tianjin, 300020 China; 9 Center for Stem Cell Medicine, Chinese Academy of Medical Sciences, Tianjin, 300020 China; 10 Department of Stem Cell & Regenerative Medicine, Peking Union Medical College, Tianjin, 300020 China

**Keywords:** Hemophilia A, CRISPR-Cas9, Genome editing, Knock-in, NHEJ

## Abstract

**Background:**

Hemophilia A, a bleeding disorder resulting from *F8* mutations, can only be cured by gene therapy. A promising strategy is CRISPR-Cas9-mediated precise insertion of *F8* in hepatocytes at highly expressed gene loci, such as albumin (*Alb*). Unfortunately, the precise in vivo integration efficiency of a long insert is very low (~ 0.1%).

**Results:**

We report that the use of a double-cut donor leads to a 10- to 20-fold increase in liver editing efficiency, thereby completely reconstituting serum F8 activity in a mouse model of hemophilia A after hydrodynamic injection of Cas9-sgAlb and B domain-deleted (BDD) F8 donor plasmids. We find that the integration of a double-cut donor at the *Alb* locus in mouse liver is mainly through non-homologous end joining (NHEJ)-mediated knock-in. We then target *BDDF8* to multiple sites on introns 11 and 13 and find that NHEJ-mediated insertion of *BDDF8* restores hemostasis. Finally, using 3 AAV8 vectors to deliver genome editing components, including Cas9, sgRNA, and *BDDF8* donor, we observe the same therapeutic effects. A follow-up of 100 mice over 1 year shows no adverse effects.

**Conclusions:**

These findings lay the foundation for curing hemophilia A by NHEJ knock-in of *BDDF8* at *Alb* introns after AAV-mediated delivery of editing components.

## Background

Hemophilia A (HA) is one of the most common genetic disorders, with an incidence of 1 in 5000 male births in the USA, representing ~ 85% of all hemophilia cases [1]. HA is caused by mutations in the blood coagulation factor VIII (*F8*) gene on chromosome X. Recombinant F8 has been widely used to treat HA, but this has led to the induction of inhibitory antibodies in 20–30% of patients [2], limiting the efficacy of the treatment.

Significant progress has been made in treating hemophilia B (induced by *F9* mutations) by adeno-associated virus (AAV)-based gene therapy due to the short length of the F9 protein (461 amino acids long). Infusion of AAV vectors expressing factor IX Padua (F9–R338L) has achieved sustained expression of active F9 protein [3]. Due to the packaging limit of AAV, however, the progress of hemophilia A gene therapy is lagging. The entire F8 protein is 2332 amino acids long [4], but the deletion of a large portion of the B domain decreases the size by 38% [5]. As such, investigators have used B domain-deleted F8 (*BDDF8*) in gene therapy studies. After injection of high-dose BDDF8-encoding AAV5 and careful management of immune reaction to AAV by prednisolone administration, multiple adult patients achieved relatively stable serum F8 activity for up to 1 year [6]. Despite the promising outcome, long-term safety and efficacy remain to be determined [6] since hepatocyte turnover will lead to a gradual loss of AAV, albeit at a slow pace in adults. Re-administering the same vector is challenging as a result of AAV-neutralizing antibodies elicited by the initial treatment. For the same reason, non-integrating AAV therapy does not apply to pediatric patients. Here, we attempt to develop a therapy that may benefit patients of all ages using a genome editing approach.

Genome editing tools, such as zinc finger nuclease (ZFN) [7–9] and CRISPR-Cas9 [10–13], have been used for treating hemophilia B in mouse models. After dsDNA cleavage, a homology-directed repair (HDR) donor guides the targeted insertion of the promoterless vector at intron 1 or exon 2 of *F9*, leading to its expression. Thus far, there has been no report of successful HA treatment using CRISPR-Cas9, primarily due to the large size of the *BDDF8* gene (4.4 kb) compared to the *F9* gene (1.4 kb). Recently, we reported a five- to tenfold increase in precise gene knock-in using a double-cut donor vector design, in which Cas9-sgRNA induces simultaneous genomic DNA (gDNA) cleavage and release of a linearized HDR template [14]. We hypothesized that this approach would also increase the insertion efficiency of a large DNA fragment in vivo.

The liver is the preferable target organ for in vivo genome editing because hepatocytes can be efficiently transfected by AAV after intravenous injection or by naked plasmids after hydrodynamic injection [15, 16]. Gene targeting to the liver offers another advantage by inducing immune tolerance to vectors like AAV and therapeutic factors [17]. Since it is endothelial cells rather than hepatocytes [18] that mostly express F8, the in situ correction of *F8* in hepatocytes is not a viable therapeutic option. Instead, we attempted to target *BDDF8* at the albumin (*Alb*) locus, a highly expressed gene in hepatocytes [9, 19].

In this study, we report that the double-cut donor design leads to the integration of *BDDF8* in 1–2% of liver cells at *Alb* after hydrodynamic injection of plasmids encoding Cas9, sgAlb, and pDonor. As a result, we effectively corrected hemophilia A in most of the affected mice. We also delivered genome editing components into hepatocytes by intravenous injection of AAV8 vectors and found that multiple sites on *Alb* introns can be harnessed for non-homologous end joining (NHEJ) insertion of the *BDDF8* donor. This approach may be further developed into a clinical therapy for curing hemophilia A.

## Results

### High knock-in efficiency at *Alb* with a double-cut donor

We have recently reported that the use of a double-cut donor leads to a 5- to 10-fold increase in knock-in efficiency relative to circular plasmid donors [14]. Almost all the editing events in human pluripotent stem cells are HDR when homology arms of 300–600 bp are used. The double-cut donor is an HDR template flanked by single-guide RNA (sgRNA)-PAM sequences and is released after Cas9-sgRNA cleavage. Encouraged by this result, we attempted to use the same approach for in vivo genome editing of HA mice. We used a mouse model of hemophilia A, induced by targeted deletion of exon 16 of the *F8* gene [20].

**Fig. 1:**
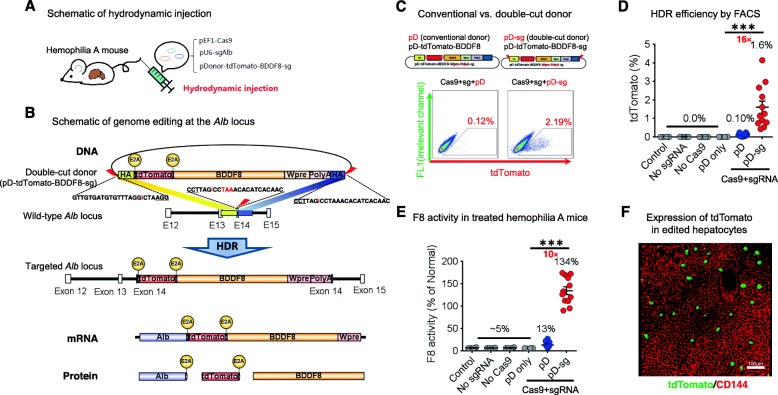
High-level insertion editing of the liver at *Alb* by a double-cut donor after hydrodynamic injection. **a** Schematic of hydrodynamic injection. Plasmids encoding Cas9 and a sgRNA targeting the *Alb* stop codon (sgAlb), together with an HDR template (pDonor), were delivered to the liver by hydrodynamic tail vein injection. **b** Schematic of genome editing at the *Alb* stop codon. Knock-in of promoterless *BDDF8* expression cassette at *Alb* was achieved by Cas9-sgAlb-mediated simultaneous cleavage of the genome and the double-cut donor pD-tdTomato-BDDF8-sg. The pD-sg template carries 600-bp homology arms, flanked by Cas9-sgAlb recognition sequences. Successful integration and transcription will produce three proteins (Alb, tdTomato, and BDDF8) as the result of E2A-mediated ribosomal skipping. **c** Knock-in efficiencies were determined by FACS analysis of tdTomato^+^ cells. Representative FACS diagrams are shown. **d** The double-cut donor considerably increases insertion efficiency in mouse liver cells after CRISPR-mediated dsDNA breakage. **e** Editing with the double-cut donor increases coagulant activity of F8. *n* = 12 mice for both conventional pD-tdTomato-BDDF8 and double-cut pD-tdTomato-BDDF8-sg donors. Omitting one or two editing components (*n* = 4 for each) served as negative controls. An unpaired *t* test with Welch’s correction was used for statistical analysis; ***P < 0.001. **f** A confocal liver section from edited mice shows expression of tdTomato in cells with hepatocyte morphology (representative of *n* = 5 mice). Scale bars are 100 μm

Similar to previous studies [19], we decided to target *BDDF8* to the fragment surrounding the *Alb* stop codon for high-level expression of the therapeutic factor. We used the plasmids pEF1-Cas9, whereby the EF1 promoter drives Cas9 expression, and pU6-sgAlb, whereby the U6 promoter drives the expression of an sgRNA targeting *Alb* (Additional file 1: Figure S1A). We first examined the cleavage efficiency by hydrodynamic tail-vein injection of CRISPR plasmids to the liver in adult mice (Fig. 1a) [16]. PCR amplification of the target site followed by deep sequencing 1 week after injection indicated indel efficiencies of 2–6% (Additional file 1: Figure S1B, C).

We then designed HDR donors in hopes to precisely insert *BDDF8* at *Alb*. To facilitate the analysis of gene-edited cells, we designed the target vector to insert both a tdTomato and BDDF8 coding sequence, linked by an E2A peptide derived from the equine rhinitis A virus (E2A) [21] (Fig. 1b), allowing the production of multiple proteins from a single reading frame by ribosomal skipping [21]. After editing, the tdTomato and BDDF8 expression cassette replaces the *Alb* stop codon, and the endogenous *Alb* transcriptional machinery drives the equimolar expression of Alb, tdTomato, and BDDF8.

We then compared pD-tdTomato-BDDF8 (pDonor), a conventional circular HDR donor, to pD-tdTomato-BDDF8-sg (pDonor-sg), a double-cut donor (Fig. 1c). The proportion of tdTomato^+^ cells in the liver 1 week after hydrodynamic injections represents the knock-in efficiencies. The pDonor-sg strikingly increased knock-in efficiency from ~ 0.1 to ~ 2% (~ 16-fold increase; Fig. 1c). As expected, the omission of sgRNA and/or Cas9 led to 0% tdTomato^+^ cells, suggesting that only precise insertion of the promoterless template leads to positive signals by FACS analysis (Fig. 1d).

After HDR integration, the *Alb* transcriptional machinery will drive the expression of Alb, tdTomato, and BDDF8. As expected, the F8 coagulant activity in treated HA mice mirrored the results of tdTomato^+^ cells, and the double-cut donor design increased F8 activity from 13 to 134% of normal levels in the plasma (Fig. 1e). We further confirmed the expression of tdTomato in edited hepatocytes by confocal imaging (Fig. 1f) and 3D reconstitution (Additional file 2: Video S1).

We also compared the pDonor vs. pDonor-sg template by targeting mNeonGreen, a bright green fluorescent protein, at *Alb*. We observed a 24-fold increase in the proportion of mNeonGreen^+^ cells 1 week after hydrodynamic injection when using the double-cut pDonor-sg compared to the conventional HDR pDonor plasmid (5.94% vs. 0.24%; Additional file 1: Figure S2).

Together, our double-cut donor vector design leads to a complete reconstitution of F8 coagulant activity 1 week after hydrodynamic injection of editing plasmids in HA mice.

### *BDDF8* knock-in at *Alb* stop codon is mediated by both NHEJ and HDR

**Fig. 2:**
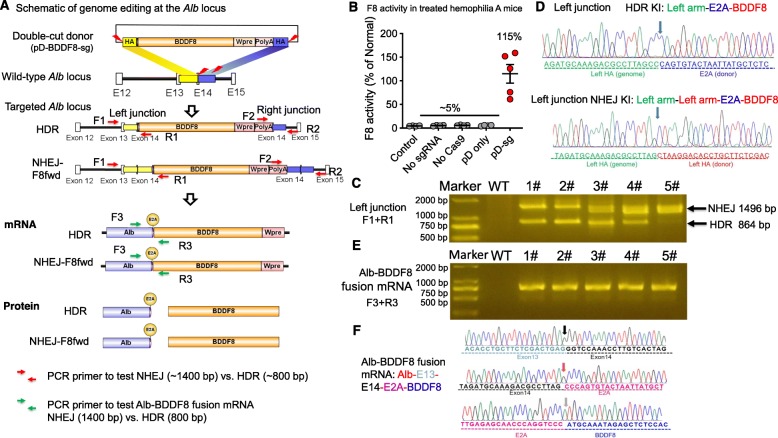
NHEJ and HDR mediated *BDDF8* knock-in at *Alb* stop codon. **a** Schematic of genome editing at the *Alb* stop codon. Knock-in of promoterless *BDDF8* expression cassette at *Alb* through NHEJ or HDR was achieved by Cas9-sgAlb mediated simultaneous cleavage of the genome and the double-cut donor pD-BDDF8-sg. The pD-sg template carries 600-bp homology arms. Knock-in by NHEJ or HDR mechanism can be differentiated by the size of the PCR products. HDR = 800 bp and NHEJ = 1400 bp. The left homology arm spans from the middle of exon 13 to the sgAlb target site. The right homology arm spans intronic sequence 3′ of the sgAlb target site. After integration and transcription by the endogenous *Alb* promoter/enhancer, two proteins (Alb and BDDF8) are produced as the result of E2A-mediated ribosomal skipping. polyA, polyadenylation site; WPRE, Woodchuck hepatitis virus (WHP) posttranscriptional regulatory element. **b** Editing with the double-cut BDDF8 donor restores F8 activity in hemophilia A (*n* = 5). Treatments without one or two editing component (*n* = 4 for each) serve as negative controls. An unpaired *t* test with Welch’s correction was used for statistical analysis; ***P < 0.001. **c** PCR analysis showing gene targeting mediated by both HDR and NHEJ. Liver samples were harvested 1 week after hydrodynamic injection of Cas9-sgAlb and the donor. We analyzed both the left and the right junctions by PCR. The locations of primers are indicated in **a**. PCR products were resolved by 2% agarose gel. gDNA from untreated mice (WT) serves as a negative control. **d** The identity of the NHEJ and HDR PCR products was confirmed by sequencing. Shown is the Sanger sequencing data of the left junction. **e** PCR analysis showing a successful fusion of *Alb* and *BDDF8* 1 week after hydrodynamic injection of Cas9-sgAlb and donor vectors. **f** DNA sequencing data confirm the correct splicing of exon 13 and exon 14, and the fusion of the E2A-BDDF8 cassette

It is also possible that the double-cut donor could insert at a target site through NHEJ directly [22]. To investigate the proportion of knock-in contributed by HDR vs. NHEJ, we amplified the left and right junctions using PCR. One primer is located at BDDF8 or PolyA and another at the outside of the homology arm (Fig. 2a). In this experiment, we did not include the marker gene tdTomato in the donor for simplicity. As expected, after injection of Cas9, sgRNA, or pDonor plasmid alone, the HA mice showed only baseline levels of F8 activity (~ 5% of normal serum levels).

Similarly to the study using the tdTomato-BDDF8 donor, the injection of all the editing vectors led to 115% F8 activity (Fig. 2b). After harvesting gDNA from the liver for PCR analysis 1 week after treatment, we observed donor integration by both HDR and NHEJ in all five mice. The band sizes for the left and right junctions are expected to be 864 bp and 835 bp for HDR knock-in and 1496 bp and 1421 bp for NHEJ-mediated insertion due to the presence of an extra copy of the homology arm of ~ 600 bp. In some cases, we observed a more intensive band, indicative of HDR insertion. However, this cannot be interpreted as a greater proportion of editing events mediated by HDR because PCR preferentially amplifies short amplicons (Fig. 2c). We attempted to quantitate the HDR vs. NHEJ insertion by droplet digital PCR (ddPCR), but failed because the ddPCR chemistry does not effectively amplify amplicons greater than 250 bp.

We then conducted pJET cloning using the PCR products. Sanger sequencing showed expected junction sequences for HDR and NHEJ insertions (Fig. 2d and Additional file 1: Figure S3). We noticed that the left homology arm contains 20 bp of exon 13 (E13) and intron 13 (In13) and 39 bp of exon 14 (E14). NHEJ insertion of pDonor is expected to create two identical copies of intron 13, which may lead to two possible splice isoforms (Additional file 1: Figure S4A). To distinguish these possibilities, we designed primers to amplify the *Alb-BDDF8* fusion transcript and observed only one band in the liver gDNA samples from five independent mice (Fig. 2e). Sequencing of the RT-PCR product showed correct splicing of *Alb* E13 and E14, and precise linkage of E14 coding sequence and E2A-BDDF8 (Fig. 2f and Additional file 1: Figure S4B-D). We speculate that the aberrant E14-E13 in-frame fusion exon was skipped for unknown reasons, likely because it is flanked by two identical introns. Together, these data demonstrate that both NHEJ and HDR insertions of the donor plasmid lead to the correct fusion transcript.

### Presence of various NHEJ insertion patterns at *Alb*

**Fig. 3:**
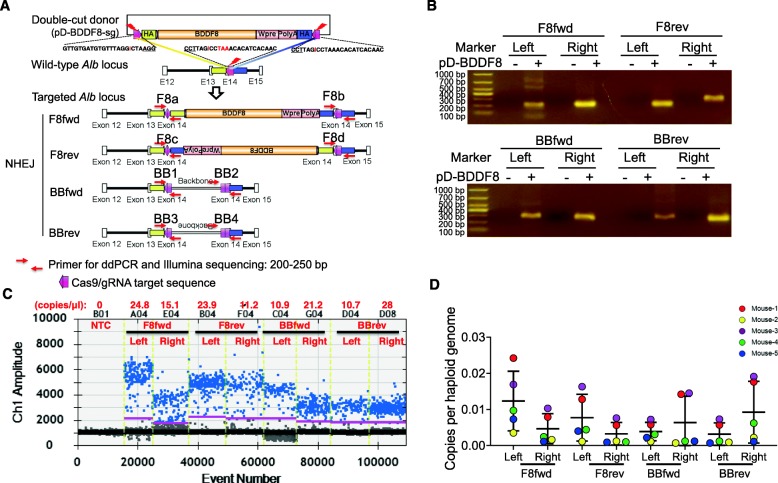
Characterization of NHEJ-mediated donor knock-in at Alb stop codon. **a** Schematic of forward and reverse integrations of the BDDF8 donor or plasmid backbone. Eight pairs of primers were designed to amplify the junctions (F8a, F8b, F8c, F8d, BB1, BB2, BB3, and BB4). The red arrow indicates the sgAlb target site. **b** Successful amplification of the eight junctions using designed primers. Shown is a representative result using live gDNA from one edited mouse. The identity of these PCR products was confirmed by Illumina sequencing (Additional file 1: Fig. S6). **c** A representative diagram of ddPCR analysis of the copy number of NHEJ-mediated knock-in. One hundred nanograms of gDNA was used in each reaction. To count the total number of haploid genomes interrogated, we used a probe that targets the *Actb* gene. **d** Quantitation of copy numbers for the eight junctions are shown. F8fwd, insertion of BDDF8 in the forward orientation; F8rev, insertion of BDDF8 in the reverse orientation; BBfwd, insertion of plasmid backbone in the forward orientation; BBrev, insertion of plasmid backbone in the reverse orientation

After cutting the double-cut *BDDF8* donor in the cells, two fragments are released: *BDDF8* and the plasmid backbone. Each of these fragments can insert at the dsDNA break via NHEJ in forward or reverse orientation (Fig. 3a). We designed eight pairs of primers (F8a-F, F8a-R; F8b-F, F8b-R; F8c-F, F8c-R; F8d-F, F8d-R; BB1-F, BB1-R; BB2-F, BB2-R; BB3-F, BB3-R; BB4-F, BB4-R) to amplify the eight possible NHEJ junctions. After validating the specificity of the primers (Fig. 3b), we conducted ddPCR to measure the precise copies of each NHEJ pattern (Fig. 3c). Copies of the *Actb* gene served as a loading control in each reaction (Additional file 1: Figure S5). Summary of ddPCR data of five mice sacrificed 3 weeks after injection showed 0.002–0.02 copies per haploid genome for each type of insertion (Fig. 3d).

Of interest, we observed similar insertion events of the *BDDF8* cassette (~ 5.5 kb) and the plasmid backbone (~ 2.2 kb), suggesting that large fragments can also effectively insert into the genome via NHEJ. If the fragment is inserted at the Cas9-sgAlb cleavage site without significant modification, we would expect identical copies of the left and right junctions. However, we observed significantly higher copy numbers of the left vs. right junctions for the *F8* fragment, and the reverse was true for the backbone fragment (Fig. 3d). We reasoned that this could attribute to the creation of a Cas9-sgAlb target site in 50% of the junctions after precise NHEJ. The secondary cut of these junctions might lead to relatively large deletions [23], which would evade detection by ddPCR. To investigate these possibilities, we sequenced the PCR products using the Illumina platform. In support of this argument, we observed 50–90% precise NHEJ whenever there was no possible secondary cut. In contrast, only 10–20% junctions were precise NHEJ whenever precise insertion of a template fragment created a Cas9-sgAlb target site (Additional file 1: Figure S6).

### Decreasing the length of homology arms does not affect therapeutic effects

**Fig. 4:**
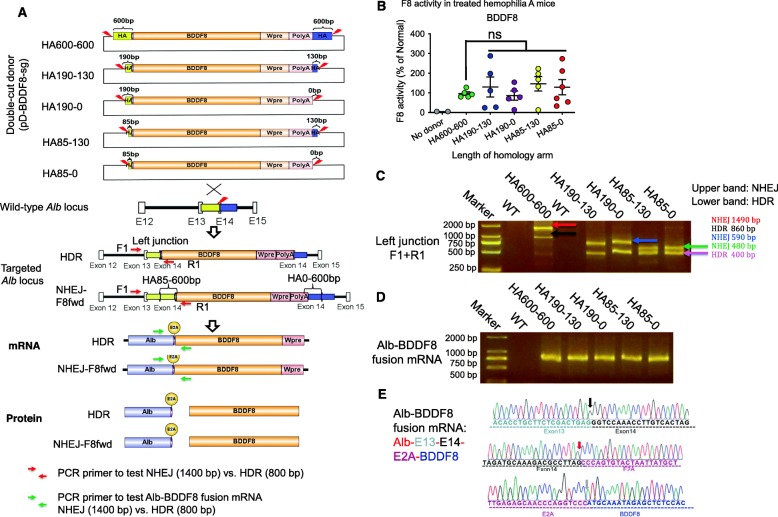
*BDDF8* insertion at the *Alb* stop codon is predominantly through NHEJ. **a** Schematic of genome editing at the *Alb* stop codon using double-cut *BDDF8* donors with different lengths of homology arms. The pD-sg templates carry the different length of homology arms (HA600-600, HA190-130, HA190-0, HA85-130, HA85-0), flanked by Cas9-sgDocut recognition sequences. NHEJ- or HDR-mediated knock-in can be differentiated by the size of the PCR product using primers F1 and R1. Successful integration leads to the transcription of an Alb-BDDF8 fusion gene, which translates to two proteins: Alb and BDDF8. **b** High-level F8 activity 1 week after injection of Cas9-sgAlb and double-cut pD-BDDF8-sg donors with different length of homology arms (*n* = 2–5 for each group). Mice treated without donor only (*n* = 2) serves as a negative control. An unpaired *t* test with Welch’s correction was used for statistical analysis; ***P < 0.001. **c** PCR analysis showing successful gene targeting by both HDR and NHEJ. PCR analysis of the left junction in edited mice. The locations of F1 and R1 primers are shown in **a**. PCR products were resolved on a 2% agarose gel. Untreated mice (WT) showed no evidence of targeting. **d** Quantification of NHEJ and HDR editing at the left junction using ddPCR. Liver gDNA was extracted 1 week after editing using donor pD-BDDF8(HA85-130). We used probes targeting both the junction (NHEJ) and HA85 (NHEJ+HDR) in ddPCR. **e** Amplification of the fusion transcript of *Alb* and *BDDF8* by RT-PCR. **f** Sanger sequencing data show correct splicing of exon 13 and exon 14 and the exon 14-E2A junction

In the above studies, the HDR donor carries a 600-bp homology arm at both ends (600-600). Since NHEJ instead of HDR accounted for large quantities of insertion events, we next attempted to examine the effects of homology arm length. To this end, we designed five additional pD-BDDF8-sg vectors with different lengths of homology arms (HA600-600, HA190-130, HA190-0, HA85-130, HA85-0) flanked by Cas9-sgDocut recognition sequences. (Fig. 4a). One week after hydrodynamic injection of Cas9-sgAlb, which targets the *Alb* stop codon on exon 14, together with one of the five pD-BDDF8-sg plasmids, we observed 100–200% F8 activity in all groups (Fig. 4b). No significant differences in the plasma coagulation activity were observed, suggesting that homology-directed repair may only play a minor role in *BDDF8* knock-in.

To investigate the knock-in patterns, we used primers to amplify the left junction (Fig. 4a). In all representative samples, we observed 2 bands with sizes identical to the predicted NHEJ (higher band) and HDR (lower band) (Fig. 4c). Sanger sequencing confirmed the identity of these bands (Additional file 1: Figure S7). We cloned the PCR product into a pJET vector and sequenced over 20 clones. We found that NHEJ accounted for over 40~70% of all knock-in events (data not shown). It is worth pointing out that this approach might have underestimated NHEJ events due to the preferential amplification and insertion of short PCR products.

We then conducted RT-PCR to amplify the junction of the *Alb*-*BDDF8* fusion transcript. We observed a single band, indicative of precise splicing of *Alb* E13 and E14, and E2A-BDDF8 in five representative samples (Fig. 4d). Sanger sequencing of the RT-PCR products verified these results (Fig. 4e and Additional file 1: Figure S8). These data demonstrate that both HDR and NHEJ knock-in lead to correct fusion transcripts.

Together, these data demonstrate that HDR editing in hepatocytes is unnecessary for successful knock-in of a therapeutic gene. Instead, NHEJ integration in the liver is more efficient, even in the presence of homology arms. As such, we decided to focus on investigating the NHEJ-mediated integration of *BDFF8* using donors without homology.

### NHEJ-mediated integration of *BDDF8* at *Alb* intron 11 or 13 cures hemophilia A

**Fig. 5:**
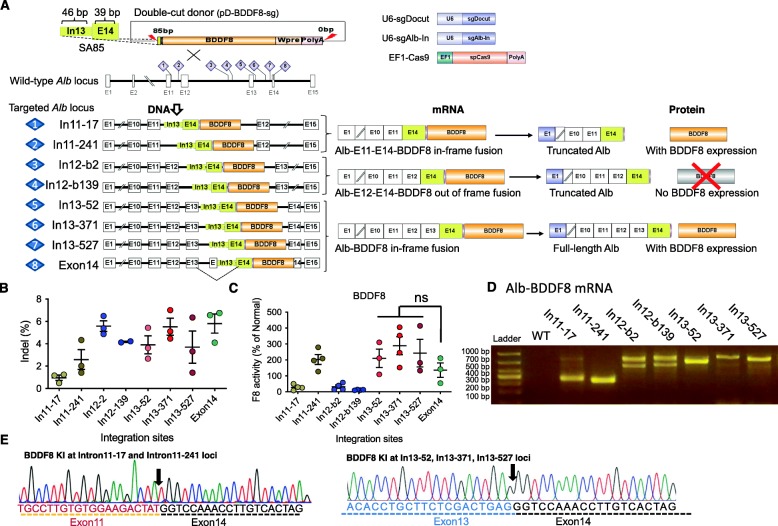
NHEJ-mediated knock-in of *BDDF8* at *Alb* introns 11 or 13 cures hemophilia A. **a** Schematic of the intron targeting of *BDDF8* at the mouse *Alb* locus. We used ten sgRNAs in this study. The yellow box indicates the *Alb* sequence on the donor: 3′ end of intron 13 (46 bp) and 5′ end of exon 14 before the stop codon (39 bp), which serves as a splice acceptor. After cut at an *Alb* intron, the linearized donor will integrate via the NHEJ repair pathway. Right panels show the predicted fusion mRNA and protein products. **b** Assessment of sgRNA cleavage efficiency by Illumina sequencing 1 week after injection of Cas9-sgRNA. An unpaired *t* test with Welch’s correction was used for statistical analysis; ***P < 0.001. **c** Differential F8 activity after knock-in of *BDDF8* donor at different loci of the *Alb* gene. sgRNAs targeting intron 1 and intron 12 serve as negative controls. **d** Analysis of Alb-BDDF8 fusion transcripts by RT-PCR. **e** DNA sequencing data confirmed the correct splicing of different *Alb* exons or skipping an *Alb* exon or inclusion of an exon from the plasmid backbone

The insertion of *BDDF8* by NHEJ instead of HDR makes it unnecessary to target the *Alb* stop codon. We designed three sgIn13 to target intron 13 and used pD-BDDF8-sgDocut (SA85-0) as a donor template. We renamed HA85, which consists of 46 bp and 39 bp of the intron 13-exon 14 junction sequence, to SA85, indicating its function as a splice acceptor. To increase the flexibility of vector combination, we flanked the *BDDF8* donor with sgDocut recognition sequences so that a single donor could be used in all cases (Fig. 5a). In this study, we included three sets of controls: (1) two sgIn12 targeting intron 12 to form an out-of-frame fusion transcript, leading to no F8 expression; (2) two sgIn11 targeting intron 11, which will result in a truncated Alb and functional BDDF8; and (3) an sgRNA targeting the stop codon on exon 14 (Fig. 5a).

We first examined the cleavage efficiency of these sgRNAs 1 week after hydrodynamic tail-vein injection of Cas9-sgRNA plasmids. PCR amplification of the target regions followed by high-throughput sequencing analysis showed that seven out of eight sgRNAs could effectively cut its target sequence, with indel efficiencies of 2–6% (Fig. 5b and Additional file 1: Figure S9). SgIn11-17 showed lower indel efficiencies of ~ 1%. We then hydrodynamically injected HA mice with pDonor, Cas9 plasmid, and two sgRNA plasmids, one cutting *Alb* intron and another linearizing the double-cut donor plasmid. One week later, we examined the F8 activity in the plasma.

Of interest, targeting intron 12 led to low levels of F8 at 10–20%. We interpreted this result as a large deletion including exon 12 in a small portion of cells, leading to the inframe splicing of exon 11 to exon 14 and E2A-BDDF8 (Fig. 5c). The use of four out of five sgRNAs targeting intron 11 or 13 showed high levels of F8 activity (Fig. 5c). SgIn11-17 led to low F8 activity, which is associated with its low cleavage efficiency (Fig. 5b). Together, NHEJ-mediated insertion of *BDDF8* at introns is a viable therapeutic strategy.

We further characterized *BDDF8* integration at the RNA level. We designed primers to amplify the junction between *Alb* exon 10 and *BDDF8*. Targeting intron 11 and intron 13 led to expected fusion transcripts. Of interest, in some samples, we also observed a lower band, indicative of exon 11 or exon 13 deletion (Additional file 1: Figure S10). We interpreted the data as large deletions in a small portion of cells.

Surprisingly, RT-PCR analysis detected two bands in all intron 12-targeting samples. Sanger sequencing revealed that the lower band was the predicted fusion transcript that lacks exon 13. However, the higher band had an extra 206 bp that matched the plasmid backbone, inserted between exon 12 and exon 14. We used the Human Splice Finder tool [24] to analyze the backbone sequence and identified an exon of 206 bp with an identical sequence to the insert (Additional file 1: Figure S11). As such, we speculate that the insertion of the plasmid backbone together with the *BDDF8* donor led to this incident.

Collectively, our data demonstrate that targeting introns is a feasible strategy for in vivo gene therapy. There may be, however, unexpected fusion transcripts due to the occasional large deletions or insertion of vector backbone.

### AAV-CRISPR therapy cures hemophilia A mice

**Fig. 6:**
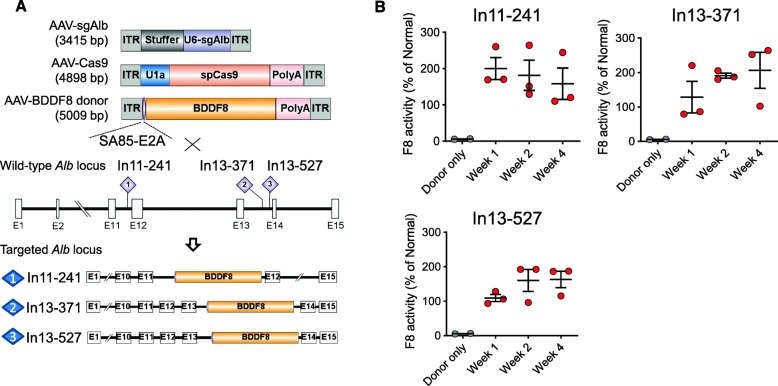
AAV-CRISPR therapy cures hemophilia A mice. **a** Schematic of the AAV vectors used in the study and experimental overview for in vivo studies. SA85 on the donor functions as a splice acceptor. **b** Stable F8 activity after injection of AAV-Cas9, three different AAV-sgRNAs targeting *Alb* introns 11 or 13, and AAV-BDDF8 donor. (*n* = 3 for each group). A group of mice treated with AAV-BDDF8 donor only (*n* = 3) serves as a negative control

We have conducted a series of experiments to optimize the conditions for knock-in of *BDDF8* at *Alb*. In the above studies, we used hydrodynamic injection for the delivery of editing plasmids to mouse liver. We then attempted to use adeno-associated vector (AAV) to deliver *BDDF8* donor and CRISPR components for clinic translation. We chose AAV8 because serotype eight adeno-associated vector can effectively transduce mouse hepatocytes [25]. After deletion of the homology sequence, the AAV-BDDF8 donor flanked by splice acceptor and a rabbit beta-globin polyadenylation signal (126 bp) has a genome size of 5009 bp, which is within the upper limit for effective AAV packaging. For the Cas9 vector, we replaced the human EF1 promoter (1.2 kb) with the mouse U1a promoter (251 bp), resulting in an AAV with a genome size of 4898 bp [26]. In the sgAlb vector, we added a stuffer of 2.5 kb in length (Fig. 6a). Droplet digital PCR analysis of AAV titers showed that all the vectors could be adequately packaged.

In the preliminary AAV-CRISPR-BDDF8 study, we selected three sgAlb-Ins that showed effective NHEJ insertion of *BDDF8* after hydrodynamic injection of plasmids. We injected adult HA mice intravenously with 1 × 10^11^ genome copies (GC) of AAV-Cas9, 1 × 10^11^ GC of AAV-sgAlb, and 5 × 10^11^ GC of AAV-BDDF8. Follow-up on the mice at 1, 2, and 4 weeks after injection showed a stable F8 activity of 100–200% (Fig. 6b). As expected, the injection of the promoterless AAV-BDDF8 alone showed no therapeutic effect (Fig. 6b). These preliminary results demonstrate the feasibility of AAV-CRISPR therapy in treating hemophilia A.

Recent reports showed high-level AAV integration in Cas9-induced double-strand breaks (DSBs) [27, 28]. We thus designed primers to amplify the junction sequences after insertion of AAV-BDDF8 or AAV-Cas9 at *Alb* intron 13–371 or 527 (Additional file 1: Figure S12A). PCR analysis identified the junction sequences of all possible AAV insertion patterns, demonstrating the insertion of both AAV-F8 and AAV-Cas9 sequences in double-strand break sites (Additional file 1: Figure S12 BC). We conducted pJET cloning and picked over 100 colonies for Sanger sequencing and found that NHEJ-mediated insertion led to an average of ~ 15 bp deletions in the genome cleavage site and ~ 80 bp deletion of AAV ITR (Additional file 1: Figure S13).

### Long-term, persistent BDDF8 expression after transient immunosuppression

We observed 50–200% F8 bioactivity 1 week after the administration of Cas9-sgAlb and pD-BDDF8-sg donor plasmids, but F8 levels decreased considerably to ~ 14% in 6 out of 13 mice 3 weeks later (Additional file 1: Figure S14). The immune response to exogenously infused F8 is a major complication in the treatment of hemophilia A patients [29]. Thus, we assessed F8 inhibitor titers with the Nijmegen-Bethesda assay (Additional file 1: Figure S14). Compared to the untreated HA mice, there were no detectable inhibitors in F8-stable mice, but a significant increase in F8-decline mice, indicating that the humoral response to F8 contributed to decreased F8 activity. As a positive control for the reaction against F8, we injected the mice with a plasmid in which the EF1 promotor drives BDDF8 expression, and high-titer F8 inhibitors were detected (Additional file 1: Figure S15).

We decided to use immunosuppressants to control the immune reaction. We tracked the F8 coagulant activity after hydrodynamic injection of Cas9-sgAlb and the donor pD-tdTomato-BDDF8-sg for 12 weeks after transient immunosuppression. We chose the combination of methylprednisolone (MPS) and cyclophosphamide (CTX), which have been used in HA gene therapy previously [6, 30]. Intraperitoneal injection of MPS (50 mg/kg) and CTX (50 mg/kg) seven times in 3 weeks increased the 3-month F8 stability to > 80% (Additional file 1: Figure S16). We then investigated how the immunosuppression maintained F8 stability. We observed an increase of edited liver cells (tdTomato^+^) from 1.0 to 2.2% after immunosuppression at 3 weeks, suggesting inhibition of the cellular immune response (Additional file 1: Figure S16). We also observed a significantly decreased humoral response against F8 (Additional file 1: Figure S16). These data demonstrate that transient immunosuppression can effectively control both cellular and humoral immune reactions to F8, leading to a sustained therapeutic efficacy.

### Life-long efficacy and safety of genome editing therapy for hemophilia A

**Fig. 7:**
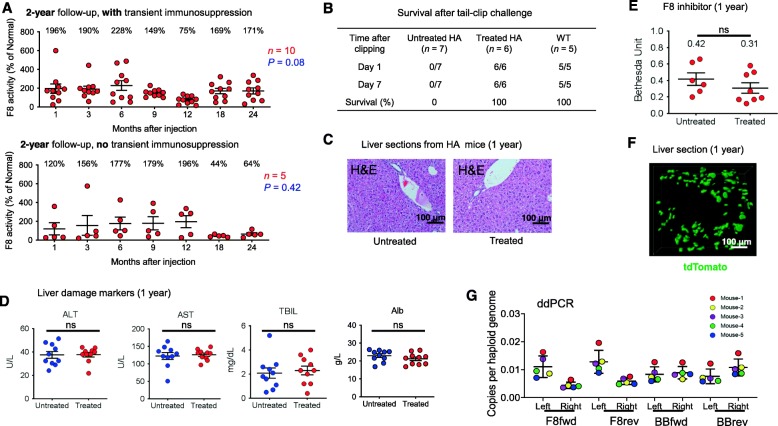
Long-term efficacy and safety in BDDF8-edited hemophilia A mice. **a** Long-term follow-up on the F8 activity of hemophilia A mice. The animals were hydrodynamically injected with Cas9-sgAlb and the double-cut donor pD-BDDF8-sg (*n* = 15). The exact *P* value is shown by a one-way ANOVA analysis. **b** Treated mice survive a tail-clip challenge. Wild-type C57BL/6 (WT) mice (*n* = 5) served as a positive control. **c** Hematoxylin and eosin (H&E) staining of the liver sections of untreated and treated HA mice 1 year after injection. Shown are representative images from five mice. **d** Liver toxicity markers 1 year after treatment. AST, aspartate aminotransferase; ALT, alanine aminotransferase; TBIL, total bilirubin; Alb, total albumin (*n* = 10). No significant differences were observed between untreated HA mice (*n* = 10) and treated HA mice (*n* = 10) by unpaired *t* test with Welch’s correction. **e** Levels of F8 inhibitors in mouse plasma measured by Bethesda assay. Untreated 1 year (*n* = 8); treated (*n* = 8). An unpaired *t* test with Welch’s correction was used for statistical analysis. ns, not significant. **f** Two-photon imaging of liver tissues indicates a stable expression of tdTomato. CD144 (VE-cadherin) stains the liver vasculature structure; edited cells (tdTomato-BDDF8) were pseudo-colored as green. Shown is a representative image of *n* = 4 mice. **g** ddPCR analysis indicates the long-term presence of junctions of NHEJ mediated knock-in 1 year after treatment. Schematic and detailed information was presented in Fig. 3

In the above studies, we tracked the F8 activity after hydrodynamic injection of plasmids for up to 3 months. We have now followed up on ~ 100 treated mice for more than 1 year (Fig. 7a and Additional file 1: Figure S17) with or without transient immunosuppression. Fifteen animals have been followed up for 2 years (the maximum lifespan of HA mice in our experience). We observed occasional fluctuations in F8 activity in individual mice, likely due to technical reasons. In all mice, however, we found overall sustained F8 activity, ~ 100% of normal levels, ranging from 20 to 400% (Fig. 7a and Additional file 1: Figure S17). To assess the coagulation activity of treated HA mice, we conducted the tail-clip challenge assay. As expected, 0% (zero out of seven) of untreated HA, 100% (six out of six) of treated HA, and 100% (five out of five) of wild-type C57BL/6 mice survived the traumatic hemorrhage (Fig. 7b).

We sacrificed multiple, randomly chosen mice 1 year after treatment for detailed analysis. Hematoxylin and eosin (H&E) staining and gross analysis of the liver showed no anatomical differences between treated and untreated mice (Fig. 7c). Serum markers of liver damage such as aspartate aminotransferase (AST), alanine aminotransferase (ALT), total bilirubin (TBIL), and total albumin (ALB) were indistinguishable between treated and untreated mice (Fig. 7d). These data suggest that hydrodynamic injection of CRISPR components in the liver is well-tolerated in mice.

We also observed no indels in the organs other than in the liver (Additional file 1: Figure S18), consistent with the reports that hydrodynamic injection predominantly delivers nucleic cargos to hepatocytes [16]. We thus focused the further analysis on our target organ, the liver. Of note, the pattern of indels at the *Alb* locus of liver cells was indistinguishable when analyzed 3 weeks vs. 1 year after treatment (compare Additional file 1: Figure S1B to Additional file 1: Figure S18 (treated liver)), suggesting that indels at *Alb* have no deleterious effects on hepatocytes. Mixed results on CRISPR-Cas9-mediated off-target editing have been reported [31, 32]. We analyzed the livers from 5 untreated and treated mice by PCR amplification of putative off-targets followed by deep sequencing. We found no evidence of indels at 20 different genomic targets that were the most likely sites for off-target cleavage (Additional file 1: Figure S19). In addition, immune responses to F8 were undetectable (Fig. 7e).

We also did not observe any changes in the growth or weight over 18 months of observation. In some mice, both BDDF8 and tdTomato were inserted at *Alb*, which allows for imaging analysis of edited cells. Two-photon imaging and 3D reconstruction of the liver sections from HA mice 1 year after hydrodynamic injection showed an even distribution of tdTomato^+^ cells in the liver (Fig. 7f and Additional file 3: Video S2). Of note, we observed clusters of tdTomato^+^ liver cells, suggesting that the edited hepatocytes have divided one to two times in 1year. However, we did not see any large clumps of edited cells, indicative of non-clonal expansion and thereby demonstrating the long-term safety of this approach. To further track the clonal evolution of edited cells, we used ddPCR to detect the copy numbers of NHEJ-mediated knock-in 1 year after treatment (Fig. 7g). Similar to the results obtained at 1 week after treatment, we observed the presence of all types of NHEJ insertions and at similar ratios. These data demonstrate that the insertion of the plasmid backbone or donor sequence in any orientation has no deleterious effects on edited hepatocytes.

## Discussion

In this study, we applied a CRISPR-Cas9 genome editing approach to knock in *BDDF8* at the *Alb* locus and achieved high-level, long-term stable F8 expression. We used hydrodynamic tail-vein injection to prove the principle and also succeeded in delivering editing components using AAV. We found that NHEJ is a more efficient DNA damage repair pathway than HDR in the liver. NHEJ-mediated knock-in at introns makes it possible to identify the best target with a high cleavage efficiency and low off-target effects. As such, NHEJ knock-in at introns may be a favorable editing approach for in vivo gene editing. These studies lay the foundation for further development of the AAV-CRISPR-F8 strategy into clinical therapy.

Several groups have used ZFN or CRISPR-mediated gene targeting at different loci to achieve therapeutic levels of gene expression in hemophilia B mouse models [7–13, 19]. Some studies used ZFN to target intron 1 of the *Alb* gene [7–9], while others targeted right before its stop codon without using a nuclease [19]. Targeting intron 1 or intron 2 of the *F9* gene has also been attempted [12, 13]. Only one earlier study targeted *BDDF8* at intron 1 of the *Alb* gene using ZFN [9], but they observed merely 20–30% F8 activity for a short period. Here, we cured hemophilia A mice by using CRISPR-Cas9-mediated *BDDF8* gene knock-in and achieved life-long stable therapeutic F8 levels of ~ 100%. To our knowledge, this is the first report of successful CRISPR-mediated *F8* targeting in hemophilia A mice. CRISPR-Cas9 has several advantages compared with ZFN or transcription activator-like endonuclease (TALEN) in genetic diseases. First, the straightforward design of sgRNA allows for a rapid screening of large quantities of potential targets. Second, ZFN and TALEN often leave staggered DNA ends, whereas CRISPR-Cas9 leaves clear-cut ends, which favor NHEJ insertion of donor template.

We initially aimed for the precise insertion of the *BDDF8* donor at *Alb*. However, molecular characterization showed that NHEJ played a predominant role in donor integration. This result is in sharp contrast to the almost exclusive editing by HDR in human pluripotent stem cells and other cell lines in culture [14]. The discrepancy could be attributed to different proportions of cells in the active cell cycle. NHEJ is the primary mechanism used to repair DSBs [33], and HDR rarely occurs in post-mitotic adult tissues, such as the skeletal muscle and liver [34]. As such, in order to treat genomic diseases by postnatal genome editing in vivo, we should exploit the NHEJ repair mechanisms after CRISPR-Cas9-mediated dsDNA breaks. To promote unidirectional insertion by NHEJ, Belmonte’s lab proposed a homology-independent targeted integration (HITI) strategy [22]. In this study, we wished to guide HDR insertion using a double-cut donor instead of NHEJ. NHEJ-mediated insertion in the liver is a serendipitous finding. As such, the sgRNA-PAM orientations in the double-cut donor were different from the HITI design. Would we have used the HITI design, we might expect more significant therapeutic effects due to the predominant forward insertion of the donor.

The double-cut donor-mediated HDR editing [14] is also termed homology-mediated end joining (HMEJ) [35]. Both use knock-in templates flanked by sgRNA-PAM sequences with different lengths of homology arms that are released after Cas9-sgRNA cleavage. In our design, we used homology arms of 300–600 bp, whereas the HMEJ approach uses longer homology arms of up to 800 bp. In comparison, microhomology-mediated end joining (MMEJ) uses short homology arms of 5–20 bp. In our experience, MMEJ is much less efficient than HMEJ in guiding HDR editing in mammalian cells.

Many gene therapists have chosen the *Alb* locus for targeted gene insertion to achieve high-level gene expression [9, 19, 36]. One concern is that the insertion of a therapeutic gene may decrease *Alb* expression. Indeed, we observed an ~ 5% decrease in total albumin protein levels in treated mice relative to untreated mice in circulation after 1-year treatment, albeit the difference was not statistically significant (Fig. 7d). Furthermore, reverse insertion of a transgene construct, Cas9-sgRNA, or plasmid backbone may lead to aberrant albumin protein, raising a safety concern. However, the long-term presence of all types of NHEJ insertions in edited liver cells argues against the potential risk of aberrant albumin. In further support of targeting the *Alb* locus in gene therapy, a 2-year follow-up on treated mice found no adverse effects.

Off-target cleavage is a significant safety concern for genome editing. A recent study showed that appropriately designed guide RNAs could direct efficient in vivo editing in mouse livers with no detectable off-target mutations [37]. We evaluated the specificity of sgAlb-E14 using next-generation sequencing validation on the top 20 potential off-target sites identified by a computer algorithm in liver DNA from treated animals. Despite efficient on-target editing, we did not detect any off-target activity, indicating a high specificity of sgAlb-E14 (Additional file 1: Figure S19). Another safety concern is the large deletions [23]. We provided RT-PCR evidence showing that a large deletion is present in a small portion of cells, which leads to the deletion of one extra exon in transcripts. However, we did not observe any deleterious consequences due to the deletion of a large piece surrounding the Cas9-sgRNA cut sites. Since we did not detect off-target cleavage of Cas9-sgAlb, we do not expect appreciable deletions at other sites of the genome.

After dsDNA breaks, AAV preferentially integrates at the break site. However, a few AAV vectors may still be able to randomly integrate into the host genome, albeit at a low frequency [38]. One study showed that genes known to have higher expression in the liver, such as albumin, are hotspots for AAV integration [39]. More recently, high levels of AAV integration into Cas9-induced double-strand breaks were observed in cultured mouse tissues [27]. Moreover, using the Nextera-transposon-based library preparation method for unbiased sequencing, AAV integrations were observed in all treated mice [28]. In our study, we also identified insertions of the AAV-F8 and AAV-Cas9 sequence at CRISPR cleavage sites. Although AAV-mediated expression of Cas9 or integration of Cas9 did not lead to significant genome-wide genotoxicity, persistent expression due to AAV-Cas9 integration may trigger immune depletion of edited cells [40, 41]. Developing strategies to minimize AAV-Cas9 integration is imperative before initiating clinical trials of AAV-CRISPR therapies. Toward that goal, the use of a self-deleting AAV-CRISPR system may be able to effectively remove Cas9 protein in mouse liver while retaining efficient in vivo editing of endogenous targets [42].

Several types of AAV vectors are currently being used in ~ 200 clinical trials, and there has not been any reported increase in the incidence of cancer [43]. These data suggest the safety of AAV vectors in clinical practice. However, further studies are required to examine the safety of AAV-CRISPR therapy for hemophilia A.

## Conclusions

In summary, we have successfully cured hemophilic mice by delivering CRISPR-Cas9 genome-editing tools, using a high-speed injection of plasmids or transfusion of AAV vector in vivo. The life-long stable therapeutic effect is a significant advantage of using AAV-CRISPR-Cas9 over the conventional AAV vector treatment. Long-term follow-up on the AAV-CRISPR-treated HA mice and comprehensive investigation into the safety profiles will be necessary before the application of this technology in treating hemophilia patients.

## Methods

### Cas9-sgRNA plasmid construction

We used the CHOPCHOP website (https://chopchop.rc.fas.harvard.edu/) to design sgRNA targeting the *Alb* stop codon in exon 14 and introns 1, 11, 12, and 13. In most experiments of this work, we used either sgAlb-E14 (GTTGTGATGTGTTTAGGCTA) or sgAlb for simplicity. We cloned the pU6-sgRNA vector using the NEBuilder HiFi DNA Assembly Kit (New England Biolabs). Sequences of all the sgRNAs used in this work are listed in Additional file 1: Table S1. We used humanized Cas9 from *Streptococcus pyogenes* (SpCas9) flanked by nuclear localization sequence (NLS) or Cas9 for simplicity. The pEF1-Cas9-Wpre-PolyA vector has been previously published [44]. AAV-U1a-Cas9 has been used in an earlier study [26]. AAV-BDDF8 was cloned by inserting SA85-E2A-BDDF8-PolyA between inverted terminal repeats of the pAAV2 plasmid. We constructed AAV-sgRNA vectors by PCR amplification of U6-sgRNA and insertion of a 2.5-kb stuffer from the *Alb* enhancer. All the vectors were verified by endonuclease digestion and Sanger sequencing (MCLAB).

### Donor plasmid construction

To construct pDonor plasmids targeting the *Alb* stop codon, the left and right homology arms of 600 bp (HA600-600) were amplified from mouse genomic DNA, with the stop codon removed and in-frame linked with the E2A sequence; the inserted Tomato, BDDF8, F8, or mNeonGreen were amplified from other vectors in the lab by PCR. The sgAlb target sequence, together with the PAM sequence (GTTGTGATGTGTTTAGGCTAAGG), was tagged upstream of the left homology arm and downstream of the right homology arm. In some double-cut donor vectors, we used the sgDocut sequence (GGTGGTGCAGATGAACTCCATGG). Multiple inserts and plasmid backbone were linked together using the NEBuilder HiFi DNA Assembly Kit. pD-mNeonGreen, pD-mNeonGreen-sg (where sg indicated the double-cut design), and pD-BDDF8-sg (HA190-130, HA190-0, HA85-130, HA85-0) were constructed using the same method. Correct clones were identified by endonuclease digestion and Sanger sequencing.

### Hemophilia A mice and hydrodynamic injection

We purchased the hemophilia A (HA) mice bearing an *F8* exon 16 knockout on a 129 × B6 background from the Jackson Laboratory (Bar Harbor, ME), which was initially obtained from Dr. H. Kazazian (University of Pennsylvania) [20]. The mice were housed and maintained at the State Key Laboratory of Experimental Hematology (SKLEH, Tianjin, China). Animal experiments were conducted according to the protocols approved by the Institutional Animal Care and Use Committee of SKLEH and the Institute of Hematology. Vectors for hydrodynamic tail vein injection were prepared using the EndoFree MaxiPrep Kits (Qiagen) or ZymoPURE II Plasmid Maxiprep Kit (Zymo Research). We screened endotoxin using Lyophilized amebocyte lysate LAL/TAL reagent (Xiamen Bioendo Technology) and abandoned endotoxin-contaminated plasmids. Before in vivo injections, we diluted plasmid DNA using sodium lactate Ringer’s solution (China Otsuka Pharmaceutica). For hydrodynamic injection, a volume equivalent to 10% of mouse body weight was administered via the tail vein in 5–6 s into 5–8-week-old HA mice. The amount of plasmid DNA was 10 μg each for Cas9, sgRNA, and pDonor. To prevent bleeding, we injected 0.5 IU F8 protein (Xyntha; Wyeth Pharmaceuticals) in each mouse, together with the editing plasmids.

### Determination of tdTomato^+^ or mNeonGreen^+^ liver cells by flow cytometry

To determine the percentage of liver cells that were tdTomato^+^ or mNeonGreen^+^, small portions of livers were cut off and fixed in a 10% formalin solution for 3–4 h. Fixed livers were ground in PBS and filtered through 70-μm cell strainers. We added DAPI before analyzing the cells on a BD FACS Aria III flow cytometer. At least 1 × 10^5^ events were analyzed to detect the percentage of tdTomato^+^ or mNeonGreen^+^ cells. Liver cells from untreated mice served as a control. To analyze the tdTomato^+^ or mNeonGreen^+^ cells, we first gated the DAPI^+^ cells and then tdTomato^+^ or mNeonGreen^+^ cells using the untreated mouse liver cells as negative controls.

### Blood collection and plasma isolation

For plasma isolation, blood samples were collected by tail vein clipping and bled into a microtube containing 3.2% sodium citrate, which was adjusted to 10% of the blood volume obtained. When 100 μl of blood was collected, styptic powder (Miracle Corp) was used to stop the bleeding. Samples were centrifuged at 2000×*g* for 15 min at 25 °C. The plasma fraction was removed, transferred to a new tube, immediately frozen on dry ice, and stored at − 80 °C. Plasma samples were thawed quickly at 37 °C immediately before measuring F8 bioactivity.

### F8 coagulation assay

We used a one-stage aPTT-based clotting assay to measure the F8 coagulation activity with a Sysmex CA1500 system (Sysmex, Kobe, Japan). Siemens reagents (Siemens; Marburg, Germany) including Dade Actin activated cephaloplast in reagent (Siemens; B4218-1) and coagulation F8-deficient plasma (Siemens; OTXW17) were used. The mouse plasma samples were diluted with Dade Owren’s Veronal Buffer (Siemens; B4234-25) by a factor of 4. The F8 activity was performed by mixing 5 μl of diluted mouse sample plasma with 45 μl of Dade Owren’s Veronal Buffer and 50 μl of F8-deficient plasma and 50 μl of aPTT reagent (Dade Actin activated cephaloplast in reagent), followed by a 120-s incubation at 37 °C. Coagulation was initiated after the addition of 50 μl of 25 mM calcium chloride. Time to clot formation was measured by the Sysmex CA1500 system. A standard curve was prepared by diluting the human calibration plasma (Siemens), and the plasma obtained from wild-type mice served as a positive control.

### F8 inhibitor assay

We used a modified Bethesda assay to determine the titer of F8-neutralizing inhibitors [45–47]. Normal plasma was prepared by mixing 100 μl of F8-deficient plasma with 0.15 IU recombinant F8 protein (Xyntha) to make 100% F8 activity of normal plasma. Mouse plasmas were thawed at 37 °C and incubated at 56 °C for 30 min to inactivate F8 activity. Normal plasma (100 μl) was mixed with an equal volume of either inactivated mouse plasma (test mixture) or 0.1 M imidazole buffer pH 7.4 (control mixture). After 2 h of incubation at 37 °C, the relative percentage of F8 coagulation activity of the test mixture compared to the control mixture (residual F8 coagulation activity) was determined. One Bethesda unit (BU) was defined as the amounts of inhibitors that result in a 50% decrease in residual F8 coagulation activity. When residual F8 activity of the undiluted sample was below 25%, it was retested by diluting in 0.1 M imidazole buffer pH 7.4 until a residual F8:C activity of 25 to 75% was obtained.

### Droplet digital PCR

Genomic DNA was extracted from untreated and treated HA mice. The DNA concentration was determined by Qubit 4 Fluorometer (Thermo Fisher Scientific). As recommended for ddPCR analysis, primers were designed to amplify products of 150–250 bp. The primer and probe sequences are listed in Additional file 1: Table S2. The reaction mixtures contained 2× ddPCR Supermix for Probes (no dUTP) (Bio-Rad, Cat#: 186–3010), primers for target and reference assay (final concentrations of 900 nM each), FAM- or HEX-labeled probes (IDT) for both assays (final concentrations of 250 nM each), and template (100 ng) in a final volume of 20 μl. Standard reagents and consumables supplied by Bio-Rad were used, including cartridges, gaskets, droplet generation oil, and droplet reader oil. After droplet generation, we carefully transferred them to a 24-well PCR plate and sealed the plate with the PX1 PCR Plate Sealer (Bio-Rad). The PCR conditions were 95 °C for 10 min, followed by 40 cycles of 94 °C for 30 s, 60 °C for 1 min, and 98 °C for 10 min, then hold at 4 °C. The ramp rate was set at 2 °C/s. Droplets were read using the QX200 Droplet Reader (Bio-Rad). We included a no-template control (NTC) and negative control for each reaction. Data analysis was conducted using the QuantaSoft Software version 1.7.4.0917. *Actb* was used as a loading control, having a single copy per genome. All DNA samples were run at least twice.

### Verification of NHEJ vs. HDR-mediated knock-in by PCR and Sanger sequencing

Genomic DNA from mouse liver tissue was extracted using DNeasy Blood & Tissue Kit (Qiagen). To distinguish between the HDR- and NHEJ-mediated knock-in, we conducted PCR using one primer that anneals to the genome sequence outside of the homology arm and another targeting the donor-specific sequence. The primers used for this experiment are listed in Additional file 1: Tables S3 and S4. We used the KAPA HiFi HotStart ReadyMix (KAPA Biosystems) for PCR. The cycling conditions were 98 °C for 2 min, followed by 35 cycles of 98 °C for 10 s, 64 °C for 5 s, 68 °C for 5 s, and 72 °C for 15 s. After separation on 1–2% agarose gels, we purified the selected DNA bands using QIAquick Gel Extraction Kit (Qiagen). The purified PCR product was inserted into a pJET1.2 vector (Thermo Fisher). We picked multiple clones for Sanger sequencing.

### Detection of fusion Alb-hF8 transcript by RT-PCR

A 30-mg piece of liver tissue was snap-frozen using liquid nitrogen. The samples were then ground into a fine powder in liquid nitrogen with a pre-chilled mortar and pestle and mixed immediately with RLT (Qiagen RNeasy Mini Kit, Valencia, CA) lysis buffer. DNA was extracted following the manufacturer’s instructions. cDNAs were synthesized using TransScript First-Strand cDNA Synthesis SuperMix (TransGen Biotech) from 1 μg RNA. PCR was performed using KAPA HiFi HotStart Ready Mix (KAPA Biosystems) to detect hybrid murine Alb-hF8 mRNA. The following pairs of primers were used: T237-CTTGGTCAAAACCAACTGTGA, which anneals to exon 10 of *Alb*, and T385-ATCGCAAAAGGCACAGAAAG, which anneals to human *F8*. The cycling condition was 98 °C for 2 min followed by 35 cycles of 98 °C for 10 s, 64 °C for 5 s, 68 °C for 5 s, and 72 °C for 20 s. The PCR products were sequenced.

### Immunosuppression

For transient immunosuppression, cyclophosphamide (50 mg/kg per injection) and/or methylprednisolone (50 mg/kg per injection) was intraperitoneally injected on the day of vector injection, followed by biweekly injections for 3 weeks (seven times in 3 weeks).

### AAV vector packaging, purification, and titering

All the AAV8 vectors were produced by three-plasmid transfection at the SKLEH Vector Core. We performed PEI-based transfections in 15-cm dishes when HEK293T cells reached 80% confluency [48]. The three plasmids were (1) *cis* plasmid pAAV-U1a-Cas9, pAAV-BDDF8 (donor), or pAAV-U6-sgRNA; (2) *trans* plasmids pAAV2/8 containing the AAV2 rep gene and capsid protein genes from AAV8; and (3) adenovirus helper plasmid pHelper (Cell Biolabs). For each microgram of DNA transfected, 2 μg of PEI Max (Polysciences) was used. Plasmids at a ratio of 2:1:1 (20 μg of helper plasmid, 10 μg of AAV *cis* plasmid, 10 μg of *trans* plasmid per plate) were used. Three days after transfection, 10 ml of fresh DMEM-10% FBS was added, and incubation continued for 2 days. We then added 25 units/ml Benzonase (Santa Cruz Biotechnology) and 500 mM NaCl (Sigma). Two hours later, the supernatant was harvested and clarified by centrifugation at 5000×*g* for 10 min. The feedstock was then concentrated by tangential flow filtration (TFF), using a TFF capsule with a 300-kDa molecular weight cutoff (Pall Minimate). A 15-fold concentration of AAV vectors was further purified by ultracentrifugation through an iodixanol density gradient, then concentrated and dialyzed against PBS, as previously described [49].

The physical particle titers (genome copies per milliliter) were determined by droplet digital PCR using primers targeting U6, F8, or Cas9 [50]. Vector aliquots were diluted 10-fold and treated with DNase I (ABM; 400 U/ml) at 37 °C for 30 min, followed by heat inactivation at 95 °C for 5 min. Treated samples were then further diluted 100,000-fold in dilution buffer using nano water with added 0.05% Kolliphor P188 (Sigma; also known as Pluronic F68). The reaction mixtures were assembled with the recommended ddPCR Supermix (Bio-Rad) and template (1 μl) in a final volume of 20 μl. The positive or negative droplets were read from the QX200 reader.

### Tail vein injection of AAV

All AAV vectors used in this study passed an endotoxin assay using the amebocyte lysate LAL/TAL reagent (Xiamen Bioendo Technology). We injected hemophilia A mice with 1 × 10^11^ AAV8-Cas9, 1 × 10^11^ AAV8-sgRNA, and 5 × 10^11^ AAV8-BDDF8. Injection with AAV8-BDDF8 donor only served as a control. AAV vectors were diluted to 200 μl in phosphate-buffered saline plus 0.001% Kolliphor P188 before the tail vein injection.

### Detection of AAV vector integration by PCR and Sanger sequencing

To determine the AAV vector integration at the gene editing site mediated by AAV-CRISPR-Cas9, we extracted genomic DNA from mouse liver using DNeasy Blood & Tissue Kit (Qiagen). One primer was designed to anneal to the Alb target site and the other primer to Cas9 or BDDF8. Additional file 1: Table S5 lists the primer sequences for this study. We used the KAPA HiFi HotStart ReadyMix (KAPA Biosystems) for PCR. To successfully amplify AAV ITR-containing sequences, we added 5–10% DMSO to the PCR mix. The cycling conditions were 98 °C for 2 min, followed by 35 cycles of 98 °C for 10 s, 64 °C for 5 s, 68 °C for 5 s, and 72 °C for 30 s. We cloned PCR products into the pJET1.2 vector (Thermo Fisher), followed by Sanger sequencing of ~ 10 clones for each product.

### Analysis of liver damage markers

After blood collection by tail vein puncture, serum coagulated naturally at room temperature for 1 h. Samples were then centrifuged at 2000×*g* for 20 min at 25 °C. The serum fraction was removed, transferred to a new tube, and immediately stored at − 80 °C. Serum samples were thawed quickly at 37 °C immediately before measuring. We used diagnostic assay kits (Beckman Coulter, Inc. Teco Diagnostics) to determine alanine aminotransferase (ALT), aspartate aminotransferase (AST), bilirubin, and total albumin (Alb) levels in the serum.

### Tail-clip challenge assay

The phenotypic correction of hemophilia was assessed by the tail clip survival test as previously described [51]. The tails of anesthetized HA mice were clipped at a diameter of 1.5 mm, without subsequent cauterization. After the procedure, we checked the mice every 4–8 h. Clot formation and survival beyond 24 h were used to indicate the correction of the murine hemophilia A phenotype.

### On-target and off-target analyses by deep sequencing

Genomic DNA from mouse livers and other organs was isolated using the DNeasy Blood & Tissue Kit (Qiagen). DNA samples from treated and untreated hemophilia mice were utilized for on-target and off-target analyses. The top 20 putative off-target sites of sgAlb were predicted using the COSMID Tool (http://crispr.bme.gatech.edu). Primers were designed using Primer3Plus to amplify fragments of 240–285 bp surrounding the on-target and off-target sequences (Additional file 1: Tables S6 and S7). The target sequences were amplified with KAPA HiFi DNA polymerase. The PCR cycling conditions were 98 °C for 2 min, followed by 30 cycles of 98 °C for 5 s, 64 °C for 10 s, and 72 °C for 5 s. All the amplicons from the same sample were mixed for 150 bp paired-end sequencing on the Illumina HiSeq X Ten (Novogene Co., Ltd). The outputs were analyzed using our optimized pipeline. Briefly, high-quality reads (*Q* score > 30) were uploaded to the Galaxy platform (https://usegalaxy.org/) [52]. After processing with fast length adjustment of short reads (FLASH) and Barcode Splitter, the demultiplexed data were analyzed using Cas-Analyzer (http://www.rgenome.net/cas-analyzer/#!) [53]. The analyzed data were transferred to MS Excel files, trimmed, and further processed using Visual Basic for Applications (VBA). For clarity, we only listed the top 10 indel patterns. For original Illumina sequencing data, please contact the corresponding authors.

### Multiphoton imaging and 3D reconstruction of edited liver tissue

At 3 weeks or 1 year after hydrodynamic injection of editing plasmid, we injected hemophilia A mice with 50 μl of APC-conjugated VE-cadherin antibody (APC anti-mouse CD144; Biolegend) through the tail vein. Mice were euthanized to harvest the liver tissue 5 min later. Small pieces from different liver lobes were immediately fixed in ice-cold 4% paraformaldehyde solution for 6–8 h. The liver pieces were then washed with PBS, immersed in 30% sucrose overnight, frozen in optimal cutting temperature (OCT) compound (TissueTek), and stored at − 80 °C. We generated 600–700-μm-thick sections using a CM1850 Cryostat (Leica) at − 20 °C. For staining, sectioned chunks were re-hydrated in PBS, the nuclei were labeled with DAPI for 10 min at room temperature, then the section was mounted on a chamber slide for imaging. High-resolution images were taken using the Olympus FV1200MPE multiphoton laser scanning microscope equipped with a water immersion lens (× 20, N.A. = 1.05). A high-precision motorized stage was used to collect the large-scale 3D mosaics using 405 nm, 561 nm, and 640 nm solid-state lasers. The boundaries (in *x*, *y*, and *z*) of the tissue section were defined using the Multi-Area Time Lapse function of the ASW microscope operating-software provided by Olympus. The software automatically generated a list of 3D stage positions covering the volume of interest. Individual image tiles were 512 × 512 with a pixel dimension of 0.62 μm, with an overlap between two adjacent images (*x*–*y*) being 10% and each *z* stack acquired in 2-μm steps (about 200 μm depth in total). Images were processed and 3D-reconstructed using the Imaris software (Bitplane).

### Statistical analysis

We used GraphPad Prism 7.0 (GraphPad Software, San Diego, CA) for the preparation of figures and statistical analysis. The mean ± SEM was determined for each treatment group in the individual experiments. We used two-tailed Welch’s paired *t* test or Welch’s unpaired *t* test to determine the significances between the treatment and control groups. A comparison among multiple groups was analyzed by one-way analysis of variance (ANOVA) followed by Tukey’s multiple comparison test. A *P* value < 0.05 was considered significant.

## Supplementary information

**Additional file 1:** **Figure S1.** Efficient cleavage of Cas9-sgAlb-E14 at the *Alb* stop codon. **Figure S2.** High-level knock-in efficiency mediated by the double-cut donor. **Figure S3.** PCR and sequencing analysis confirmed *BDDF8* knock-in at *Alb* stop codon mediated by both NHEJ and HDR. **Figure S4.** Schematic of genome editing at the *Alb* stop codon. Knock-in of promoterless *BDDF8* expression cassette at *Alb* through NHEJ was achieved by Cas9-sgAlb-mediated simultaneous cleavage of the genome and the double-cut donor pD-BDDF8-sg. **Figure S5.** A representative diagram of ddPCR analysis of the copy number of *Actb*. **Figure S6.** Junction sequences after NHEJ integration of *BDDF8* donor at the *Alb* stop codon. **Figure S7.** Sanger sequencing demonstrates the integration of *BDDF8* by NHEJ and HDR. **Figure S8.** Editing with all five pDonor forms correct fusion transcript. **Figure S9.** Indel patterns and cleavage efficiencies of ten sgRNAs used in this study. **Figure S10.** Targeting intron 11, intron 12, and intron 13 led to expected fusion transcripts. **Figure S11.** Insertion of plasmid backbone at intron 12. **Figure S12.** Characterization of insertion of AAV-BDDF8 and AAV-Cas9 at double-strand break (DSB). **Figure S13.** Analysis of AAV-BDDF8 and AAV-Cas9 at Alb-Intron13-371 and Alb-Intron13-527. **Figure S14.** Immune responses against F8 after CRISPR-mediated insertion of *BDDF8*. **Figure S15.** Positive controls for humoral response to F8. **Figure S16.** Long-term stable expression of F8 in hemophilia A mice after immunosuppression treatment. **Figure S17.** Long-term stable F8 activity after CRISPR-BDDF8 treatment. **Figure S18.** Genome editing only occurs in the liver after hydrodynamic injection of CRISPR-Cas9 and donor plasmids. **Figure S19.** Representative deep sequencing results of off-target cleavage. **Table S1.** Target sequences of sgRNAs. **Table S2.** The primer and probe sequences used in ddPCR. **Table S3.** The primers for amplifying the NHEJ junctions. **Table S4.** The primers used for verification of NHEJ vs. HDR-mediated knock-in. **Table S5.** The primers used for verification of AAV insertion at DSB loci in intron13. **Table S6.** Primers used for determining cleavage efficiencies of sgRNAs. **Table S7.** Primers for on-target and off-target analysis of sgAlb-E14.

**Additional file 2.** Video S1. (3 weeks, related to Fig. 1f).

**Additional file 3.** Video S2. (1 year, related to Fig. 7f).

**Additional file 4.** Review history.

## Data Availability

Deep sequencing data are available under BioProject ID PRJNA591714 [54] (https://www.ncbi.nlm.nih.gov/bioproject/591714).

## References

[1] Roth DA, Tawa NE, O’Brien JM, Treco DA, Selden RF (2001). Nonviral transfer of the gene encoding coagulation factor VIII in patients with severe hemophilia A. N Engl J Med.

[2] Hoyer LW, Scandella D (1994). Factor VIII inhibitors: structure and function in autoantibody and hemophilia A patients. Semin Hematol.

[3] George LA, Sullivan SK, Giermasz A, Rasko JEJ, Samelson-Jones BJ, Ducore J, Cuker A, Sullivan LM, Majumdar S, Teitel J (2017). Hemophilia B gene therapy with a high-specific-activity factor IX variant. N Engl J Med.

[4] Vehar GA, Keyt B, Eaton D, Rodriguez H, O’Brien DP, Rotblat F, Oppermann H, Keck R, Wood WI, Harkins RN (1984). Structure of human factor VIII. Nature.

[5] Toole JJ, Pittman DD, Orr EC, Murtha P, Wasley LC, Kaufman RJ (1986). A large region (approximately equal to 95 kDa) of human factor VIII is dispensable for in vitro procoagulant activity. Proc Natl Acad Sci U S A.

[6] Rangarajan S, Walsh L, Lester W, Perry D, Madan B, Laffan M, Yu H, Vettermann C, Pierce GF, Wong WY, Pasi KJ (2017). AAV5-factor VIII gene transfer in severe hemophilia A. N Engl J Med.

[7] Li H, Haurigot V, Doyon Y, Li T, Wong SY, Bhagwat AS, Malani N, Anguela XM, Sharma R, Ivanciu L (2011). In vivo genome editing restores haemostasis in a mouse model of haemophilia. Nature.

[8] Anguela XM, Sharma R, Doyon Y, Miller JC, Li H, Haurigot V, Rohde ME, Wong SY, Davidson RJ, Zhou S (2013). Robust ZFN-mediated genome editing in adult hemophilic mice. Blood.

[9] Sharma R, Anguela XM, Doyon Y, Wechsler T, DeKelver RC, Sproul S, Paschon DE, Miller JC, Davidson RJ, Shivak D (2015). In vivo genome editing of the albumin locus as a platform for protein replacement therapy. Blood.

[10] Guan Y, Ma Y, Li Q, Sun Z, Ma L, Wu L, Wang L, Zeng L, Shao Y, Chen Y (2016). CRISPR/Cas9-mediated somatic correction of a novel coagulator factor IX gene mutation ameliorates hemophilia in mouse. EMBO Mol Med.

[11] Huai C, Jia C, Sun R, Xu P, Min T, Wang Q, Zheng C, Chen H, Lu D (2017). CRISPR/Cas9-mediated somatic and germline gene correction to restore hemostasis in hemophilia B mice. Hum Genet.

[12] Ohmori T, Nagao Y, Mizukami H, Sakata A, Muramatsu SI, Ozawa K, Tominaga SI, Hanazono Y, Nishimura S, Nureki O, Sakata Y (2017). CRISPR/Cas9-mediated genome editing via postnatal administration of AAV vector cures haemophilia B mice. Sci Rep.

[13] Wang L, Yang Y, Breton CA, White J, Zhang J, Che Y, Saveliev A, McMenamin D, He Z, Latshaw C (2019). CRISPR/Cas9-mediated in vivo gene targeting corrects hemostasis in newborn and adult factor IX-knockout mice. Blood.

[14] Zhang JP, Li XL, Li GH, Chen W, Arakaki C, Botimer GD, Baylink D, Zhang L, Wen W, Fu YW (2017). Efficient precise knockin with a double cut HDR donor after CRISPR/Cas9-mediated double-stranded DNA cleavage. Genome Biol.

[15] Xiao W, Berta SC, Lu MM, Moscioni AD, Tazelaar J, Wilson JM (1998). Adeno-associated virus as a vector for liver-directed gene therapy. J Virol.

[16] Liu F, Song Y, Liu D (1999). Hydrodynamics-based transfection in animals by systemic administration of plasmid DNA. Gene Ther.

[17] Tiegs G, Lohse AW (2010). Immune tolerance: what is unique about the liver. J Autoimmun.

[18] Fahs SA, Hille MT, Shi Q, Weiler H, Montgomery RR (2014). A conditional knockout mouse model reveals endothelial cells as the principal and possibly exclusive source of plasma factor VIII. Blood.

[19] Barzel A, Paulk NK, Shi Y, Huang Y, Chu K, Zhang F, Valdmanis PN, Spector LP, Porteus MH, Gaensler KM, Kay MA (2015). Promoterless gene targeting without nucleases ameliorates haemophilia B in mice. Nature.

[20] Bi L, Lawler AM, Antonarakis SE, High KA, Gearhart JD, Kazazian HH (1995). Targeted disruption of the mouse factor VIII gene produces a model of haemophilia A. Nat Genet.

[21] Kim JH, Lee SR, Li LH, Park HJ, Park JH, Lee KY, Kim MK, Shin BA, Choi SY (2011). High cleavage efficiency of a 2A peptide derived from porcine teschovirus-1 in human cell lines, zebrafish and mice. PLoS One.

[22] Suzuki K, Tsunekawa Y, Hernandez-Benitez R, Wu J, Zhu J, Kim EJ, Hatanaka F, Yamamoto M, Araoka T, Li Z (2016). In vivo genome editing via CRISPR/Cas9 mediated homology-independent targeted integration. Nature.

[23] Kosicki M, Tomberg K, Bradley A (2018). Repair of double-strand breaks induced by CRISPR-Cas9 leads to large deletions and complex rearrangements. Nat Biotechnol.

[24] Desmet FO, Hamroun D, Lalande M, Collod-Beroud G, Claustres M, Beroud C (2009). Human Splicing Finder: an online bioinformatics tool to predict splicing signals. Nucleic Acids Res.

[25] Sarkar R, Tetreault R, Gao G, Wang L, Bell P, Chandler R, Wilson JM, Kazazian HH (2004). Total correction of hemophilia A mice with canine FVIII using an AAV 8 serotype. Blood.

[26] Wang D, Li J, Song CQ, Tran K, Mou H, Wu PH, Tai PWL, Mendonca CA, Ren L, Wang BY (2018). Cas9-mediated allelic exchange repairs compound heterozygous recessive mutations in mice. Nat Biotechnol.

[27] Hanlon KS, Kleinstiver BP, Garcia SP, Zaborowski MP, Volak A, Spirig SE, Muller A, Sousa AA, Tsai SQ, Bengtsson NE (2019). High levels of AAV vector integration into CRISPR-induced DNA breaks. Nat Commun.

[28] Nelson CE, Wu Y, Gemberling MP, Oliver ML, Waller MA, Bohning JD, Robinson-Hamm JN, Bulaklak K, Castellanos Rivera RM, Collier JH (2019). Long-term evaluation of AAV-CRISPR genome editing for Duchenne muscular dystrophy. Nat Med.

[29] Witmer C, Young G (2013). Factor VIII inhibitors in hemophilia A: rationale and latest evidence. Ther Adv Hematol.

[30] Matsui H, Fujimoto N, Sasakawa N, Ohinata Y, Shima M, Yamanaka S, Sugimoto M, Hotta A (2014). Delivery of full-length factor VIII using a piggyBac transposon vector to correct a mouse model of hemophilia A. PLoS One.

[31] Zuo E, Sun Y, Wei W, Yuan T, Ying W, Sun H, Yuan L, Steinmetz LM, Li Y, Yang H. Cytosine base editor generates substantial off-target single-nucleotide variants in mouse embryos. Science. 2019;364(6437):289–92. 10.1126/science.aav9973 PMC7301308 30819928

[32] Schaefer KA, Wu WH, Colgan DF, Tsang SH, Bassuk AG, Mahajan VB (2017). Unexpected mutations after CRISPR-Cas9 editing in vivo. Nat Methods.

[33] Vartak SV, Raghavan SC (2015). Inhibition of nonhomologous end joining to increase the specificity of CRISPR/Cas9 genome editing. FEBS J.

[34] Hsu PD, Lander ES, Zhang F (2014). Development and applications of CRISPR-Cas9 for genome engineering. Cell.

[35] Yao X, Wang X, Hu X, Liu Z, Liu J, Zhou H, Shen X, Wei Y, Huang Z, Ying W (2017). Homology-mediated end joining-based targeted integration using CRISPR/Cas9. Cell Res.

[36] Laoharawee K, DeKelver RC, Podetz-Pedersen KM, Rohde M, Sproul S, Nguyen HO, Nguyen T, St Martin SJ, Ou L, Tom S (2018). Dose-dependent prevention of metabolic and neurologic disease in murine MPS II by ZFN-mediated in vivo genome editing. Mol Ther.

[37] Akcakaya P, Bobbin ML, Guo JA, Malagon-Lopez J, Clement K, Garcia SP, Fellows MD, Porritt MJ, Firth MA, Carreras A (2018). In vivo CRISPR editing with no detectable genome-wide off-target mutations. Nature.

[38] Nakai H, Yant SR, Storm TA, Fuess S, Meuse L, Kay MA (2001). Extrachromosomal recombinant adeno-associated virus vector genomes are primarily responsible for stable liver transduction in vivo. J Virol.

[39] Chandler RJ, LaFave MC, Varshney GK, Trivedi NS, Carrillo-Carrasco N, Senac JS, Wu W, Hoffmann V, Elkahloun AG, Burgess SM, Venditti CP (2015). Vector design influences hepatic genotoxicity after adeno-associated virus gene therapy. J Clin Invest.

[40] Wagner DL, Amini L, Wendering DJ, Burkhardt LM, Akyuz L, Reinke P, Volk HD, Schmueck-Henneresse M (2019). High prevalence of Streptococcus pyogenes Cas9-reactive T cells within the adult human population. Nat Med.

[41] Charlesworth CT, Deshpande PS, Dever DP, Camarena J, Lemgart VT, Cromer MK, Vakulskas CA, Collingwood MA, Zhang L, Bode NM (2019). Identification of preexisting adaptive immunity to Cas9 proteins in humans. Nat Med.

[42] Li A, Lee CM, Hurley AE, Jarrett KE, De Giorgi M, Lu W, Balderrama KS, Doerfler AM, Deshmukh H, Ray A (2019). A self-deleting AAV-CRISPR system for in vivo genome editing. Mol Ther Methods Clin Dev.

[43] Srivastava A, Carter BJ (2017). AAV infection: protection from cancer. Hum Gene Ther.

[44] Li XL, Li GH, Fu J, Fu YW, Zhang L, Chen W, Arakaki C, Zhang JP, Wen W, Zhao M (2018). Highly efficient genome editing via CRISPR-Cas9 in human pluripotent stem cells is achieved by transient BCL-XL overexpression. Nucleic Acids Res.

[45] Kasper CK, Aledort L, Aronson D, Counts R, Edson JR, van Eys J, Fratantoni J, Green D, Hampton J, Hilgartner M (1975). Proceedings: a more uniform measurement of factor VIII inhibitors. Thromb Diath Haemorrh.

[46] Verbruggen B, Novakova I, Wessels H, Boezeman J, van den Berg M, Mauser-Bunschoten E (1995). The Nijmegen modification of the Bethesda assay for factor VIII:C inhibitors: improved specificity and reliability. Thromb Haemost.

[47] Miller CH, Platt SJ, Rice AS, Kelly F, Soucie JM, Hemophilia Inhibitor Research Study I (2012). Validation of Nijmegen-Bethesda assay modifications to allow inhibitor measurement during replacement therapy and facilitate inhibitor surveillance. J Thromb Haemost.

[48] Fripont S, Marneffe C, Marino M, Rincon MY, Holt MG. Production, purification, and quality control for adeno-associated virus-based vectors. J Vis Exp. 2019;(143). 10.3791/58960. 10.3791/58960 30774140

[49] Lock M, Alvira M, Vandenberghe LH, Samanta A, Toelen J, Debyser Z, Wilson JM (2010). Rapid, simple, and versatile manufacturing of recombinant adeno-associated viral vectors at scale. Hum Gene Ther.

[50] Lock M, Alvira MR, Chen SJ, Wilson JM (2014). Absolute determination of single-stranded and self-complementary adeno-associated viral vector genome titers by droplet digital PCR. Hum Gene Ther Methods.

[51] Connelly S, Andrews JL, Gallo AM, Kayda DB, Qian J, Hoyer L, Kadan MJ, Gorziglia MI, Trapnell BC, McClelland A, Kaleko M (1998). Sustained phenotypic correction of murine hemophilia A by in vivo gene therapy. Blood.

[52] Afgan E, Baker D, Batut B, van den Beek M, Bouvier D, Cech M, Chilton J, Clements D, Coraor N, Gruning BA (2018). The Galaxy platform for accessible, reproducible and collaborative biomedical analyses: 2018 update. Nucleic Acids Res.

[53] Park J, Lim K, Kim JS, Bae S (2017). Cas-analyzer: an online tool for assessing genome editing results using NGS data. Bioinformatics.

[54] Zhang JP, Cheng XX, Zhao M, Li GH, Xu J, Zhang F, Yin MD, Meng FY, Dai XY, Fu YW, Yang ZX, Arakaki, C, Su RJ, Wen W, Wang WT, Chen WQ, Choi H, Wang C, Gao GP, Zhang L, Cheng T, Zhang XB. Curing hemophilia A by NHEJ-mediated ectopic F8 insertion in the mouse. https://www.ncbi.nlm.nih.gov/bioproject/591714. Accessed 26 Nov 2019. 10.1186/s13059-019-1907-9 PMC6912951 31843008

