# Supplementary material for: Curing hemophilia A by NHEJ-mediated ectopic F8 insertion in the mouse
Source: Genome Biol. 2019 Dec 16;20:276. doi: 10.1186/s13059-019-1907-9 (PMC6912951; doi:10.1186/s13059-019-1907-9)
Supplement: Supplementary file 1 — Additional file 1: Figure S1. Efficient cleavage of Cas9-sgAlb-E14 at the Alb stop codon. Figure S2. High-level knock-in efficiency mediated by the double-cut donor. Figure S3. PCR and sequencing analysis confirmed BDDF8 knock-in at Alb stop codon mediated by both NHEJ and HDR. Figure S4. Schematic of genome editing at the Alb stop codon. Knock-in of promoterless BDDF8 expression cassette at Alb through NHEJ was achieved by Cas9-sgAlb-mediated simultaneous cleavage of the genome and the double-cut donor pD-BDDF8-sg. Figure S5. A representative diagram of ddPCR analysis of the copy number of Actb. Figure S6. Junction sequences after NHEJ integration of BDDF8 donor at the Alb stop codon. Figure S7. Sanger sequencing demonstrates the integration of BDDF8 by NHEJ and HDR. Figure S8. Editing with all five pDonor forms correct fusion transcript. Figure S9. Indel patterns and cleavage efficiencies of ten sgRNAs used in this study. Figure S10. Targeting intron 11, intron 12, and intron 13 led to expected fusion transcripts. Figure S11. Insertion of plasmid backbone at intron 12. Figure S12. Characterization of insertion of AAV-BDDF8 and AAV-Cas9 at double-strand break (DSB). Figure S13. Analysis of AAV-BDDF8 and AAV-Cas9 at Alb-Intron13-371 and Alb-Intron13-527. Figure S14. Immune responses against F8 after CRISPR-mediated insertion of BDDF8. Figure S15. Positive controls for humoral response to F8. Figure S16. Long-term stable expression of F8 in hemophilia A mice after immunosuppression treatment. Figure S17. Long-term stable F8 activity after CRISPR-BDDF8 treatment. Figure S18. Genome editing only occurs in the liver after hydrodynamic injection of CRISPR-Cas9 and donor plasmids. Figure S19. Representative deep sequencing results of off-target cleavage. Table S1. Target sequences of sgRNAs. Table S2. The primer and probe sequences used in ddPCR. Table S3. The primers for amplifying the NHEJ junctions. Table S4. The primers used for verification of NHEJ vs. HDR-mediated k [file 13059_2019_1907_MOESM1_ESM.pptx]

## Slide 1
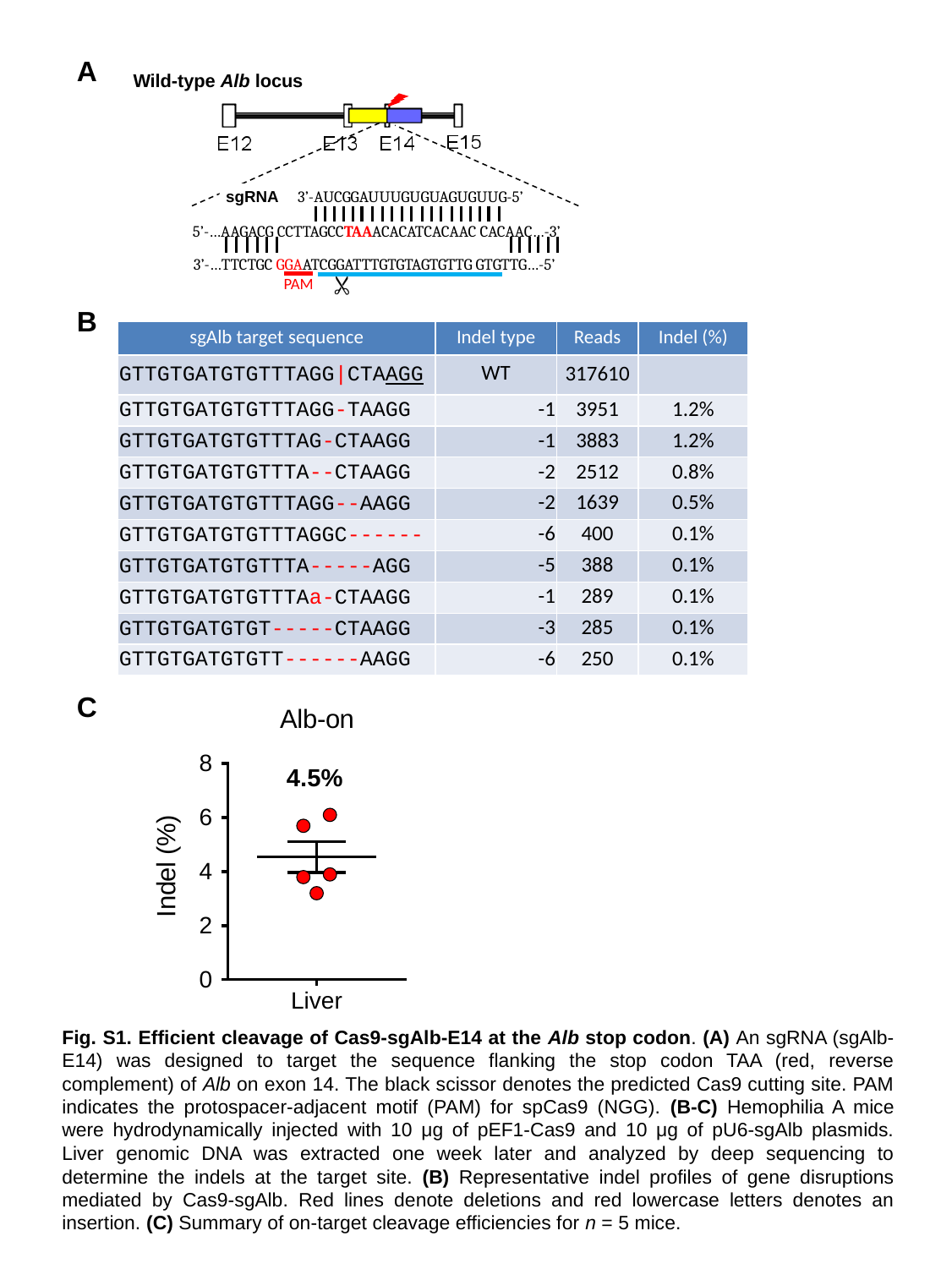

A
Wild-type Alb locus
3’-AUCGGAUUUGUGUAGUGUUG-5’
sgRNA
5’-…AAGACG CCTTAGCCTAAACACATCACAAC CACAAC…-3’
3’-…TTCTGC GGAATCGGATTTGTGTAGTGTTG GTGTTG…-5’
PAM
B
| sgAlb target sequence | Indel type | Reads | Indel (%) |
| --- | --- | --- | --- |
| GTTGTGATGTGTTTAGG|CTAAGG | WT | 317610 | |
| GTTGTGATGTGTTTAGG-TAAGG | -1 | 3951 | 1.2% |
| GTTGTGATGTGTTTAG-CTAAGG | -1 | 3883 | 1.2% |
| GTTGTGATGTGTTTA--CTAAGG | -2 | 2512 | 0.8% |
| GTTGTGATGTGTTTAGG--AAGG | -2 | 1639 | 0.5% |
| GTTGTGATGTGTTTAGGC------ | -6 | 400 | 0.1% |
| GTTGTGATGTGTTTA-----AGG | -5 | 388 | 0.1% |
| GTTGTGATGTGTTTAa-CTAAGG | -1 | 289 | 0.1% |
| GTTGTGATGTGT-----CTAAGG | -3 | 285 | 0.1% |
| GTTGTGATGTGTT------AAGG | -6 | 250 | 0.1% |
C
4.5%
Fig. S1. Efficient cleavage of Cas9-sgAlb-E14 at the Alb stop codon. (A) An sgRNA (sgAlb-E14) was designed to target the sequence flanking the stop codon TAA (red, reverse complement) of Alb on exon 14. The black scissor denotes the predicted Cas9 cutting site. PAM indicates the protospacer-adjacent motif (PAM) for spCas9 (NGG). (B-C) Hemophilia A mice were hydrodynamically injected with 10 μg of pEF1-Cas9 and 10 μg of pU6-sgAlb plasmids. Liver genomic DNA was extracted one week later and analyzed by deep sequencing to determine the indels at the target site. (B) Representative indel profiles of gene disruptions mediated by Cas9-sgAlb. Red lines denote deletions and red lowercase letters denotes an insertion. (C) Summary of on-target cleavage efficiencies for n = 5 mice.

## Slide 2
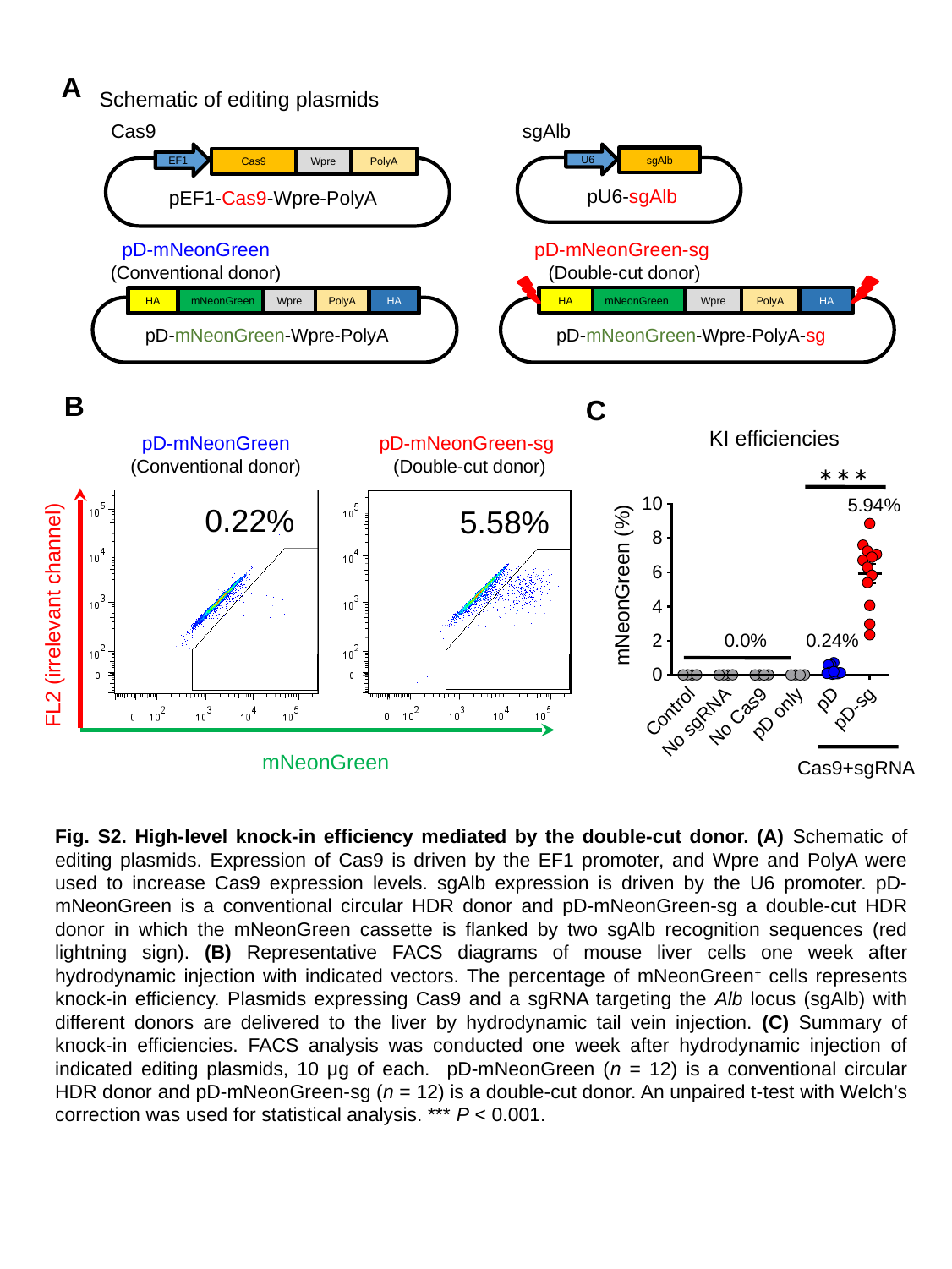

A
Schematic of editing plasmids
Cas9
sgAlb
EF1
Cas9
Wpre
PolyA
pEF1-Cas9-Wpre-PolyA
U6
sgAlb
pU6-sgAlb
pD-mNeonGreen
(Conventional donor)
pD-mNeonGreen-sg
(Double-cut donor)
HA
mNeonGreen
Wpre
PolyA
HA
pD-mNeonGreen-Wpre-PolyA-sg
HA
mNeonGreen
Wpre
PolyA
HA
pD-mNeonGreen-Wpre-PolyA
B
C
KI efficiencies
pD-mNeonGreen
(Conventional donor)
pD-mNeonGreen-sg
(Double-cut donor)
***
0.22%
5.58%
FL2 (irrelevant channel)
mNeonGreen
Cas9+sgRNA
Fig. S2. High-level knock-in efficiency mediated by the double-cut donor. (A) Schematic of editing plasmids. Expression of Cas9 is driven by the EF1 promoter, and Wpre and PolyA were used to increase Cas9 expression levels. sgAlb expression is driven by the U6 promoter. pD-mNeonGreen is a conventional circular HDR donor and pD-mNeonGreen-sg a double-cut HDR donor in which the mNeonGreen cassette is flanked by two sgAlb recognition sequences (red lightning sign). (B) Representative FACS diagrams of mouse liver cells one week after hydrodynamic injection with indicated vectors. The percentage of mNeonGreen+ cells represents knock-in efficiency. Plasmids expressing Cas9 and a sgRNA targeting the Alb locus (sgAlb) with different donors are delivered to the liver by hydrodynamic tail vein injection. (C) Summary of knock-in efficiencies. FACS analysis was conducted one week after hydrodynamic injection of indicated editing plasmids, 10 μg of each. pD-mNeonGreen (n = 12) is a conventional circular HDR donor and pD-mNeonGreen-sg (n = 12) is a double-cut donor. An unpaired t-test with Welch’s correction was used for statistical analysis. *** P < 0.001.

## Slide 3
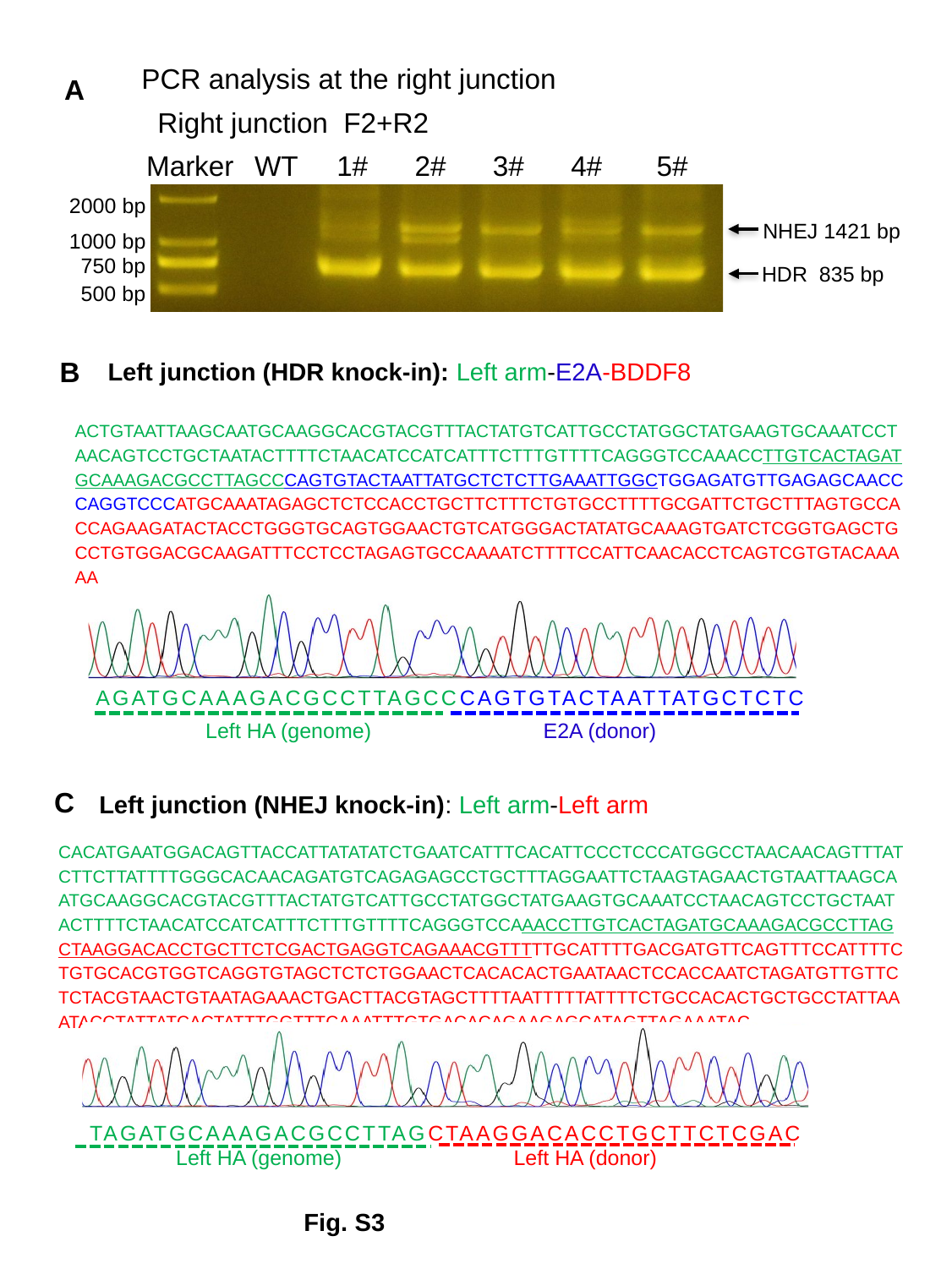

PCR analysis at the right junction
A
Right junction F2+R2
Marker
WT 1# 2# 3# 4# 5#
2000 bp
1000 bp
750 bp
500 bp
NHEJ 1421 bp
HDR 835 bp
B
Left junction (HDR knock-in): Left arm-E2A-BDDF8
actgtaattaagcaatgcaaggcacgtacgtttactatgtcattgcctatggctatgaagtgcaaatcctaacagtcctgctaatacttttctaacatccatcatttctttgttttcagggtccaaaccttgtcactagatgcaaagacgccttagccCAGTGTACTAATTATGCTCTCTTGAAATTGGCTGGAGATGTTGAGAGCAACCCAGGTCCCAtgcaaatagagctctccacctgcttctttctgtgccttttgcgattctgctttagtgccaccagaagatactacctgggtgcagtggaactgtcatgggactatatgcaaagtgatctcggtgagctgcctgtggacgcaagatttcctcctagagtgccaaaatcttttccattcaacacctcagtcgtgtacaaaaa
AGATGCAAAGACGCCTTAGCCCAGTGTACTAATTATGCTCTC
Left HA (genome) E2A (donor)
C
Left junction (NHEJ knock-in): Left arm-Left arm
CACATGAATGGACAGTTACCATTATATATCTGAATCATTTCACATTCCCTCCCATGGCCTAACAACAGTTTATCTTCTTATTTTGGGCACAACAGATGTCAGAGAGCCTGCTTTAGGAATTCTAAGTAGAACTGTAATTAAGCAATGCAAGGCACGTACGTTTACTATGTCATTGCCTATGGCTATGAAGTGCAAATCCTAACAGTCCTGCTAATACTTTTCTAACATCCATCATTTCTTTGTTTTCAGGGTCCAAACCTTGTCACTAGATGCAAAGACGCCTTAGctaaggacacctgcttctcgactgaggtcagaaacgtttttgcattttgacgatgttcagtttccattttctgtgcacgtggtcaggtgtagctctctggaactcacacactgaataactccaccaatctagatgttgttctctacgtaactgtaatagaaactgacttacgtagcttttaatttttattttctgccacactgctgcctattaaatacctattatcactatttggtttcaaatttgtgacacagaagagcatagttagaaatac
TAGATGCAAAGACGCCTTAGCTAAGGACACCTGCTTCTCGAC
Left HA (genome) Left HA (donor)
Fig. S3

## Slide 4
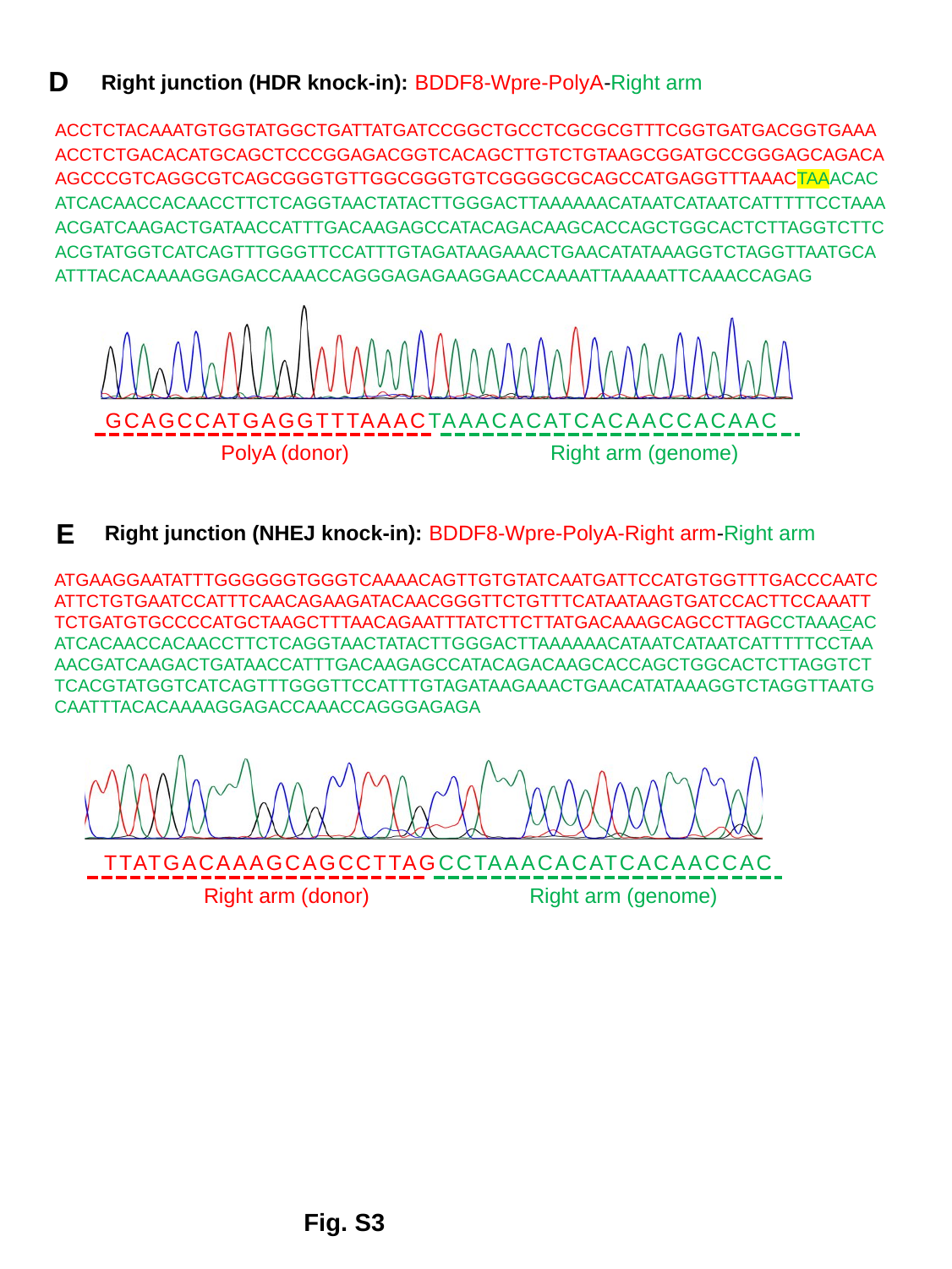

D
Right junction (HDR knock-in): BDDF8-Wpre-PolyA-Right arm
ACCTCTACAAATGTGGTATGGCTGATTATGATCCGGCTGCCTCGCGCGTTTCGGTGATGACGGTGAAAACCTCTGACACATGCAGCTCCCGGAGACGGTCACAGCTTGTCTGTAAGCGGATGCCGGGAGCAGACAAGCCCGTCAGGCGTCAGCGGGTGTTGGCGGGTGTCGGGGCGCAGCCATGAGGTTTAAACTAAACacatcacaaccacaaccttctcaggtaactatacttgggacttaaaaaacataatcataatcatttttcctaaaacgatcaagactgataaccatttgacaagagccatacagacaagcaccagctggcactcttaggtcttcacgtatggtcatcagtttgggttccatttgtagataagaaactgaacatataaaggtctaggttaatgcaatttacacaaaaggagaccaaaccagggagagaaggaaccaaaattaaaaattcaaaccagag
GCAGCCATGAGGTTTAAACTAAACACATCACAACCACAAC
PolyA (donor) Right arm (genome)
E
Right junction (NHEJ knock-in): BDDF8-Wpre-PolyA-Right arm-Right arm
ATGAAGGAATATTTGGGGGGTGGGTCAAAACAGTTGTGTATCAATGATTCCATGTGGTTTGACCCAATCATTCTGTGAATCCATTTCAACAGAAGATACAACGGGTTCTGTTTCATAATAAGTGATCCACTTCCAAATTTCTGATGTGCCCCATGCTAAGCTTTAACAGAATTTATCTTCTTATGACAAAGCAGCCTTAGccTAAACacatcacaaccacaaccttctcaggtaactatacttgggacttaaaaaacataatcataatcatttttcctaaaacgatcaagactgataaccatttgacaagagccatacagacaagcaccagctggcactcttaggtcttcacgtatggtcatcagtttgggttccatttgtagataagaaactgaacatataaaggtctaggttaatgcaatttacacaaaaggagaccaaaccagggagaga
TTATGACAAAGCAGCCTTAGCCTAAACACATCACAACCAC
Right arm (donor) Right arm (genome)
Fig. S3

## Slide 5
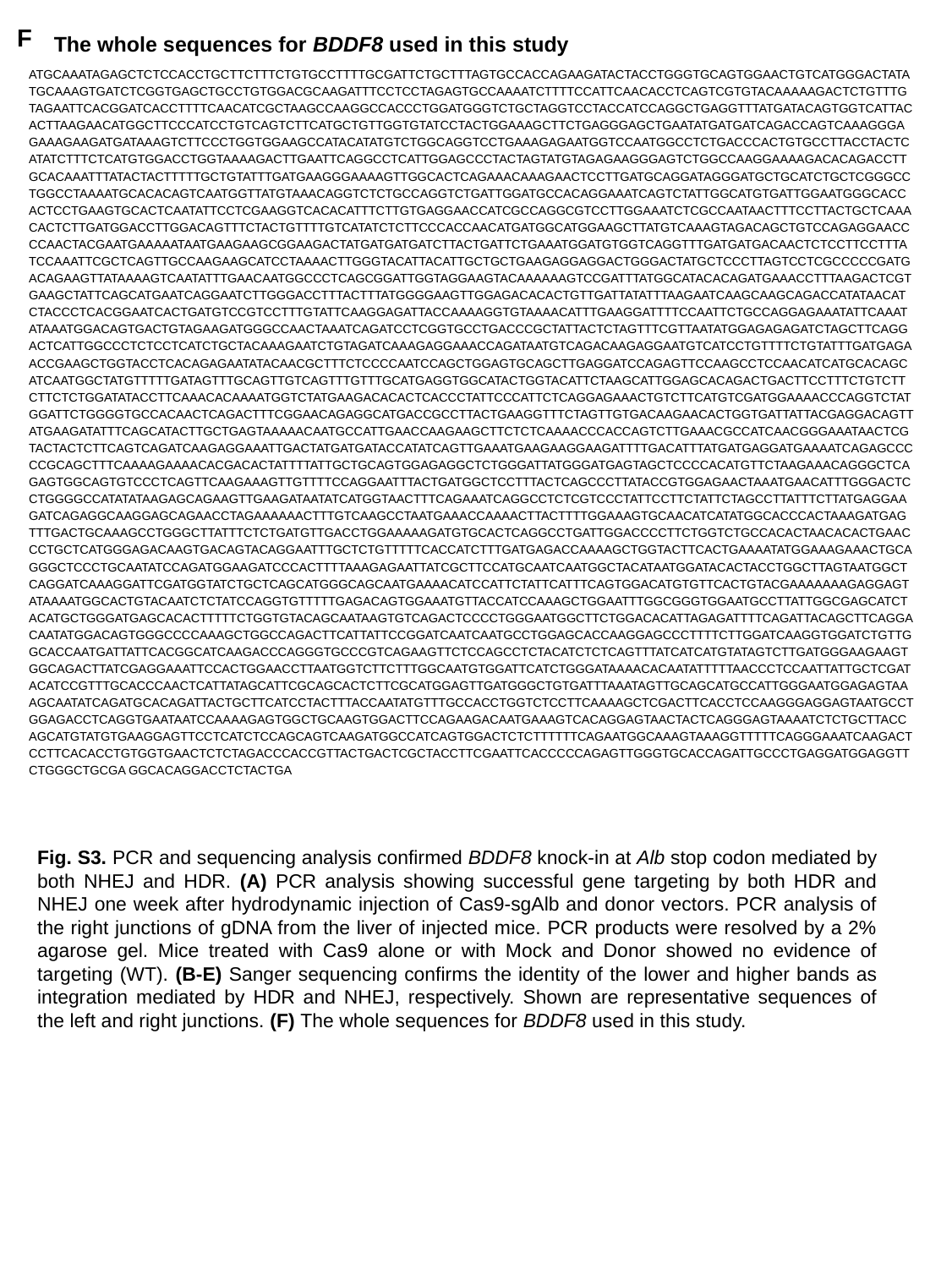

F
The whole sequences for BDDF8 used in this study
Atgcaaatagagctctccacctgcttctttctgtgccttttgcgattctgctttagtgccaccagaagatactacctgggtgcagtggaactgtcatgggactatatgcaaagtgatctcggtgagctgcctgtggacgcaagatttcctcctagagtgccaaaatcttttccattcaacacctcagtcgtgtacaaaaagactctgtttgtagaattcacggatcaccttttcaacatcgctaagccaaggccaccctggatgggtctgctaggtcctaccatccaggctgaggtttatgatacagtggtcattacacttaagaacatggcttcccatcctgtcagtcttcatgctgttggtgtatcctactggaaagcttctgagggagctgaatatgatgatcagaccagtcaaagggagaaagaagatgataaagtcttccctggtggaagccatacatatgtctggcaggtcctgaaagagaatggtccaatggcctctgacccactgtgccttacctactcatatctttctcatgtggacctggtaaaagacttgaattcaggcctcattggagccctactagtatgtagagaagggagtctggccaaggaaaagacacagaccttgcacaaatttatactactttttgctgtatttgatgaagggaaaagttggcactcagaaacaaagaactccttgatgcaggatagggatgctgcatctgctcgggcctggcctaaaatgcacacagtcaatggttatgtaaacaggtctctgccaggtctgattggatgccacaggaaatcagtctattggcatgtgattggaatgggcaccactcctgaagtgcactcaatattcctcgaaggtcacacatttcttgtgaggaaccatcgccaggcgtccttggaaatctcgccaataactttccttactgctcaaacactcttgatggaccttggacagtttctactgttttgtcatatctcttcccaccaacatgatggcatggaagcttatgtcaaagtagacagctgtccagaggaaccccaactacgaatgaaaaataatgaagaagcggaagactatgatgatgatcttactgattctgaaatggatgtggtcaggtttgatgatgacaactctccttcctttatccaaattcgctcagttgccaagaagcatcctaaaacttgggtacattacattgctgctgaagaggaggactgggactatgctcccttagtcctcgcccccgatgacagaagttataaaagtcaatatttgaacaatggccctcagcggattggtaggaagtacaaaaaagtccgatttatggcatacacagatgaaacctttaagactcgtgaagctattcagcatgaatcaggaatcttgggacctttactttatggggaagttggagacacactgttgattatatttaagaatcaagcaagcagaccatataacatctaccctcacggaatcactgatgtccgtcctttgtattcaaggagattaccaaaaggtgtaaaacatttgaaggattttccaattctgccaggagaaatattcaaatataaatggacagtgactgtagaagatgggccaactaaatcagatcctcggtgcctgacccgctattactctagtttcgttaatatggagagagatctagcttcaggactcattggccctctcctcatctgctacaaagaatctgtagatcaaagaggaaaccagataatgtcagacaagaggaatgtcatcctgttttctgtatttgatgagaaccgaagctggtacctcacagagaatatacaacgctttctccccaatccagctggagtgcagcttgaggatccagagttccaagcctccaacatcatgcacagcatcaatggctatgtttttgatagtttgcagttgtcagtttgtttgcatgaggtggcatactggtacattctaagcattggagcacagactgacttcctttctgtcttcttctctggatataccttcaaacacaaaatggtctatgaagacacactcaccctattcccattctcaggagaaactgtcttcatgtcgatggaaaacccaggtctatggattctggggtgccacaactcagactttcggaacagaggcatgaccgccttactgaaggtttctagttgtgacaagaacactggtgattattacgaggacagttatgaagatatttcagcatacttgctgagtaaaaacaatgccattgaaccaagaagcttctctcaaaacccaccagtcttgaaacgccatcaacgggaaataactcgtactactcttcagtcagatcaagaggaaattgactatgatgataccatatcagttgaaatgaagaaggaagattttgacatttatgatgaggatgaaaatcagagcccccgcagctttcaaaagaaaacacgacactattttattgctgcagtggagaggctctgggattatgggatgagtagctccccacatgttctaagaaacagggctcagagtggcagtgtccctcagttcaagaaagttgttttccaggaatttactgatggctcctttactcagcccttataccgtggagaactaaatgaacatttgggactcctggggccatatataagagcagaagttgaagataatatcatggtaactttcagaaatcaggcctctcgtccctattccttctattctagccttatttcttatgaggaagatcagaggcaaggagcagaacctagaaaaaactttgtcaagcctaatgaaaccaaaacttacttttggaaagtgcaacatcatatggcacccactaaagatgagtttgactgcaaagcctgggcttatttctctgatgttgacctggaaaaagatgtgcactcaggcctgattggaccccttctggtctgccacactaacacactgaaccctgctcatgggagacaagtgacagtacaggaatttgctctgtttttcaccatctttgatgagaccaaaagctggtacttcactgaaaatatggaaagaaactgcagggctccctgcaatatccagatggaagatcccacttttaaagagaattatcgcttccatgcaatcaatggctacataatggatacactacctggcttagtaatggctcaggatcaaaggattcgatggtatctgctcagcatgggcagcaatgaaaacatccattctattcatttcagtggacatgtgttcactgtacgaaaaaaagaggagtataaaatggcactgtacaatctctatccaggtgtttttgagacagtggaaatgttaccatccaaagctggaatttggcgggtggaatgccttattggcgagcatctacatgctgggatgagcacactttttctggtgtacagcaataagtgtcagactcccctgggaatggcttctggacacattagagattttcagattacagcttcaggacaatatggacagtgggccccaaagctggccagacttcattattccggatcaatcaatgcctggagcaccaaggagcccttttcttggatcaaggtggatctgttggcaccaatgattattcacggcatcaagacccagggtgcccgtcagaagttctccagcctctacatctctcagtttatcatcatgtatagtcttgatgggaagaagtggcagacttatcgaggaaattccactggaaccttaatggtcttctttggcaatgtggattcatctgggataaaacacaatatttttaaccctccaattattgctcgatacatccgtttgcacccaactcattatagcattcgcagcactcttcgcatggagttgatgggctgtgatttaaatagttgcagcatgccattgggaatggagagtaaagcaatatcagatgcacagattactgcttcatcctactttaccaatatgtttgccacctggtctccttcaaaagctcgacttcacctccaagggaggagtaatgcctggagacctcaggtgaataatccaaaagagtggctgcaagtggacttccagaagacaatgaaagtcacaggagtaactactcagggagtaaaatctctgcttaccagcatgtatgtgaaggagttcctcatctccagcagtcaagatggccatcagtggactctcttttttcagaatggcaaagtaaaggtttttcagggaaatcaagactccttcacacctgtggtgaactctctagacccaccgttactgactcgctaccttcgaattcacccccagagttgggtgcaccagattgccctgaggatggaggttctgggctgcga ggcacaggacctctactga
Fig. S3. PCR and sequencing analysis confirmed BDDF8 knock-in at Alb stop codon mediated by both NHEJ and HDR. (A) PCR analysis showing successful gene targeting by both HDR and NHEJ one week after hydrodynamic injection of Cas9-sgAlb and donor vectors. PCR analysis of the right junctions of gDNA from the liver of injected mice. PCR products were resolved by a 2% agarose gel. Mice treated with Cas9 alone or with Mock and Donor showed no evidence of targeting (WT). (B-E) Sanger sequencing confirms the identity of the lower and higher bands as integration mediated by HDR and NHEJ, respectively. Shown are representative sequences of the left and right junctions. (F) The whole sequences for BDDF8 used in this study.

## Slide 6
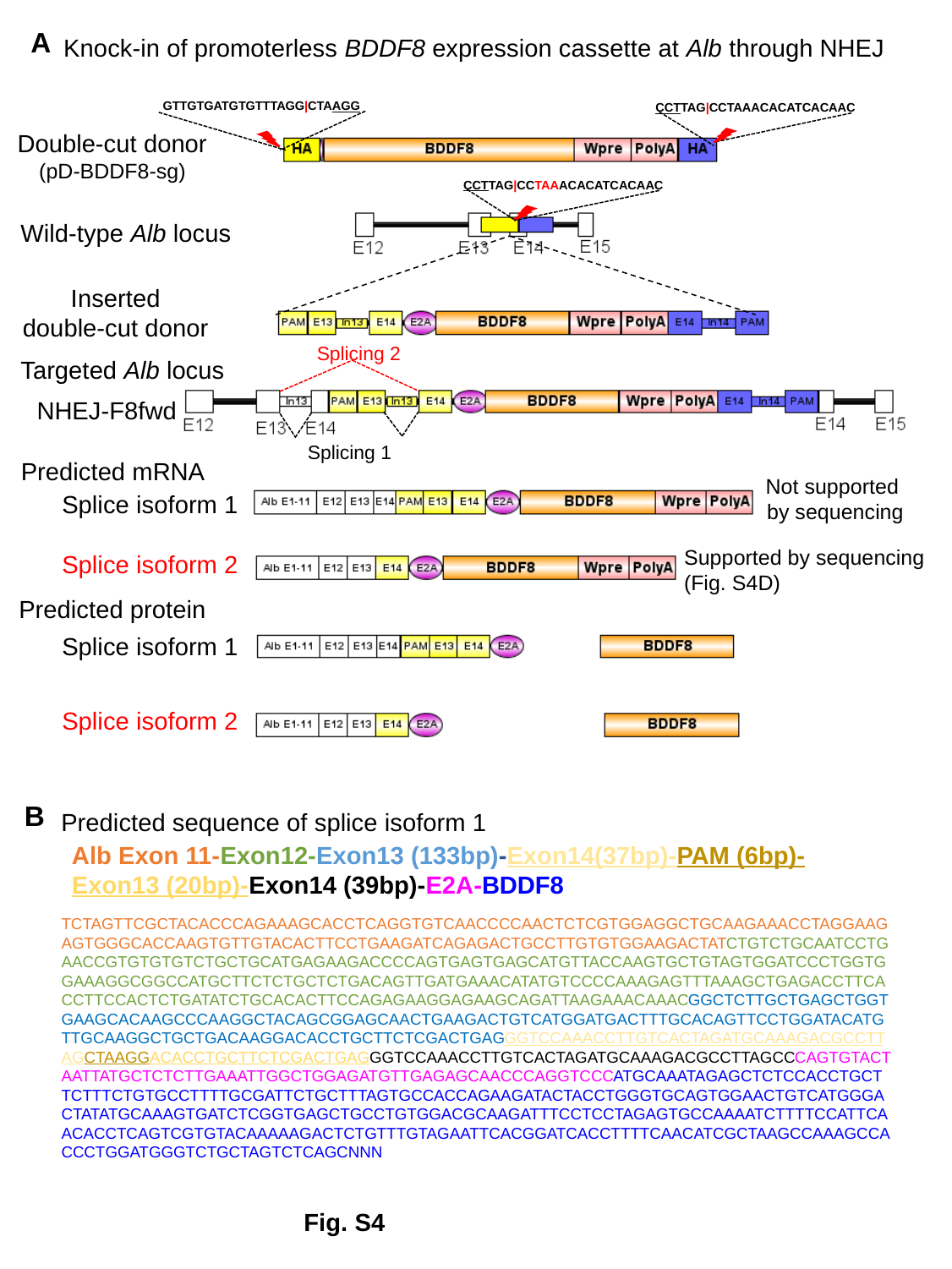

A
Knock-in of promoterless BDDF8 expression cassette at Alb through NHEJ
GTTGTGATGTGTTTAGG|CTAAGG
CCTTAG|CCTAAACACATCACAAC
Double-cut donor
(pD-BDDF8-sg)
CCTTAG|CCTAAACACATCACAAC
Wild-type Alb locus
Inserted
double-cut donor
Splicing 2
Targeted Alb locus
NHEJ-F8fwd
Splicing 1
Predicted mRNA
Not supported
by sequencing
Splice isoform 1
Supported by sequencing
(Fig. S4D)
Splice isoform 2
Predicted protein
Splice isoform 1
Splice isoform 2
B
Predicted sequence of splice isoform 1
Alb Exon 11-Exon12-Exon13 (133bp)-Exon14(37bp)-PAM (6bp)-Exon13 (20bp)-Exon14 (39bp)-E2A-BDDF8
TCTAGTTCGCTACACCCAGAAAGCACCTCAGGTGTCAACCCCAACTCTCGTGGAGGCTGCAAGAAACCTAGGAAGAGTGGGCACCAAGTGTTGTACACTTCCTGAAGATCAGAGACTGCCTTGTGTGGAAGACTATCTGTCTGCAATCCTGAACCGTGTGTGTCTGCTGCATGAGAAGACCCCAGTGAGTGAGCATGTTACCAAGTGCTGTAGTGGATCCCTGGTGGAAAGGCGGCCATGCTTCTCTGCTCTGACAGTTGATGAAACATATGTCCCCAAAGAGTTTAAAGCTGAGACCTTCACCTTCCACTCTGATATCTGCACACTTCCAGAGAAGGAGAAGCAGATTAAGAAACAAACGGCTCTTGCTGAGCTGGTGAAGCACAAGCCCAAGGCTACAGCGGAGCAACTGAAGACTGTCATGGATGACTTTGCACAGTTCCTGGATACATGTTGCAAGGCTGCTGACAAGGACACCTGCTTCTCGACTGAGGGTCCAAACCTTGTCACTAGATGCAAAGACGCCTTAGctaaggacacctgcttctcgactgagggtccaaaccttgtcactagatgcaaagacgccttagccCAGTGTACTAATTATGCTCTCTTGAAATTGGCTGGAGATGTTGAGAGCAACCCAGGTCCCATGCAAATAGAGCTCTCCACCTGCTTCTTTCTGTGCCTTTTGCGATTCTGCTTTAGTGCCACCAGAAGATACTACCTGGGTGCAGTGGAACTGTCATGGGACTATATGCAAAGTGATCTCGGTGAGCTGCCTGTGGACGCAAGATTTCCTCCTAGAGTGCCAAAATCTTTTCCATTCAACACCTCAGTCGTGTACAAAAAGACTCTGTTTGTAGAATTCACGGATCACCTTTTCAACATCGCTAAGCCAAAGCCACCCTGGATGGGTCTGCTAGTCTCAGCNNN
Fig. S4

## Slide 7
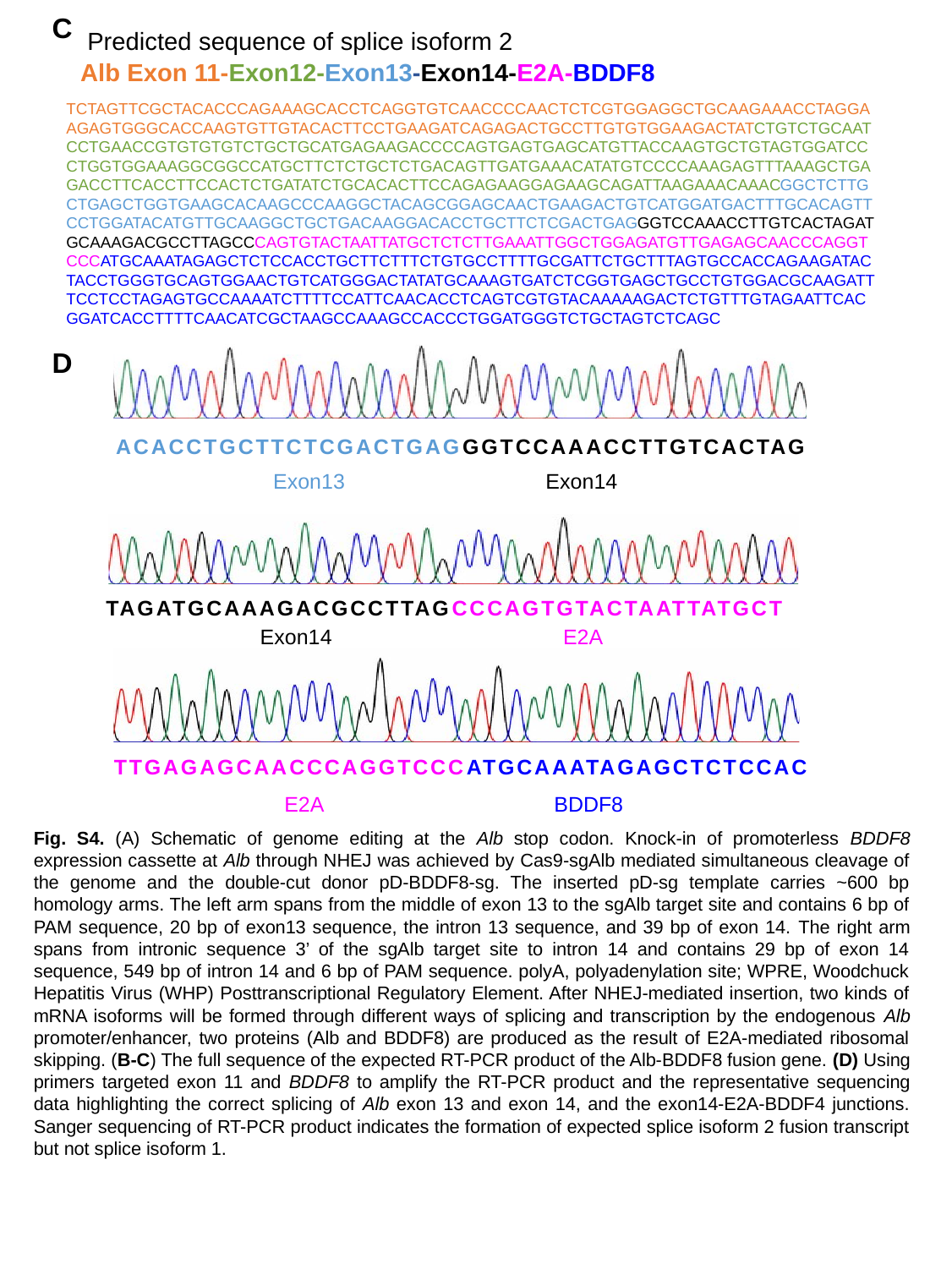

C
Predicted sequence of splice isoform 2
Alb Exon 11-Exon12-Exon13-Exon14-E2A-BDDF8
TCTAGTTCGCTACACCCAGAAAGCACCTCAGGTGTCAACCCCAACTCTCGTGGAGGCTGCAAGAAACCTAGGAAGAGTGGGCACCAAGTGTTGTACACTTCCTGAAGATCAGAGACTGCCTTGTGTGGAAGACTATCTGTCTGCAATCCTGAACCGTGTGTGTCTGCTGCATGAGAAGACCCCAGTGAGTGAGCATGTTACCAAGTGCTGTAGTGGATCCCTGGTGGAAAGGCGGCCATGCTTCTCTGCTCTGACAGTTGATGAAACATATGTCCCCAAAGAGTTTAAAGCTGAGACCTTCACCTTCCACTCTGATATCTGCACACTTCCAGAGAAGGAGAAGCAGATTAAGAAACAAACGGCTCTTGCTGAGCTGGTGAAGCACAAGCCCAAGGCTACAGCGGAGCAACTGAAGACTGTCATGGATGACTTTGCACAGTTCCTGGATACATGTTGCAAGGCTGCTGACAAGGACACCTGCTTCTCGACTGAGGGTCCAAACCTTGTCACTAGATGCAAAGACGCCTTAGCCCAGTGTACTAATTATGCTCTCTTGAAATTGGCTGGAGATGTTGAGAGCAACCCAGGTCCCATGCAAATAGAGCTCTCCACCTGCTTCTTTCTGTGCCTTTTGCGATTCTGCTTTAGTGCCACCAGAAGATACTACCTGGGTGCAGTGGAACTGTCATGGGACTATATGCAAAGTGATCTCGGTGAGCTGCCTGTGGACGCAAGATTTCCTCCTAGAGTGCCAAAATCTTTTCCATTCAACACCTCAGTCGTGTACAAAAAGACTCTGTTTGTAGAATTCACGGATCACCTTTTCAACATCGCTAAGCCAAAGCCACCCTGGATGGGTCTGCTAGTCTCAGC
D
ACACCTGCTTCTCGACTGAGGGTCCAAACCTTGTCACTAG
Exon13 Exon14
TAGATGCAAAGACGCCTTAGCCCAGTGTACTAATTATGCT
Exon14 E2A
TTGAGAGCAACCCAGGTCCCATGCAAATAGAGCTCTCCAC
E2A BDDF8
Fig. S4. (A) Schematic of genome editing at the Alb stop codon. Knock-in of promoterless BDDF8 expression cassette at Alb through NHEJ was achieved by Cas9-sgAlb mediated simultaneous cleavage of the genome and the double-cut donor pD-BDDF8-sg. The inserted pD-sg template carries ~600 bp homology arms. The left arm spans from the middle of exon 13 to the sgAlb target site and contains 6 bp of PAM sequence, 20 bp of exon13 sequence, the intron 13 sequence, and 39 bp of exon 14. The right arm spans from intronic sequence 3’ of the sgAlb target site to intron 14 and contains 29 bp of exon 14 sequence, 549 bp of intron 14 and 6 bp of PAM sequence. polyA, polyadenylation site; WPRE, Woodchuck Hepatitis Virus (WHP) Posttranscriptional Regulatory Element. After NHEJ-mediated insertion, two kinds of mRNA isoforms will be formed through different ways of splicing and transcription by the endogenous Alb promoter/enhancer, two proteins (Alb and BDDF8) are produced as the result of E2A-mediated ribosomal skipping. (B-C) The full sequence of the expected RT-PCR product of the Alb-BDDF8 fusion gene. (D) Using primers targeted exon 11 and BDDF8 to amplify the RT-PCR product and the representative sequencing data highlighting the correct splicing of Alb exon 13 and exon 14, and the exon14-E2A-BDDF4 junctions. Sanger sequencing of RT-PCR product indicates the formation of expected splice isoform 2 fusion transcript but not splice isoform 1.

## Slide 8
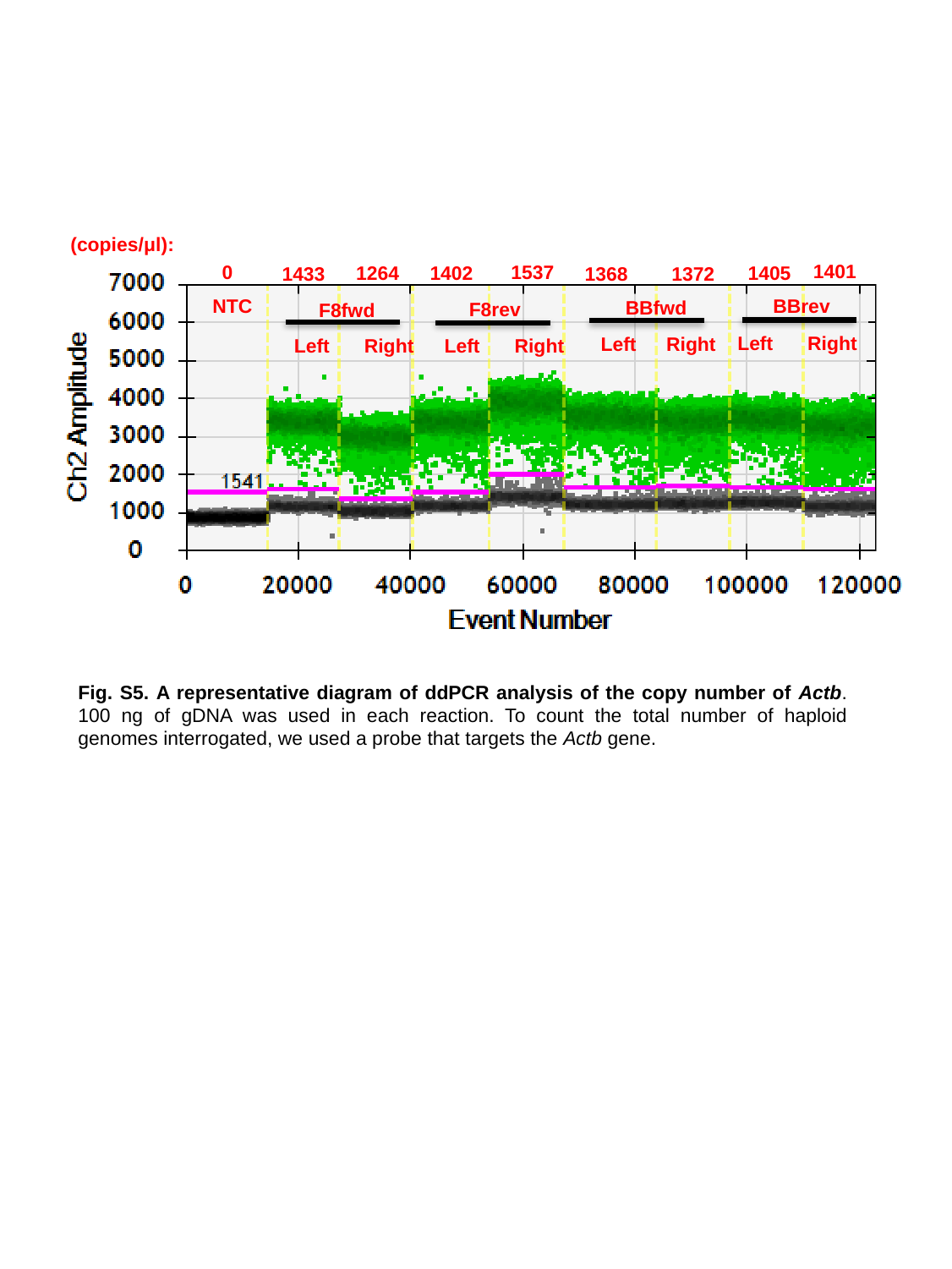

(copies/μl):
1401
0
1537
1402
1405
1264
1368
1372
1433
BBrev
NTC
BBfwd
F8rev
F8fwd
Left
Right
Left
Right
Left
Right
Left
Right
Fig. S5. A representative diagram of ddPCR analysis of the copy number of Actb. 100 ng of gDNA was used in each reaction. To count the total number of haploid genomes interrogated, we used a probe that targets the Actb gene.

## Slide 9
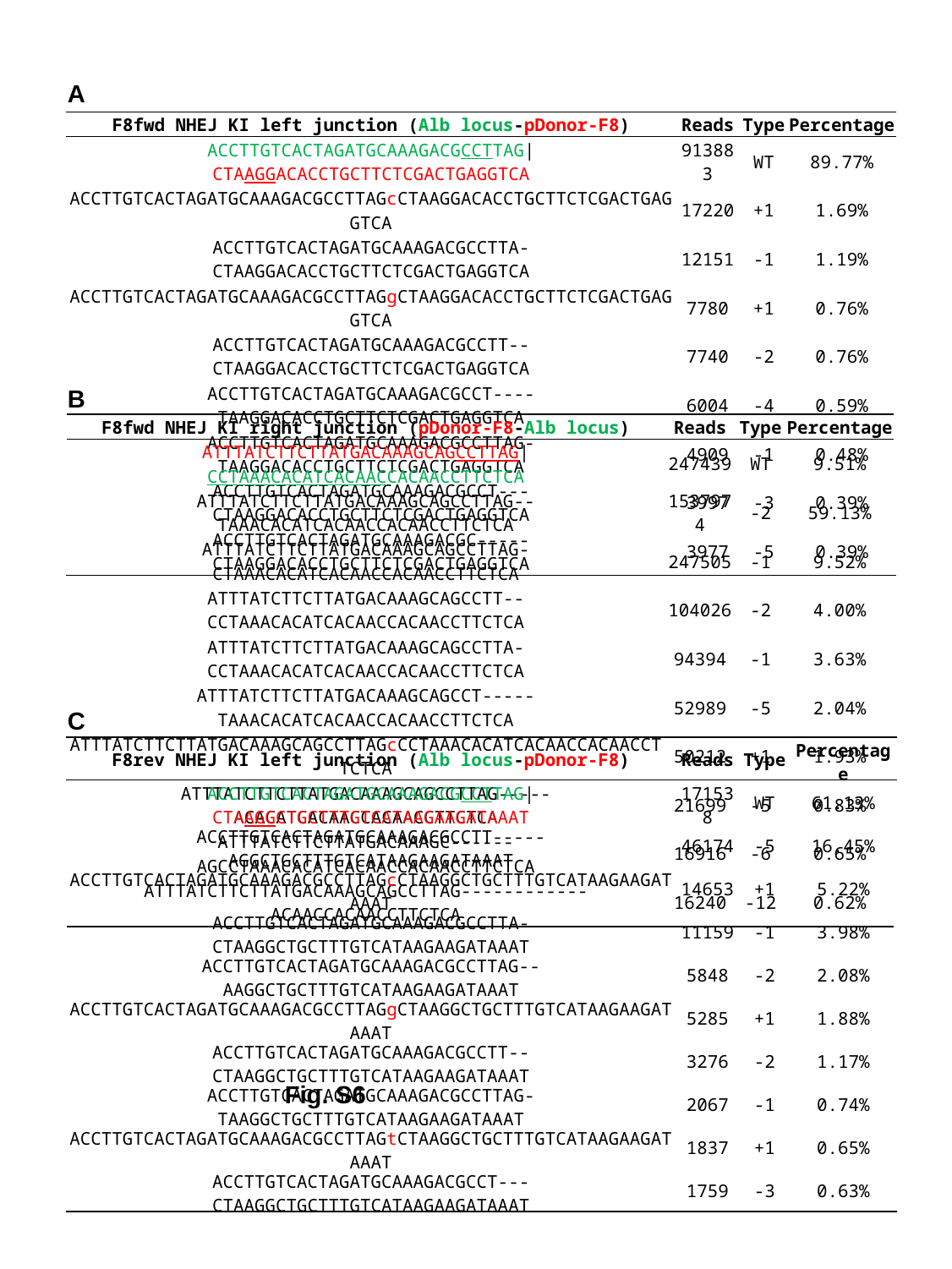

A
| F8fwd NHEJ KI left junction (Alb locus-pDonor-F8) | Reads | Type | Percentage |
| --- | --- | --- | --- |
| ACCTTGTCACTAGATGCAAAGACGCCTTAG|CTAAGGACACCTGCTTCTCGACTGAGGTCA | 913883 | WT | 89.77% |
| ACCTTGTCACTAGATGCAAAGACGCCTTAGcCTAAGGACACCTGCTTCTCGACTGAGGTCA | 17220 | +1 | 1.69% |
| ACCTTGTCACTAGATGCAAAGACGCCTTA-CTAAGGACACCTGCTTCTCGACTGAGGTCA | 12151 | -1 | 1.19% |
| ACCTTGTCACTAGATGCAAAGACGCCTTAGgCTAAGGACACCTGCTTCTCGACTGAGGTCA | 7780 | +1 | 0.76% |
| ACCTTGTCACTAGATGCAAAGACGCCTT--CTAAGGACACCTGCTTCTCGACTGAGGTCA | 7740 | -2 | 0.76% |
| ACCTTGTCACTAGATGCAAAGACGCCT----TAAGGACACCTGCTTCTCGACTGAGGTCA | 6004 | -4 | 0.59% |
| ACCTTGTCACTAGATGCAAAGACGCCTTAG-TAAGGACACCTGCTTCTCGACTGAGGTCA | 4909 | -1 | 0.48% |
| ACCTTGTCACTAGATGCAAAGACGCCT---CTAAGGACACCTGCTTCTCGACTGAGGTCA | 3997 | -3 | 0.39% |
| ACCTTGTCACTAGATGCAAAGACGC-----CTAAGGACACCTGCTTCTCGACTGAGGTCA | 3977 | -5 | 0.39% |
B
| F8fwd NHEJ KI right junction (pDonor-F8-Alb locus) | Reads | Type | Percentage |
| --- | --- | --- | --- |
| ATTTATCTTCTTATGACAAAGCAGCCTTAG|CCTAAACACATCACAACCACAACCTTCTCA | 247439 | WT | 9.51% |
| ATTTATCTTCTTATGACAAAGCAGCCTTAG--TAAACACATCACAACCACAACCTTCTCA | 1537974 | -2 | 59.13% |
| ATTTATCTTCTTATGACAAAGCAGCCTTAG-CTAAACACATCACAACCACAACCTTCTCA | 247505 | -1 | 9.52% |
| ATTTATCTTCTTATGACAAAGCAGCCTT--CCTAAACACATCACAACCACAACCTTCTCA | 104026 | -2 | 4.00% |
| ATTTATCTTCTTATGACAAAGCAGCCTTA-CCTAAACACATCACAACCACAACCTTCTCA | 94394 | -1 | 3.63% |
| ATTTATCTTCTTATGACAAAGCAGCCT-----TAAACACATCACAACCACAACCTTCTCA | 52989 | -5 | 2.04% |
| ATTTATCTTCTTATGACAAAGCAGCCTTAGcCCTAAACACATCACAACCACAACCTTCTCA | 50212 | +1 | 1.93% |
| ATTTATCTTCTTATGACAAAGCAGCCTTAG-----ACACATCACAACCACAACCTTCTCA | 21699 | -5 | 0.83% |
| ATTTATCTTCTTATGACAAAGC------AGCCTAAACACATCACAACCACAACCTTCTCA | 16916 | -6 | 0.65% |
| ATTTATCTTCTTATGACAAAGCAGCCTTAG------------ACAACCACAACCTTCTCA | 16240 | -12 | 0.62% |
C
| F8rev NHEJ KI left junction (Alb locus-pDonor-F8) | Reads | Type | Percentage |
| --- | --- | --- | --- |
| ACCTTGTCACTAGATGCAAAGACGCCTTAG|CTAAGGCTGCTTTGTCATAAGAAGATAAAT | 171538 | WT | 61.13% |
| ACCTTGTCACTAGATGCAAAGACGCCTT-----AGGCTGCTTTGTCATAAGAAGATAAAT | 46174 | -5 | 16.45% |
| ACCTTGTCACTAGATGCAAAGACGCCTTAGcCTAAGGCTGCTTTGTCATAAGAAGATAAAT | 14653 | +1 | 5.22% |
| ACCTTGTCACTAGATGCAAAGACGCCTTA-CTAAGGCTGCTTTGTCATAAGAAGATAAAT | 11159 | -1 | 3.98% |
| ACCTTGTCACTAGATGCAAAGACGCCTTAG--AAGGCTGCTTTGTCATAAGAAGATAAAT | 5848 | -2 | 2.08% |
| ACCTTGTCACTAGATGCAAAGACGCCTTAGgCTAAGGCTGCTTTGTCATAAGAAGATAAAT | 5285 | +1 | 1.88% |
| ACCTTGTCACTAGATGCAAAGACGCCTT--CTAAGGCTGCTTTGTCATAAGAAGATAAAT | 3276 | -2 | 1.17% |
| ACCTTGTCACTAGATGCAAAGACGCCTTAG-TAAGGCTGCTTTGTCATAAGAAGATAAAT | 2067 | -1 | 0.74% |
| ACCTTGTCACTAGATGCAAAGACGCCTTAGtCTAAGGCTGCTTTGTCATAAGAAGATAAAT | 1837 | +1 | 0.65% |
| ACCTTGTCACTAGATGCAAAGACGCCT---CTAAGGCTGCTTTGTCATAAGAAGATAAAT | 1759 | -3 | 0.63% |
Fig. S6

## Slide 10
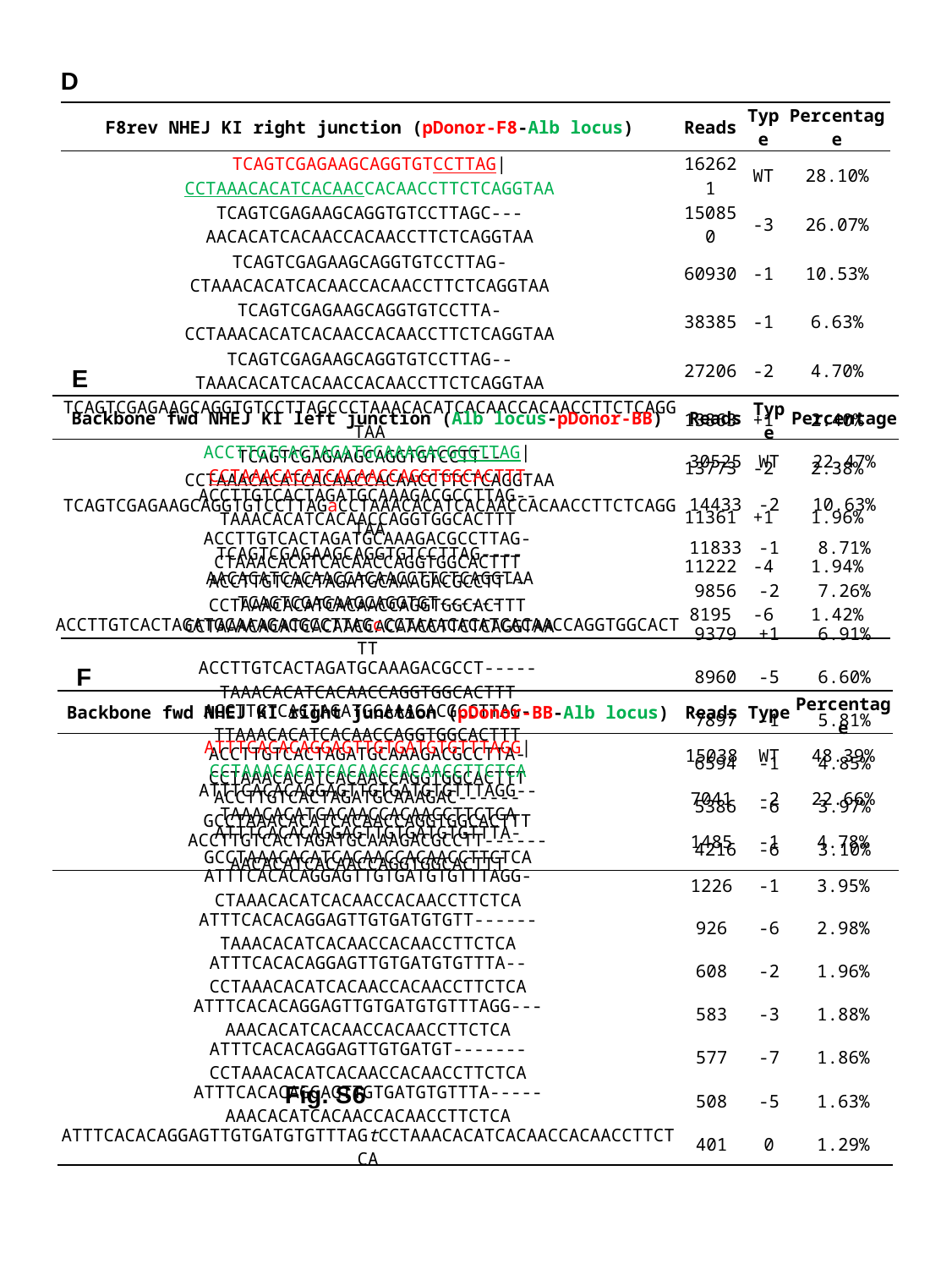

D
| F8rev NHEJ KI right junction (pDonor-F8-Alb locus) | Reads | Type | Percentage |
| --- | --- | --- | --- |
| TCAGTCGAGAAGCAGGTGTCCTTAG|CCTAAACACATCACAACCACAACCTTCTCAGGTAA | 162621 | WT | 28.10% |
| TCAGTCGAGAAGCAGGTGTCCTTAGC---AACACATCACAACCACAACCTTCTCAGGTAA | 150850 | -3 | 26.07% |
| TCAGTCGAGAAGCAGGTGTCCTTAG-CTAAACACATCACAACCACAACCTTCTCAGGTAA | 60930 | -1 | 10.53% |
| TCAGTCGAGAAGCAGGTGTCCTTA-CCTAAACACATCACAACCACAACCTTCTCAGGTAA | 38385 | -1 | 6.63% |
| TCAGTCGAGAAGCAGGTGTCCTTAG--TAAACACATCACAACCACAACCTTCTCAGGTAA | 27206 | -2 | 4.70% |
| TCAGTCGAGAAGCAGGTGTCCTTAGCCCTAAACACATCACAACCACAACCTTCTCAGGTAA | 13863 | +1 | 2.40% |
| TCAGTCGAGAAGCAGGTGTCCTT--CCTAAACACATCACAACCACAACCTTCTCAGGTAA | 13773 | -2 | 2.38% |
| TCAGTCGAGAAGCAGGTGTCCTTAGaCCTAAACACATCACAACCACAACCTTCTCAGGTAA | 11361 | +1 | 1.96% |
| TCAGTCGAGAAGCAGGTGTCCTTAG----AACACATCACAACCACAACCTTCTCAGGTAA | 11222 | -4 | 1.94% |
| TCAGTCGAGAAGCAGGTGT------CCTAAACACATCACAACCACAACCTTCTCAGGTAA | 8195 | -6 | 1.42% |
E
| Backbone fwd NHEJ KI left junction (Alb locus-pDonor-BB) | Reads | Type | Percentage |
| --- | --- | --- | --- |
| ACCTTGTCACTAGATGCAAAGACGCCTTAG|CCTAAACACATCACAACCAGGTGGCACTTT | 30525 | WT | 22.47% |
| ACCTTGTCACTAGATGCAAAGACGCCTTAG--TAAACACATCACAACCAGGTGGCACTTT | 14433 | -2 | 10.63% |
| ACCTTGTCACTAGATGCAAAGACGCCTTAG-CTAAACACATCACAACCAGGTGGCACTTT | 11833 | -1 | 8.71% |
| ACCTTGTCACTAGATGCAAAGACGCCTT--CCTAAACACATCACAACCAGGTGGCACTTT | 9856 | -2 | 7.26% |
| ACCTTGTCACTAGATGCAAAGACGCCTTAGcCCTAAACACATCACAACCAGGTGGCACTTT | 9379 | +1 | 6.91% |
| ACCTTGTCACTAGATGCAAAGACGCCT-----TAAACACATCACAACCAGGTGGCACTTT | 8960 | -5 | 6.60% |
| ACCTTGTCACTAGATGCAAAGACGCCTTAG-TTAAACACATCACAACCAGGTGGCACTTT | 7897 | -1 | 5.81% |
| ACCTTGTCACTAGATGCAAAGACGCCTTA-CCTAAACACATCACAACCAGGTGGCACTTT | 6594 | -1 | 4.85% |
| ACCTTGTCACTAGATGCAAAGAC------GCCTAAACACATCACAACCAGGTGGCACTTT | 5386 | -6 | 3.97% |
| ACCTTGTCACTAGATGCAAAGACGCCTT------AACACATCACAACCAGGTGGCACTTT | 4216 | -6 | 3.10% |
F
| Backbone fwd NHEJ KI right junction (pDonor-BB-Alb locus) | Reads | Type | Percentage |
| --- | --- | --- | --- |
| ATTTCACACAGGAGTTGTGATGTGTTTAGG|CCTAAACACATCACAACCACAACCTTCTCA | 15038 | WT | 48.39% |
| ATTTCACACAGGAGTTGTGATGTGTTTAGG--TAAACACATCACAACCACAACCTTCTCA | 7041 | -2 | 22.66% |
| ATTTCACACAGGAGTTGTGATGTGTTTA-GCCTAAACACATCACAACCACAACCTTCTCA | 1485 | -1 | 4.78% |
| ATTTCACACAGGAGTTGTGATGTGTTTAGG-CTAAACACATCACAACCACAACCTTCTCA | 1226 | -1 | 3.95% |
| ATTTCACACAGGAGTTGTGATGTGTT------TAAACACATCACAACCACAACCTTCTCA | 926 | -6 | 2.98% |
| ATTTCACACAGGAGTTGTGATGTGTTTA--CCTAAACACATCACAACCACAACCTTCTCA | 608 | -2 | 1.96% |
| ATTTCACACAGGAGTTGTGATGTGTTTAGG---AAACACATCACAACCACAACCTTCTCA | 583 | -3 | 1.88% |
| ATTTCACACAGGAGTTGTGATGT-------CCTAAACACATCACAACCACAACCTTCTCA | 577 | -7 | 1.86% |
| ATTTCACACAGGAGTTGTGATGTGTTTA-----AAACACATCACAACCACAACCTTCTCA | 508 | -5 | 1.63% |
| ATTTCACACAGGAGTTGTGATGTGTTTAGtCCTAAACACATCACAACCACAACCTTCTCA | 401 | 0 | 1.29% |
Fig. S6

## Slide 11
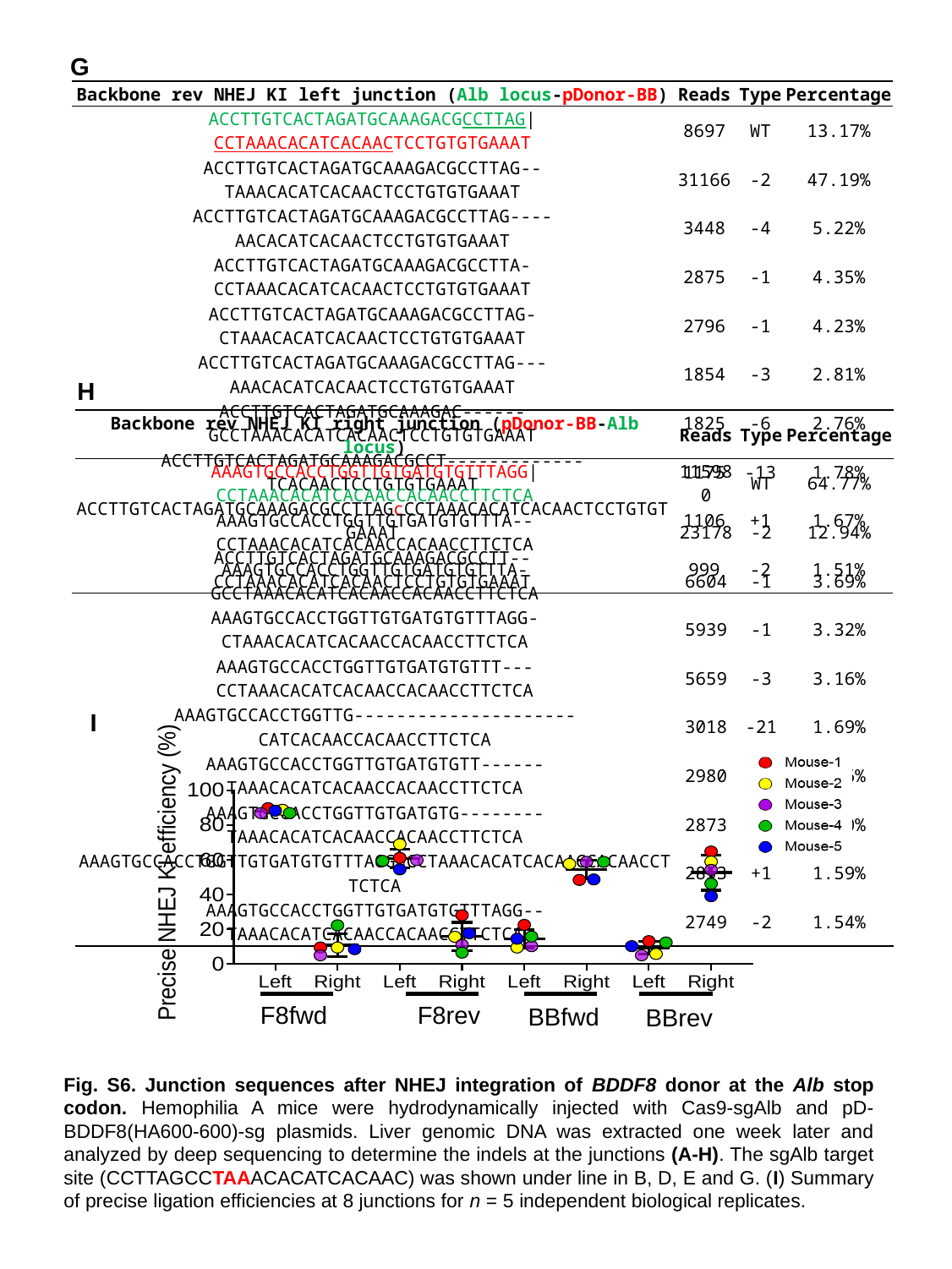

G
| Backbone rev NHEJ KI left junction (Alb locus-pDonor-BB) | Reads | Type | Percentage |
| --- | --- | --- | --- |
| ACCTTGTCACTAGATGCAAAGACGCCTTAG|CCTAAACACATCACAACTCCTGTGTGAAAT | 8697 | WT | 13.17% |
| ACCTTGTCACTAGATGCAAAGACGCCTTAG--TAAACACATCACAACTCCTGTGTGAAAT | 31166 | -2 | 47.19% |
| ACCTTGTCACTAGATGCAAAGACGCCTTAG----AACACATCACAACTCCTGTGTGAAAT | 3448 | -4 | 5.22% |
| ACCTTGTCACTAGATGCAAAGACGCCTTA-CCTAAACACATCACAACTCCTGTGTGAAAT | 2875 | -1 | 4.35% |
| ACCTTGTCACTAGATGCAAAGACGCCTTAG-CTAAACACATCACAACTCCTGTGTGAAAT | 2796 | -1 | 4.23% |
| ACCTTGTCACTAGATGCAAAGACGCCTTAG---AAACACATCACAACTCCTGTGTGAAAT | 1854 | -3 | 2.81% |
| ACCTTGTCACTAGATGCAAAGAC------GCCTAAACACATCACAACTCCTGTGTGAAAT | 1825 | -6 | 2.76% |
| ACCTTGTCACTAGATGCAAAGACGCCT-------------TCACAACTCCTGTGTGAAAT | 1175 | -13 | 1.78% |
| ACCTTGTCACTAGATGCAAAGACGCCTTAGcCCTAAACACATCACAACTCCTGTGTGAAAT | 1106 | +1 | 1.67% |
| ACCTTGTCACTAGATGCAAAGACGCCTT--CCTAAACACATCACAACTCCTGTGTGAAAT | 999 | -2 | 1.51% |
H
| Backbone rev NHEJ KI right junction (pDonor-BB-Alb locus) | Reads | Type | Percentage |
| --- | --- | --- | --- |
| AAAGTGCCACCTGGTTGTGATGTGTTTAGG|CCTAAACACATCACAACCACAACCTTCTCA | 115980 | WT | 64.77% |
| AAAGTGCCACCTGGTTGTGATGTGTTTA--CCTAAACACATCACAACCACAACCTTCTCA | 23178 | -2 | 12.94% |
| AAAGTGCCACCTGGTTGTGATGTGTTTA-GCCTAAACACATCACAACCACAACCTTCTCA | 6604 | -1 | 3.69% |
| AAAGTGCCACCTGGTTGTGATGTGTTTAGG-CTAAACACATCACAACCACAACCTTCTCA | 5939 | -1 | 3.32% |
| AAAGTGCCACCTGGTTGTGATGTGTTT---CCTAAACACATCACAACCACAACCTTCTCA | 5659 | -3 | 3.16% |
| AAAGTGCCACCTGGTTG---------------------CATCACAACCACAACCTTCTCA | 3018 | -21 | 1.69% |
| AAAGTGCCACCTGGTTGTGATGTGTT------TAAACACATCACAACCACAACCTTCTCA | 2980 | -6 | 1.66% |
| AAAGTGCCACCTGGTTGTGATGTG--------TAAACACATCACAACCACAACCTTCTCA | 2873 | -8 | 1.60% |
| AAAGTGCCACCTGGTTGTGATGTGTTTAGGaCCTAAACACATCACAACCACAACCTTCTCA | 2853 | +1 | 1.59% |
| AAAGTGCCACCTGGTTGTGATGTGTTTAGG--TAAACACATCACAACCACAACCTTCTCA | 2749 | -2 | 1.54% |
I
F8fwd
F8rev
BBfwd
BBrev
Fig. S6. Junction sequences after NHEJ integration of BDDF8 donor at the Alb stop codon. Hemophilia A mice were hydrodynamically injected with Cas9-sgAlb and pD-BDDF8(HA600-600)-sg plasmids. Liver genomic DNA was extracted one week later and analyzed by deep sequencing to determine the indels at the junctions (A-H). The sgAlb target site (CCTTAGCCTAAACACATCACAAC) was shown under line in B, D, E and G. (I) Summary of precise ligation efficiencies at 8 junctions for n = 5 independent biological replicates.

## Slide 12
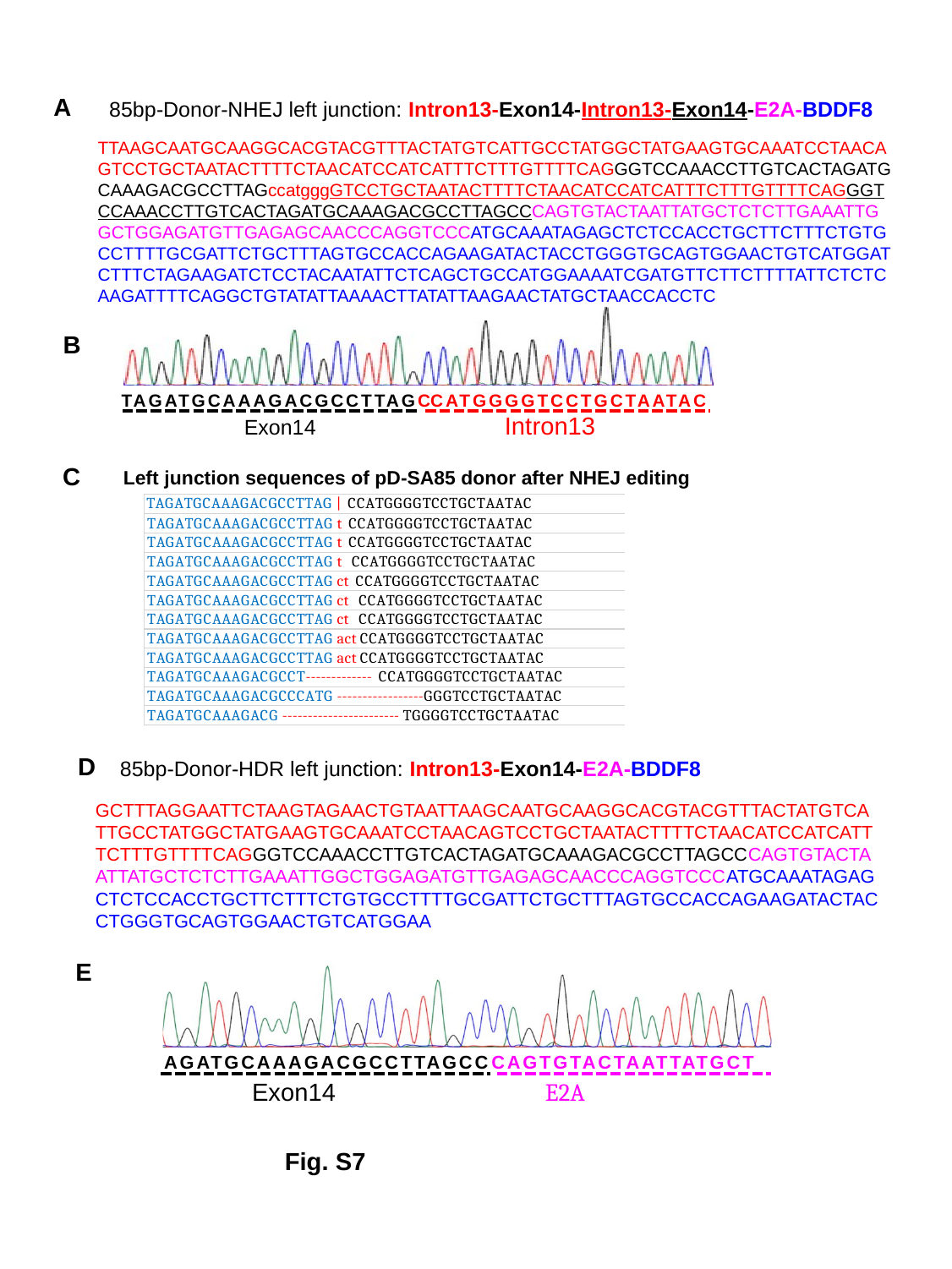

A
85bp-Donor-NHEJ left junction: Intron13-Exon14-Intron13-Exon14-E2A-BDDF8
TTAAGCAATGCAAGGCACGTACGTTTACTATGTCATTGCCTATGGCTATGAAGTGCAAATCCTAACAGTCCTGCTAATACTTTTCTAACATCCATCATTTCTTTGTTTTCAGGGTCCAAACCTTGTCACTAGATGCAAAGACGCCTTAGccatgggGTCCTGCTAATACTTTTCTAACATCCATCATTTCTTTGTTTTCAGGGTCCAAACCTTGTCACTAGATGCAAAGACGCCTTAGCCCAGTGTACTAATTATGCTCTCTTGAAATTGGCTGGAGATGTTGAGAGCAACCCAGGTCCCATGCAAATAGAGCTCTCCACCTGCTTCTTTCTGTGCCTTTTGCGATTCTGCTTTAGTGCCACCAGAAGATACTACCTGGGTGCAGTGGAACTGTCATGGATCTTTCTAGAAGATCTCCTACAATATTCTCAGCTGCCATGGAAAATCGATGTTCTTCTTTTATTCTCTCAAGATTTTCAGGCTGTATATTAAAACTTATATTAAGAACTATGCTAACCACCTC
B
TAGATGCAAAGACGCCTTAGCCATGGGGTCCTGCTAATAC
Exon14 Intron13
C
Left junction sequences of pD-SA85 donor after NHEJ editing
D
85bp-Donor-HDR left junction: Intron13-Exon14-E2A-BDDF8
GCTTTAGGAATTCTAAGTAGAACTGTAATTAAGCAATGCAAGGCACGTACGTTTACTATGTCATTGCCTATGGCTATGAAGTGCAAATCCTAACAGTCCTGCTAATACTTTTCTAACATCCATCATTTCTTTGTTTTCAGGGTCCAAACCTTGTCACTAGATGCAAAGACGCCTTAGCCCAGTGTACTAATTATGCTCTCTTGAAATTGGCTGGAGATGTTGAGAGCAACCCAGGTCCCATGCAAATAGAGCTCTCCACCTGCTTCTTTCTGTGCCTTTTGCGATTCTGCTTTAGTGCCACCAGAAGATACTACCTGGGTGCAGTGGAACTGTCATGGAA
E
AGATGCAAAGACGCCTTAGCCCAGTGTACTAATTATGCT
Exon14 E2A
Fig. S7

## Slide 13
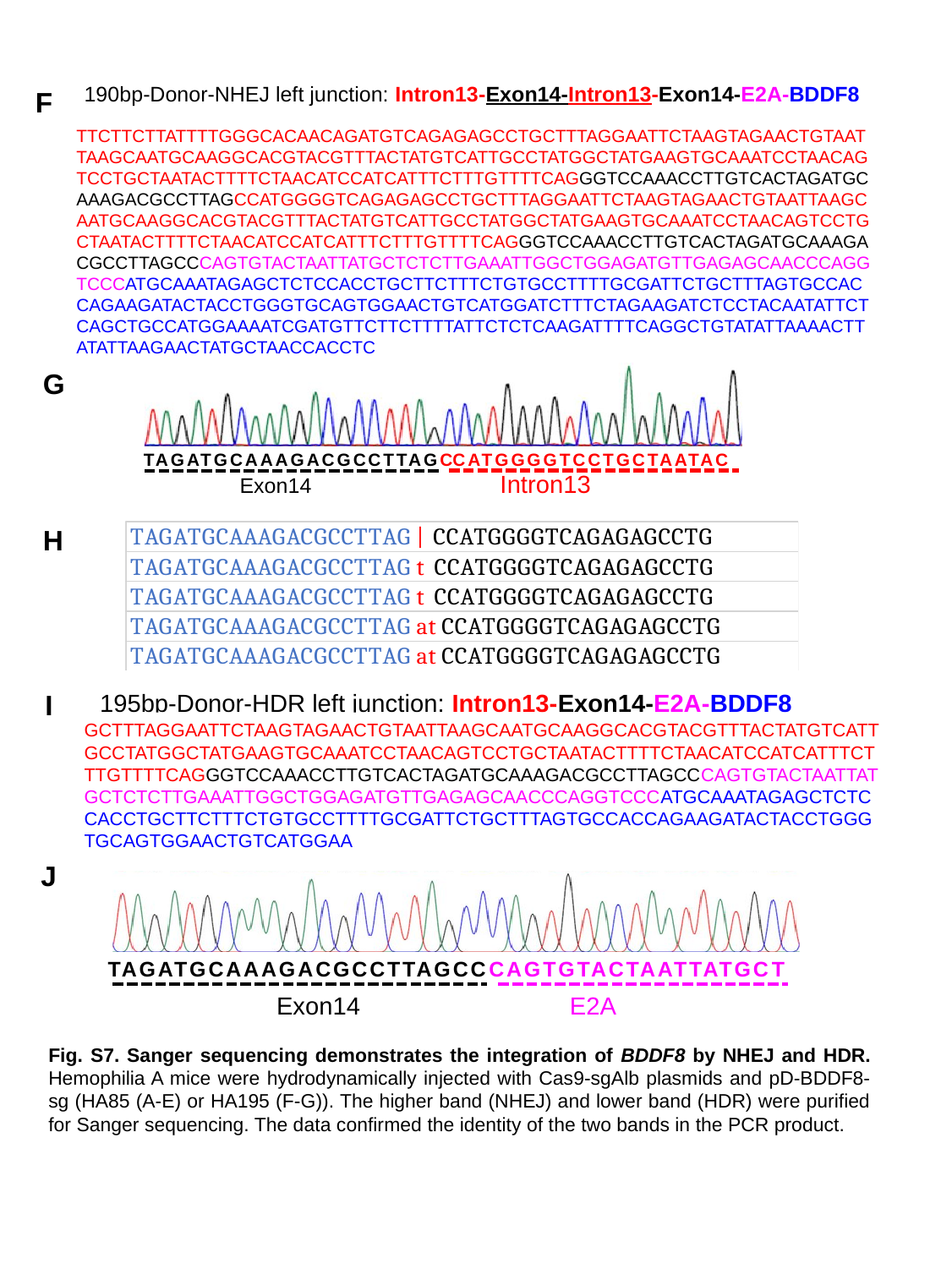

190bp-Donor-NHEJ left junction: Intron13-Exon14-Intron13-Exon14-E2A-BDDF8
F
TTCTTCTTATTTTGGGCACAACAGATGTCAGAGAGCCTGCTTTAGGAATTCTAAGTAGAACTGTAATTAAGCAATGCAAGGCACGTACGTTTACTATGTCATTGCCTATGGCTATGAAGTGCAAATCCTAACAGTCCTGCTAATACTTTTCTAACATCCATCATTTCTTTGTTTTCAGGGTCCAAACCTTGTCACTAGATGCAAAGACGCCTTAGCCATGGGGTCAGAGAGCCTGCTTTAGGAATTCTAAGTAGAACTGTAATTAAGCAATGCAAGGCACGTACGTTTACTATGTCATTGCCTATGGCTATGAAGTGCAAATCCTAACAGTCCTGCTAATACTTTTCTAACATCCATCATTTCTTTGTTTTCAGGGTCCAAACCTTGTCACTAGATGCAAAGACGCCTTAGCCCAGTGTACTAATTATGCTCTCTTGAAATTGGCTGGAGATGTTGAGAGCAACCCAGGTCCCATGCAAATAGAGCTCTCCACCTGCTTCTTTCTGTGCCTTTTGCGATTCTGCTTTAGTGCCACCAGAAGATACTACCTGGGTGCAGTGGAACTGTCATGGATCTTTCTAGAAGATCTCCTACAATATTCTCAGCTGCCATGGAAAATCGATGTTCTTCTTTTATTCTCTCAAGATTTTCAGGCTGTATATTAAAACTTATATTAAGAACTATGCTAACCACCTC
G
TAGATGCAAAGACGCCTTAGCCATGGGGTCCTGCTAATAC
Exon14 Intron13
H
I
195bp-Donor-HDR left junction: Intron13-Exon14-E2A-BDDF8
GCTTTAGGAATTCTAAGTAGAACTGTAATTAAGCAATGCAAGGCACGTACGTTTACTATGTCATTGCCTATGGCTATGAAGTGCAAATCCTAACAGTCCTGCTAATACTTTTCTAACATCCATCATTTCTTTGTTTTCAGGGTCCAAACCTTGTCACTAGATGCAAAGACGCCTTAGCCCAGTGTACTAATTATGCTCTCTTGAAATTGGCTGGAGATGTTGAGAGCAACCCAGGTCCCATGCAAATAGAGCTCTCCACCTGCTTCTTTCTGTGCCTTTTGCGATTCTGCTTTAGTGCCACCAGAAGATACTACCTGGGTGCAGTGGAACTGTCATGGAA
J
TAGATGCAAAGACGCCTTAGCCCAGTGTACTAATTATGCT
Exon14 E2A
Fig. S7. Sanger sequencing demonstrates the integration of BDDF8 by NHEJ and HDR. Hemophilia A mice were hydrodynamically injected with Cas9-sgAlb plasmids and pD-BDDF8-sg (HA85 (A-E) or HA195 (F-G)). The higher band (NHEJ) and lower band (HDR) were purified for Sanger sequencing. The data confirmed the identity of the two bands in the PCR product.

## Slide 14
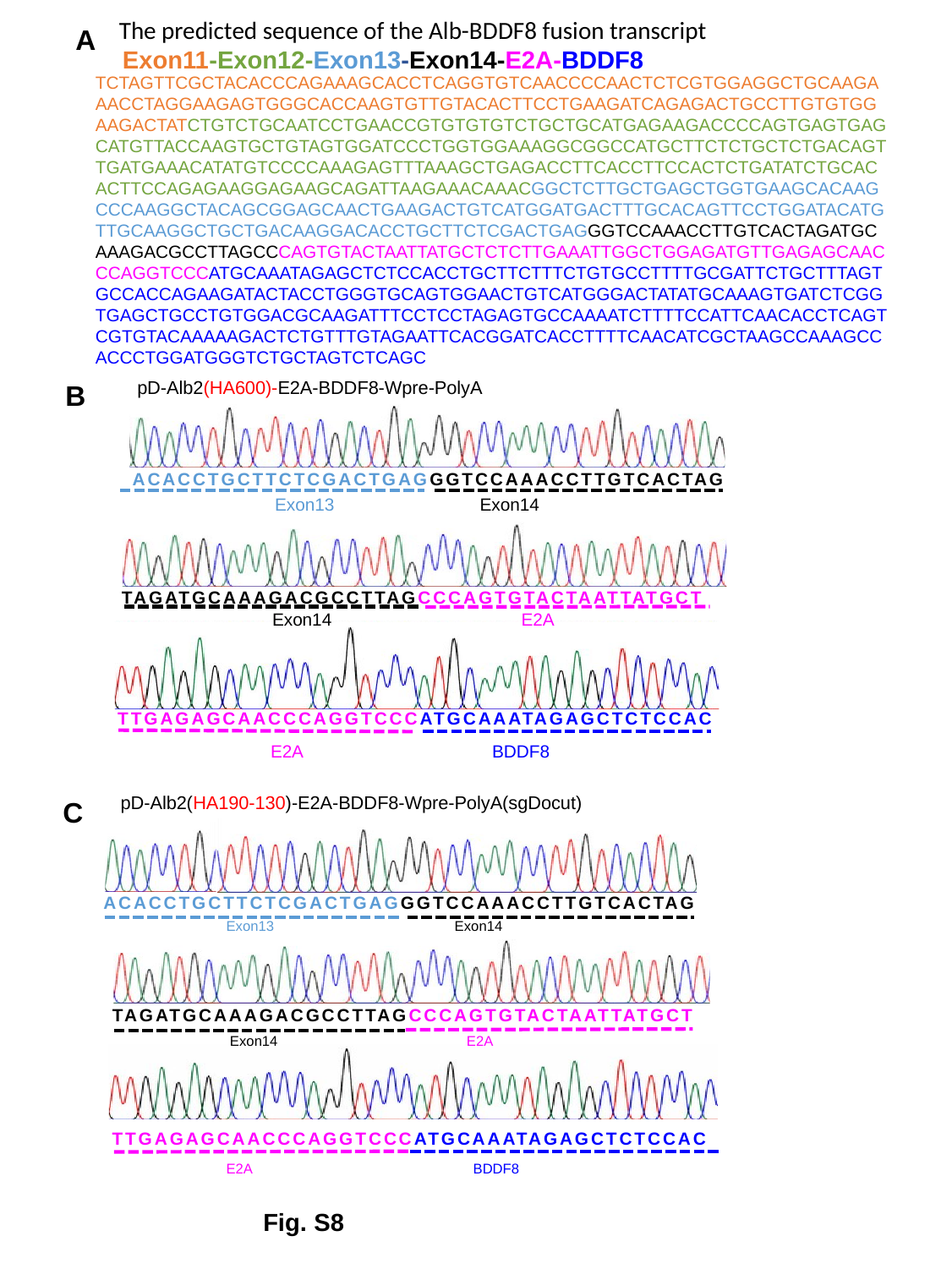

The predicted sequence of the Alb-BDDF8 fusion transcript
A
Exon11-Exon12-Exon13-Exon14-E2A-BDDF8
TCTAGTTCGCTACACCCAGAAAGCACCTCAGGTGTCAACCCCAACTCTCGTGGAGGCTGCAAGAAACCTAGGAAGAGTGGGCACCAAGTGTTGTACACTTCCTGAAGATCAGAGACTGCCTTGTGTGGAAGACTATCTGTCTGCAATCCTGAACCGTGTGTGTCTGCTGCATGAGAAGACCCCAGTGAGTGAGCATGTTACCAAGTGCTGTAGTGGATCCCTGGTGGAAAGGCGGCCATGCTTCTCTGCTCTGACAGTTGATGAAACATATGTCCCCAAAGAGTTTAAAGCTGAGACCTTCACCTTCCACTCTGATATCTGCACACTTCCAGAGAAGGAGAAGCAGATTAAGAAACAAACGGCTCTTGCTGAGCTGGTGAAGCACAAGCCCAAGGCTACAGCGGAGCAACTGAAGACTGTCATGGATGACTTTGCACAGTTCCTGGATACATGTTGCAAGGCTGCTGACAAGGACACCTGCTTCTCGACTGAGGGTCCAAACCTTGTCACTAGATGCAAAGACGCCTTAGCCCAGTGTACTAATTATGCTCTCTTGAAATTGGCTGGAGATGTTGAGAGCAACCCAGGTCCCATGCAAATAGAGCTCTCCACCTGCTTCTTTCTGTGCCTTTTGCGATTCTGCTTTAGTGCCACCAGAAGATACTACCTGGGTGCAGTGGAACTGTCATGGGACTATATGCAAAGTGATCTCGGTGAGCTGCCTGTGGACGCAAGATTTCCTCCTAGAGTGCCAAAATCTTTTCCATTCAACACCTCAGTCGTGTACAAAAAGACTCTGTTTGTAGAATTCACGGATCACCTTTTCAACATCGCTAAGCCAAAGCCACCCTGGATGGGTCTGCTAGTCTCAGC
pD-Alb2(HA600)-E2A-BDDF8-Wpre-PolyA
B
ACACCTGCTTCTCGACTGAGGGTCCAAACCTTGTCACTAG
Exon13 Exon14
TAGATGCAAAGACGCCTTAGCCCAGTGTACTAATTATGCT
Exon14 E2A
TTGAGAGCAACCCAGGTCCCATGCAAATAGAGCTCTCCAC
E2A BDDF8
pD-Alb2(HA190-130)-E2A-BDDF8-Wpre-PolyA(sgDocut)
C
Exon13 Exon14
TAGATGCAAAGACGCCTTAGCCCAGTGTACTAATTATGCT
Exon14 E2A
TTGAGAGCAACCCAGGTCCCATGCAAATAGAGCTCTCCAC
E2A BDDF8
ACACCTGCTTCTCGACTGAGGGTCCAAACCTTGTCACTAG
Fig. S8

## Slide 15
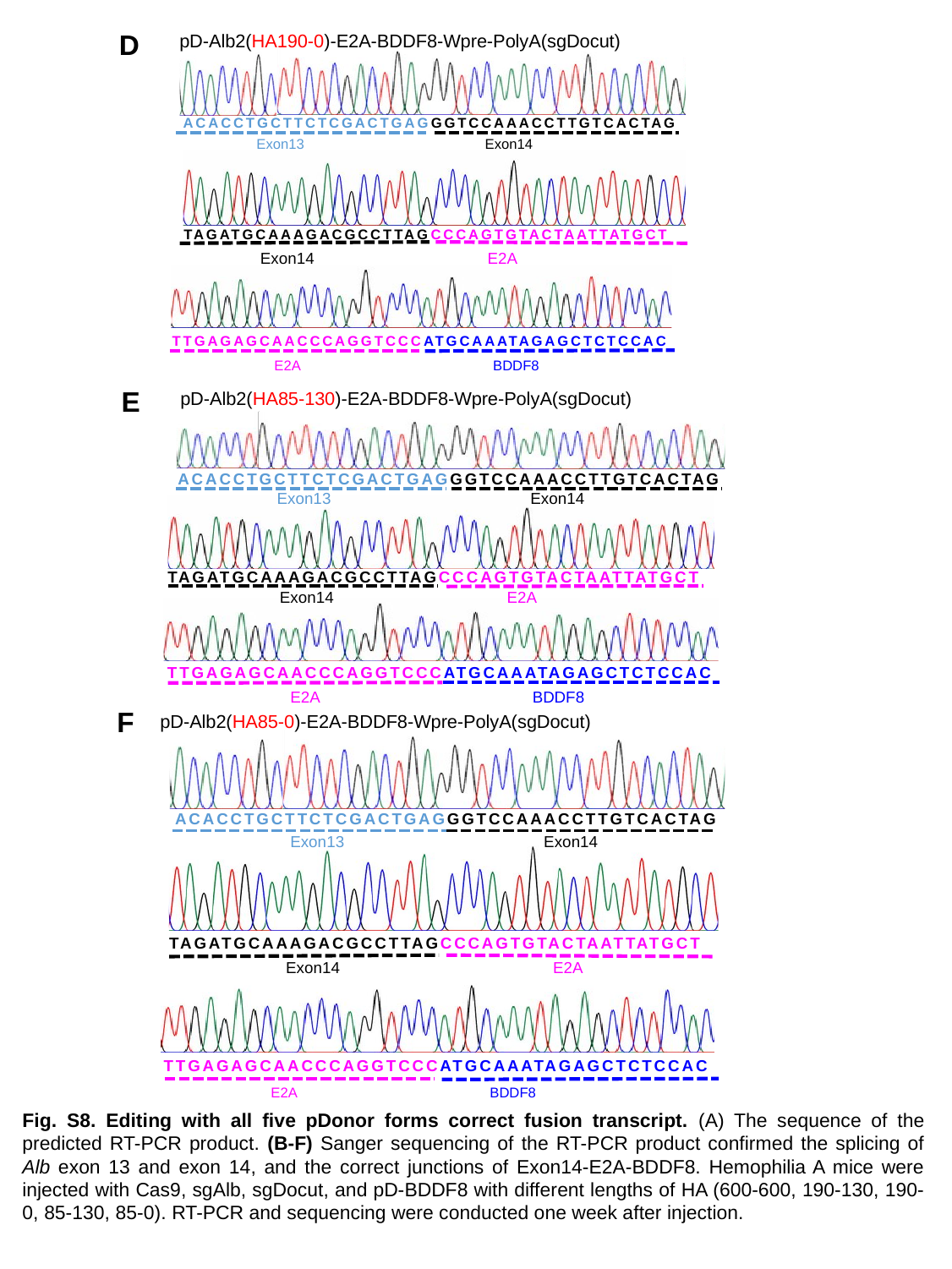

D
pD-Alb2(HA190-0)-E2A-BDDF8-Wpre-PolyA(sgDocut)
ACACCTGCTTCTCGACTGAGGGTCCAAACCTTGTCACTAG
Exon13 Exon14
TAGATGCAAAGACGCCTTAGCCCAGTGTACTAATTATGCT
Exon14 E2A
TTGAGAGCAACCCAGGTCCCATGCAAATAGAGCTCTCCAC
E2A BDDF8
E
pD-Alb2(HA85-130)-E2A-BDDF8-Wpre-PolyA(sgDocut)
ACACCTGCTTCTCGACTGAGGGTCCAAACCTTGTCACTAG
Exon13 Exon14
Exon13 Exon14
TAGATGCAAAGACGCCTTAGCCCAGTGTACTAATTATGCT
Exon14 E2A
TTGAGAGCAACCCAGGTCCCATGCAAATAGAGCTCTCCAC
E2A BDDF8
F
pD-Alb2(HA85-0)-E2A-BDDF8-Wpre-PolyA(sgDocut)
ACACCTGCTTCTCGACTGAGGGTCCAAACCTTGTCACTAG
Exon13 Exon14
TAGATGCAAAGACGCCTTAGCCCAGTGTACTAATTATGCT
Exon14 E2A
TTGAGAGCAACCCAGGTCCCATGCAAATAGAGCTCTCCAC
E2A BDDF8
Exon13 Exon14
Fig. S8. Editing with all five pDonor forms correct fusion transcript. (A) The sequence of the predicted RT-PCR product. (B-F) Sanger sequencing of the RT-PCR product confirmed the splicing of Alb exon 13 and exon 14, and the correct junctions of Exon14-E2A-BDDF8. Hemophilia A mice were injected with Cas9, sgAlb, sgDocut, and pD-BDDF8 with different lengths of HA (600-600, 190-130, 190-0, 85-130, 85-0). RT-PCR and sequencing were conducted one week after injection.

## Slide 16
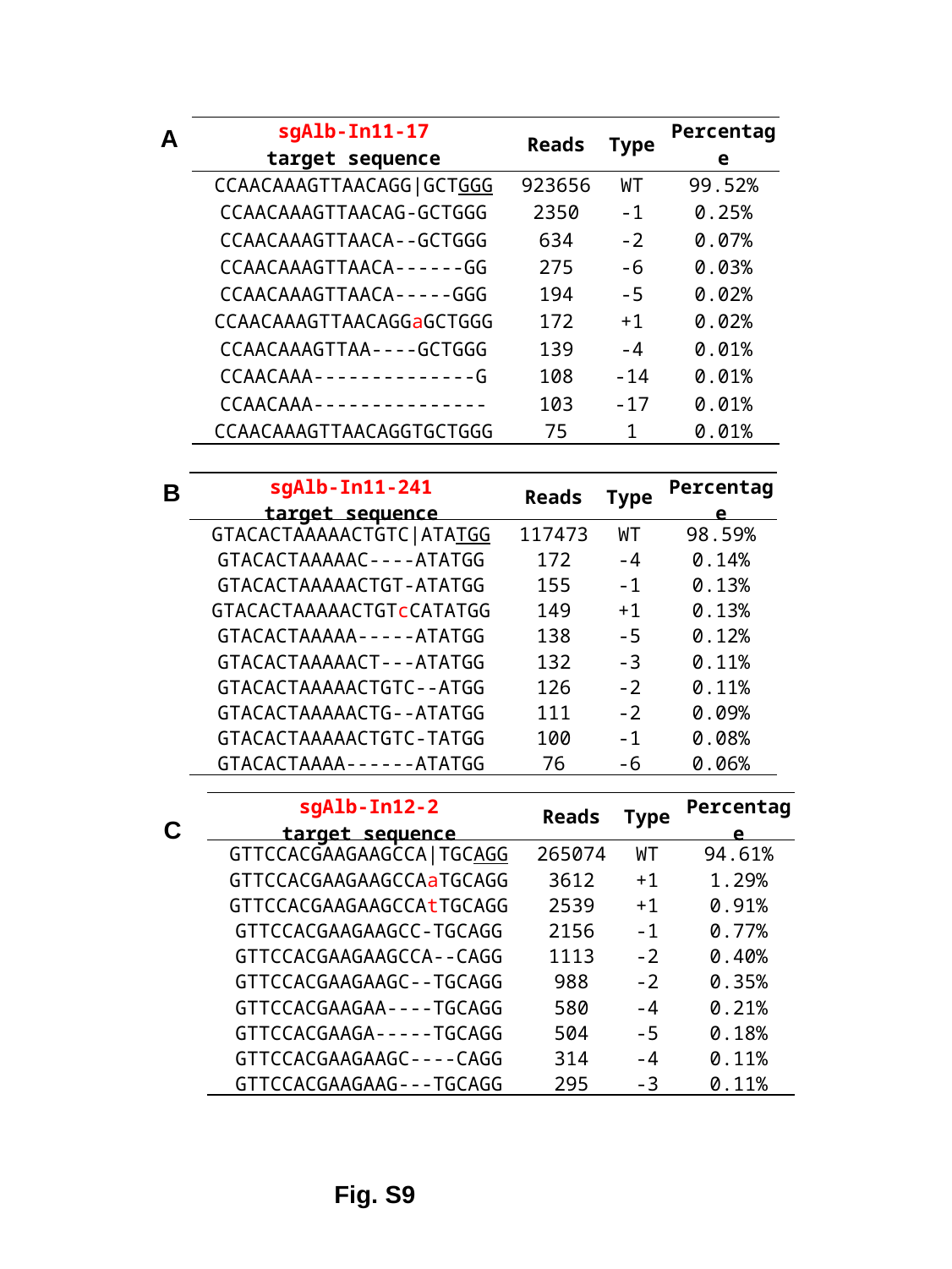

A
| sgAlb-In11-17 target sequence | Reads | Type | Percentage |
| --- | --- | --- | --- |
| CCAACAAAGTTAACAGG|GCTGGG | 923656 | WT | 99.52% |
| CCAACAAAGTTAACAG-GCTGGG | 2350 | -1 | 0.25% |
| CCAACAAAGTTAACA--GCTGGG | 634 | -2 | 0.07% |
| CCAACAAAGTTAACA------GG | 275 | -6 | 0.03% |
| CCAACAAAGTTAACA-----GGG | 194 | -5 | 0.02% |
| CCAACAAAGTTAACAGGaGCTGGG | 172 | +1 | 0.02% |
| CCAACAAAGTTAA----GCTGGG | 139 | -4 | 0.01% |
| CCAACAAA--------------G | 108 | -14 | 0.01% |
| CCAACAAA--------------- | 103 | -17 | 0.01% |
| CCAACAAAGTTAACAGGTGCTGGG | 75 | 1 | 0.01% |
B
| sgAlb-In11-241 target sequence | Reads | Type | Percentage |
| --- | --- | --- | --- |
| GTACACTAAAAACTGTC|ATATGG | 117473 | WT | 98.59% |
| GTACACTAAAAAC----ATATGG | 172 | -4 | 0.14% |
| GTACACTAAAAACTGT-ATATGG | 155 | -1 | 0.13% |
| GTACACTAAAAACTGTcCATATGG | 149 | +1 | 0.13% |
| GTACACTAAAAA-----ATATGG | 138 | -5 | 0.12% |
| GTACACTAAAAACT---ATATGG | 132 | -3 | 0.11% |
| GTACACTAAAAACTGTC--ATGG | 126 | -2 | 0.11% |
| GTACACTAAAAACTG--ATATGG | 111 | -2 | 0.09% |
| GTACACTAAAAACTGTC-TATGG | 100 | -1 | 0.08% |
| GTACACTAAAA------ATATGG | 76 | -6 | 0.06% |
| sgAlb-In12-2 target sequence | Reads | Type | Percentage |
| --- | --- | --- | --- |
| GTTCCACGAAGAAGCCA|TGCAGG | 265074 | WT | 94.61% |
| GTTCCACGAAGAAGCCAaTGCAGG | 3612 | +1 | 1.29% |
| GTTCCACGAAGAAGCCAtTGCAGG | 2539 | +1 | 0.91% |
| GTTCCACGAAGAAGCC-TGCAGG | 2156 | -1 | 0.77% |
| GTTCCACGAAGAAGCCA--CAGG | 1113 | -2 | 0.40% |
| GTTCCACGAAGAAGC--TGCAGG | 988 | -2 | 0.35% |
| GTTCCACGAAGAA----TGCAGG | 580 | -4 | 0.21% |
| GTTCCACGAAGA-----TGCAGG | 504 | -5 | 0.18% |
| GTTCCACGAAGAAGC----CAGG | 314 | -4 | 0.11% |
| GTTCCACGAAGAAG---TGCAGG | 295 | -3 | 0.11% |
C
Fig. S9

## Slide 17
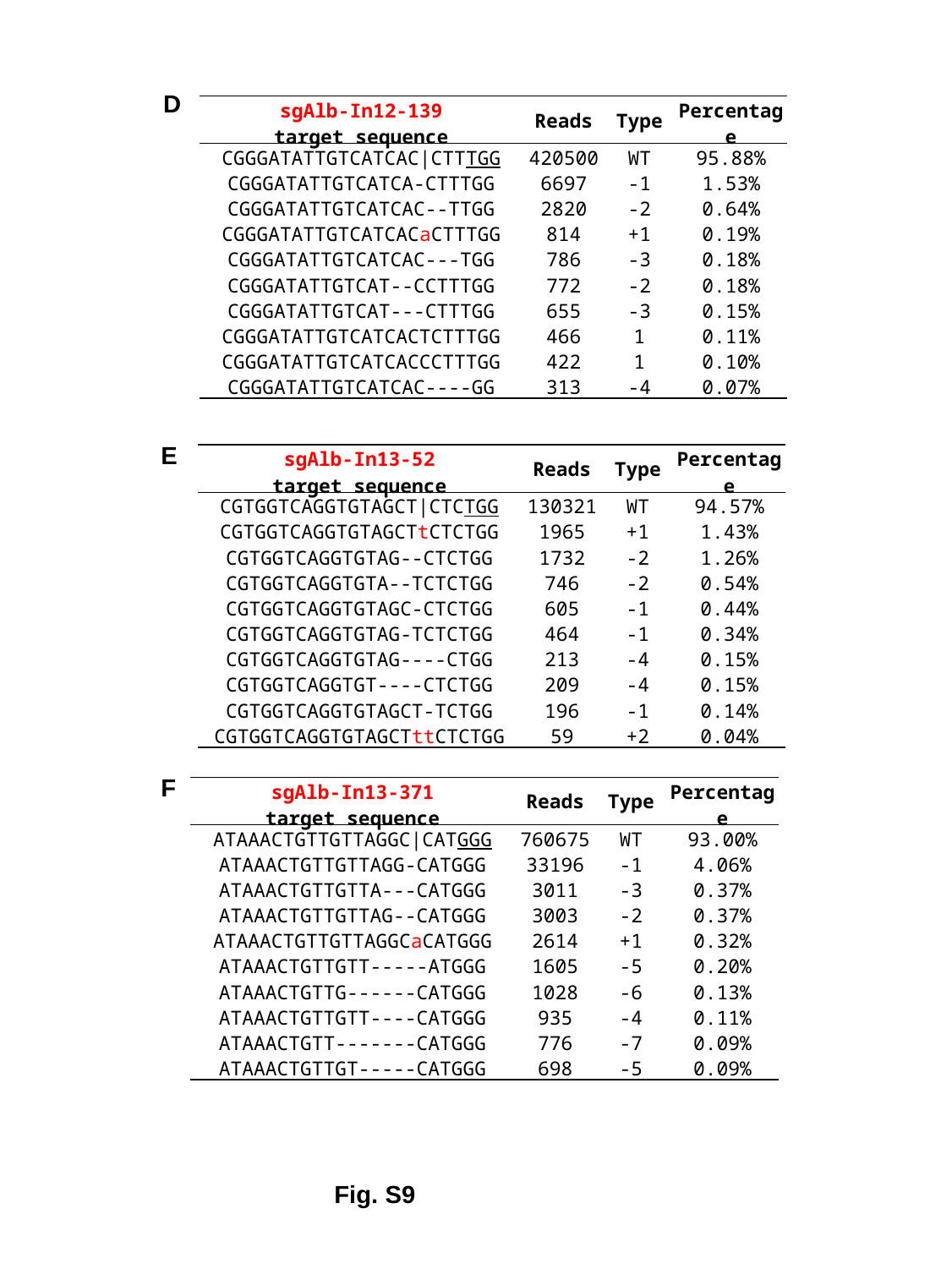

D
| sgAlb-In12-139 target sequence | Reads | Type | Percentage |
| --- | --- | --- | --- |
| CGGGATATTGTCATCAC|CTTTGG | 420500 | WT | 95.88% |
| CGGGATATTGTCATCA-CTTTGG | 6697 | -1 | 1.53% |
| CGGGATATTGTCATCAC--TTGG | 2820 | -2 | 0.64% |
| CGGGATATTGTCATCACaCTTTGG | 814 | +1 | 0.19% |
| CGGGATATTGTCATCAC---TGG | 786 | -3 | 0.18% |
| CGGGATATTGTCAT--CCTTTGG | 772 | -2 | 0.18% |
| CGGGATATTGTCAT---CTTTGG | 655 | -3 | 0.15% |
| CGGGATATTGTCATCACTCTTTGG | 466 | 1 | 0.11% |
| CGGGATATTGTCATCACCCTTTGG | 422 | 1 | 0.10% |
| CGGGATATTGTCATCAC----GG | 313 | -4 | 0.07% |
E
| sgAlb-In13-52 target sequence | Reads | Type | Percentage |
| --- | --- | --- | --- |
| CGTGGTCAGGTGTAGCT|CTCTGG | 130321 | WT | 94.57% |
| CGTGGTCAGGTGTAGCTtCTCTGG | 1965 | +1 | 1.43% |
| CGTGGTCAGGTGTAG--CTCTGG | 1732 | -2 | 1.26% |
| CGTGGTCAGGTGTA--TCTCTGG | 746 | -2 | 0.54% |
| CGTGGTCAGGTGTAGC-CTCTGG | 605 | -1 | 0.44% |
| CGTGGTCAGGTGTAG-TCTCTGG | 464 | -1 | 0.34% |
| CGTGGTCAGGTGTAG----CTGG | 213 | -4 | 0.15% |
| CGTGGTCAGGTGT----CTCTGG | 209 | -4 | 0.15% |
| CGTGGTCAGGTGTAGCT-TCTGG | 196 | -1 | 0.14% |
| CGTGGTCAGGTGTAGCTttCTCTGG | 59 | +2 | 0.04% |
F
| sgAlb-In13-371 target sequence | Reads | Type | Percentage |
| --- | --- | --- | --- |
| ATAAACTGTTGTTAGGC|CATGGG | 760675 | WT | 93.00% |
| ATAAACTGTTGTTAGG-CATGGG | 33196 | -1 | 4.06% |
| ATAAACTGTTGTTA---CATGGG | 3011 | -3 | 0.37% |
| ATAAACTGTTGTTAG--CATGGG | 3003 | -2 | 0.37% |
| ATAAACTGTTGTTAGGCaCATGGG | 2614 | +1 | 0.32% |
| ATAAACTGTTGTT-----ATGGG | 1605 | -5 | 0.20% |
| ATAAACTGTTG------CATGGG | 1028 | -6 | 0.13% |
| ATAAACTGTTGTT----CATGGG | 935 | -4 | 0.11% |
| ATAAACTGTT-------CATGGG | 776 | -7 | 0.09% |
| ATAAACTGTTGT-----CATGGG | 698 | -5 | 0.09% |
Fig. S9

## Slide 18
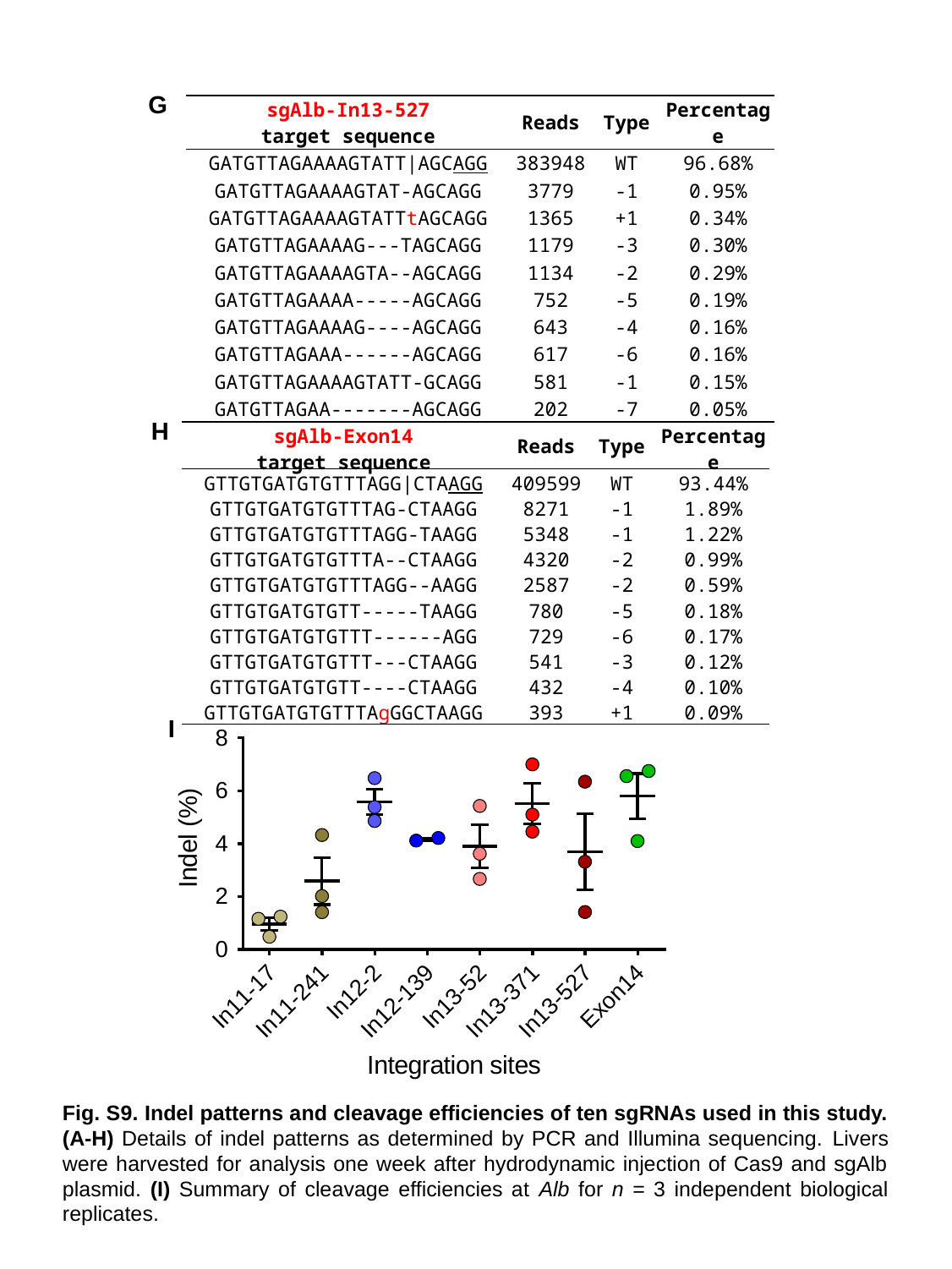

G
| sgAlb-In13-527 target sequence | Reads | Type | Percentage |
| --- | --- | --- | --- |
| GATGTTAGAAAAGTATT|AGCAGG | 383948 | WT | 96.68% |
| GATGTTAGAAAAGTAT-AGCAGG | 3779 | -1 | 0.95% |
| GATGTTAGAAAAGTATTtAGCAGG | 1365 | +1 | 0.34% |
| GATGTTAGAAAAG---TAGCAGG | 1179 | -3 | 0.30% |
| GATGTTAGAAAAGTA--AGCAGG | 1134 | -2 | 0.29% |
| GATGTTAGAAAA-----AGCAGG | 752 | -5 | 0.19% |
| GATGTTAGAAAAG----AGCAGG | 643 | -4 | 0.16% |
| GATGTTAGAAA------AGCAGG | 617 | -6 | 0.16% |
| GATGTTAGAAAAGTATT-GCAGG | 581 | -1 | 0.15% |
| GATGTTAGAA-------AGCAGG | 202 | -7 | 0.05% |
H
| sgAlb-Exon14 target sequence | Reads | Type | Percentage |
| --- | --- | --- | --- |
| GTTGTGATGTGTTTAGG|CTAAGG | 409599 | WT | 93.44% |
| GTTGTGATGTGTTTAG-CTAAGG | 8271 | -1 | 1.89% |
| GTTGTGATGTGTTTAGG-TAAGG | 5348 | -1 | 1.22% |
| GTTGTGATGTGTTTA--CTAAGG | 4320 | -2 | 0.99% |
| GTTGTGATGTGTTTAGG--AAGG | 2587 | -2 | 0.59% |
| GTTGTGATGTGTT-----TAAGG | 780 | -5 | 0.18% |
| GTTGTGATGTGTTT------AGG | 729 | -6 | 0.17% |
| GTTGTGATGTGTTT---CTAAGG | 541 | -3 | 0.12% |
| GTTGTGATGTGTT----CTAAGG | 432 | -4 | 0.10% |
| GTTGTGATGTGTTTAgGGCTAAGG | 393 | +1 | 0.09% |
I
Fig. S9. Indel patterns and cleavage efficiencies of ten sgRNAs used in this study. (A-H) Details of indel patterns as determined by PCR and Illumina sequencing. Livers were harvested for analysis one week after hydrodynamic injection of Cas9 and sgAlb plasmid. (I) Summary of cleavage efficiencies at Alb for n = 3 independent biological replicates.

## Slide 19
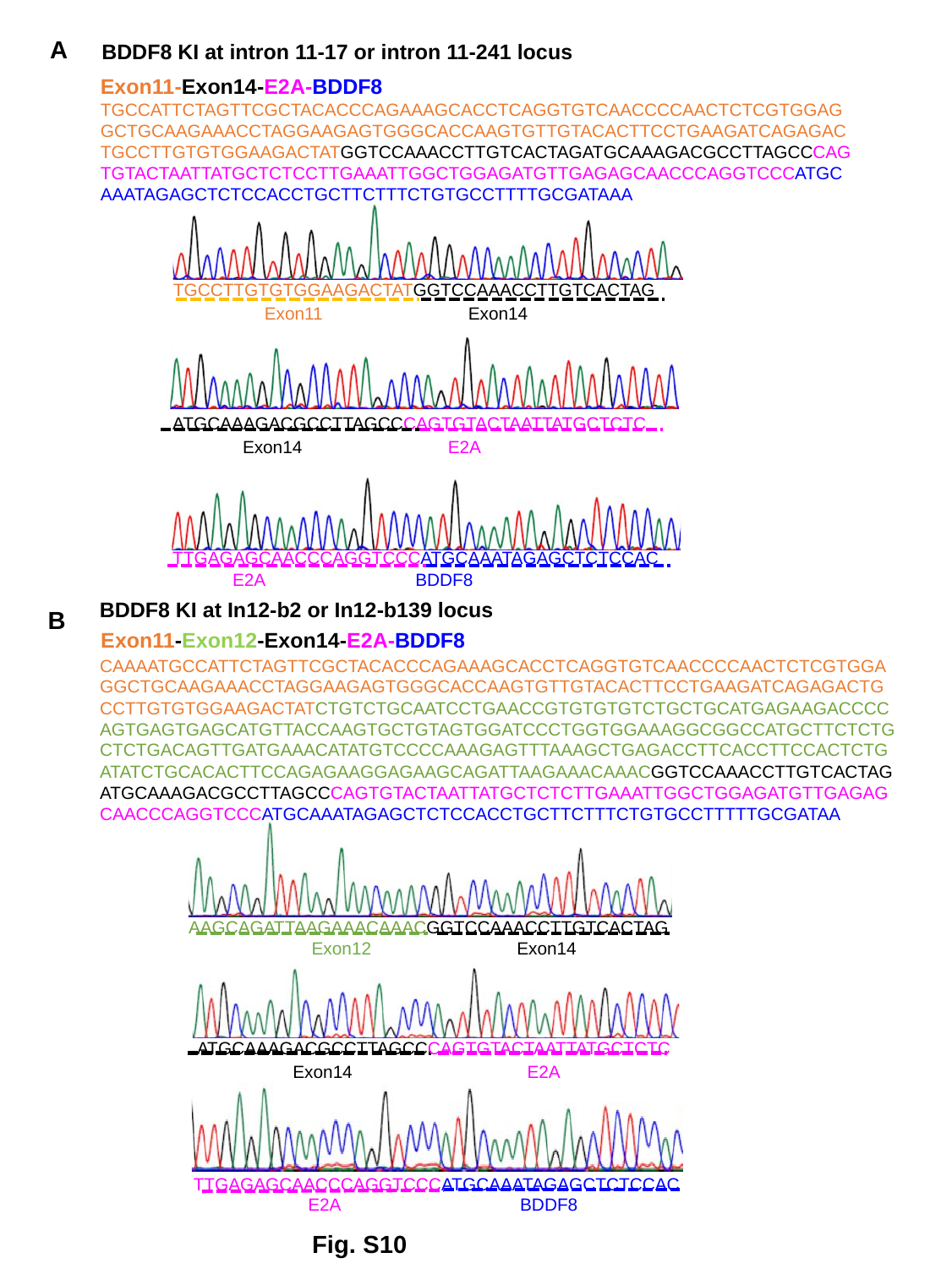

A
BDDF8 KI at intron 11-17 or intron 11-241 locus
Exon11-Exon14-E2A-BDDF8
TGCCATTCTAGTTCGCTACACCCAGAAAGCACCTCAGGTGTCAACCCCAACTCTCGTGGAGGCTGCAAGAAACCTAGGAAGAGTGGGCACCAAGTGTTGTACACTTCCTGAAGATCAGAGACTGCCTTGTGTGGAAGACTATGGTCCAAACCTTGTCACTAGATGCAAAGACGCCTTAGCCCAGTGTACTAATTATGCTCTCCTTGAAATTGGCTGGAGATGTTGAGAGCAACCCAGGTCCCATGCAAATAGAGCTCTCCACCTGCTTCTTTCTGTGCCTTTTGCGATAAA
Exon11 Exon14
Exon14 E2A
ATGCAAAGACGCCTTAGCCCAGTGTACTAATTATGCTCTC
E2A BDDF8
TTGAGAGCAACCCAGGTCCCATGCAAATAGAGCTCTCCAC
TGCCTTGTGTGGAAGACTATGGTCCAAACCTTGTCACTAG
BDDF8 KI at In12-b2 or In12-b139 locus
B
Exon11-Exon12-Exon14-E2A-BDDF8
CAAAATGCCATTCTAGTTCGCTACACCCAGAAAGCACCTCAGGTGTCAACCCCAACTCTCGTGGAGGCTGCAAGAAACCTAGGAAGAGTGGGCACCAAGTGTTGTACACTTCCTGAAGATCAGAGACTGCCTTGTGTGGAAGACTATCTGTCTGCAATCCTGAACCGTGTGTGTCTGCTGCATGAGAAGACCCCAGTGAGTGAGCATGTTACCAAGTGCTGTAGTGGATCCCTGGTGGAAAGGCGGCCATGCTTCTCTGCTCTGACAGTTGATGAAACATATGTCCCCAAAGAGTTTAAAGCTGAGACCTTCACCTTCCACTCTGATATCTGCACACTTCCAGAGAAGGAGAAGCAGATTAAGAAACAAACGGTCCAAACCTTGTCACTAGATGCAAAGACGCCTTAGCCCAGTGTACTAATTATGCTCTCTTGAAATTGGCTGGAGATGTTGAGAGCAACCCAGGTCCCATGCAAATAGAGCTCTCCACCTGCTTCTTTCTGTGCCTTTTTGCGATAA
AAGCAGATTAAGAAACAAACGGTCCAAACCTTGTCACTAG
Exon12 Exon14
ATGCAAAGACGCCTTAGCCCAGTGTACTAATTATGCTCTC
Exon14 E2A
TTGAGAGCAACCCAGGTCCCATGCAAATAGAGCTCTCCAC
E2A BDDF8
Fig. S10

## Slide 20
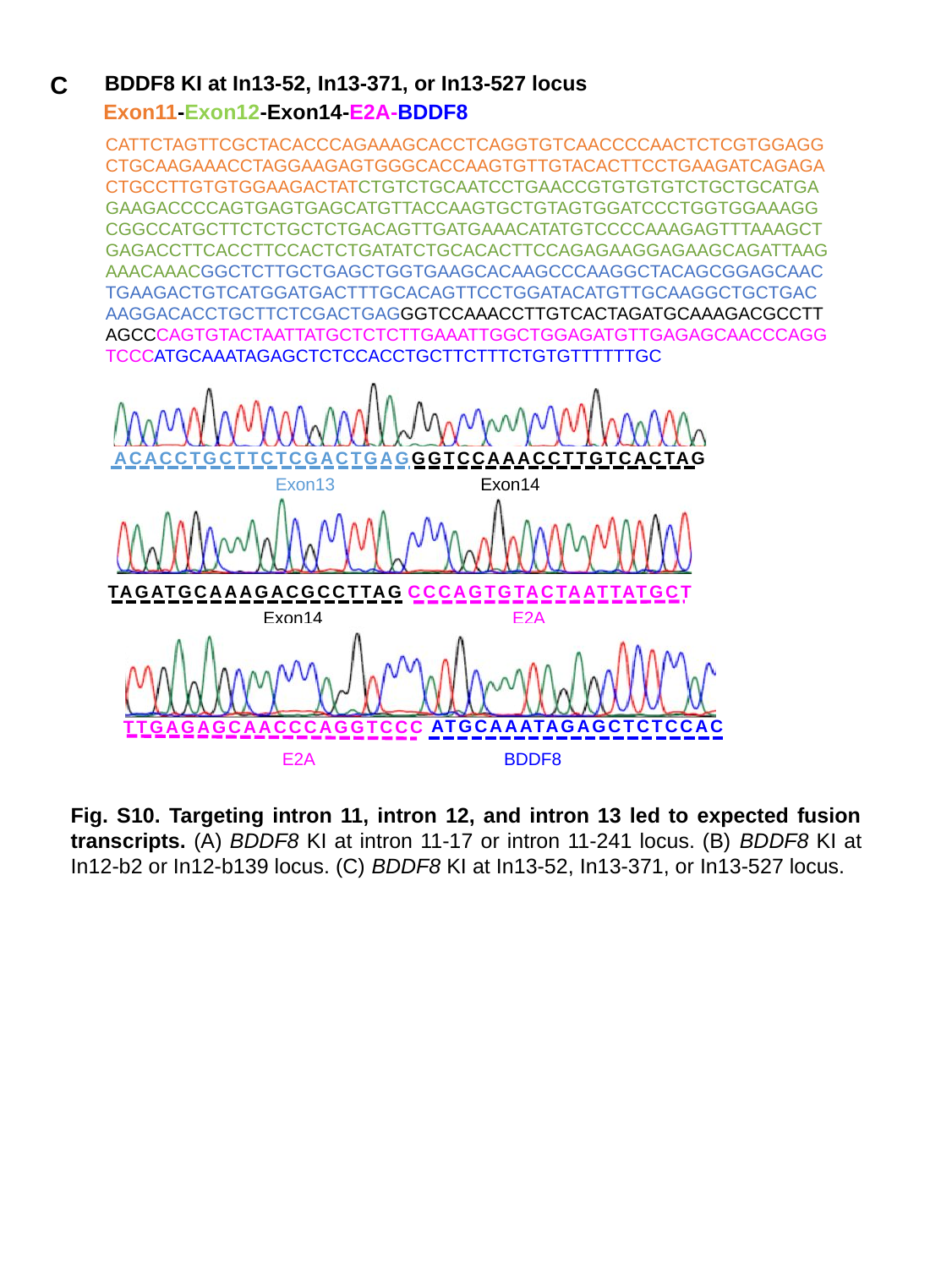

C
BDDF8 KI at In13-52, In13-371, or In13-527 locus
Exon11-Exon12-Exon14-E2A-BDDF8
CATTCTAGTTCGCTACACCCAGAAAGCACCTCAGGTGTCAACCCCAACTCTCGTGGAGGCTGCAAGAAACCTAGGAAGAGTGGGCACCAAGTGTTGTACACTTCCTGAAGATCAGAGACTGCCTTGTGTGGAAGACTATCTGTCTGCAATCCTGAACCGTGTGTGTCTGCTGCATGAGAAGACCCCAGTGAGTGAGCATGTTACCAAGTGCTGTAGTGGATCCCTGGTGGAAAGGCGGCCATGCTTCTCTGCTCTGACAGTTGATGAAACATATGTCCCCAAAGAGTTTAAAGCTGAGACCTTCACCTTCCACTCTGATATCTGCACACTTCCAGAGAAGGAGAAGCAGATTAAGAAACAAACGGCTCTTGCTGAGCTGGTGAAGCACAAGCCCAAGGCTACAGCGGAGCAACTGAAGACTGTCATGGATGACTTTGCACAGTTCCTGGATACATGTTGCAAGGCTGCTGACAAGGACACCTGCTTCTCGACTGAGGGTCCAAACCTTGTCACTAGATGCAAAGACGCCTTAGCCCAGTGTACTAATTATGCTCTCTTGAAATTGGCTGGAGATGTTGAGAGCAACCCAGGTCCCATGCAAATAGAGCTCTCCACCTGCTTCTTTCTGTGTTTTTTGC
ACACCTGCTTCTCGACTGAGGGTCCAAACCTTGTCACTAG
Exon13 Exon14
TAGATGCAAAGACGCCTTAG
CCCAGTGTACTAATTATGCT
Exon14 E2A
ATGCAAATAGAGCTCTCCAC
TTGAGAGCAACCCAGGTCCC
E2A BDDF8
Fig. S10. Targeting intron 11, intron 12, and intron 13 led to expected fusion transcripts. (A) BDDF8 KI at intron 11-17 or intron 11-241 locus. (B) BDDF8 KI at In12-b2 or In12-b139 locus. (C) BDDF8 KI at In13-52, In13-371, or In13-527 locus.

## Slide 21
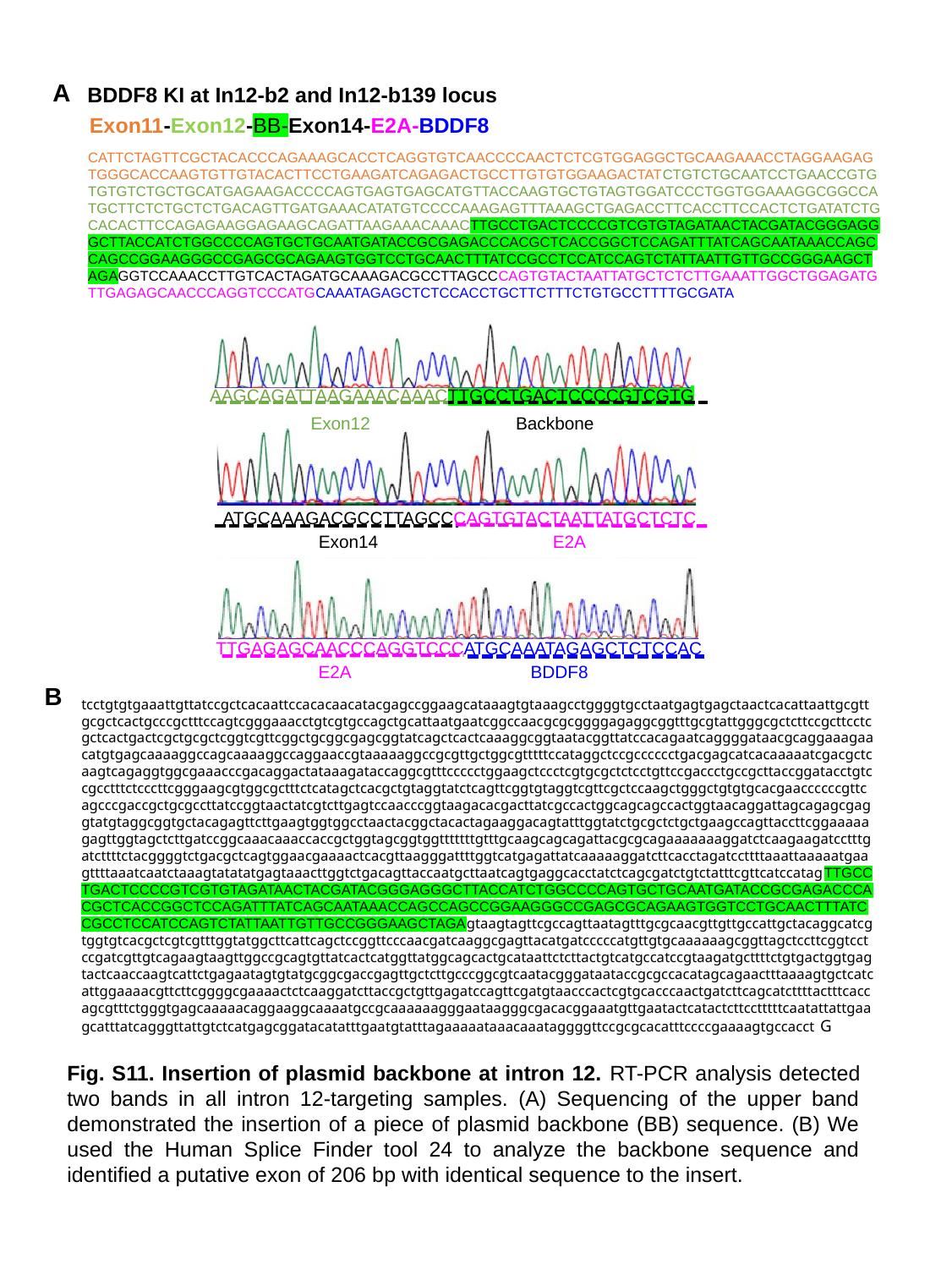

A
BDDF8 KI at In12-b2 and In12-b139 locus
Exon11-Exon12-BB-Exon14-E2A-BDDF8
CATTCTAGTTCGCTACACCCAGAAAGCACCTCAGGTGTCAACCCCAACTCTCGTGGAGGCTGCAAGAAACCTAGGAAGAGTGGGCACCAAGTGTTGTACACTTCCTGAAGATCAGAGACTGCCTTGTGTGGAAGACTATCTGTCTGCAATCCTGAACCGTGTGTGTCTGCTGCATGAGAAGACCCCAGTGAGTGAGCATGTTACCAAGTGCTGTAGTGGATCCCTGGTGGAAAGGCGGCCATGCTTCTCTGCTCTGACAGTTGATGAAACATATGTCCCCAAAGAGTTTAAAGCTGAGACCTTCACCTTCCACTCTGATATCTGCACACTTCCAGAGAAGGAGAAGCAGATTAAGAAACAAACTTGCCTGACTCCCCGTCGTGTAGATAACTACGATACGGGAGGGCTTACCATCTGGCCCCAGTGCTGCAATGATACCGCGAGACCCACGCTCACCGGCTCCAGATTTATCAGCAATAAACCAGCCAGCCGGAAGGGCCGAGCGCAGAAGTGGTCCTGCAACTTTATCCGCCTCCATCCAGTCTATTAATTGTTGCCGGGAAGCTAGAGGTCCAAACCTTGTCACTAGATGCAAAGACGCCTTAGCCCAGTGTACTAATTATGCTCTCTTGAAATTGGCTGGAGATGTTGAGAGCAACCCAGGTCCCATGCAAATAGAGCTCTCCACCTGCTTCTTTCTGTGCCTTTTGCGATA
Exon12 Backbone
ATGCAAAGACGCCTTAGCCCAGTGTACTAATTATGCTCTC
Exon14 E2A
TTGAGAGCAACCCAGGTCCCATGCAAATAGAGCTCTCCAC
E2A BDDF8
AAGCAGATTAAGAAACAAACTTGCCTGACTCCCCGTCGTG
B
tcctgtgtgaaattgttatccgctcacaattccacacaacatacgagccggaagcataaagtgtaaagcctggggtgcctaatgagtgagctaactcacattaattgcgttgcgctcactgcccgctttccagtcgggaaacctgtcgtgccagctgcattaatgaatcggccaacgcgcggggagaggcggtttgcgtattgggcgctcttccgcttcctcgctcactgactcgctgcgctcggtcgttcggctgcggcgagcggtatcagctcactcaaaggcggtaatacggttatccacagaatcaggggataacgcaggaaagaacatgtgagcaaaaggccagcaaaaggccaggaaccgtaaaaaggccgcgttgctggcgtttttccataggctccgcccccctgacgagcatcacaaaaatcgacgctcaagtcagaggtggcgaaacccgacaggactataaagataccaggcgtttccccctggaagctccctcgtgcgctctcctgttccgaccctgccgcttaccggatacctgtccgcctttctcccttcgggaagcgtggcgctttctcatagctcacgctgtaggtatctcagttcggtgtaggtcgttcgctccaagctgggctgtgtgcacgaaccccccgttcagcccgaccgctgcgccttatccggtaactatcgtcttgagtccaacccggtaagacacgacttatcgccactggcagcagccactggtaacaggattagcagagcgaggtatgtaggcggtgctacagagttcttgaagtggtggcctaactacggctacactagaaggacagtatttggtatctgcgctctgctgaagccagttaccttcggaaaaagagttggtagctcttgatccggcaaacaaaccaccgctggtagcggtggtttttttgtttgcaagcagcagattacgcgcagaaaaaaaggatctcaagaagatcctttgatcttttctacggggtctgacgctcagtggaacgaaaactcacgttaagggattttggtcatgagattatcaaaaaggatcttcacctagatccttttaaattaaaaatgaagttttaaatcaatctaaagtatatatgagtaaacttggtctgacagttaccaatgcttaatcagtgaggcacctatctcagcgatctgtctatttcgttcatccatagTTGCCTGACTCCCCGTCGTGTAGATAACTACGATACGGGAGGGCTTACCATCTGGCCCCAGTGCTGCAATGATACCGCGAGACCCACGCTCACCGGCTCCAGATTTATCAGCAATAAACCAGCCAGCCGGAAGGGCCGAGCGCAGAAGTGGTCCTGCAACTTTATCCGCCTCCATCCAGTCTATTAATTGTTGCCGGGAAGCTAGAgtaagtagttcgccagttaatagtttgcgcaacgttgttgccattgctacaggcatcgtggtgtcacgctcgtcgtttggtatggcttcattcagctccggttcccaacgatcaaggcgagttacatgatcccccatgttgtgcaaaaaagcggttagctccttcggtcctccgatcgttgtcagaagtaagttggccgcagtgttatcactcatggttatggcagcactgcataattctcttactgtcatgccatccgtaagatgcttttctgtgactggtgagtactcaaccaagtcattctgagaatagtgtatgcggcgaccgagttgctcttgcccggcgtcaatacgggataataccgcgccacatagcagaactttaaaagtgctcatcattggaaaacgttcttcggggcgaaaactctcaaggatcttaccgctgttgagatccagttcgatgtaacccactcgtgcacccaactgatcttcagcatcttttactttcaccagcgtttctgggtgagcaaaaacaggaaggcaaaatgccgcaaaaaagggaataagggcgacacggaaatgttgaatactcatactcttcctttttcaatattattgaagcatttatcagggttattgtctcatgagcggatacatatttgaatgtatttagaaaaataaacaaataggggttccgcgcacatttccccgaaaagtgccacct G
Fig. S11. Insertion of plasmid backbone at intron 12. RT-PCR analysis detected two bands in all intron 12-targeting samples. (A) Sequencing of the upper band demonstrated the insertion of a piece of plasmid backbone (BB) sequence. (B) We used the Human Splice Finder tool 24 to analyze the backbone sequence and identified a putative exon of 206 bp with identical sequence to the insert.

## Slide 22
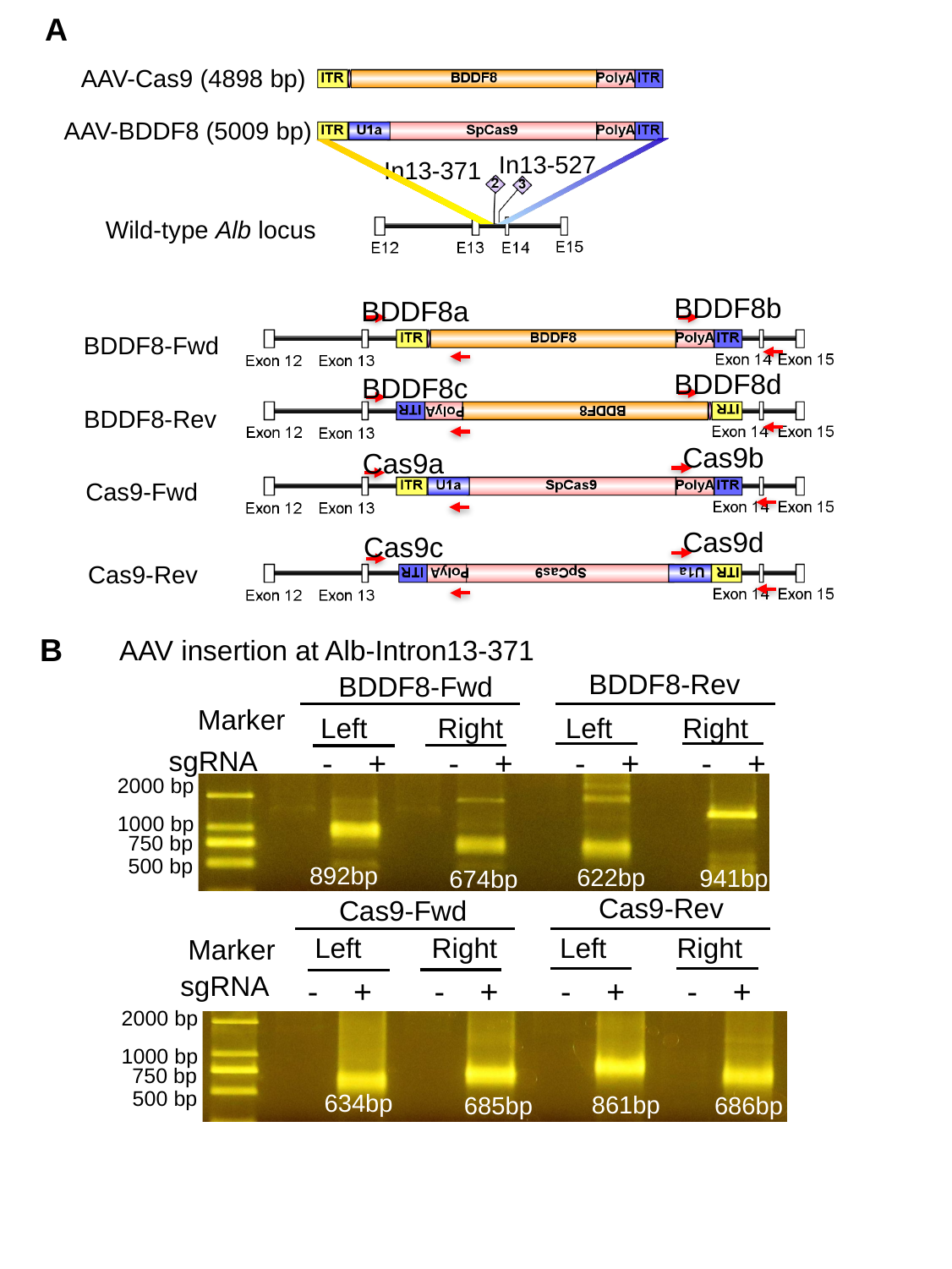

A
AAV-Cas9 (4898 bp)
AAV-BDDF8 (5009 bp)
In13-527
In13-371
Wild-type Alb locus
BDDF8b
BDDF8a
BDDF8-Fwd
BDDF8d
BDDF8c
BDDF8-Rev
Cas9b
Cas9a
Cas9-Fwd
Cas9d
Cas9c
Cas9-Rev
AAV insertion at Alb-Intron13-371
BDDF8-Rev
BDDF8-Fwd
Marker
 - + - + - + - +
 Left Right Left Right
sgRNA
Cas9-Rev
Cas9-Fwd
Marker
 Left Right Left Right
sgRNA
- + - + - + - +
B
2000 bp
1000 bp
750 bp
500 bp
892bp
622bp
941bp
674bp
2000 bp
1000 bp
750 bp
500 bp
634bp
861bp
686bp
685bp

## Slide 23
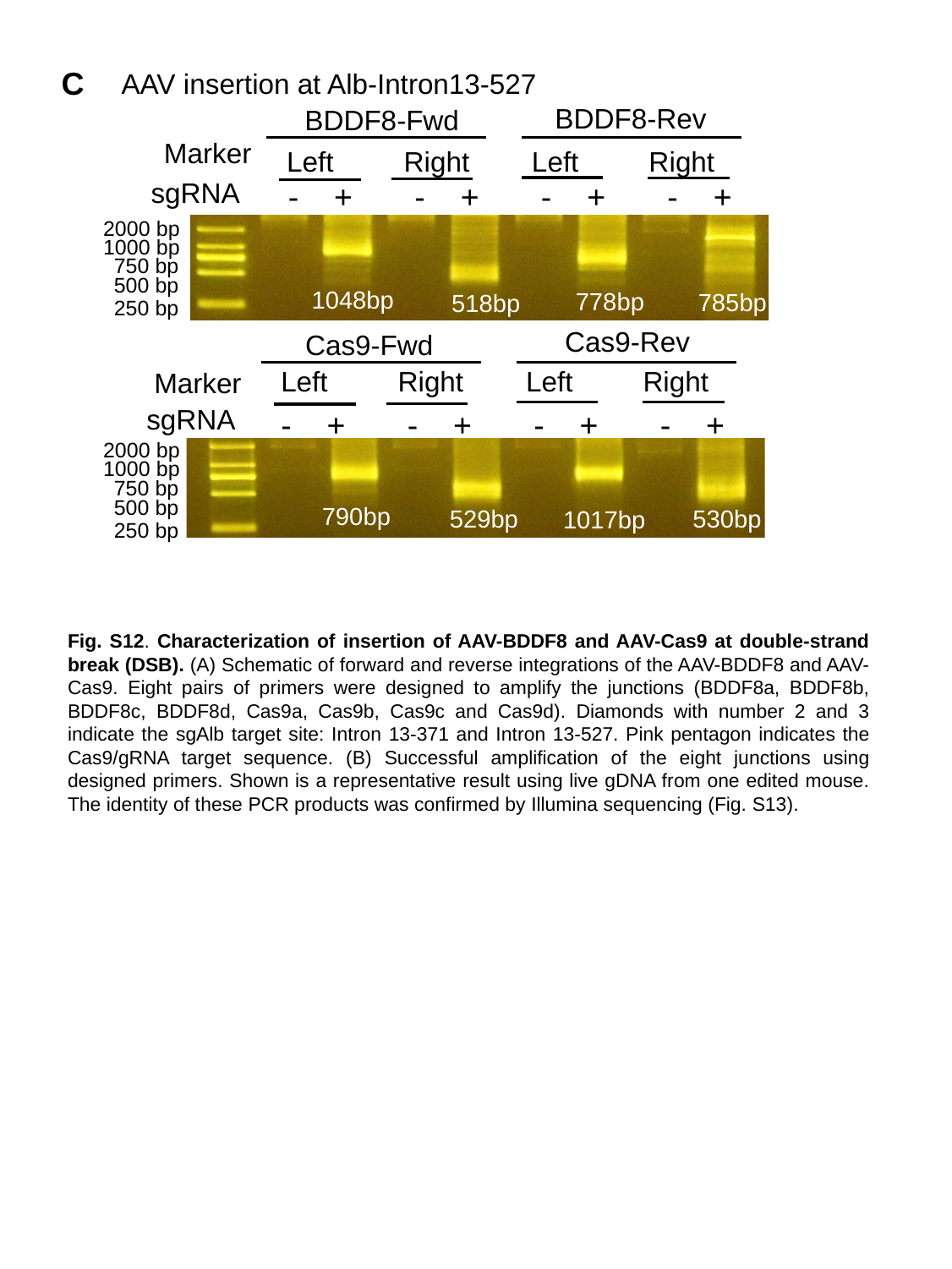

C
AAV insertion at Alb-Intron13-527
BDDF8-Rev
BDDF8-Fwd
Marker
 - + - + - + - +
 Left Right Left Right
sgRNA
Cas9-Rev
Cas9-Fwd
Marker
 Left Right Left Right
sgRNA
- + - + - + - +
2000 bp
1000 bp
750 bp
500 bp
1048bp
778bp
785bp
518bp
250 bp
2000 bp
1000 bp
750 bp
500 bp
790bp
530bp
529bp
1017bp
250 bp
Fig. S12. Characterization of insertion of AAV-BDDF8 and AAV-Cas9 at double-strand break (DSB). (A) Schematic of forward and reverse integrations of the AAV-BDDF8 and AAV-Cas9. Eight pairs of primers were designed to amplify the junctions (BDDF8a, BDDF8b, BDDF8c, BDDF8d, Cas9a, Cas9b, Cas9c and Cas9d). Diamonds with number 2 and 3 indicate the sgAlb target site: Intron 13-371 and Intron 13-527. Pink pentagon indicates the Cas9/gRNA target sequence. (B) Successful amplification of the eight junctions using designed primers. Shown is a representative result using live gDNA from one edited mouse. The identity of these PCR products was confirmed by Illumina sequencing (Fig. S13).

## Slide 24
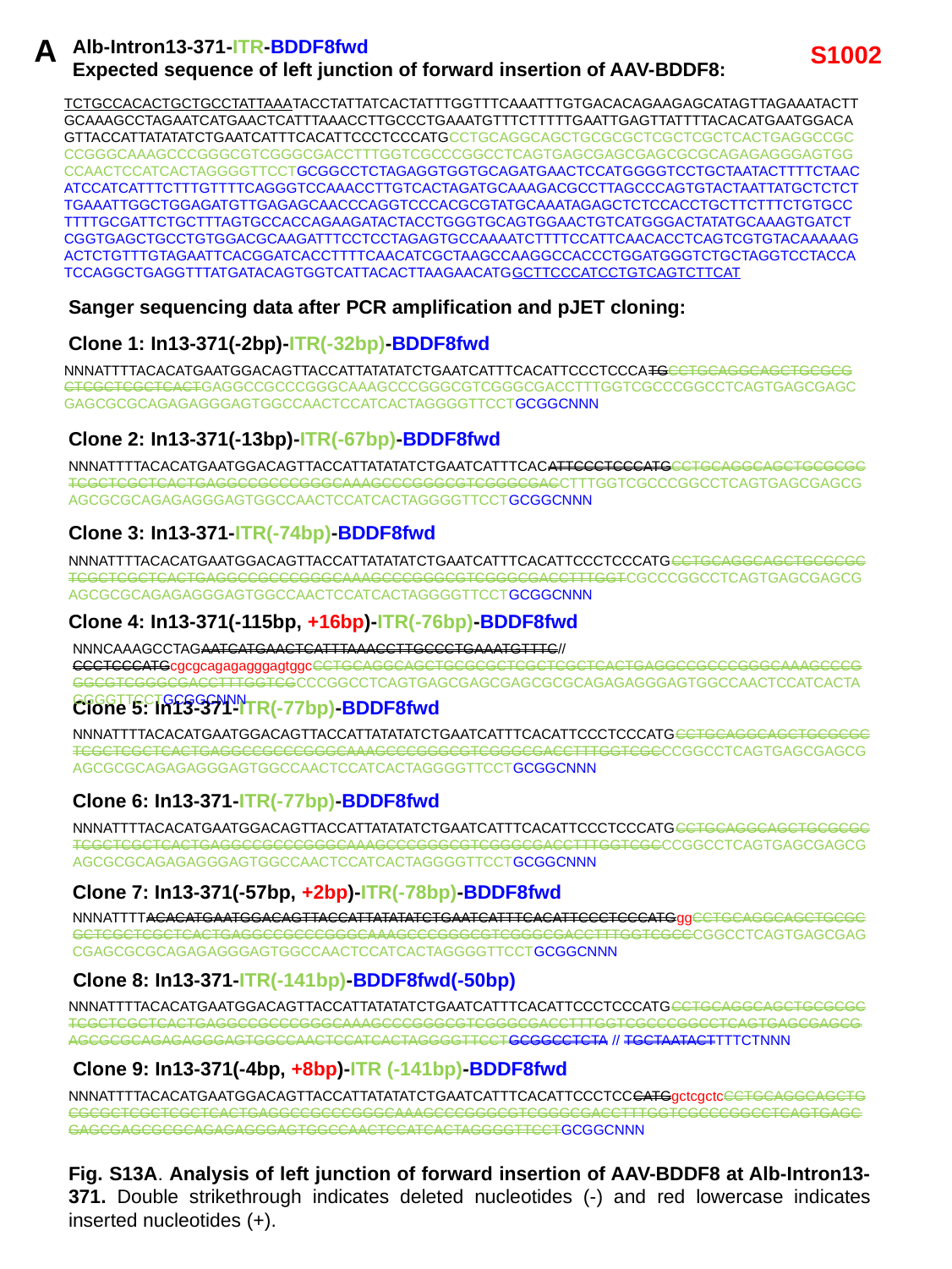

Alb-Intron13-371-ITR-BDDF8fwd
Expected sequence of left junction of forward insertion of AAV-BDDF8:
A
S1002
tctgccacactgctgcctattaaatacctattatcactatttggtttcaaatttgtgacacagaagagcatagttagaaatacttgcaaagcctagaatcatgaactcatttaaaccttgccctgaaatgtttctttttgaattgagttattttacacatgaatggacagttaccattatatatctgaatcatttcacattccctcccatgCCTGCAGGCAGCTGCGCGCTCGCTCGCTCACTGAGGCCGCCCGGGCAAAGCCCGGGCGTCGGGCGACCTTTGGTCGCCCGGCCTCAGTGAGCGAGCGAGCGCGCAGAGAGGGAGTGGCCAACTCCATCACTAGGGGTTCCTGCGGCCTCTAGAGGTGGTGCAGATGAACTCCATGGGGTCCTGCTAATACTTTTCTAACATCCATCATTTCTTTGTTTTCAGGGTCCAAACCTTGTCACTAGATGCAAAGACGCCTTAGCCCAGTGTACTAATTATGCTCTCTTGAAATTGGCTGGAGATGTTGAGAGCAACCCAGGTCCCACGCGTATGCAAATAGAGCTCTCCACCTGCTTCTTTCTGTGCCTTTTGCGATTCTGCTTTAGTGCCACCAGAAGATACTACCTGGGTGCAGTGGAACTGTCATGGGACTATATGCAAAGTGATCTCGGTGAGCTGCCTGTGGACGCAAGATTTCCTCCTAGAGTGCCAAAATCTTTTCCATTCAACACCTCAGTCGTGTACAAAAAGACTCTGTTTGTAGAATTCACGGATCACCTTTTCAACATCGCTAAGCCAAGGCCACCCTGGATGGGTCTGCTAGGTCCTACCATCCAGGCTGAGGTTTATGATACAGTGGTCATTACACTTAAGAACATGGCTTCCCATCCTGTCAGTCTTCAT
Sanger sequencing data after PCR amplification and pJET cloning:
Clone 1: In13-371(-2bp)-ITR(-32bp)-BDDF8fwd
NNNattttacacatgaatggacagttaccattatatatctgaatcatttcacattccctcccatgCCTGCAGGCAGCTGCGCGCTCGCTCGCTCACTGAGGCCGCCCGGGCAAAGCCCGGGCGTCGGGCGACCTTTGGTCGCCCGGCCTCAGTGAGCGAGCGAGCGCGCAGAGAGGGAGTGGCCAACTCCATCACTAGGGGTTCCTGCGGCNNN
Clone 2: In13-371(-13bp)-ITR(-67bp)-BDDF8fwd
NNNattttacacatgaatggacagttaccattatatatctgaatcatttcacattccctcccatgCCTGCAGGCAGCTGCGCGCTCGCTCGCTCACTGAGGCCGCCCGGGCAAAGCCCGGGCGTCGGGCGACCTTTGGTCGCCCGGCCTCAGTGAGCGAGCGAGCGCGCAGAGAGGGAGTGGCCAACTCCATCACTAGGGGTTCCTGCGGCNNN
Clone 3: In13-371-ITR(-74bp)-BDDF8fwd
NNNattttacacatgaatggacagttaccattatatatctgaatcatttcacattccctcccatgCCTGCAGGCAGCTGCGCGCTCGCTCGCTCACTGAGGCCGCCCGGGCAAAGCCCGGGCGTCGGGCGACCTTTGGTCGCCCGGCCTCAGTGAGCGAGCGAGCGCGCAGAGAGGGAGTGGCCAACTCCATCACTAGGGGTTCCTGCGGCNNN
Clone 4: In13-371(-115bp, +16bp)-ITR(-76bp)-BDDF8fwd
NNNcaaagcctagaatcatgaactcatttaaaccttgccctgaaatgtttc//ccctcccatgcgcgcagagagggagtggcCCTGCAGGCAGCTGCGCGCTCGCTCGCTCACTGAGGCCGCCCGGGCAAAGCCCGGGCGTCGGGCGACCTTTGGTCGCCCGGCCTCAGTGAGCGAGCGAGCGCGCAGAGAGGGAGTGGCCAACTCCATCACTAGGGGTTCCTGCGGCNNN
Clone 5: In13-371-ITR(-77bp)-BDDF8fwd
NNNattttacacatgaatggacagttaccattatatatctgaatcatttcacattccctcccatgCCTGCAGGCAGCTGCGCGCTCGCTCGCTCACTGAGGCCGCCCGGGCAAAGCCCGGGCGTCGGGCGACCTTTGGTCGCCCGGCCTCAGTGAGCGAGCGAGCGCGCAGAGAGGGAGTGGCCAACTCCATCACTAGGGGTTCCTGCGGCNNN
Clone 6: In13-371-ITR(-77bp)-BDDF8fwd
NNNattttacacatgaatggacagttaccattatatatctgaatcatttcacattccctcccatgCCTGCAGGCAGCTGCGCGCTCGCTCGCTCACTGAGGCCGCCCGGGCAAAGCCCGGGCGTCGGGCGACCTTTGGTCGCCCGGCCTCAGTGAGCGAGCGAGCGCGCAGAGAGGGAGTGGCCAACTCCATCACTAGGGGTTCCTGCGGCNNN
Clone 7: In13-371(-57bp, +2bp)-ITR(-78bp)-BDDF8fwd
NNNattttacacatgaatggacagttaccattatatatctgaatcatttcacattccctcccatgggCCTGCAGGCAGCTGCGCGCTCGCTCGCTCACTGAGGCCGCCCGGGCAAAGCCCGGGCGTCGGGCGACCTTTGGTCGCCCGGCCTCAGTGAGCGAGCGAGCGCGCAGAGAGGGAGTGGCCAACTCCATCACTAGGGGTTCCTGCGGCNNN
Clone 8: In13-371-ITR(-141bp)-BDDF8fwd(-50bp)
NNNattttacacatgaatggacagttaccattatatatctgaatcatttcacattccctcccatgCCTGCAGGCAGCTGCGCGCTCGCTCGCTCACTGAGGCCGCCCGGGCAAAGCCCGGGCGTCGGGCGACCTTTGGTCGCCCGGCCTCAGTGAGCGAGCGAGCGCGCAGAGAGGGAGTGGCCAACTCCATCACTAGGGGTTCCTGCGGCCTCTA // TGCTAATACTTTTCTNNN
Clone 9: In13-371(-4bp, +8bp)-ITR (-141bp)-BDDF8fwd
NNNattttacacatgaatggacagttaccattatatatctgaatcatttcacattccctcccatggctcgctcCCTGCAGGCAGCTGCGCGCTCGCTCGCTCACTGAGGCCGCCCGGGCAAAGCCCGGGCGTCGGGCGACCTTTGGTCGCCCGGCCTCAGTGAGCGAGCGAGCGCGCAGAGAGGGAGTGGCCAACTCCATCACTAGGGGTTCCTGCGGCNNN
Fig. S13A. Analysis of left junction of forward insertion of AAV-BDDF8 at Alb-Intron13-371. Double strikethrough indicates deleted nucleotides (-) and red lowercase indicates inserted nucleotides (+).

## Slide 25
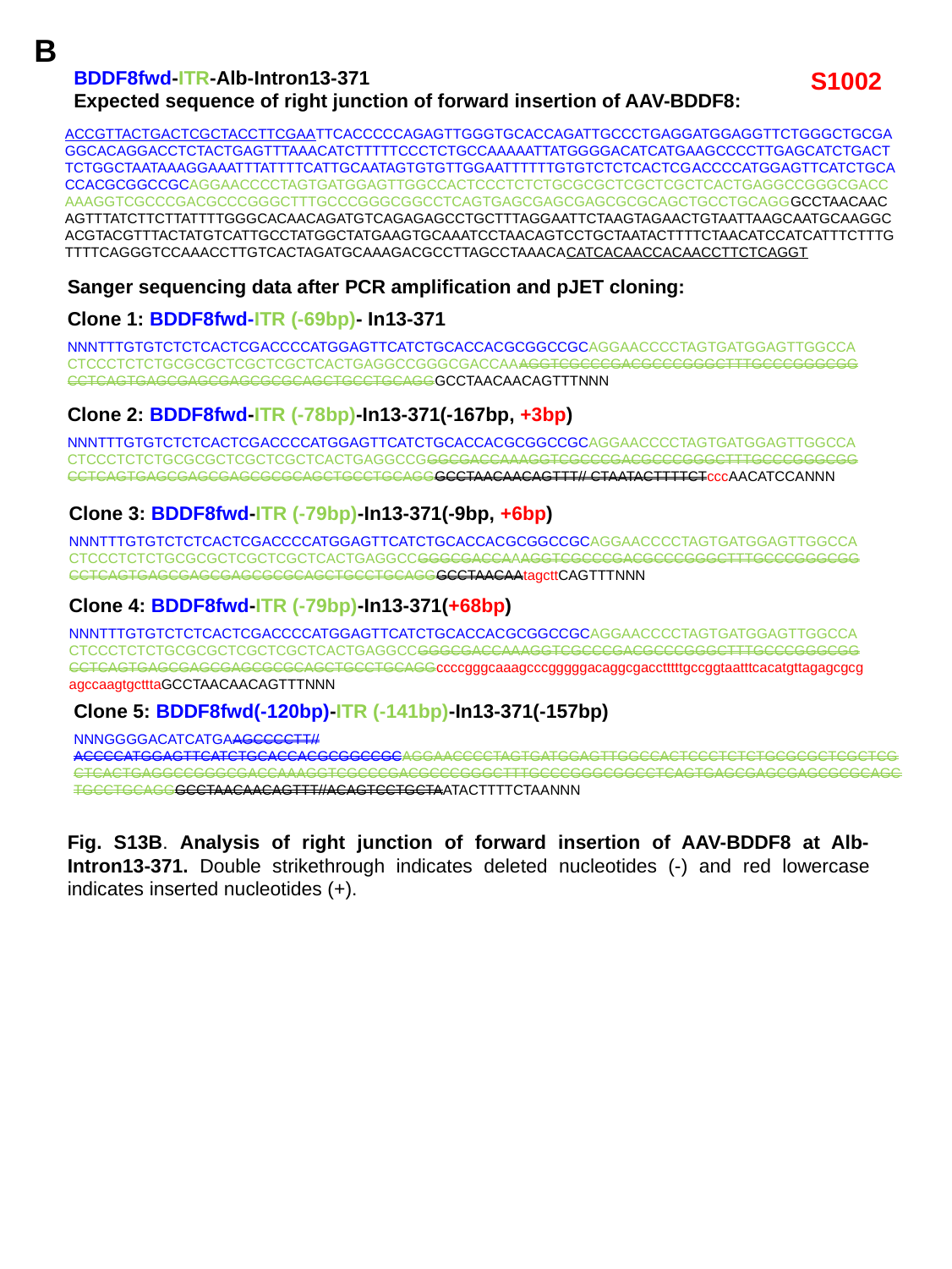

B
BDDF8fwd-ITR-Alb-Intron13-371
Expected sequence of right junction of forward insertion of AAV-BDDF8:
S1002
ACCGTTACTGACTCGCTACCTTCGAATTCACCCCCAGAGTTGGGTGCACCAGATTGCCCTGAGGATGGAGGTTCTGGGCTGCGAGGCACAGGACCTCTACTGAGTTTAAACATCTTTTTCCCTCTGCCAAAAATTATGGGGACATCATGAAGCCCCTTGAGCATCTGACTTCTGGCTAATAAAGGAAATTTATTTTCATTGCAATAGTGTGTTGGAATTTTTTGTGTCTCTCACTCGACCCCATGGAGTTCATCTGCACCACGCGGCCGCAGGAACCCCTAGTGATGGAGTTGGCCACTCCCTCTCTGCGCGCTCGCTCGCTCACTGAGGCCGGGCGACCAAAGGTCGCCCGACGCCCGGGCTTTGCCCGGGCGGCCTCAGTGAGCGAGCGAGCGCGCAGCTGCCTGCAGGgcctaacaacagtttatcttcttattttgggcacaacagatgtcagagagcctgctttaggaattctaagtagaactgtaattaagcaatgcaaggcacgtacgtttactatgtcattgcctatggctatgaagtgcaaatcctaacagtcctgctaatacttttctaacatccatcatttctttgttttcagggtccaaaccttgtcactagatgcaaagacgccttagcctaaacacatcacaaccacaaccttctcaggt
Sanger sequencing data after PCR amplification and pJET cloning:
Clone 1: BDDF8fwd-ITR (-69bp)- In13-371
NNNTTTGTGTCTCTCACTCGACCCCATGGAGTTCATCTGCACCACGCGGCCGCAGGAACCCCTAGTGATGGAGTTGGCCACTCCCTCTCTGCGCGCTCGCTCGCTCACTGAGGCCGGGCGACCAAAGGTCGCCCGACGCCCGGGCTTTGCCCGGGCGGCCTCAGTGAGCGAGCGAGCGCGCAGCTGCCTGCAGGgcctaacaacagtttNNN
Clone 2: BDDF8fwd-ITR (-78bp)-In13-371(-167bp, +3bp)
NNNTTTGTGTCTCTCACTCGACCCCATGGAGTTCATCTGCACCACGCGGCCGCAGGAACCCCTAGTGATGGAGTTGGCCACTCCCTCTCTGCGCGCTCGCTCGCTCACTGAGGCCGGGCGACCAAAGGTCGCCCGACGCCCGGGCTTTGCCCGGGCGGCCTCAGTGAGCGAGCGAGCGCGCAGCTGCCTGCAGGgcctaacaacagttt// ctaatacttttctcccAACATCCANNN
Clone 3: BDDF8fwd-ITR (-79bp)-In13-371(-9bp, +6bp)
NNNTTTGTGTCTCTCACTCGACCCCATGGAGTTCATCTGCACCACGCGGCCGCAGGAACCCCTAGTGATGGAGTTGGCCACTCCCTCTCTGCGCGCTCGCTCGCTCACTGAGGCCGGGCGACCAAAGGTCGCCCGACGCCCGGGCTTTGCCCGGGCGGCCTCAGTGAGCGAGCGAGCGCGCAGCTGCCTGCAGGgcctaacaatagcttcagtttNNN
Clone 4: BDDF8fwd-ITR (-79bp)-In13-371(+68bp)
NNNTTTGTGTCTCTCACTCGACCCCATGGAGTTCATCTGCACCACGCGGCCGCAGGAACCCCTAGTGATGGAGTTGGCCACTCCCTCTCTGCGCGCTCGCTCGCTCACTGAGGCCGGGCGACCAAAGGTCGCCCGACGCCCGGGCTTTGCCCGGGCGGCCTCAGTGAGCGAGCGAGCGCGCAGCTGCCTGCAGGccccgggcaaagcccgggggacaggcgacctttttgccggtaatttcacatgttagagcgcgagccaagtgctttagcctaacaacagtttNNN
Clone 5: BDDF8fwd(-120bp)-ITR (-141bp)-In13-371(-157bp)
NNNGGGGACATCATGAAGCCCCTT//ACCCCATGGAGTTCATCTGCACCACGCGGCCGCAGGAACCCCTAGTGATGGAGTTGGCCACTCCCTCTCTGCGCGCTCGCTCGCTCACTGAGGCCGGGCGACCAAAGGTCGCCCGACGCCCGGGCTTTGCCCGGGCGGCCTCAGTGAGCGAGCGAGCGCGCAGCTGCCTGCAGGgcctaacaacagttt//ACAGTCCTGCTAATACTTTTCTAANNN
Fig. S13B. Analysis of right junction of forward insertion of AAV-BDDF8 at Alb-Intron13-371. Double strikethrough indicates deleted nucleotides (-) and red lowercase indicates inserted nucleotides (+).

## Slide 26
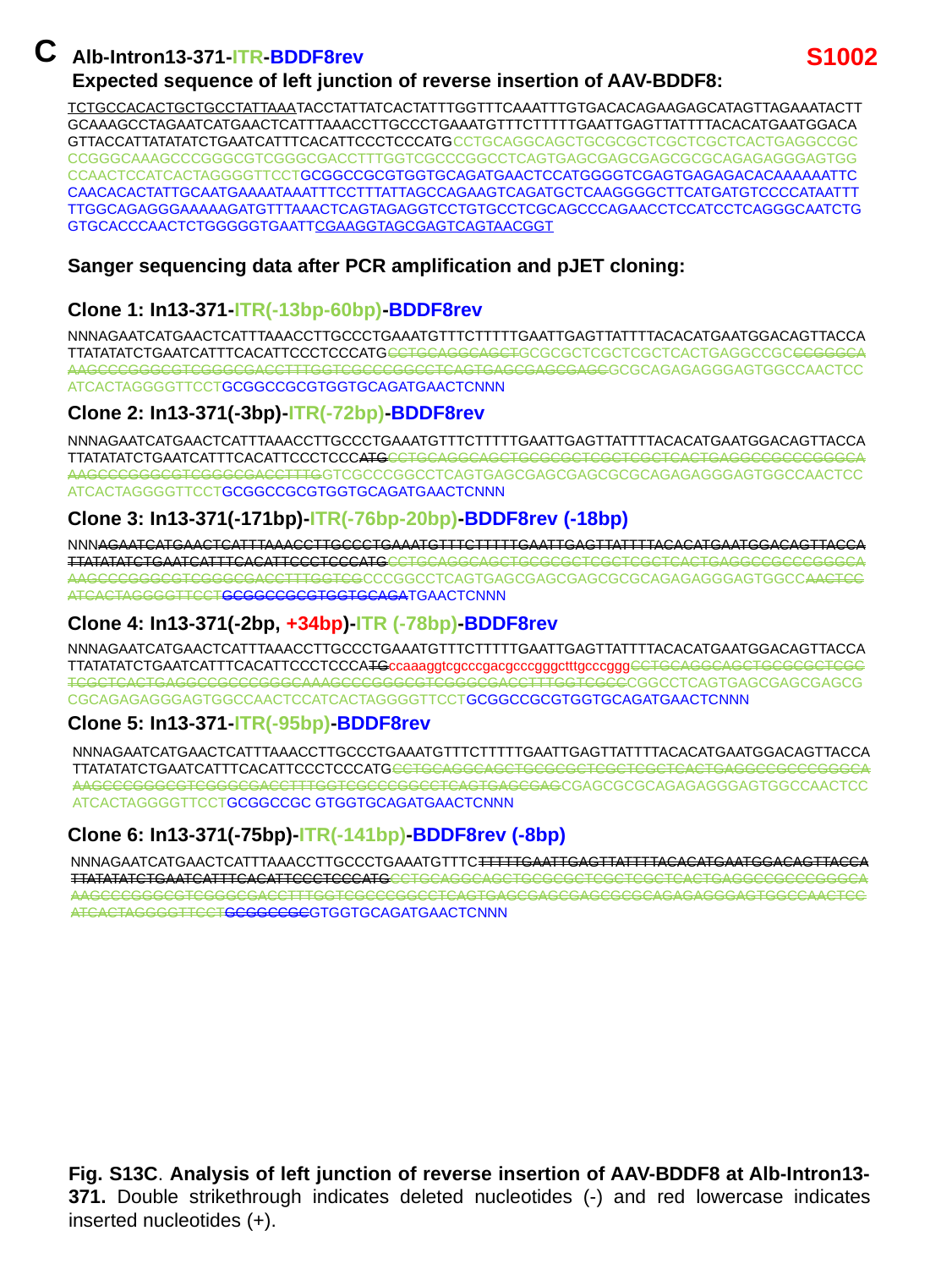

C
S1002
Alb-Intron13-371-ITR-BDDF8rev
Expected sequence of left junction of reverse insertion of AAV-BDDF8:
TCTGCCACACTGCTGCCTATTAAATACCTATTATCACTATTTGGTTTCAAATTTGTGACACAGAAGAGCATAGTTAGAAATACTTGCAAAGCCTAGAATCATGAACTCATTTAAACCTTGCCCTGAAATGTTTCTTTTTGAATTGAGTTATTTTACACATGAATGGACAGTTACCATTATATATCTGAATCATTTCACATTCCCTCCCATGCCTGCAGGCAGCTGCGCGCTCGCTCGCTCACTGAGGCCGCCCGGGCAAAGCCCGGGCGTCGGGCGACCTTTGGTCGCCCGGCCTCAGTGAGCGAGCGAGCGCGCAGAGAGGGAGTGGCCAACTCCATCACTAGGGGTTCCTGCGGCCGCGTGGTGCAGATGAACTCCATGGGGTCGAGTGAGAGACACAAAAAATTCCAACACACTATTGCAATGAAAATAAATTTCCTTTATTAGCCAGAAGTCAGATGCTCAAGGGGCTTCATGATGTCCCCATAATTTTTGGCAGAGGGAAAAAGATGTTTAAACTCAGTAGAGGTCCTGTGCCTCGCAGCCCAGAACCTCCATCCTCAGGGCAATCTGGTGCACCCAACTCTGGGGGTGAATTCGAAGGTAGCGAGTCAGTAACGGT
Sanger sequencing data after PCR amplification and pJET cloning:
Clone 1: In13-371-ITR(-13bp-60bp)-BDDF8rev
NNNAGAATCATGAACTCATTTAAACCTTGCCCTGAAATGTTTCTTTTTGAATTGAGTTATTTTACACATGAATGGACAGTTACCATTATATATCTGAATCATTTCACATTCCCTCCCATGCCTGCAGGCAGCTGCGCGCTCGCTCGCTCACTGAGGCCGCCCGGGCAAAGCCCGGGCGTCGGGCGACCTTTGGTCGCCCGGCCTCAGTGAGCGAGCGAGCGCGCAGAGAGGGAGTGGCCAACTCCATCACTAGGGGTTCCTGCGGCCGCGTGGTGCAGATGAACTCNNN
Clone 2: In13-371(-3bp)-ITR(-72bp)-BDDF8rev
NNNAGAATCATGAACTCATTTAAACCTTGCCCTGAAATGTTTCTTTTTGAATTGAGTTATTTTACACATGAATGGACAGTTACCATTATATATCTGAATCATTTCACATTCCCTCCCATGCCTGCAGGCAGCTGCGCGCTCGCTCGCTCACTGAGGCCGCCCGGGCAAAGCCCGGGCGTCGGGCGACCTTTGGTCGCCCGGCCTCAGTGAGCGAGCGAGCGCGCAGAGAGGGAGTGGCCAACTCCATCACTAGGGGTTCCTGCGGCCGCGTGGTGCAGATGAACTCNNN
Clone 3: In13-371(-171bp)-ITR(-76bp-20bp)-BDDF8rev (-18bp)
NNNAGAATCATGAACTCATTTAAACCTTGCCCTGAAATGTTTCTTTTTGAATTGAGTTATTTTACACATGAATGGACAGTTACCATTATATATCTGAATCATTTCACATTCCCTCCCATGCCTGCAGGCAGCTGCGCGCTCGCTCGCTCACTGAGGCCGCCCGGGCAAAGCCCGGGCGTCGGGCGACCTTTGGTCGCCCGGCCTCAGTGAGCGAGCGAGCGCGCAGAGAGGGAGTGGCCAACTCCATCACTAGGGGTTCCTGCGGCCGCGTGGTGCAGATGAACTCNNN
Clone 4: In13-371(-2bp, +34bp)-ITR (-78bp)-BDDF8rev
NNNAGAATCATGAACTCATTTAAACCTTGCCCTGAAATGTTTCTTTTTGAATTGAGTTATTTTACACATGAATGGACAGTTACCATTATATATCTGAATCATTTCACATTCCCTCCCATGccaaaggtcgcccgacgcccgggctttgcccgggCCTGCAGGCAGCTGCGCGCTCGCTCGCTCACTGAGGCCGCCCGGGCAAAGCCCGGGCGTCGGGCGACCTTTGGTCGCCCGGCCTCAGTGAGCGAGCGAGCGCGCAGAGAGGGAGTGGCCAACTCCATCACTAGGGGTTCCTGCGGCCGCGTGGTGCAGATGAACTCNNN
Clone 5: In13-371-ITR(-95bp)-BDDF8rev
NNNAGAATCATGAACTCATTTAAACCTTGCCCTGAAATGTTTCTTTTTGAATTGAGTTATTTTACACATGAATGGACAGTTACCATTATATATCTGAATCATTTCACATTCCCTCCCATGCCTGCAGGCAGCTGCGCGCTCGCTCGCTCACTGAGGCCGCCCGGGCAAAGCCCGGGCGTCGGGCGACCTTTGGTCGCCCGGCCTCAGTGAGCGAGCGAGCGCGCAGAGAGGGAGTGGCCAACTCCATCACTAGGGGTTCCTGCGGCCGC GTGGTGCAGATGAACTCNNN
Clone 6: In13-371(-75bp)-ITR(-141bp)-BDDF8rev (-8bp)
NNNAGAATCATGAACTCATTTAAACCTTGCCCTGAAATGTTTCTTTTTGAATTGAGTTATTTTACACATGAATGGACAGTTACCATTATATATCTGAATCATTTCACATTCCCTCCCATGCCTGCAGGCAGCTGCGCGCTCGCTCGCTCACTGAGGCCGCCCGGGCAAAGCCCGGGCGTCGGGCGACCTTTGGTCGCCCGGCCTCAGTGAGCGAGCGAGCGCGCAGAGAGGGAGTGGCCAACTCCATCACTAGGGGTTCCTGCGGCCGCGTGGTGCAGATGAACTCNNN
Fig. S13C. Analysis of left junction of reverse insertion of AAV-BDDF8 at Alb-Intron13-371. Double strikethrough indicates deleted nucleotides (-) and red lowercase indicates inserted nucleotides (+).

## Slide 27
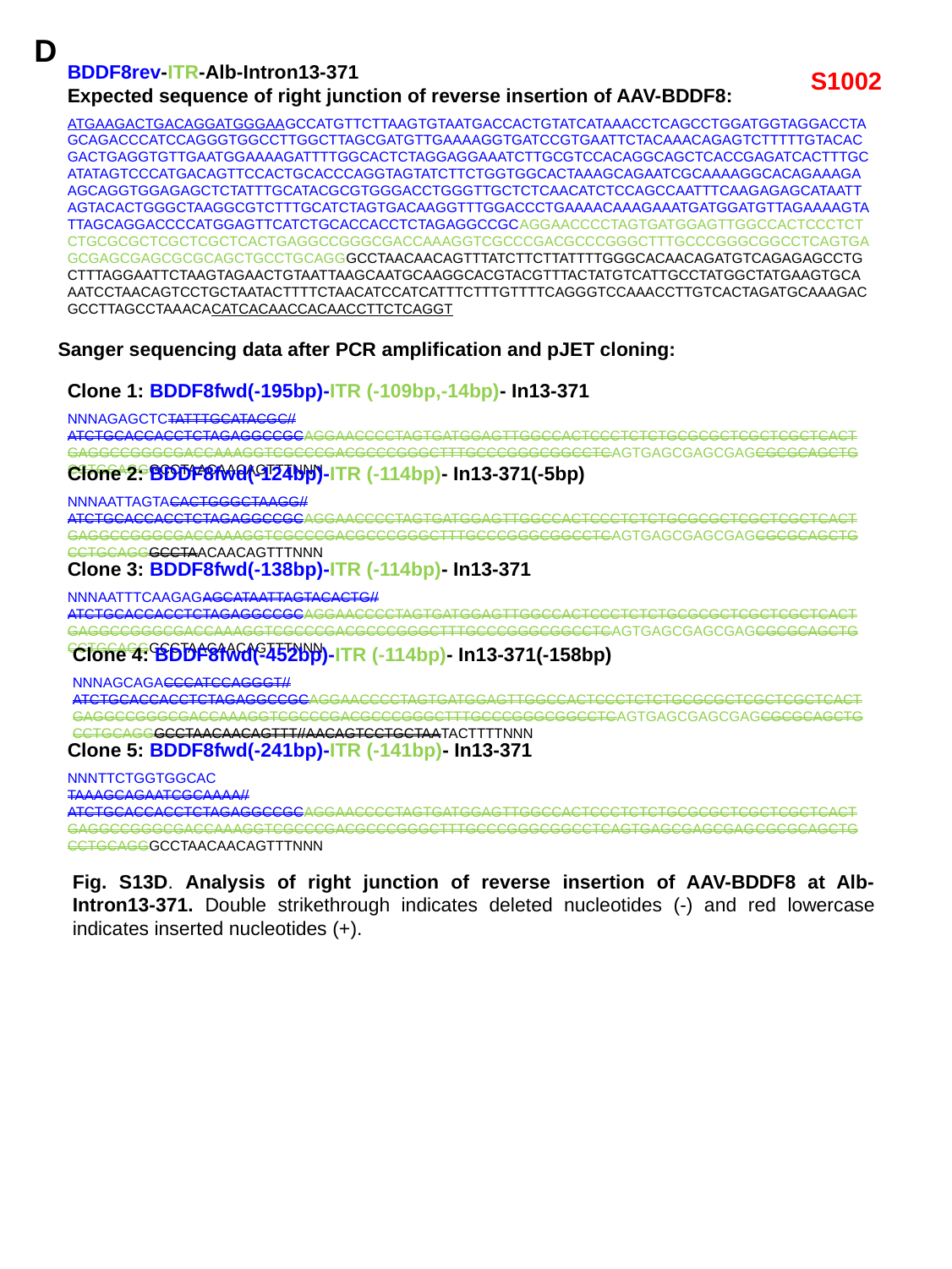

D
BDDF8rev-ITR-Alb-Intron13-371
Expected sequence of right junction of reverse insertion of AAV-BDDF8:
S1002
ATGAAGACTGACAGGATGGGAAGCCATGTTCTTAAGTGTAATGACCACTGTATCATAAACCTCAGCCTGGATGGTAGGACCTAGCAGACCCATCCAGGGTGGCCTTGGCTTAGCGATGTTGAAAAGGTGATCCGTGAATTCTACAAACAGAGTCTTTTTGTACACGACTGAGGTGTTGAATGGAAAAGATTTTGGCACTCTAGGAGGAAATCTTGCGTCCACAGGCAGCTCACCGAGATCACTTTGCATATAGTCCCATGACAGTTCCACTGCACCCAGGTAGTATCTTCTGGTGGCACTAAAGCAGAATCGCAAAAGGCACAGAAAGAAGCAGGTGGAGAGCTCTATTTGCATACGCGTGGGACCTGGGTTGCTCTCAACATCTCCAGCCAATTTCAAGAGAGCATAATTAGTACACTGGGCTAAGGCGTCTTTGCATCTAGTGACAAGGTTTGGACCCTGAAAACAAAGAAATGATGGATGTTAGAAAAGTATTAGCAGGACCCCATGGAGTTCATCTGCACCACCTCTAGAGGCCGCAGGAACCCCTAGTGATGGAGTTGGCCACTCCCTCTCTGCGCGCTCGCTCGCTCACTGAGGCCGGGCGACCAAAGGTCGCCCGACGCCCGGGCTTTGCCCGGGCGGCCTCAGTGAGCGAGCGAGCGCGCAGCTGCCTGCAGGgcctaacaacagtttatcttcttattttgggcacaacagatgtcagagagcctgctttaggaattctaagtagaactgtaattaagcaatgcaaggcacgtacgtttactatgtcattgcctatggctatgaagtgcaaatcctaacagtcctgctaatacttttctaacatccatcatttctttgttttcagggtccaaaccttgtcactagatgcaaagacgccttagcctaaacacatcacaaccacaaccttctcaggt
Sanger sequencing data after PCR amplification and pJET cloning:
Clone 1: BDDF8fwd(-195bp)-ITR (-109bp,-14bp)- In13-371
NNNAGAGCTCTATTTGCATACGC//ATCTGCACCACCTCTAGAGGCCGCAGGAACCCCTAGTGATGGAGTTGGCCACTCCCTCTCTGCGCGCTCGCTCGCTCACTGAGGCCGGGCGACCAAAGGTCGCCCGACGCCCGGGCTTTGCCCGGGCGGCCTCAGTGAGCGAGCGAGCGCGCAGCTGCCTGCAGGgcctaacaacagtttNNN
Clone 2: BDDF8fwd(-124bp)-ITR (-114bp)- In13-371(-5bp)
NNNAATTAGTACACTGGGCTAAGG//ATCTGCACCACCTCTAGAGGCCGCAGGAACCCCTAGTGATGGAGTTGGCCACTCCCTCTCTGCGCGCTCGCTCGCTCACTGAGGCCGGGCGACCAAAGGTCGCCCGACGCCCGGGCTTTGCCCGGGCGGCCTCAGTGAGCGAGCGAGCGCGCAGCTGCCTGCAGGgcctaacaacagtttNNN
Clone 3: BDDF8fwd(-138bp)-ITR (-114bp)- In13-371
NNNAATTTCAAGAGAGCATAATTAGTACACTG//ATCTGCACCACCTCTAGAGGCCGCAGGAACCCCTAGTGATGGAGTTGGCCACTCCCTCTCTGCGCGCTCGCTCGCTCACTGAGGCCGGGCGACCAAAGGTCGCCCGACGCCCGGGCTTTGCCCGGGCGGCCTCAGTGAGCGAGCGAGCGCGCAGCTGCCTGCAGGgcctaacaacagtttNNN
Clone 4: BDDF8fwd(-452bp)-ITR (-114bp)- In13-371(-158bp)
NNNAGCAGACCCATCCAGGGT//ATCTGCACCACCTCTAGAGGCCGCAGGAACCCCTAGTGATGGAGTTGGCCACTCCCTCTCTGCGCGCTCGCTCGCTCACTGAGGCCGGGCGACCAAAGGTCGCCCGACGCCCGGGCTTTGCCCGGGCGGCCTCAGTGAGCGAGCGAGCGCGCAGCTGCCTGCAGGgcctaacaacagttt//AACAGTCCTGCTAAtacttttNNN
Clone 5: BDDF8fwd(-241bp)-ITR (-141bp)- In13-371
NNNTTCTGGTGGCAC
TAAAGCAGAATCGCAAAA//ATCTGCACCACCTCTAGAGGCCGCAGGAACCCCTAGTGATGGAGTTGGCCACTCCCTCTCTGCGCGCTCGCTCGCTCACTGAGGCCGGGCGACCAAAGGTCGCCCGACGCCCGGGCTTTGCCCGGGCGGCCTCAGTGAGCGAGCGAGCGCGCAGCTGCCTGCAGGgcctaacaacagtttNNN
Fig. S13D. Analysis of right junction of reverse insertion of AAV-BDDF8 at Alb-Intron13-371. Double strikethrough indicates deleted nucleotides (-) and red lowercase indicates inserted nucleotides (+).

## Slide 28
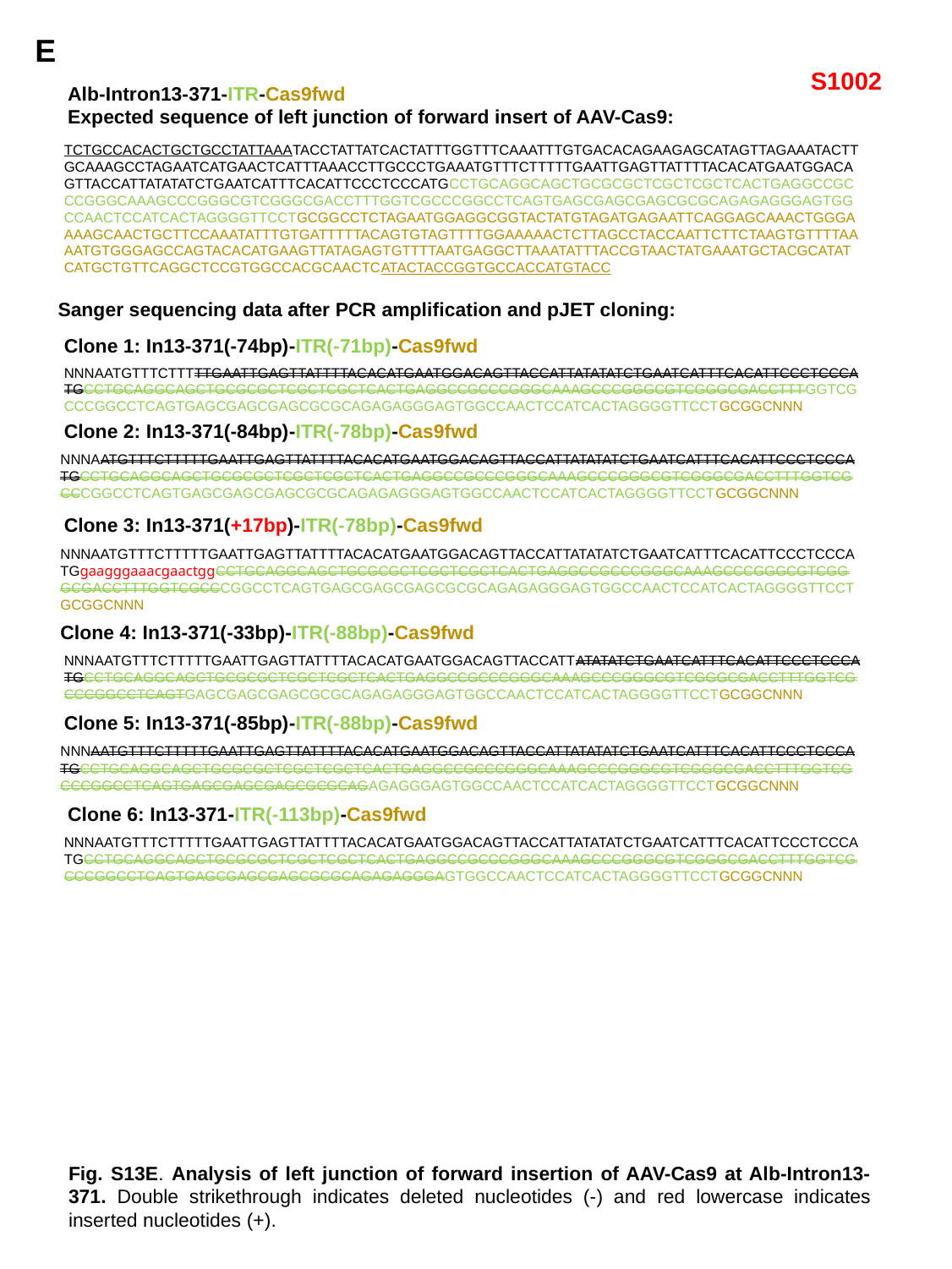

E
Alb-Intron13-371-ITR-Cas9fwd
Expected sequence of left junction of forward insert of AAV-Cas9:
S1002
tctgccacactgctgcctattaaatacctattatcactatttggtttcaaatttgtgacacagaagagcatagttagaaatacttgcaaagcctagaatcatgaactcatttaaaccttgccctgaaatgtttctttttgaattgagttattttacacatgaatggacagttaccattatatatctgaatcatttcacattccctcccatgCCTGCAGGCAGCTGCGCGCTCGCTCGCTCACTGAGGCCGCCCGGGCAAAGCCCGGGCGTCGGGCGACCTTTGGTCGCCCGGCCTCAGTGAGCGAGCGAGCGCGCAGAGAGGGAGTGGCCAACTCCATCACTAGGGGTTCCTGCGGCCTCTAGAATGGAGGCGGTACTATGTAGATGAGAATTCAGGAGCAAACTGGGAAAAGCAACTGCTTCCAAATATTTGTGATTTTTACAGTGTAGTTTTGGAAAAACTCTTAGCCTACCAATTCTTCTAAGTGTTTTAAAATGTGGGAGCCAGTACACATGAAGTTATAGAGTGTTTTAATGAGGCTTAAATATTTACCGTAACTATGAAATGCTACGCATATCATGCTGTTCAGGCTCCGTGGCCACGCAACTCATACTACCGGTGCCACCATGTACC
Sanger sequencing data after PCR amplification and pJET cloning:
Clone 1: In13-371(-74bp)-ITR(-71bp)-Cas9fwd
NNNAatgtttctttttgaattgagttattttacacatgaatggacagttaccattatatatctgaatcatttcacattccctcccatgCCTGCAGGCAGCTGCGCGCTCGCTCGCTCACTGAGGCCGCCCGGGCAAAGCCCGGGCGTCGGGCGACCTTTGGTCGCCCGGCCTCAGTGAGCGAGCGAGCGCGCAGAGAGGGAGTGGCCAACTCCATCACTAGGGGTTCCTGCGGCNNN
Clone 2: In13-371(-84bp)-ITR(-78bp)-Cas9fwd
NNNAatgtttctttttgaattgagttattttacacatgaatggacagttaccattatatatctgaatcatttcacattccctcccatgCCTGCAGGCAGCTGCGCGCTCGCTCGCTCACTGAGGCCGCCCGGGCAAAGCCCGGGCGTCGGGCGACCTTTGGTCGCCCGGCCTCAGTGAGCGAGCGAGCGCGCAGAGAGGGAGTGGCCAACTCCATCACTAGGGGTTCCTGCGGCNNN
Clone 3: In13-371(+17bp)-ITR(-78bp)-Cas9fwd
NNNAatgtttctttttgaattgagttattttacacatgaatggacagttaccattatatatctgaatcatttcacattccctcccatggaagggaaacgaactggCCTGCAGGCAGCTGCGCGCTCGCTCGCTCACTGAGGCCGCCCGGGCAAAGCCCGGGCGTCGGGCGACCTTTGGTCGCCCGGCCTCAGTGAGCGAGCGAGCGCGCAGAGAGGGAGTGGCCAACTCCATCACTAGGGGTTCCTGCGGCNNN
Clone 4: In13-371(-33bp)-ITR(-88bp)-Cas9fwd
NNNAatgtttctttttgaattgagttattttacacatgaatggacagttaccattatatatctgaatcatttcacattccctcccatgCCTGCAGGCAGCTGCGCGCTCGCTCGCTCACTGAGGCCGCCCGGGCAAAGCCCGGGCGTCGGGCGACCTTTGGTCGCCCGGCCTCAGTGAGCGAGCGAGCGCGCAGAGAGGGAGTGGCCAACTCCATCACTAGGGGTTCCTGCGGCNNN
Clone 5: In13-371(-85bp)-ITR(-88bp)-Cas9fwd
NNNAatgtttctttttgaattgagttattttacacatgaatggacagttaccattatatatctgaatcatttcacattccctcccatgCCTGCAGGCAGCTGCGCGCTCGCTCGCTCACTGAGGCCGCCCGGGCAAAGCCCGGGCGTCGGGCGACCTTTGGTCGCCCGGCCTCAGTGAGCGAGCGAGCGCGCAGAGAGGGAGTGGCCAACTCCATCACTAGGGGTTCCTGCGGCNNN
Clone 6: In13-371-ITR(-113bp)-Cas9fwd
NNNAatgtttctttttgaattgagttattttacacatgaatggacagttaccattatatatctgaatcatttcacattccctcccatgCCTGCAGGCAGCTGCGCGCTCGCTCGCTCACTGAGGCCGCCCGGGCAAAGCCCGGGCGTCGGGCGACCTTTGGTCGCCCGGCCTCAGTGAGCGAGCGAGCGCGCAGAGAGGGAGTGGCCAACTCCATCACTAGGGGTTCCTGCGGCNNN
Fig. S13E. Analysis of left junction of forward insertion of AAV-Cas9 at Alb-Intron13-371. Double strikethrough indicates deleted nucleotides (-) and red lowercase indicates inserted nucleotides (+).

## Slide 29
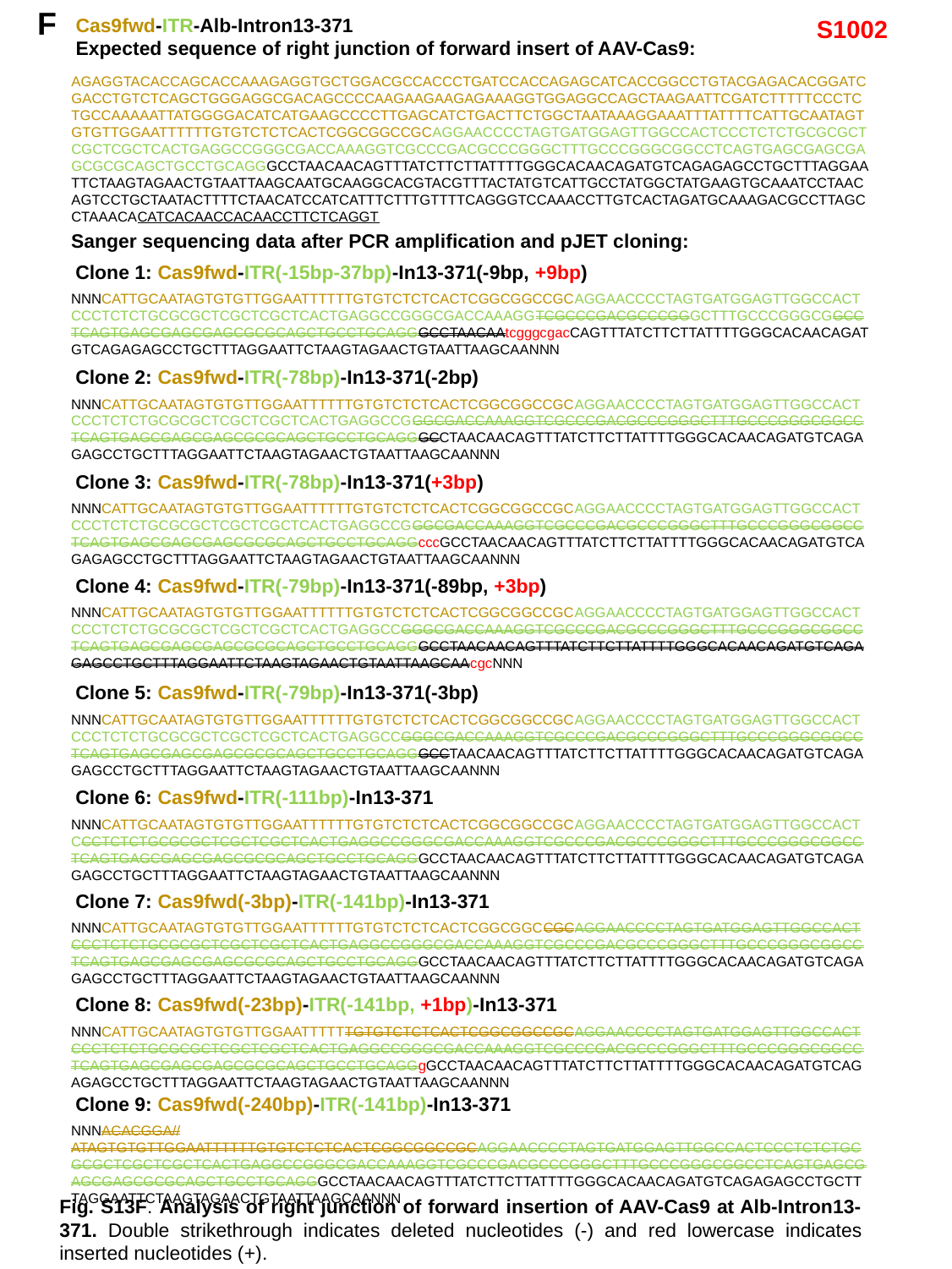

F
Cas9fwd-ITR-Alb-Intron13-371
Expected sequence of right junction of forward insert of AAV-Cas9:
S1002
AGAGGTACACCAGCACCAAAGAGGTGCTGGACGCCACCCTGATCCACCAGAGCATCACCGGCCTGTACGAGACACGGATCGACCTGTCTCAGCTGGGAGGCGACAGCCCCAAGAAGAAGAGAAAGGTGGAGGCCAGCTAAGAATTCGATCTTTTTCCCTCTGCCAAAAATTATGGGGACATCATGAAGCCCCTTGAGCATCTGACTTCTGGCTAATAAAGGAAATTTATTTTCATTGCAATAGTGTGTTGGAATTTTTTGTGTCTCTCACTCGGCGGCCGCAGGAACCCCTAGTGATGGAGTTGGCCACTCCCTCTCTGCGCGCTCGCTCGCTCACTGAGGCCGGGCGACCAAAGGTCGCCCGACGCCCGGGCTTTGCCCGGGCGGCCTCAGTGAGCGAGCGAGCGCGCAGCTGCCTGCAGGgcctaacaacagtttatcttcttattttgggcacaacagatgtcagagagcctgctttaggaattctaagtagaactgtaattaagcaatgcaaggcacgtacgtttactatgtcattgcctatggctatgaagtgcaaatcctaacagtcctgctaatacttttctaacatccatcatttctttgttttcagggtccaaaccttgtcactagatgcaaagacgccttagcctaaacacatcacaaccacaaccttctcaggt
Sanger sequencing data after PCR amplification and pJET cloning:
Clone 1: Cas9fwd-ITR(-15bp-37bp)-In13-371(-9bp, +9bp)
NNNCATTGCAATAGTGTGTTGGAATTTTTTGTGTCTCTCACTCGGCGGCCGCAGGAACCCCTAGTGATGGAGTTGGCCACTCCCTCTCTGCGCGCTCGCTCGCTCACTGAGGCCGGGCGACCAAAGGTCGCCCGACGCCCGGGCTTTGCCCGGGCGGCCTCAGTGAGCGAGCGAGCGCGCAGCTGCCTGCAGGgcctaacaatcgggcgaccagtttatcttcttattttgggcacaacagatgtcagagagcctgctttaggaattctaagtagaactgtaattaagcaaNNN
Clone 2: Cas9fwd-ITR(-78bp)-In13-371(-2bp)
NNNCATTGCAATAGTGTGTTGGAATTTTTTGTGTCTCTCACTCGGCGGCCGCAGGAACCCCTAGTGATGGAGTTGGCCACTCCCTCTCTGCGCGCTCGCTCGCTCACTGAGGCCGGGCGACCAAAGGTCGCCCGACGCCCGGGCTTTGCCCGGGCGGCCTCAGTGAGCGAGCGAGCGCGCAGCTGCCTGCAGGgcctaacaacagtttatcttcttattttgggcacaacagatgtcagagagcctgctttaggaattctaagtagaactgtaattaagcaaNNN
Clone 3: Cas9fwd-ITR(-78bp)-In13-371(+3bp)
NNNCATTGCAATAGTGTGTTGGAATTTTTTGTGTCTCTCACTCGGCGGCCGCAGGAACCCCTAGTGATGGAGTTGGCCACTCCCTCTCTGCGCGCTCGCTCGCTCACTGAGGCCGGGCGACCAAAGGTCGCCCGACGCCCGGGCTTTGCCCGGGCGGCCTCAGTGAGCGAGCGAGCGCGCAGCTGCCTGCAGGcccgcctaacaacagtttatcttcttattttgggcacaacagatgtcagagagcctgctttaggaattctaagtagaactgtaattaagcaaNNN
Clone 4: Cas9fwd-ITR(-79bp)-In13-371(-89bp, +3bp)
NNNCATTGCAATAGTGTGTTGGAATTTTTTGTGTCTCTCACTCGGCGGCCGCAGGAACCCCTAGTGATGGAGTTGGCCACTCCCTCTCTGCGCGCTCGCTCGCTCACTGAGGCCGGGCGACCAAAGGTCGCCCGACGCCCGGGCTTTGCCCGGGCGGCCTCAGTGAGCGAGCGAGCGCGCAGCTGCCTGCAGGgcctaacaacagtttatcttcttattttgggcacaacagatgtcagagagcctgctttaggaattctaagtagaactgtaattaagcaacgcNNN
Clone 5: Cas9fwd-ITR(-79bp)-In13-371(-3bp)
NNNCATTGCAATAGTGTGTTGGAATTTTTTGTGTCTCTCACTCGGCGGCCGCAGGAACCCCTAGTGATGGAGTTGGCCACTCCCTCTCTGCGCGCTCGCTCGCTCACTGAGGCCGGGCGACCAAAGGTCGCCCGACGCCCGGGCTTTGCCCGGGCGGCCTCAGTGAGCGAGCGAGCGCGCAGCTGCCTGCAGGgcctaacaacagtttatcttcttattttgggcacaacagatgtcagagagcctgctttaggaattctaagtagaactgtaattaagcaaNNN
Clone 6: Cas9fwd-ITR(-111bp)-In13-371
NNNCATTGCAATAGTGTGTTGGAATTTTTTGTGTCTCTCACTCGGCGGCCGCAGGAACCCCTAGTGATGGAGTTGGCCACTCCCTCTCTGCGCGCTCGCTCGCTCACTGAGGCCGGGCGACCAAAGGTCGCCCGACGCCCGGGCTTTGCCCGGGCGGCCTCAGTGAGCGAGCGAGCGCGCAGCTGCCTGCAGGgcctaacaacagtttatcttcttattttgggcacaacagatgtcagagagcctgctttaggaattctaagtagaactgtaattaagcaaNNN
Clone 7: Cas9fwd(-3bp)-ITR(-141bp)-In13-371
NNNCATTGCAATAGTGTGTTGGAATTTTTTGTGTCTCTCACTCGGCGGCCGCAGGAACCCCTAGTGATGGAGTTGGCCACTCCCTCTCTGCGCGCTCGCTCGCTCACTGAGGCCGGGCGACCAAAGGTCGCCCGACGCCCGGGCTTTGCCCGGGCGGCCTCAGTGAGCGAGCGAGCGCGCAGCTGCCTGCAGGgcctaacaacagtttatcttcttattttgggcacaacagatgtcagagagcctgctttaggaattctaagtagaactgtaattaagcaaNNN
Clone 8: Cas9fwd(-23bp)-ITR(-141bp, +1bp)-In13-371
NNNCATTGCAATAGTGTGTTGGAATTTTTTGTGTCTCTCACTCGGCGGCCGCAGGAACCCCTAGTGATGGAGTTGGCCACTCCCTCTCTGCGCGCTCGCTCGCTCACTGAGGCCGGGCGACCAAAGGTCGCCCGACGCCCGGGCTTTGCCCGGGCGGCCTCAGTGAGCGAGCGAGCGCGCAGCTGCCTGCAGGggcctaacaacagtttatcttcttattttgggcacaacagatgtcagagagcctgctttaggaattctaagtagaactgtaattaagcaaNNN
Clone 9: Cas9fwd(-240bp)-ITR(-141bp)-In13-371
NNNACACGGA//ATAGTGTGTTGGAATTTTTTGTGTCTCTCACTCGGCGGCCGCAGGAACCCCTAGTGATGGAGTTGGCCACTCCCTCTCTGCGCGCTCGCTCGCTCACTGAGGCCGGGCGACCAAAGGTCGCCCGACGCCCGGGCTTTGCCCGGGCGGCCTCAGTGAGCGAGCGAGCGCGCAGCTGCCTGCAGGgcctaacaacagtttatcttcttattttgggcacaacagatgtcagagagcctgctttaggaattctaagtagaactgtaattaagcaaNNN
Fig. S13F. Analysis of right junction of forward insertion of AAV-Cas9 at Alb-Intron13-371. Double strikethrough indicates deleted nucleotides (-) and red lowercase indicates inserted nucleotides (+).

## Slide 30
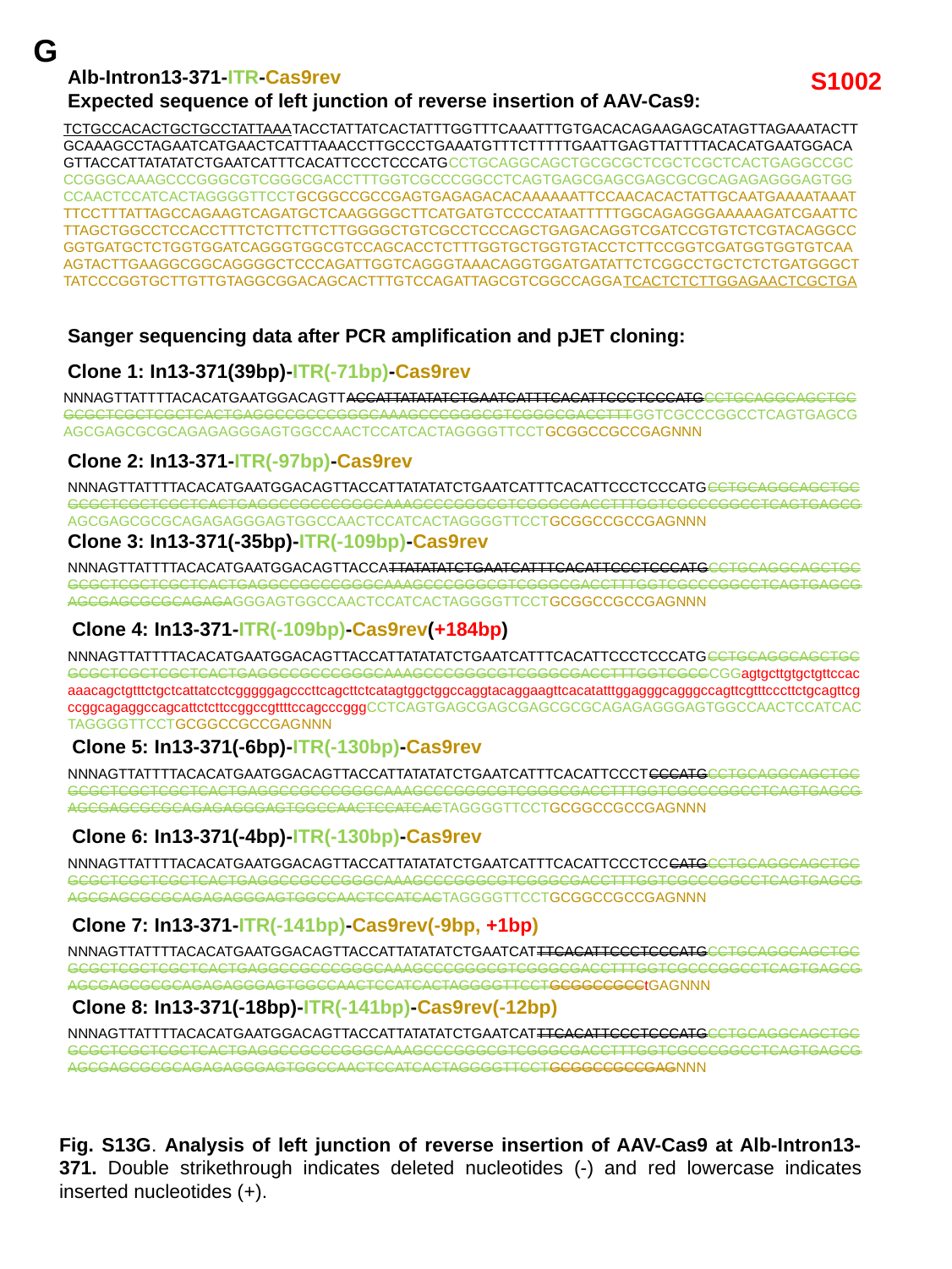

G
Alb-Intron13-371-ITR-Cas9rev
Expected sequence of left junction of reverse insertion of AAV-Cas9:
S1002
tctgccacactgctgcctattaaatacctattatcactatttggtttcaaatttgtgacacagaagagcatagttagaaatacttgcaaagcctagaatcatgaactcatttaaaccttgccctgaaatgtttctttttgaattgagttattttacacatgaatggacagttaccattatatatctgaatcatttcacattccctcccatgCCTGCAGGCAGCTGCGCGCTCGCTCGCTCACTGAGGCCGCCCGGGCAAAGCCCGGGCGTCGGGCGACCTTTGGTCGCCCGGCCTCAGTGAGCGAGCGAGCGCGCAGAGAGGGAGTGGCCAACTCCATCACTAGGGGTTCCTGCGGCCGCCGAGTGAGAGACACAAAAAATTCCAACACACTATTGCAATGAAAATAAATTTCCTTTATTAGCCAGAAGTCAGATGCTCAAGGGGCTTCATGATGTCCCCATAATTTTTGGCAGAGGGAAAAAGATCGAATTCTTAGCTGGCCTCCACCTTTCTCTTCTTCTTGGGGCTGTCGCCTCCCAGCTGAGACAGGTCGATCCGTGTCTCGTACAGGCCGGTGATGCTCTGGTGGATCAGGGTGGCGTCCAGCACCTCTTTGGTGCTGGTGTACCTCTTCCGGTCGATGGTGGTGTCAAAGTACTTGAAGGCGGCAGGGGCTCCCAGATTGGTCAGGGTAAACAGGTGGATGATATTCTCGGCCTGCTCTCTGATGGGCTTATCCCGGTGCTTGTTGTAGGCGGACAGCACTTTGTCCAGATTAGCGTCGGCCAGGATCACTCTCTTGGAGAACTCGCTGA
Sanger sequencing data after PCR amplification and pJET cloning:
Clone 1: In13-371(39bp)-ITR(-71bp)-Cas9rev
NNNagttattttacacatgaatggacagttaccattatatatctgaatcatttcacattccctcccatgCCTGCAGGCAGCTGCGCGCTCGCTCGCTCACTGAGGCCGCCCGGGCAAAGCCCGGGCGTCGGGCGACCTTTGGTCGCCCGGCCTCAGTGAGCGAGCGAGCGCGCAGAGAGGGAGTGGCCAACTCCATCACTAGGGGTTCCTGCGGCCGCCGAGNNN
Clone 2: In13-371-ITR(-97bp)-Cas9rev
NNNagttattttacacatgaatggacagttaccattatatatctgaatcatttcacattccctcccatgCCTGCAGGCAGCTGCGCGCTCGCTCGCTCACTGAGGCCGCCCGGGCAAAGCCCGGGCGTCGGGCGACCTTTGGTCGCCCGGCCTCAGTGAGCGAGCGAGCGCGCAGAGAGGGAGTGGCCAACTCCATCACTAGGGGTTCCTGCGGCCGCCGAGNNN
Clone 3: In13-371(-35bp)-ITR(-109bp)-Cas9rev
NNNagttattttacacatgaatggacagttaccattatatatctgaatcatttcacattccctcccatgCCTGCAGGCAGCTGCGCGCTCGCTCGCTCACTGAGGCCGCCCGGGCAAAGCCCGGGCGTCGGGCGACCTTTGGTCGCCCGGCCTCAGTGAGCGAGCGAGCGCGCAGAGAGGGAGTGGCCAACTCCATCACTAGGGGTTCCTGCGGCCGCCGAGNNN
Clone 4: In13-371-ITR(-109bp)-Cas9rev(+184bp)
NNNagttattttacacatgaatggacagttaccattatatatctgaatcatttcacattccctcccatgCCTGCAGGCAGCTGCGCGCTCGCTCGCTCACTGAGGCCGCCCGGGCAAAGCCCGGGCGTCGGGCGACCTTTGGTCGCCCGGagtgcttgtgctgttccacaaacagctgtttctgctcattatcctcgggggagcccttcagcttctcatagtggctggccaggtacaggaagttcacatatttggagggcagggccagttcgtttcccttctgcagttcgccggcagaggccagcattctcttccggccgttttccagcccgggCCTCAGTGAGCGAGCGAGCGCGCAGAGAGGGAGTGGCCAACTCCATCACTAGGGGTTCCTGCGGCCGCCGAGNNN
Clone 5: In13-371(-6bp)-ITR(-130bp)-Cas9rev
NNNagttattttacacatgaatggacagttaccattatatatctgaatcatttcacattccctcccatgCCTGCAGGCAGCTGCGCGCTCGCTCGCTCACTGAGGCCGCCCGGGCAAAGCCCGGGCGTCGGGCGACCTTTGGTCGCCCGGCCTCAGTGAGCGAGCGAGCGCGCAGAGAGGGAGTGGCCAACTCCATCACTAGGGGTTCCTGCGGCCGCCGAGNNN
Clone 6: In13-371(-4bp)-ITR(-130bp)-Cas9rev
NNNagttattttacacatgaatggacagttaccattatatatctgaatcatttcacattccctcccatgCCTGCAGGCAGCTGCGCGCTCGCTCGCTCACTGAGGCCGCCCGGGCAAAGCCCGGGCGTCGGGCGACCTTTGGTCGCCCGGCCTCAGTGAGCGAGCGAGCGCGCAGAGAGGGAGTGGCCAACTCCATCACTAGGGGTTCCTGCGGCCGCCGAGNNN
Clone 7: In13-371-ITR(-141bp)-Cas9rev(-9bp, +1bp)
NNNagttattttacacatgaatggacagttaccattatatatctgaatcatttcacattccctcccatgCCTGCAGGCAGCTGCGCGCTCGCTCGCTCACTGAGGCCGCCCGGGCAAAGCCCGGGCGTCGGGCGACCTTTGGTCGCCCGGCCTCAGTGAGCGAGCGAGCGCGCAGAGAGGGAGTGGCCAACTCCATCACTAGGGGTTCCTGCGGCCGCCtGAGNNN
Clone 8: In13-371(-18bp)-ITR(-141bp)-Cas9rev(-12bp)
NNNagttattttacacatgaatggacagttaccattatatatctgaatcatttcacattccctcccatgCCTGCAGGCAGCTGCGCGCTCGCTCGCTCACTGAGGCCGCCCGGGCAAAGCCCGGGCGTCGGGCGACCTTTGGTCGCCCGGCCTCAGTGAGCGAGCGAGCGCGCAGAGAGGGAGTGGCCAACTCCATCACTAGGGGTTCCTGCGGCCGCCGAGNNN
Fig. S13G. Analysis of left junction of reverse insertion of AAV-Cas9 at Alb-Intron13-371. Double strikethrough indicates deleted nucleotides (-) and red lowercase indicates inserted nucleotides (+).

## Slide 31
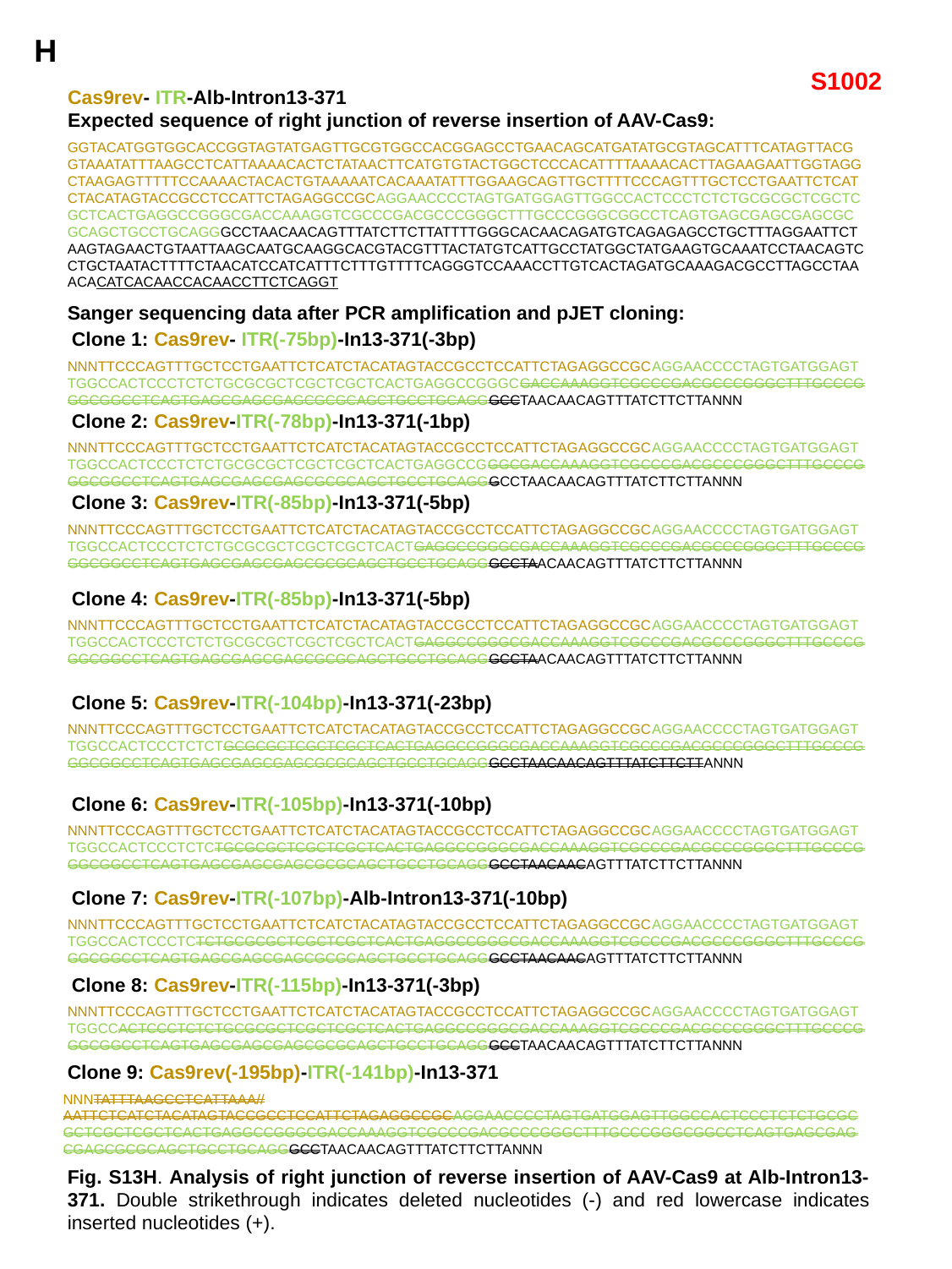

H
S1002
Cas9rev- ITR-Alb-Intron13-371
Expected sequence of right junction of reverse insertion of AAV-Cas9:
GGTACATGGTGGCACCGGTAGTATGAGTTGCGTGGCCACGGAGCCTGAACAGCATGATATGCGTAGCATTTCATAGTTACGGTAAATATTTAAGCCTCATTAAAACACTCTATAACTTCATGTGTACTGGCTCCCACATTTTAAAACACTTAGAAGAATTGGTAGGCTAAGAGTTTTTCCAAAACTACACTGTAAAAATCACAAATATTTGGAAGCAGTTGCTTTTCCCAGTTTGCTCCTGAATTCTCATCTACATAGTACCGCCTCCATTCTAGAGGCCGCAGGAACCCCTAGTGATGGAGTTGGCCACTCCCTCTCTGCGCGCTCGCTCGCTCACTGAGGCCGGGCGACCAAAGGTCGCCCGACGCCCGGGCTTTGCCCGGGCGGCCTCAGTGAGCGAGCGAGCGCGCAGCTGCCTGCAGGgcctaacaacagtttatcttcttattttgggcacaacagatgtcagagagcctgctttaggaattctaagtagaactgtaattaagcaatgcaaggcacgtacgtttactatgtcattgcctatggctatgaagtgcaaatcctaacagtcctgctaatacttttctaacatccatcatttctttgttttcagggtccaaaccttgtcactagatgcaaagacgccttagcctaaacacatcacaaccacaaccttctcaggt
Sanger sequencing data after PCR amplification and pJET cloning:
Clone 1: Cas9rev- ITR(-75bp)-In13-371(-3bp)
NNNTTCCCAGTTTGCTCCTGAATTCTCATCTACATAGTACCGCCTCCATTCTAGAGGCCGCAGGAACCCCTAGTGATGGAGTTGGCCACTCCCTCTCTGCGCGCTCGCTCGCTCACTGAGGCCGGGCGACCAAAGGTCGCCCGACGCCCGGGCTTTGCCCGGGCGGCCTCAGTGAGCGAGCGAGCGCGCAGCTGCCTGCAGGgcctaacaacagtttatcttcttaNNN
Clone 2: Cas9rev-ITR(-78bp)-In13-371(-1bp)
NNNTTCCCAGTTTGCTCCTGAATTCTCATCTACATAGTACCGCCTCCATTCTAGAGGCCGCAGGAACCCCTAGTGATGGAGTTGGCCACTCCCTCTCTGCGCGCTCGCTCGCTCACTGAGGCCGGGCGACCAAAGGTCGCCCGACGCCCGGGCTTTGCCCGGGCGGCCTCAGTGAGCGAGCGAGCGCGCAGCTGCCTGCAGGgcctaacaacagtttatcttcttaNNN
Clone 3: Cas9rev-ITR(-85bp)-In13-371(-5bp)
NNNTTCCCAGTTTGCTCCTGAATTCTCATCTACATAGTACCGCCTCCATTCTAGAGGCCGCAGGAACCCCTAGTGATGGAGTTGGCCACTCCCTCTCTGCGCGCTCGCTCGCTCACTGAGGCCGGGCGACCAAAGGTCGCCCGACGCCCGGGCTTTGCCCGGGCGGCCTCAGTGAGCGAGCGAGCGCGCAGCTGCCTGCAGGgcctaacaacagtttatcttcttaNNN
Clone 4: Cas9rev-ITR(-85bp)-In13-371(-5bp)
NNNTTCCCAGTTTGCTCCTGAATTCTCATCTACATAGTACCGCCTCCATTCTAGAGGCCGCAGGAACCCCTAGTGATGGAGTTGGCCACTCCCTCTCTGCGCGCTCGCTCGCTCACTGAGGCCGGGCGACCAAAGGTCGCCCGACGCCCGGGCTTTGCCCGGGCGGCCTCAGTGAGCGAGCGAGCGCGCAGCTGCCTGCAGGgcctaacaacagtttatcttcttaNNN
Clone 5: Cas9rev-ITR(-104bp)-In13-371(-23bp)
NNNTTCCCAGTTTGCTCCTGAATTCTCATCTACATAGTACCGCCTCCATTCTAGAGGCCGCAGGAACCCCTAGTGATGGAGTTGGCCACTCCCTCTCTGCGCGCTCGCTCGCTCACTGAGGCCGGGCGACCAAAGGTCGCCCGACGCCCGGGCTTTGCCCGGGCGGCCTCAGTGAGCGAGCGAGCGCGCAGCTGCCTGCAGGgcctaacaacagtttatcttcttaNNN
Clone 6: Cas9rev-ITR(-105bp)-In13-371(-10bp)
NNNTTCCCAGTTTGCTCCTGAATTCTCATCTACATAGTACCGCCTCCATTCTAGAGGCCGCAGGAACCCCTAGTGATGGAGTTGGCCACTCCCTCTCTGCGCGCTCGCTCGCTCACTGAGGCCGGGCGACCAAAGGTCGCCCGACGCCCGGGCTTTGCCCGGGCGGCCTCAGTGAGCGAGCGAGCGCGCAGCTGCCTGCAGGgcctaacaacagtttatcttcttaNNN
Clone 7: Cas9rev-ITR(-107bp)-Alb-Intron13-371(-10bp)
NNNTTCCCAGTTTGCTCCTGAATTCTCATCTACATAGTACCGCCTCCATTCTAGAGGCCGCAGGAACCCCTAGTGATGGAGTTGGCCACTCCCTCTCTGCGCGCTCGCTCGCTCACTGAGGCCGGGCGACCAAAGGTCGCCCGACGCCCGGGCTTTGCCCGGGCGGCCTCAGTGAGCGAGCGAGCGCGCAGCTGCCTGCAGGgcctaacaacagtttatcttcttaNNN
Clone 8: Cas9rev-ITR(-115bp)-In13-371(-3bp)
NNNTTCCCAGTTTGCTCCTGAATTCTCATCTACATAGTACCGCCTCCATTCTAGAGGCCGCAGGAACCCCTAGTGATGGAGTTGGCCACTCCCTCTCTGCGCGCTCGCTCGCTCACTGAGGCCGGGCGACCAAAGGTCGCCCGACGCCCGGGCTTTGCCCGGGCGGCCTCAGTGAGCGAGCGAGCGCGCAGCTGCCTGCAGGgcctaacaacagtttatcttcttaNNN
Clone 9: Cas9rev(-195bp)-ITR(-141bp)-In13-371
NNNTATTTAAGCCTCATTAAA//AATTCTCATCTACATAGTACCGCCTCCATTCTAGAGGCCGCAGGAACCCCTAGTGATGGAGTTGGCCACTCCCTCTCTGCGCGCTCGCTCGCTCACTGAGGCCGGGCGACCAAAGGTCGCCCGACGCCCGGGCTTTGCCCGGGCGGCCTCAGTGAGCGAGCGAGCGCGCAGCTGCCTGCAGGgcctaacaacagtttatcttcttaNNN
Fig. S13H. Analysis of right junction of reverse insertion of AAV-Cas9 at Alb-Intron13-371. Double strikethrough indicates deleted nucleotides (-) and red lowercase indicates inserted nucleotides (+).

## Slide 32
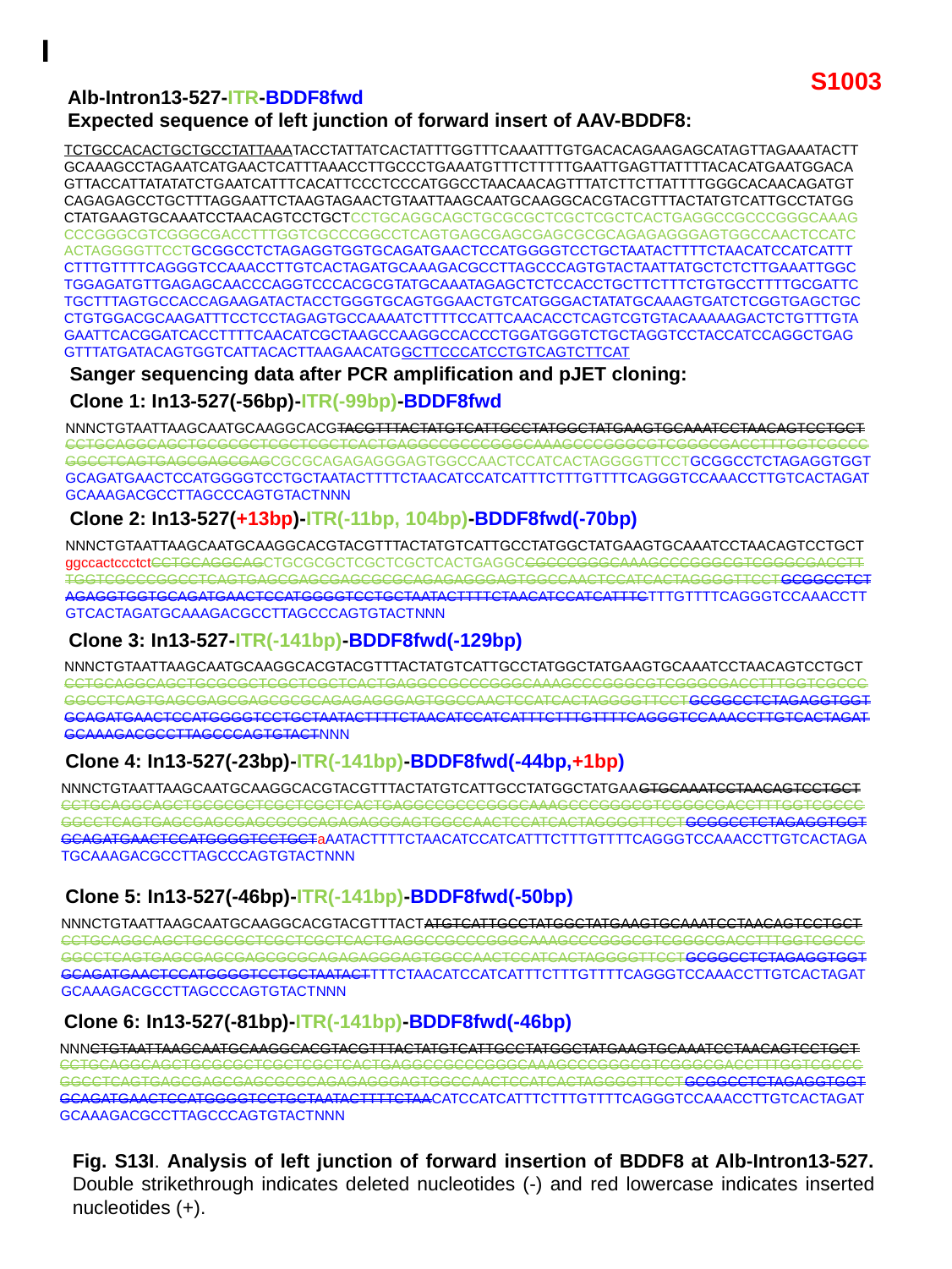

I
S1003
Alb-Intron13-527-ITR-BDDF8fwd
Expected sequence of left junction of forward insert of AAV-BDDF8:
tctgccacactgctgcctattaaatacctattatcactatttggtttcaaatttgtgacacagaagagcatagttagaaatacttgcaaagcctagaatcatgaactcatttaaaccttgccctgaaatgtttctttttgaattgagttattttacacatgaatggacagttaccattatatatctgaatcatttcacattccctcccatggcctaacaacagtttatcttcttattttgggcacaacagatgtcagagagcctgctttaggaattctaagtagaactgtaattaagcaatgcaaggcacgtacgtttactatgtcattgcctatggctatgaagtgcaaatcctaacagtcctgctCCTGCAGGCAGCTGCGCGCTCGCTCGCTCACTGAGGCCGCCCGGGCAAAGCCCGGGCGTCGGGCGACCTTTGGTCGCCCGGCCTCAGTGAGCGAGCGAGCGCGCAGAGAGGGAGTGGCCAACTCCATCACTAGGGGTTCCTGCGGCCTCTAGAGGTGGTGCAGATGAACTCCATGGGGTCCTGCTAATACTTTTCTAACATCCATCATTTCTTTGTTTTCAGGGTCCAAACCTTGTCACTAGATGCAAAGACGCCTTAGCCCAGTGTACTAATTATGCTCTCTTGAAATTGGCTGGAGATGTTGAGAGCAACCCAGGTCCCACGCGTATGCAAATAGAGCTCTCCACCTGCTTCTTTCTGTGCCTTTTGCGATTCTGCTTTAGTGCCACCAGAAGATACTACCTGGGTGCAGTGGAACTGTCATGGGACTATATGCAAAGTGATCTCGGTGAGCTGCCTGTGGACGCAAGATTTCCTCCTAGAGTGCCAAAATCTTTTCCATTCAACACCTCAGTCGTGTACAAAAAGACTCTGTTTGTAGAATTCACGGATCACCTTTTCAACATCGCTAAGCCAAGGCCACCCTGGATGGGTCTGCTAGGTCCTACCATCCAGGCTGAGGTTTATGATACAGTGGTCATTACACTTAAGAACATGGCTTCCCATCCTGTCAGTCTTCAT
Sanger sequencing data after PCR amplification and pJET cloning:
Clone 1: In13-527(-56bp)-ITR(-99bp)-BDDF8fwd
NNNCTGTAATTAAGCAATGCAAGGCACGTACGTTTACTATGTCATTGCCTATGGCTATGAAGTGCAAATCCTAACAGTCCTGCTCCTGCAGGCAGCTGCGCGCTCGCTCGCTCACTGAGGCCGCCCGGGCAAAGCCCGGGCGTCGGGCGACCTTTGGTCGCCCGGCCTCAGTGAGCGAGCGAGCGCGCAGAGAGGGAGTGGCCAACTCCATCACTAGGGGTTCCTGCGGCCTCTAGAGGTGGTGCAGATGAACTCCATGGGGTCCTGCTAATACTTTTCTAACATCCATCATTTCTTTGTTTTCAGGGTCCAAACCTTGTCACTAGATGCAAAGACGCCTTAGCCCAGTGTACTNNN
Clone 2: In13-527(+13bp)-ITR(-11bp, 104bp)-BDDF8fwd(-70bp)
NNNCTGTAATTAAGCAATGCAAGGCACGTACGTTTACTATGTCATTGCCTATGGCTATGAAGTGCAAATCCTAACAGTCCTGCTggccactccctctCCTGCAGGCAGCTGCGCGCTCGCTCGCTCACTGAGGCCGCCCGGGCAAAGCCCGGGCGTCGGGCGACCTTTGGTCGCCCGGCCTCAGTGAGCGAGCGAGCGCGCAGAGAGGGAGTGGCCAACTCCATCACTAGGGGTTCCTGCGGCCTCTAGAGGTGGTGCAGATGAACTCCATGGGGTCCTGCTAATACTTTTCTAACATCCATCATTTCTTTGTTTTCAGGGTCCAAACCTTGTCACTAGATGCAAAGACGCCTTAGCCCAGTGTACTNNN
Clone 3: In13-527-ITR(-141bp)-BDDF8fwd(-129bp)
NNNCTGTAATTAAGCAATGCAAGGCACGTACGTTTACTATGTCATTGCCTATGGCTATGAAGTGCAAATCCTAACAGTCCTGCTCCTGCAGGCAGCTGCGCGCTCGCTCGCTCACTGAGGCCGCCCGGGCAAAGCCCGGGCGTCGGGCGACCTTTGGTCGCCCGGCCTCAGTGAGCGAGCGAGCGCGCAGAGAGGGAGTGGCCAACTCCATCACTAGGGGTTCCTGCGGCCTCTAGAGGTGGTGCAGATGAACTCCATGGGGTCCTGCTAATACTTTTCTAACATCCATCATTTCTTTGTTTTCAGGGTCCAAACCTTGTCACTAGATGCAAAGACGCCTTAGCCCAGTGTACTNNN
Clone 4: In13-527(-23bp)-ITR(-141bp)-BDDF8fwd(-44bp,+1bp)
NNNCTGTAATTAAGCAATGCAAGGCACGTACGTTTACTATGTCATTGCCTATGGCTATGAAGTGCAAATCCTAACAGTCCTGCTCCTGCAGGCAGCTGCGCGCTCGCTCGCTCACTGAGGCCGCCCGGGCAAAGCCCGGGCGTCGGGCGACCTTTGGTCGCCCGGCCTCAGTGAGCGAGCGAGCGCGCAGAGAGGGAGTGGCCAACTCCATCACTAGGGGTTCCTGCGGCCTCTAGAGGTGGTGCAGATGAACTCCATGGGGTCCTGCTaAATACTTTTCTAACATCCATCATTTCTTTGTTTTCAGGGTCCAAACCTTGTCACTAGATGCAAAGACGCCTTAGCCCAGTGTACTNNN
Clone 5: In13-527(-46bp)-ITR(-141bp)-BDDF8fwd(-50bp)
NNNCTGTAATTAAGCAATGCAAGGCACGTACGTTTACTATGTCATTGCCTATGGCTATGAAGTGCAAATCCTAACAGTCCTGCTCCTGCAGGCAGCTGCGCGCTCGCTCGCTCACTGAGGCCGCCCGGGCAAAGCCCGGGCGTCGGGCGACCTTTGGTCGCCCGGCCTCAGTGAGCGAGCGAGCGCGCAGAGAGGGAGTGGCCAACTCCATCACTAGGGGTTCCTGCGGCCTCTAGAGGTGGTGCAGATGAACTCCATGGGGTCCTGCTAATACTTTTCTAACATCCATCATTTCTTTGTTTTCAGGGTCCAAACCTTGTCACTAGATGCAAAGACGCCTTAGCCCAGTGTACTNNN
Clone 6: In13-527(-81bp)-ITR(-141bp)-BDDF8fwd(-46bp)
NNNCTGTAATTAAGCAATGCAAGGCACGTACGTTTACTATGTCATTGCCTATGGCTATGAAGTGCAAATCCTAACAGTCCTGCTCCTGCAGGCAGCTGCGCGCTCGCTCGCTCACTGAGGCCGCCCGGGCAAAGCCCGGGCGTCGGGCGACCTTTGGTCGCCCGGCCTCAGTGAGCGAGCGAGCGCGCAGAGAGGGAGTGGCCAACTCCATCACTAGGGGTTCCTGCGGCCTCTAGAGGTGGTGCAGATGAACTCCATGGGGTCCTGCTAATACTTTTCTAACATCCATCATTTCTTTGTTTTCAGGGTCCAAACCTTGTCACTAGATGCAAAGACGCCTTAGCCCAGTGTACTNNN
Fig. S13I. Analysis of left junction of forward insertion of BDDF8 at Alb-Intron13-527. Double strikethrough indicates deleted nucleotides (-) and red lowercase indicates inserted nucleotides (+).

## Slide 33
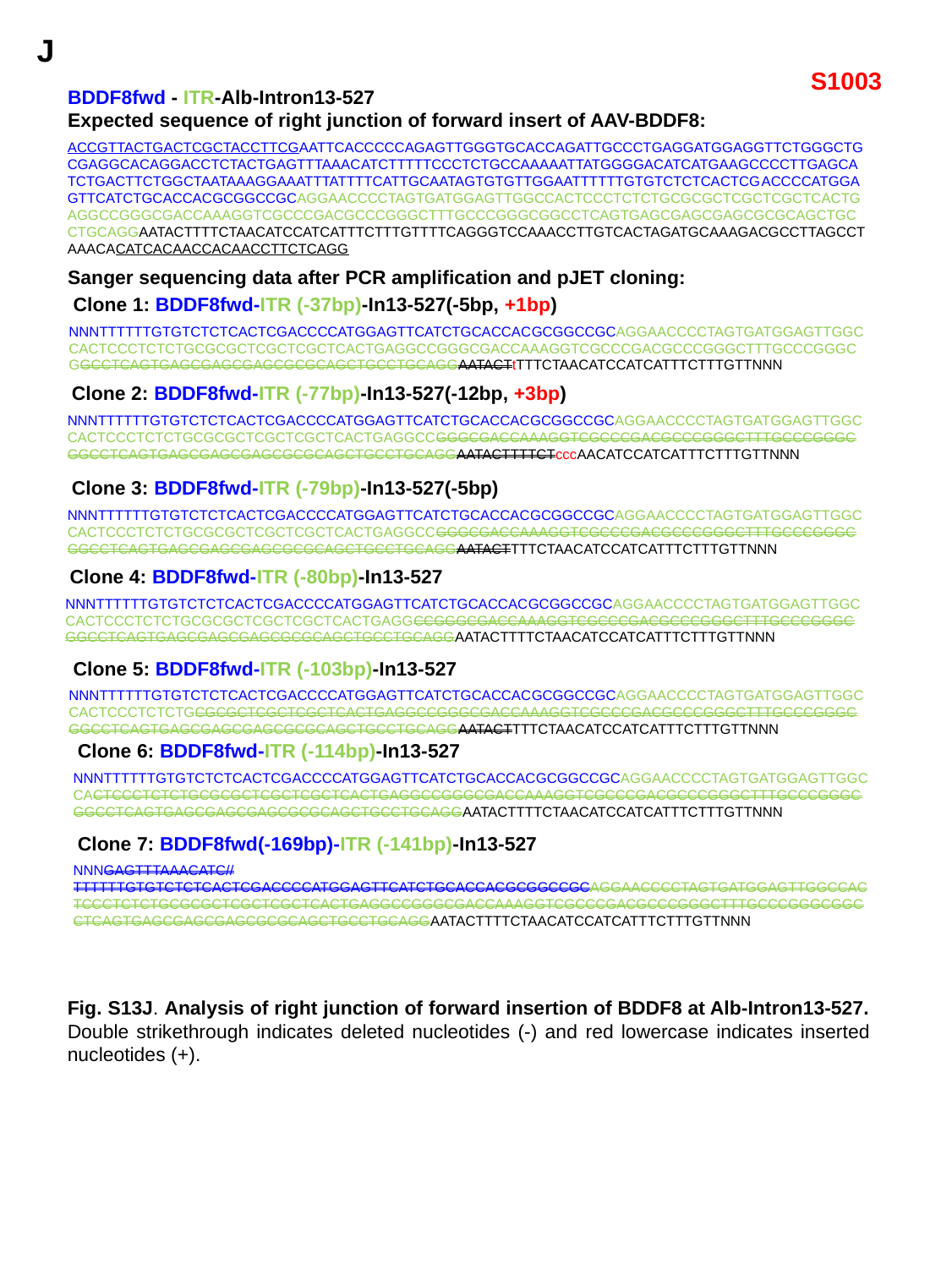

J
S1003
BDDF8fwd - ITR-Alb-Intron13-527
Expected sequence of right junction of forward insert of AAV-BDDF8:
ACCGTTACTGACTCGCTACCTTCGAATTCACCCCCAGAGTTGGGTGCACCAGATTGCCCTGAGGATGGAGGTTCTGGGCTGCGAGGCACAGGACCTCTACTGAGTTTAAACATCTTTTTCCCTCTGCCAAAAATTATGGGGACATCATGAAGCCCCTTGAGCATCTGACTTCTGGCTAATAAAGGAAATTTATTTTCATTGCAATAGTGTGTTGGAATTTTTTGTGTCTCTCACTCGACCCCATGGAGTTCATCTGCACCACGCGGCCGCAGGAACCCCTAGTGATGGAGTTGGCCACTCCCTCTCTGCGCGCTCGCTCGCTCACTGAGGCCGGGCGACCAAAGGTCGCCCGACGCCCGGGCTTTGCCCGGGCGGCCTCAGTGAGCGAGCGAGCGCGCAGCTGCCTGCAGGaatacttttctaacatccatcatttctttgttttcagggtccaaaccttgtcactagatgcaaagacgccttagcctaaacacatcacaaccacaaccttctcagg
Sanger sequencing data after PCR amplification and pJET cloning:
Clone 1: BDDF8fwd-ITR (-37bp)-In13-527(-5bp, +1bp)
NNNTTTTTTGTGTCTCTCACTCGACCCCATGGAGTTCATCTGCACCACGCGGCCGCAGGAACCCCTAGTGATGGAGTTGGCCACTCCCTCTCTGCGCGCTCGCTCGCTCACTGAGGCCGGGCGACCAAAGGTCGCCCGACGCCCGGGCTTTGCCCGGGCGGCCTCAGTGAGCGAGCGAGCGCGCAGCTGCCTGCAGGaatactttttctaacatccatcatttctttgttNNN
Clone 2: BDDF8fwd-ITR (-77bp)-In13-527(-12bp, +3bp)
NNNTTTTTTGTGTCTCTCACTCGACCCCATGGAGTTCATCTGCACCACGCGGCCGCAGGAACCCCTAGTGATGGAGTTGGCCACTCCCTCTCTGCGCGCTCGCTCGCTCACTGAGGCCGGGCGACCAAAGGTCGCCCGACGCCCGGGCTTTGCCCGGGCGGCCTCAGTGAGCGAGCGAGCGCGCAGCTGCCTGCAGGaatacttttctcccaacatccatcatttctttgttNNN
Clone 3: BDDF8fwd-ITR (-79bp)-In13-527(-5bp)
NNNTTTTTTGTGTCTCTCACTCGACCCCATGGAGTTCATCTGCACCACGCGGCCGCAGGAACCCCTAGTGATGGAGTTGGCCACTCCCTCTCTGCGCGCTCGCTCGCTCACTGAGGCCGGGCGACCAAAGGTCGCCCGACGCCCGGGCTTTGCCCGGGCGGCCTCAGTGAGCGAGCGAGCGCGCAGCTGCCTGCAGGaatacttttctaacatccatcatttctttgttNNN
Clone 4: BDDF8fwd-ITR (-80bp)-In13-527
NNNTTTTTTGTGTCTCTCACTCGACCCCATGGAGTTCATCTGCACCACGCGGCCGCAGGAACCCCTAGTGATGGAGTTGGCCACTCCCTCTCTGCGCGCTCGCTCGCTCACTGAGGCCGGGCGACCAAAGGTCGCCCGACGCCCGGGCTTTGCCCGGGCGGCCTCAGTGAGCGAGCGAGCGCGCAGCTGCCTGCAGGaatacttttctaacatccatcatttctttgttNNN
Clone 5: BDDF8fwd-ITR (-103bp)-In13-527
NNNTTTTTTGTGTCTCTCACTCGACCCCATGGAGTTCATCTGCACCACGCGGCCGCAGGAACCCCTAGTGATGGAGTTGGCCACTCCCTCTCTGCGCGCTCGCTCGCTCACTGAGGCCGGGCGACCAAAGGTCGCCCGACGCCCGGGCTTTGCCCGGGCGGCCTCAGTGAGCGAGCGAGCGCGCAGCTGCCTGCAGGaatacttttctaacatccatcatttctttgttNNN
Clone 6: BDDF8fwd-ITR (-114bp)-In13-527
NNNTTTTTTGTGTCTCTCACTCGACCCCATGGAGTTCATCTGCACCACGCGGCCGCAGGAACCCCTAGTGATGGAGTTGGCCACTCCCTCTCTGCGCGCTCGCTCGCTCACTGAGGCCGGGCGACCAAAGGTCGCCCGACGCCCGGGCTTTGCCCGGGCGGCCTCAGTGAGCGAGCGAGCGCGCAGCTGCCTGCAGGaatacttttctaacatccatcatttctttgttNNN
Clone 7: BDDF8fwd(-169bp)-ITR (-141bp)-In13-527
NNNGAGTTTAAACATC//TTTTTTGTGTCTCTCACTCGACCCCATGGAGTTCATCTGCACCACGCGGCCGCAGGAACCCCTAGTGATGGAGTTGGCCACTCCCTCTCTGCGCGCTCGCTCGCTCACTGAGGCCGGGCGACCAAAGGTCGCCCGACGCCCGGGCTTTGCCCGGGCGGCCTCAGTGAGCGAGCGAGCGCGCAGCTGCCTGCAGGaatacttttctaacatccatcatttctttgttNNN
Fig. S13J. Analysis of right junction of forward insertion of BDDF8 at Alb-Intron13-527. Double strikethrough indicates deleted nucleotides (-) and red lowercase indicates inserted nucleotides (+).

## Slide 34
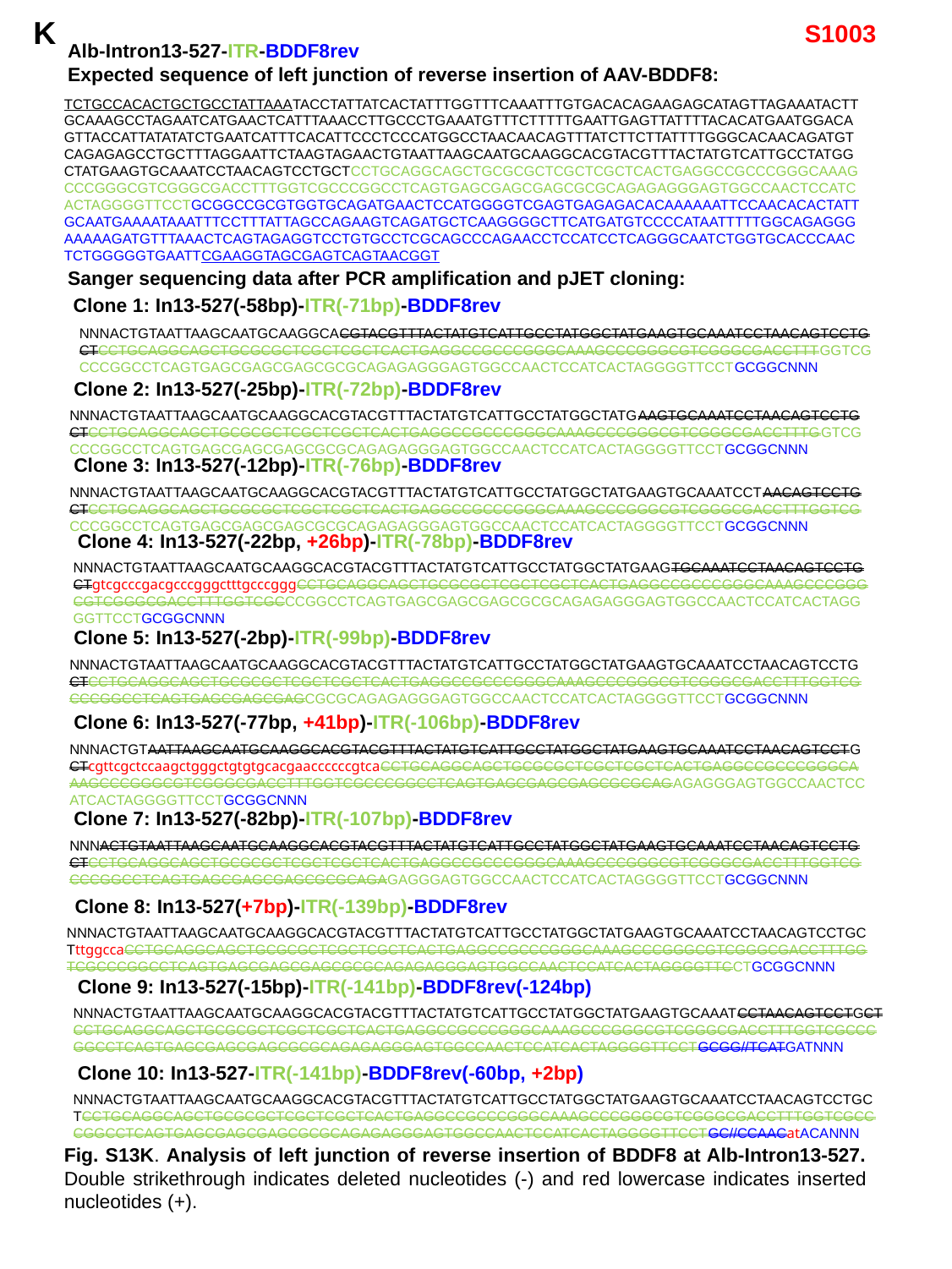

K
S1003
Alb-Intron13-527-ITR-BDDF8rev
Expected sequence of left junction of reverse insertion of AAV-BDDF8:
tctgccacactgctgcctattaaatacctattatcactatttggtttcaaatttgtgacacagaagagcatagttagaaatacttgcaaagcctagaatcatgaactcatttaaaccttgccctgaaatgtttctttttgaattgagttattttacacatgaatggacagttaccattatatatctgaatcatttcacattccctcccatggcctaacaacagtttatcttcttattttgggcacaacagatgtcagagagcctgctttaggaattctaagtagaactgtaattaagcaatgcaaggcacgtacgtttactatgtcattgcctatggctatgaagtgcaaatcctaacagtcctgctCCTGCAGGCAGCTGCGCGCTCGCTCGCTCACTGAGGCCGCCCGGGCAAAGCCCGGGCGTCGGGCGACCTTTGGTCGCCCGGCCTCAGTGAGCGAGCGAGCGCGCAGAGAGGGAGTGGCCAACTCCATCACTAGGGGTTCCTGCGGCCGCGTGGTGCAGATGAACTCCATGGGGTCGAGTGAGAGACACAAAAAATTCCAACACACTATTGCAATGAAAATAAATTTCCTTTATTAGCCAGAAGTCAGATGCTCAAGGGGCTTCATGATGTCCCCATAATTTTTGGCAGAGGGAAAAAGATGTTTAAACTCAGTAGAGGTCCTGTGCCTCGCAGCCCAGAACCTCCATCCTCAGGGCAATCTGGTGCACCCAACTCTGGGGGTGAATTCGAAGGTAGCGAGTCAGTAACGGT
Sanger sequencing data after PCR amplification and pJET cloning:
Clone 1: In13-527(-58bp)-ITR(-71bp)-BDDF8rev
NNNactgtaattaagcaatgcaaggcacgtacgtttactatgtcattgcctatggctatgaagtgcaaatcctaacagtcctgctCCTGCAGGCAGCTGCGCGCTCGCTCGCTCACTGAGGCCGCCCGGGCAAAGCCCGGGCGTCGGGCGACCTTTGGTCGCCCGGCCTCAGTGAGCGAGCGAGCGCGCAGAGAGGGAGTGGCCAACTCCATCACTAGGGGTTCCTGCGGCNNN
Clone 2: In13-527(-25bp)-ITR(-72bp)-BDDF8rev
NNNactgtaattaagcaatgcaaggcacgtacgtttactatgtcattgcctatggctatgaagtgcaaatcctaacagtcctgctCCTGCAGGCAGCTGCGCGCTCGCTCGCTCACTGAGGCCGCCCGGGCAAAGCCCGGGCGTCGGGCGACCTTTGGTCGCCCGGCCTCAGTGAGCGAGCGAGCGCGCAGAGAGGGAGTGGCCAACTCCATCACTAGGGGTTCCTGCGGCNNN
Clone 3: In13-527(-12bp)-ITR(-76bp)-BDDF8rev
NNNactgtaattaagcaatgcaaggcacgtacgtttactatgtcattgcctatggctatgaagtgcaaatcctaacagtcctgctCCTGCAGGCAGCTGCGCGCTCGCTCGCTCACTGAGGCCGCCCGGGCAAAGCCCGGGCGTCGGGCGACCTTTGGTCGCCCGGCCTCAGTGAGCGAGCGAGCGCGCAGAGAGGGAGTGGCCAACTCCATCACTAGGGGTTCCTGCGGCNNN
Clone 4: In13-527(-22bp, +26bp)-ITR(-78bp)-BDDF8rev
NNNactgtaattaagcaatgcaaggcacgtacgtttactatgtcattgcctatggctatgaagtgcaaatcctaacagtcctgctgtcgcccgacgcccgggctttgcccgggCCTGCAGGCAGCTGCGCGCTCGCTCGCTCACTGAGGCCGCCCGGGCAAAGCCCGGGCGTCGGGCGACCTTTGGTCGCCCGGCCTCAGTGAGCGAGCGAGCGCGCAGAGAGGGAGTGGCCAACTCCATCACTAGGGGTTCCTGCGGCNNN
Clone 5: In13-527(-2bp)-ITR(-99bp)-BDDF8rev
NNNactgtaattaagcaatgcaaggcacgtacgtttactatgtcattgcctatggctatgaagtgcaaatcctaacagtcctgctCCTGCAGGCAGCTGCGCGCTCGCTCGCTCACTGAGGCCGCCCGGGCAAAGCCCGGGCGTCGGGCGACCTTTGGTCGCCCGGCCTCAGTGAGCGAGCGAGCGCGCAGAGAGGGAGTGGCCAACTCCATCACTAGGGGTTCCTGCGGCNNN
Clone 6: In13-527(-77bp, +41bp)-ITR(-106bp)-BDDF8rev
NNNactgtaattaagcaatgcaaggcacgtacgtttactatgtcattgcctatggctatgaagtgcaaatcctaacagtcctgctcgttcgctccaagctgggctgtgtgcacgaaccccccgtcaCCTGCAGGCAGCTGCGCGCTCGCTCGCTCACTGAGGCCGCCCGGGCAAAGCCCGGGCGTCGGGCGACCTTTGGTCGCCCGGCCTCAGTGAGCGAGCGAGCGCGCAGAGAGGGAGTGGCCAACTCCATCACTAGGGGTTCCTGCGGCNNN
Clone 7: In13-527(-82bp)-ITR(-107bp)-BDDF8rev
NNNactgtaattaagcaatgcaaggcacgtacgtttactatgtcattgcctatggctatgaagtgcaaatcctaacagtcctgctCCTGCAGGCAGCTGCGCGCTCGCTCGCTCACTGAGGCCGCCCGGGCAAAGCCCGGGCGTCGGGCGACCTTTGGTCGCCCGGCCTCAGTGAGCGAGCGAGCGCGCAGAGAGGGAGTGGCCAACTCCATCACTAGGGGTTCCTGCGGCNNN
Clone 8: In13-527(+7bp)-ITR(-139bp)-BDDF8rev
NNNactgtaattaagcaatgcaaggcacgtacgtttactatgtcattgcctatggctatgaagtgcaaatcctaacagtcctgctttggccaCCTGCAGGCAGCTGCGCGCTCGCTCGCTCACTGAGGCCGCCCGGGCAAAGCCCGGGCGTCGGGCGACCTTTGGTCGCCCGGCCTCAGTGAGCGAGCGAGCGCGCAGAGAGGGAGTGGCCAACTCCATCACTAGGGGTTCCTGCGGCNNN
Clone 9: In13-527(-15bp)-ITR(-141bp)-BDDF8rev(-124bp)
NNNactgtaattaagcaatgcaaggcacgtacgtttactatgtcattgcctatggctatgaagtgcaaatcctaacagtcctgctCCTGCAGGCAGCTGCGCGCTCGCTCGCTCACTGAGGCCGCCCGGGCAAAGCCCGGGCGTCGGGCGACCTTTGGTCGCCCGGCCTCAGTGAGCGAGCGAGCGCGCAGAGAGGGAGTGGCCAACTCCATCACTAGGGGTTCCTGCGG//TCATGATNNN
Clone 10: In13-527-ITR(-141bp)-BDDF8rev(-60bp, +2bp)
NNNactgtaattaagcaatgcaaggcacgtacgtttactatgtcattgcctatggctatgaagtgcaaatcctaacagtcctgctCCTGCAGGCAGCTGCGCGCTCGCTCGCTCACTGAGGCCGCCCGGGCAAAGCCCGGGCGTCGGGCGACCTTTGGTCGCCCGGCCTCAGTGAGCGAGCGAGCGCGCAGAGAGGGAGTGGCCAACTCCATCACTAGGGGTTCCTGC//CCAACatACANNN
Fig. S13K. Analysis of left junction of reverse insertion of BDDF8 at Alb-Intron13-527. Double strikethrough indicates deleted nucleotides (-) and red lowercase indicates inserted nucleotides (+).

## Slide 35
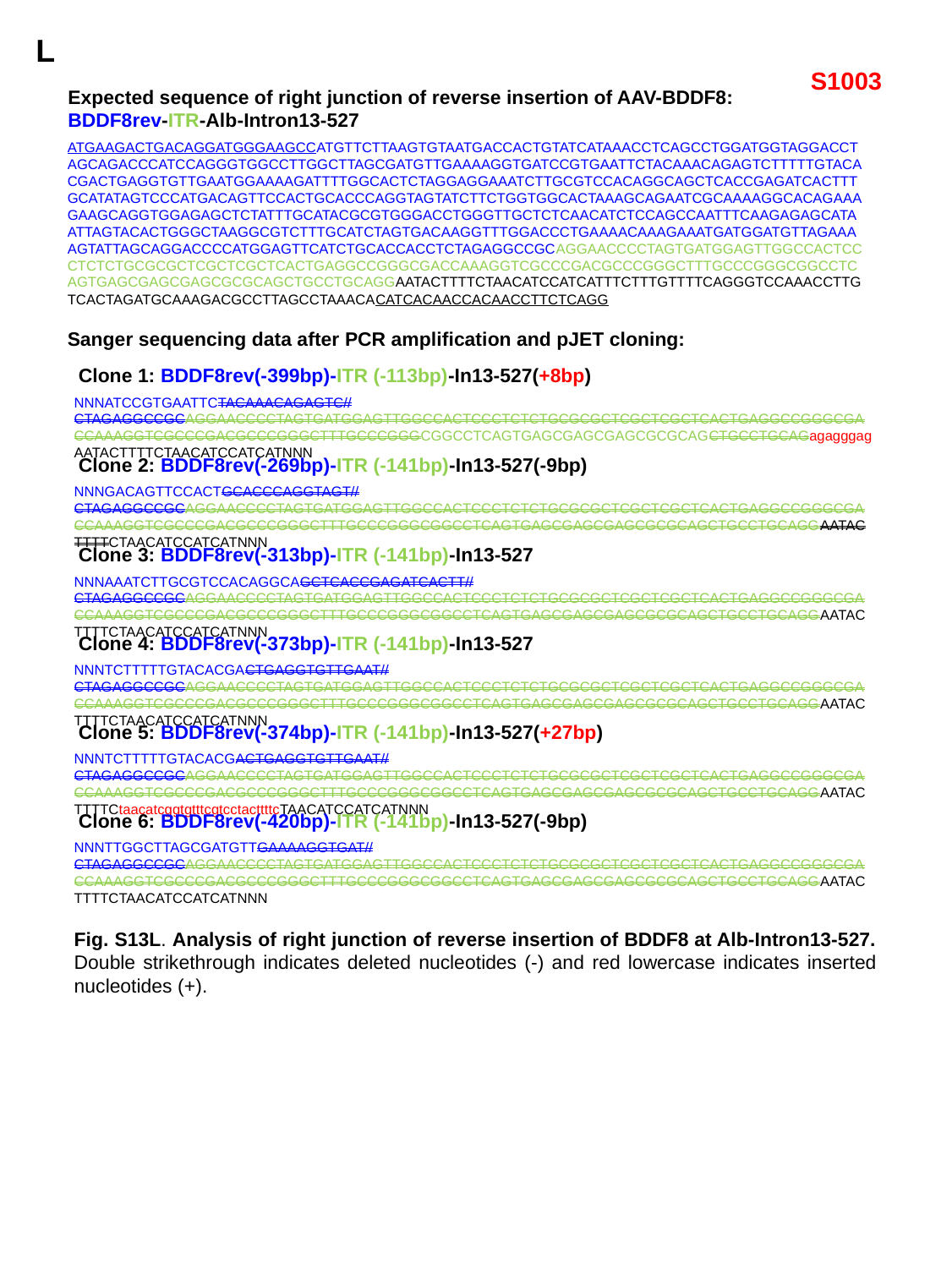

L
S1003
Expected sequence of right junction of reverse insertion of AAV-BDDF8: BDDF8rev-ITR-Alb-Intron13-527
ATGAAGACTGACAGGATGGGAAGCCATGTTCTTAAGTGTAATGACCACTGTATCATAAACCTCAGCCTGGATGGTAGGACCTAGCAGACCCATCCAGGGTGGCCTTGGCTTAGCGATGTTGAAAAGGTGATCCGTGAATTCTACAAACAGAGTCTTTTTGTACACGACTGAGGTGTTGAATGGAAAAGATTTTGGCACTCTAGGAGGAAATCTTGCGTCCACAGGCAGCTCACCGAGATCACTTTGCATATAGTCCCATGACAGTTCCACTGCACCCAGGTAGTATCTTCTGGTGGCACTAAAGCAGAATCGCAAAAGGCACAGAAAGAAGCAGGTGGAGAGCTCTATTTGCATACGCGTGGGACCTGGGTTGCTCTCAACATCTCCAGCCAATTTCAAGAGAGCATAATTAGTACACTGGGCTAAGGCGTCTTTGCATCTAGTGACAAGGTTTGGACCCTGAAAACAAAGAAATGATGGATGTTAGAAAAGTATTAGCAGGACCCCATGGAGTTCATCTGCACCACCTCTAGAGGCCGCAGGAACCCCTAGTGATGGAGTTGGCCACTCCCTCTCTGCGCGCTCGCTCGCTCACTGAGGCCGGGCGACCAAAGGTCGCCCGACGCCCGGGCTTTGCCCGGGCGGCCTCAGTGAGCGAGCGAGCGCGCAGCTGCCTGCAGGaatacttttctaacatccatcatttctttgttttcagggtccaaaccttgtcactagatgcaaagacgccttagcctaaacacatcacaaccacaaccttctcagg
Sanger sequencing data after PCR amplification and pJET cloning:
Clone 1: BDDF8rev(-399bp)-ITR (-113bp)-In13-527(+8bp)
NNNATCCGTGAATTCTACAAACAGAGTC//CTAGAGGCCGCAGGAACCCCTAGTGATGGAGTTGGCCACTCCCTCTCTGCGCGCTCGCTCGCTCACTGAGGCCGGGCGACCAAAGGTCGCCCGACGCCCGGGCTTTGCCCGGGCGGCCTCAGTGAGCGAGCGAGCGCGCAGCTGCCTGCAGagagggagaatacttttctaacatccatcatNNN
Clone 2: BDDF8rev(-269bp)-ITR (-141bp)-In13-527(-9bp)
NNNGACAGTTCCACTGCACCCAGGTAGT//CTAGAGGCCGCAGGAACCCCTAGTGATGGAGTTGGCCACTCCCTCTCTGCGCGCTCGCTCGCTCACTGAGGCCGGGCGACCAAAGGTCGCCCGACGCCCGGGCTTTGCCCGGGCGGCCTCAGTGAGCGAGCGAGCGCGCAGCTGCCTGCAGGaatacttttctaacatccatcatNNN
Clone 3: BDDF8rev(-313bp)-ITR (-141bp)-In13-527
NNNAAATCTTGCGTCCACAGGCAGCTCACCGAGATCACTT//CTAGAGGCCGCAGGAACCCCTAGTGATGGAGTTGGCCACTCCCTCTCTGCGCGCTCGCTCGCTCACTGAGGCCGGGCGACCAAAGGTCGCCCGACGCCCGGGCTTTGCCCGGGCGGCCTCAGTGAGCGAGCGAGCGCGCAGCTGCCTGCAGGaatacttttctaacatccatcatNNN
Clone 4: BDDF8rev(-373bp)-ITR (-141bp)-In13-527
NNNTCTTTTTGTACACGACTGAGGTGTTGAAT//CTAGAGGCCGCAGGAACCCCTAGTGATGGAGTTGGCCACTCCCTCTCTGCGCGCTCGCTCGCTCACTGAGGCCGGGCGACCAAAGGTCGCCCGACGCCCGGGCTTTGCCCGGGCGGCCTCAGTGAGCGAGCGAGCGCGCAGCTGCCTGCAGGaatacttttctaacatccatcatNNN
Clone 5: BDDF8rev(-374bp)-ITR (-141bp)-In13-527(+27bp)
NNNTCTTTTTGTACACGACTGAGGTGTTGAAT//CTAGAGGCCGCAGGAACCCCTAGTGATGGAGTTGGCCACTCCCTCTCTGCGCGCTCGCTCGCTCACTGAGGCCGGGCGACCAAAGGTCGCCCGACGCCCGGGCTTTGCCCGGGCGGCCTCAGTGAGCGAGCGAGCGCGCAGCTGCCTGCAGGaatacttttctaacatcggtgtttcgtcctacttttctaacatccatcatNNN
Clone 6: BDDF8rev(-420bp)-ITR (-141bp)-In13-527(-9bp)
NNNTTGGCTTAGCGATGTTGAAAAGGTGAT//CTAGAGGCCGCAGGAACCCCTAGTGATGGAGTTGGCCACTCCCTCTCTGCGCGCTCGCTCGCTCACTGAGGCCGGGCGACCAAAGGTCGCCCGACGCCCGGGCTTTGCCCGGGCGGCCTCAGTGAGCGAGCGAGCGCGCAGCTGCCTGCAGGaatacttttctaacatccatcatNNN
Fig. S13L. Analysis of right junction of reverse insertion of BDDF8 at Alb-Intron13-527. Double strikethrough indicates deleted nucleotides (-) and red lowercase indicates inserted nucleotides (+).

## Slide 36
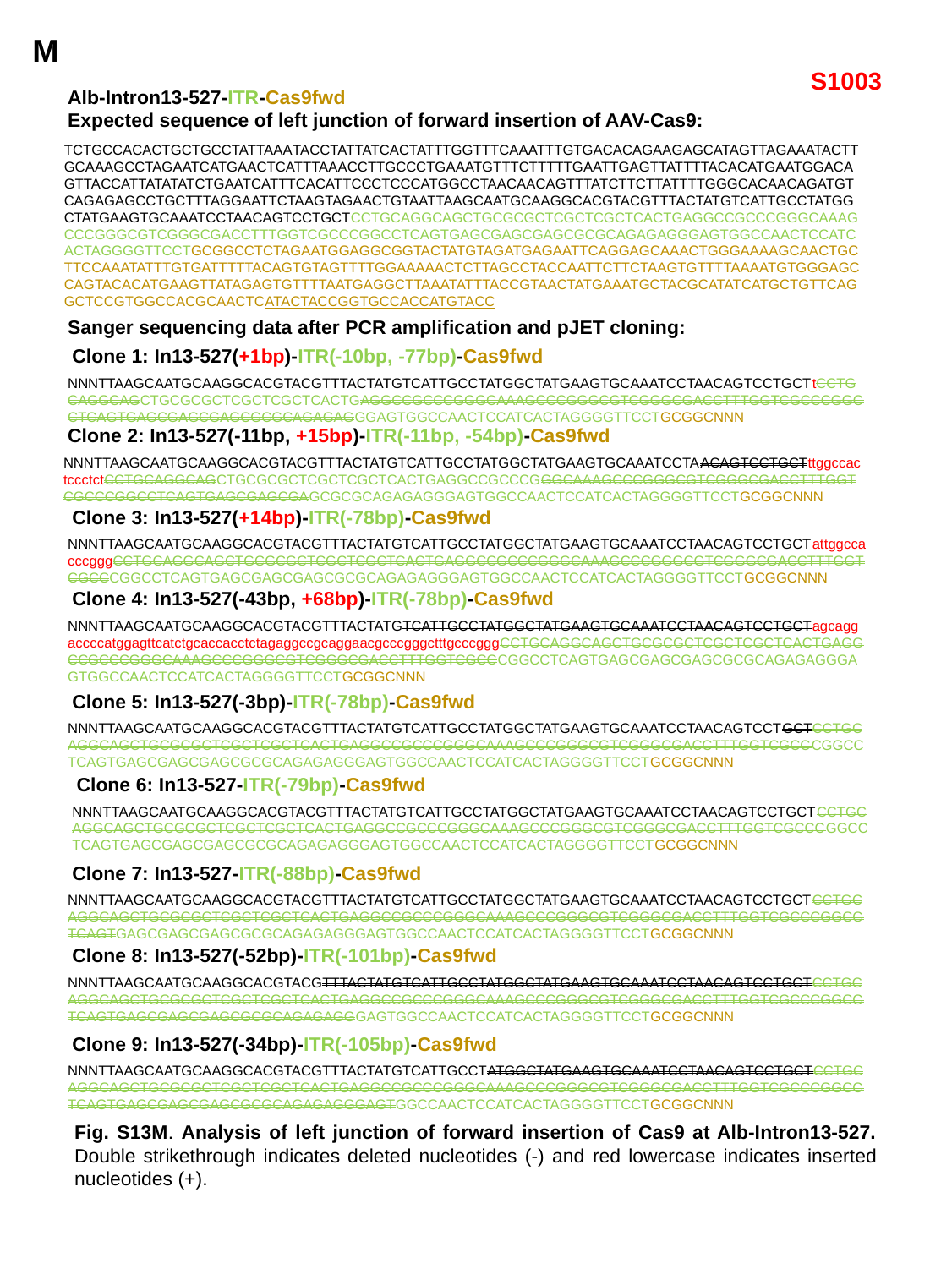

M
S1003
Alb-Intron13-527-ITR-Cas9fwd
Expected sequence of left junction of forward insertion of AAV-Cas9:
tctgccacactgctgcctattaaatacctattatcactatttggtttcaaatttgtgacacagaagagcatagttagaaatacttgcaaagcctagaatcatgaactcatttaaaccttgccctgaaatgtttctttttgaattgagttattttacacatgaatggacagttaccattatatatctgaatcatttcacattccctcccatggcctaacaacagtttatcttcttattttgggcacaacagatgtcagagagcctgctttaggaattctaagtagaactgtaattaagcaatgcaaggcacgtacgtttactatgtcattgcctatggctatgaagtgcaaatcctaacagtcctgctCCTGCAGGCAGCTGCGCGCTCGCTCGCTCACTGAGGCCGCCCGGGCAAAGCCCGGGCGTCGGGCGACCTTTGGTCGCCCGGCCTCAGTGAGCGAGCGAGCGCGCAGAGAGGGAGTGGCCAACTCCATCACTAGGGGTTCCTGCGGCCTCTAGAATGGAGGCGGTACTATGTAGATGAGAATTCAGGAGCAAACTGGGAAAAGCAACTGCTTCCAAATATTTGTGATTTTTACAGTGTAGTTTTGGAAAAACTCTTAGCCTACCAATTCTTCTAAGTGTTTTAAAATGTGGGAGCCAGTACACATGAAGTTATAGAGTGTTTTAATGAGGCTTAAATATTTACCGTAACTATGAAATGCTACGCATATCATGCTGTTCAGGCTCCGTGGCCACGCAACTCATACTACCGGTGCCACCATGTACC
Sanger sequencing data after PCR amplification and pJET cloning:
Clone 1: In13-527(+1bp)-ITR(-10bp, -77bp)-Cas9fwd
NNNttaagcaatgcaaggcacgtacgtttactatgtcattgcctatggctatgaagtgcaaatcctaacagtcctgcttCCTGCAGGCAGCTGCGCGCTCGCTCGCTCACTGAGGCCGCCCGGGCAAAGCCCGGGCGTCGGGCGACCTTTGGTCGCCCGGCCTCAGTGAGCGAGCGAGCGCGCAGAGAGGGAGTGGCCAACTCCATCACTAGGGGTTCCTGCGGCNNN
Clone 2: In13-527(-11bp, +15bp)-ITR(-11bp, -54bp)-Cas9fwd
NNNttaagcaatgcaaggcacgtacgtttactatgtcattgcctatggctatgaagtgcaaatcctaacagtcctgctttggccactccctctCCTGCAGGCAGCTGCGCGCTCGCTCGCTCACTGAGGCCGCCCGGGCAAAGCCCGGGCGTCGGGCGACCTTTGGTCGCCCGGCCTCAGTGAGCGAGCGAGCGCGCAGAGAGGGAGTGGCCAACTCCATCACTAGGGGTTCCTGCGGCNNN
Clone 3: In13-527(+14bp)-ITR(-78bp)-Cas9fwd
NNNttaagcaatgcaaggcacgtacgtttactatgtcattgcctatggctatgaagtgcaaatcctaacagtcctgctattggccacccgggCCTGCAGGCAGCTGCGCGCTCGCTCGCTCACTGAGGCCGCCCGGGCAAAGCCCGGGCGTCGGGCGACCTTTGGTCGCCCGGCCTCAGTGAGCGAGCGAGCGCGCAGAGAGGGAGTGGCCAACTCCATCACTAGGGGTTCCTGCGGCNNN
Clone 4: In13-527(-43bp, +68bp)-ITR(-78bp)-Cas9fwd
NNNttaagcaatgcaaggcacgtacgtttactatgtcattgcctatggctatgaagtgcaaatcctaacagtcctgctagcaggaccccatggagttcatctgcaccacctctagaggccgcaggaacgcccgggctttgcccgggCCTGCAGGCAGCTGCGCGCTCGCTCGCTCACTGAGGCCGCCCGGGCAAAGCCCGGGCGTCGGGCGACCTTTGGTCGCCCGGCCTCAGTGAGCGAGCGAGCGCGCAGAGAGGGAGTGGCCAACTCCATCACTAGGGGTTCCTGCGGCNNN
Clone 5: In13-527(-3bp)-ITR(-78bp)-Cas9fwd
NNNttaagcaatgcaaggcacgtacgtttactatgtcattgcctatggctatgaagtgcaaatcctaacagtcctgctCCTGCAGGCAGCTGCGCGCTCGCTCGCTCACTGAGGCCGCCCGGGCAAAGCCCGGGCGTCGGGCGACCTTTGGTCGCCCGGCCTCAGTGAGCGAGCGAGCGCGCAGAGAGGGAGTGGCCAACTCCATCACTAGGGGTTCCTGCGGCNNN
Clone 6: In13-527-ITR(-79bp)-Cas9fwd
NNNttaagcaatgcaaggcacgtacgtttactatgtcattgcctatggctatgaagtgcaaatcctaacagtcctgctCCTGCAGGCAGCTGCGCGCTCGCTCGCTCACTGAGGCCGCCCGGGCAAAGCCCGGGCGTCGGGCGACCTTTGGTCGCCCGGCCTCAGTGAGCGAGCGAGCGCGCAGAGAGGGAGTGGCCAACTCCATCACTAGGGGTTCCTGCGGCNNN
Clone 7: In13-527-ITR(-88bp)-Cas9fwd
NNNttaagcaatgcaaggcacgtacgtttactatgtcattgcctatggctatgaagtgcaaatcctaacagtcctgctCCTGCAGGCAGCTGCGCGCTCGCTCGCTCACTGAGGCCGCCCGGGCAAAGCCCGGGCGTCGGGCGACCTTTGGTCGCCCGGCCTCAGTGAGCGAGCGAGCGCGCAGAGAGGGAGTGGCCAACTCCATCACTAGGGGTTCCTGCGGCNNN
Clone 8: In13-527(-52bp)-ITR(-101bp)-Cas9fwd
NNNttaagcaatgcaaggcacgtacgtttactatgtcattgcctatggctatgaagtgcaaatcctaacagtcctgctCCTGCAGGCAGCTGCGCGCTCGCTCGCTCACTGAGGCCGCCCGGGCAAAGCCCGGGCGTCGGGCGACCTTTGGTCGCCCGGCCTCAGTGAGCGAGCGAGCGCGCAGAGAGGGAGTGGCCAACTCCATCACTAGGGGTTCCTGCGGCNNN
Clone 9: In13-527(-34bp)-ITR(-105bp)-Cas9fwd
NNNttaagcaatgcaaggcacgtacgtttactatgtcattgcctatggctatgaagtgcaaatcctaacagtcctgctCCTGCAGGCAGCTGCGCGCTCGCTCGCTCACTGAGGCCGCCCGGGCAAAGCCCGGGCGTCGGGCGACCTTTGGTCGCCCGGCCTCAGTGAGCGAGCGAGCGCGCAGAGAGGGAGTGGCCAACTCCATCACTAGGGGTTCCTGCGGCNNN
Fig. S13M. Analysis of left junction of forward insertion of Cas9 at Alb-Intron13-527. Double strikethrough indicates deleted nucleotides (-) and red lowercase indicates inserted nucleotides (+).

## Slide 37
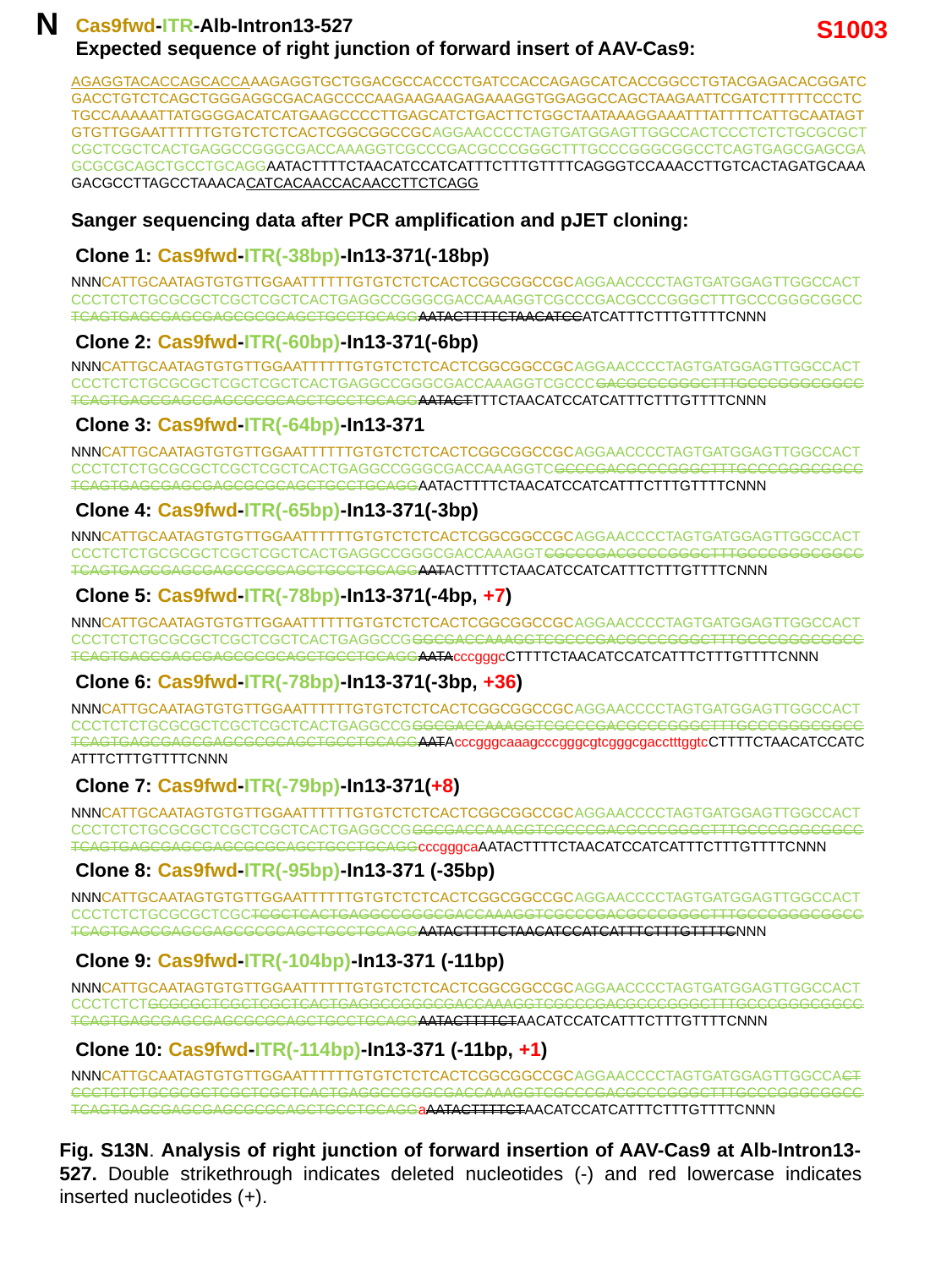

N
Cas9fwd-ITR-Alb-Intron13-527
Expected sequence of right junction of forward insert of AAV-Cas9:
S1003
AGAGGTACACCAGCACCAAAGAGGTGCTGGACGCCACCCTGATCCACCAGAGCATCACCGGCCTGTACGAGACACGGATCGACCTGTCTCAGCTGGGAGGCGACAGCCCCAAGAAGAAGAGAAAGGTGGAGGCCAGCTAAGAATTCGATCTTTTTCCCTCTGCCAAAAATTATGGGGACATCATGAAGCCCCTTGAGCATCTGACTTCTGGCTAATAAAGGAAATTTATTTTCATTGCAATAGTGTGTTGGAATTTTTTGTGTCTCTCACTCGGCGGCCGCAGGAACCCCTAGTGATGGAGTTGGCCACTCCCTCTCTGCGCGCTCGCTCGCTCACTGAGGCCGGGCGACCAAAGGTCGCCCGACGCCCGGGCTTTGCCCGGGCGGCCTCAGTGAGCGAGCGAGCGCGCAGCTGCCTGCAGGaatacttttctaacatccatcatttctttgttttcagggtccaaaccttgtcactagatgcaaagacgccttagcctaaacacatcacaaccacaaccttctcagg
Sanger sequencing data after PCR amplification and pJET cloning:
Clone 1: Cas9fwd-ITR(-38bp)-In13-371(-18bp)
NNNCATTGCAATAGTGTGTTGGAATTTTTTGTGTCTCTCACTCGGCGGCCGCAGGAACCCCTAGTGATGGAGTTGGCCACTCCCTCTCTGCGCGCTCGCTCGCTCACTGAGGCCGGGCGACCAAAGGTCGCCCGACGCCCGGGCTTTGCCCGGGCGGCCTCAGTGAGCGAGCGAGCGCGCAGCTGCCTGCAGGaatacttttctaacatccatcatttctttgttttcNNN
Clone 2: Cas9fwd-ITR(-60bp)-In13-371(-6bp)
NNNCATTGCAATAGTGTGTTGGAATTTTTTGTGTCTCTCACTCGGCGGCCGCAGGAACCCCTAGTGATGGAGTTGGCCACTCCCTCTCTGCGCGCTCGCTCGCTCACTGAGGCCGGGCGACCAAAGGTCGCCCGACGCCCGGGCTTTGCCCGGGCGGCCTCAGTGAGCGAGCGAGCGCGCAGCTGCCTGCAGGaatacttttctaacatccatcatttctttgttttcNNN
Clone 3: Cas9fwd-ITR(-64bp)-In13-371
NNNCATTGCAATAGTGTGTTGGAATTTTTTGTGTCTCTCACTCGGCGGCCGCAGGAACCCCTAGTGATGGAGTTGGCCACTCCCTCTCTGCGCGCTCGCTCGCTCACTGAGGCCGGGCGACCAAAGGTCGCCCGACGCCCGGGCTTTGCCCGGGCGGCCTCAGTGAGCGAGCGAGCGCGCAGCTGCCTGCAGGaatacttttctaacatccatcatttctttgttttcNNN
Clone 4: Cas9fwd-ITR(-65bp)-In13-371(-3bp)
NNNCATTGCAATAGTGTGTTGGAATTTTTTGTGTCTCTCACTCGGCGGCCGCAGGAACCCCTAGTGATGGAGTTGGCCACTCCCTCTCTGCGCGCTCGCTCGCTCACTGAGGCCGGGCGACCAAAGGTCGCCCGACGCCCGGGCTTTGCCCGGGCGGCCTCAGTGAGCGAGCGAGCGCGCAGCTGCCTGCAGGaatacttttctaacatccatcatttctttgttttcNNN
Clone 5: Cas9fwd-ITR(-78bp)-In13-371(-4bp, +7)
NNNCATTGCAATAGTGTGTTGGAATTTTTTGTGTCTCTCACTCGGCGGCCGCAGGAACCCCTAGTGATGGAGTTGGCCACTCCCTCTCTGCGCGCTCGCTCGCTCACTGAGGCCGGGCGACCAAAGGTCGCCCGACGCCCGGGCTTTGCCCGGGCGGCCTCAGTGAGCGAGCGAGCGCGCAGCTGCCTGCAGGaatacccgggccttttctaacatccatcatttctttgttttcNNN
Clone 6: Cas9fwd-ITR(-78bp)-In13-371(-3bp, +36)
NNNCATTGCAATAGTGTGTTGGAATTTTTTGTGTCTCTCACTCGGCGGCCGCAGGAACCCCTAGTGATGGAGTTGGCCACTCCCTCTCTGCGCGCTCGCTCGCTCACTGAGGCCGGGCGACCAAAGGTCGCCCGACGCCCGGGCTTTGCCCGGGCGGCCTCAGTGAGCGAGCGAGCGCGCAGCTGCCTGCAGGaatacccgggcaaagcccgggcgtcgggcgacctttggtccttttctaacatccatcatttctttgttttcNNN
Clone 7: Cas9fwd-ITR(-79bp)-In13-371(+8)
NNNCATTGCAATAGTGTGTTGGAATTTTTTGTGTCTCTCACTCGGCGGCCGCAGGAACCCCTAGTGATGGAGTTGGCCACTCCCTCTCTGCGCGCTCGCTCGCTCACTGAGGCCGGGCGACCAAAGGTCGCCCGACGCCCGGGCTTTGCCCGGGCGGCCTCAGTGAGCGAGCGAGCGCGCAGCTGCCTGCAGGcccgggcaaatacttttctaacatccatcatttctttgttttcNNN
Clone 8: Cas9fwd-ITR(-95bp)-In13-371 (-35bp)
NNNCATTGCAATAGTGTGTTGGAATTTTTTGTGTCTCTCACTCGGCGGCCGCAGGAACCCCTAGTGATGGAGTTGGCCACTCCCTCTCTGCGCGCTCGCTCGCTCACTGAGGCCGGGCGACCAAAGGTCGCCCGACGCCCGGGCTTTGCCCGGGCGGCCTCAGTGAGCGAGCGAGCGCGCAGCTGCCTGCAGGaatacttttctaacatccatcatttctttgttttcNNN
Clone 9: Cas9fwd-ITR(-104bp)-In13-371 (-11bp)
NNNCATTGCAATAGTGTGTTGGAATTTTTTGTGTCTCTCACTCGGCGGCCGCAGGAACCCCTAGTGATGGAGTTGGCCACTCCCTCTCTGCGCGCTCGCTCGCTCACTGAGGCCGGGCGACCAAAGGTCGCCCGACGCCCGGGCTTTGCCCGGGCGGCCTCAGTGAGCGAGCGAGCGCGCAGCTGCCTGCAGGaatacttttctaacatccatcatttctttgttttcNNN
Clone 10: Cas9fwd-ITR(-114bp)-In13-371 (-11bp, +1)
NNNCATTGCAATAGTGTGTTGGAATTTTTTGTGTCTCTCACTCGGCGGCCGCAGGAACCCCTAGTGATGGAGTTGGCCACTCCCTCTCTGCGCGCTCGCTCGCTCACTGAGGCCGGGCGACCAAAGGTCGCCCGACGCCCGGGCTTTGCCCGGGCGGCCTCAGTGAGCGAGCGAGCGCGCAGCTGCCTGCAGGaaatacttttctaacatccatcatttctttgttttcNNN
Fig. S13N. Analysis of right junction of forward insertion of AAV-Cas9 at Alb-Intron13-527. Double strikethrough indicates deleted nucleotides (-) and red lowercase indicates inserted nucleotides (+).

## Slide 38
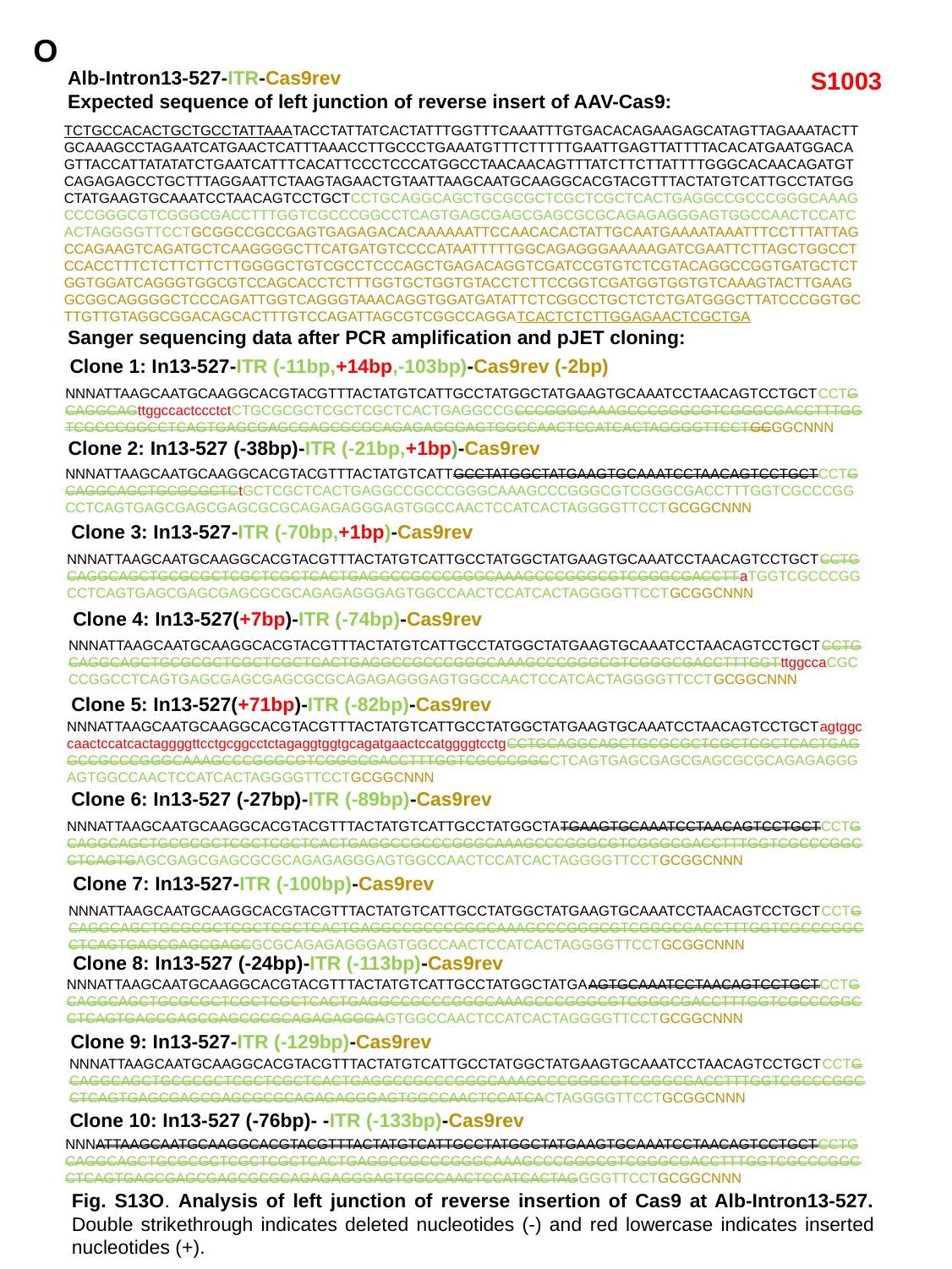

O
S1003
Alb-Intron13-527-ITR-Cas9rev
Expected sequence of left junction of reverse insert of AAV-Cas9:
tctgccacactgctgcctattaaatacctattatcactatttggtttcaaatttgtgacacagaagagcatagttagaaatacttgcaaagcctagaatcatgaactcatttaaaccttgccctgaaatgtttctttttgaattgagttattttacacatgaatggacagttaccattatatatctgaatcatttcacattccctcccatggcctaacaacagtttatcttcttattttgggcacaacagatgtcagagagcctgctttaggaattctaagtagaactgtaattaagcaatgcaaggcacgtacgtttactatgtcattgcctatggctatgaagtgcaaatcctaacagtcctgctCCTGCAGGCAGCTGCGCGCTCGCTCGCTCACTGAGGCCGCCCGGGCAAAGCCCGGGCGTCGGGCGACCTTTGGTCGCCCGGCCTCAGTGAGCGAGCGAGCGCGCAGAGAGGGAGTGGCCAACTCCATCACTAGGGGTTCCTGCGGCCGCCGAGTGAGAGACACAAAAAATTCCAACACACTATTGCAATGAAAATAAATTTCCTTTATTAGCCAGAAGTCAGATGCTCAAGGGGCTTCATGATGTCCCCATAATTTTTGGCAGAGGGAAAAAGATCGAATTCTTAGCTGGCCTCCACCTTTCTCTTCTTCTTGGGGCTGTCGCCTCCCAGCTGAGACAGGTCGATCCGTGTCTCGTACAGGCCGGTGATGCTCTGGTGGATCAGGGTGGCGTCCAGCACCTCTTTGGTGCTGGTGTACCTCTTCCGGTCGATGGTGGTGTCAAAGTACTTGAAGGCGGCAGGGGCTCCCAGATTGGTCAGGGTAAACAGGTGGATGATATTCTCGGCCTGCTCTCTGATGGGCTTATCCCGGTGCTTGTTGTAGGCGGACAGCACTTTGTCCAGATTAGCGTCGGCCAGGATCACTCTCTTGGAGAACTCGCTGA
Sanger sequencing data after PCR amplification and pJET cloning:
Clone 1: In13-527-ITR (-11bp,+14bp,-103bp)-Cas9rev (-2bp)
NNNattaagcaatgcaaggcacgtacgtttactatgtcattgcctatggctatgaagtgcaaatcctaacagtcctgctCCTGCAGGCAGttggccactccctctCTGCGCGCTCGCTCGCTCACTGAGGCCGCCCGGGCAAAGCCCGGGCGTCGGGCGACCTTTGGTCGCCCGGCCTCAGTGAGCGAGCGAGCGCGCAGAGAGGGAGTGGCCAACTCCATCACTAGGGGTTCCTGCGGCNNN
Clone 2: In13-527 (-38bp)-ITR (-21bp,+1bp)-Cas9rev
NNNattaagcaatgcaaggcacgtacgtttactatgtcattgcctatggctatgaagtgcaaatcctaacagtcctgctCCTGCAGGCAGCTGCGCGCTCtGCTCGCTCACTGAGGCCGCCCGGGCAAAGCCCGGGCGTCGGGCGACCTTTGGTCGCCCGGCCTCAGTGAGCGAGCGAGCGCGCAGAGAGGGAGTGGCCAACTCCATCACTAGGGGTTCCTGCGGCNNN
Clone 3: In13-527-ITR (-70bp,+1bp)-Cas9rev
NNNattaagcaatgcaaggcacgtacgtttactatgtcattgcctatggctatgaagtgcaaatcctaacagtcctgctCCTGCAGGCAGCTGCGCGCTCGCTCGCTCACTGAGGCCGCCCGGGCAAAGCCCGGGCGTCGGGCGACCTTaTGGTCGCCCGGCCTCAGTGAGCGAGCGAGCGCGCAGAGAGGGAGTGGCCAACTCCATCACTAGGGGTTCCTGCGGCNNN
Clone 4: In13-527(+7bp)-ITR (-74bp)-Cas9rev
NNNattaagcaatgcaaggcacgtacgtttactatgtcattgcctatggctatgaagtgcaaatcctaacagtcctgctCCTGCAGGCAGCTGCGCGCTCGCTCGCTCACTGAGGCCGCCCGGGCAAAGCCCGGGCGTCGGGCGACCTTTGGTttggccaCGCCCGGCCTCAGTGAGCGAGCGAGCGCGCAGAGAGGGAGTGGCCAACTCCATCACTAGGGGTTCCTGCGGCNNN
Clone 5: In13-527(+71bp)-ITR (-82bp)-Cas9rev
NNNattaagcaatgcaaggcacgtacgtttactatgtcattgcctatggctatgaagtgcaaatcctaacagtcctgctagtggccaactccatcactaggggttcctgcggcctctagaggtggtgcagatgaactccatggggtcctgCCTGCAGGCAGCTGCGCGCTCGCTCGCTCACTGAGGCCGCCCGGGCAAAGCCCGGGCGTCGGGCGACCTTTGGTCGCCCGGCCTCAGTGAGCGAGCGAGCGCGCAGAGAGGGAGTGGCCAACTCCATCACTAGGGGTTCCTGCGGCNNN
Clone 6: In13-527 (-27bp)-ITR (-89bp)-Cas9rev
NNNattaagcaatgcaaggcacgtacgtttactatgtcattgcctatggctatgaagtgcaaatcctaacagtcctgctCCTGCAGGCAGCTGCGCGCTCGCTCGCTCACTGAGGCCGCCCGGGCAAAGCCCGGGCGTCGGGCGACCTTTGGTCGCCCGGCCTCAGTGAGCGAGCGAGCGCGCAGAGAGGGAGTGGCCAACTCCATCACTAGGGGTTCCTGCGGCNNN
Clone 7: In13-527-ITR (-100bp)-Cas9rev
NNNattaagcaatgcaaggcacgtacgtttactatgtcattgcctatggctatgaagtgcaaatcctaacagtcctgctCCTGCAGGCAGCTGCGCGCTCGCTCGCTCACTGAGGCCGCCCGGGCAAAGCCCGGGCGTCGGGCGACCTTTGGTCGCCCGGCCTCAGTGAGCGAGCGAGCGCGCAGAGAGGGAGTGGCCAACTCCATCACTAGGGGTTCCTGCGGCNNN
Clone 8: In13-527 (-24bp)-ITR (-113bp)-Cas9rev
NNNattaagcaatgcaaggcacgtacgtttactatgtcattgcctatggctatgaagtgcaaatcctaacagtcctgctCCTGCAGGCAGCTGCGCGCTCGCTCGCTCACTGAGGCCGCCCGGGCAAAGCCCGGGCGTCGGGCGACCTTTGGTCGCCCGGCCTCAGTGAGCGAGCGAGCGCGCAGAGAGGGAGTGGCCAACTCCATCACTAGGGGTTCCTGCGGCNNN
Clone 9: In13-527-ITR (-129bp)-Cas9rev
NNNattaagcaatgcaaggcacgtacgtttactatgtcattgcctatggctatgaagtgcaaatcctaacagtcctgctCCTGCAGGCAGCTGCGCGCTCGCTCGCTCACTGAGGCCGCCCGGGCAAAGCCCGGGCGTCGGGCGACCTTTGGTCGCCCGGCCTCAGTGAGCGAGCGAGCGCGCAGAGAGGGAGTGGCCAACTCCATCACTAGGGGTTCCTGCGGCNNN
Clone 10: In13-527 (-76bp)- -ITR (-133bp)-Cas9rev
NNNattaagcaatgcaaggcacgtacgtttactatgtcattgcctatggctatgaagtgcaaatcctaacagtcctgctCCTGCAGGCAGCTGCGCGCTCGCTCGCTCACTGAGGCCGCCCGGGCAAAGCCCGGGCGTCGGGCGACCTTTGGTCGCCCGGCCTCAGTGAGCGAGCGAGCGCGCAGAGAGGGAGTGGCCAACTCCATCACTAGGGGTTCCTGCGGCNNN
Fig. S13O. Analysis of left junction of reverse insertion of Cas9 at Alb-Intron13-527. Double strikethrough indicates deleted nucleotides (-) and red lowercase indicates inserted nucleotides (+).

## Slide 39
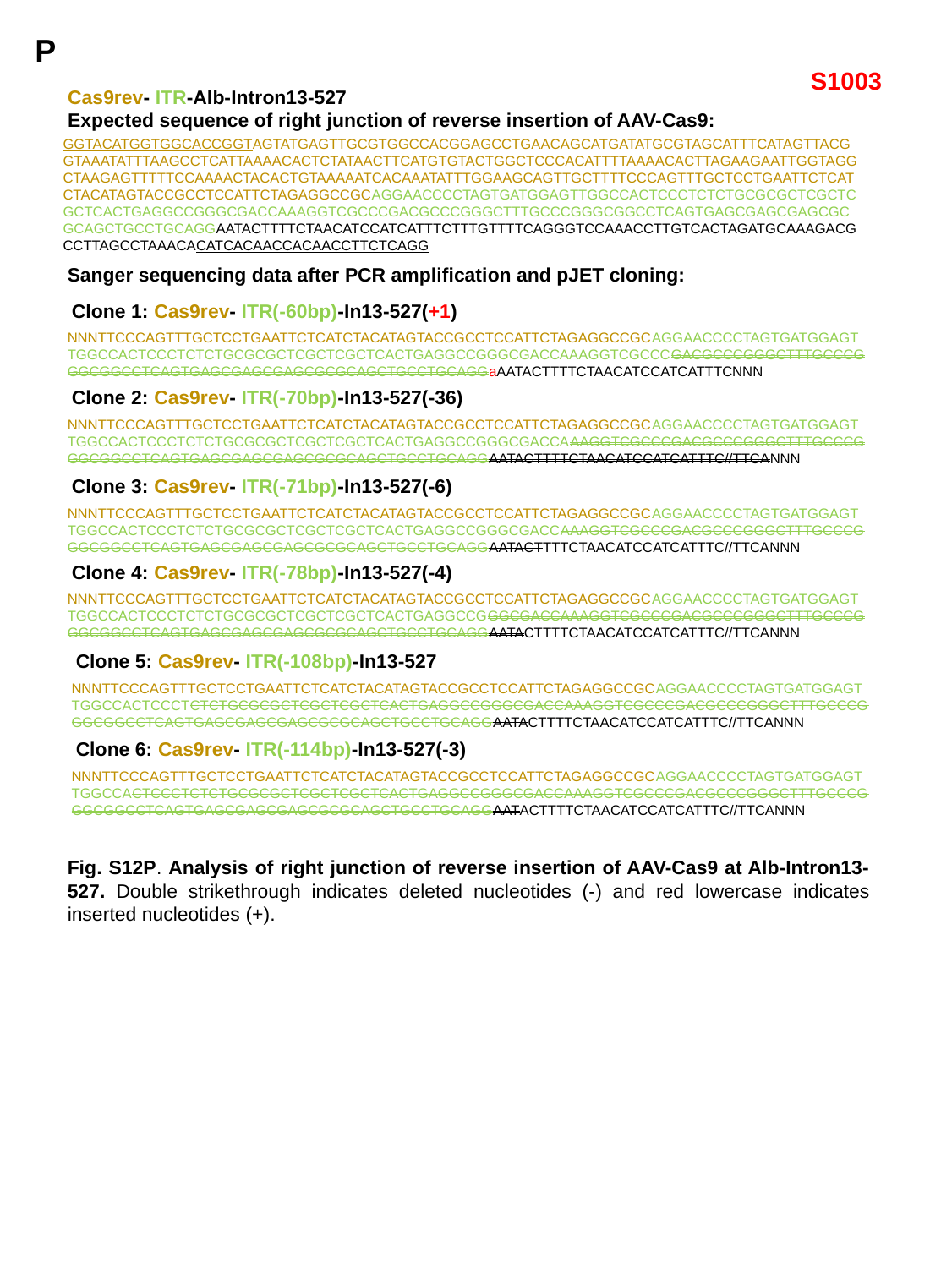

P
S1003
Cas9rev- ITR-Alb-Intron13-527
Expected sequence of right junction of reverse insertion of AAV-Cas9:
GGTACATGGTGGCACCGGTAGTATGAGTTGCGTGGCCACGGAGCCTGAACAGCATGATATGCGTAGCATTTCATAGTTACGGTAAATATTTAAGCCTCATTAAAACACTCTATAACTTCATGTGTACTGGCTCCCACATTTTAAAACACTTAGAAGAATTGGTAGGCTAAGAGTTTTTCCAAAACTACACTGTAAAAATCACAAATATTTGGAAGCAGTTGCTTTTCCCAGTTTGCTCCTGAATTCTCATCTACATAGTACCGCCTCCATTCTAGAGGCCGCAGGAACCCCTAGTGATGGAGTTGGCCACTCCCTCTCTGCGCGCTCGCTCGCTCACTGAGGCCGGGCGACCAAAGGTCGCCCGACGCCCGGGCTTTGCCCGGGCGGCCTCAGTGAGCGAGCGAGCGCGCAGCTGCCTGCAGGaatacttttctaacatccatcatttctttgttttcagggtccaaaccttgtcactagatgcaaagacgccttagcctaaacacatcacaaccacaaccttctcagg
Sanger sequencing data after PCR amplification and pJET cloning:
Clone 1: Cas9rev- ITR(-60bp)-In13-527(+1)
NNNTTCCCAGTTTGCTCCTGAATTCTCATCTACATAGTACCGCCTCCATTCTAGAGGCCGCAGGAACCCCTAGTGATGGAGTTGGCCACTCCCTCTCTGCGCGCTCGCTCGCTCACTGAGGCCGGGCGACCAAAGGTCGCCCGACGCCCGGGCTTTGCCCGGGCGGCCTCAGTGAGCGAGCGAGCGCGCAGCTGCCTGCAGGaaatacttttctaacatccatcatttcNNN
Clone 2: Cas9rev- ITR(-70bp)-In13-527(-36)
NNNTTCCCAGTTTGCTCCTGAATTCTCATCTACATAGTACCGCCTCCATTCTAGAGGCCGCAGGAACCCCTAGTGATGGAGTTGGCCACTCCCTCTCTGCGCGCTCGCTCGCTCACTGAGGCCGGGCGACCAAAGGTCGCCCGACGCCCGGGCTTTGCCCGGGCGGCCTCAGTGAGCGAGCGAGCGCGCAGCTGCCTGCAGGaatacttttctaacatccatcatttc//TTCANNN
Clone 3: Cas9rev- ITR(-71bp)-In13-527(-6)
NNNTTCCCAGTTTGCTCCTGAATTCTCATCTACATAGTACCGCCTCCATTCTAGAGGCCGCAGGAACCCCTAGTGATGGAGTTGGCCACTCCCTCTCTGCGCGCTCGCTCGCTCACTGAGGCCGGGCGACCAAAGGTCGCCCGACGCCCGGGCTTTGCCCGGGCGGCCTCAGTGAGCGAGCGAGCGCGCAGCTGCCTGCAGGaatacttttctaacatccatcatttc//TTCANNN
Clone 4: Cas9rev- ITR(-78bp)-In13-527(-4)
NNNTTCCCAGTTTGCTCCTGAATTCTCATCTACATAGTACCGCCTCCATTCTAGAGGCCGCAGGAACCCCTAGTGATGGAGTTGGCCACTCCCTCTCTGCGCGCTCGCTCGCTCACTGAGGCCGGGCGACCAAAGGTCGCCCGACGCCCGGGCTTTGCCCGGGCGGCCTCAGTGAGCGAGCGAGCGCGCAGCTGCCTGCAGGaatacttttctaacatccatcatttc//TTCANNN
Clone 5: Cas9rev- ITR(-108bp)-In13-527
NNNTTCCCAGTTTGCTCCTGAATTCTCATCTACATAGTACCGCCTCCATTCTAGAGGCCGCAGGAACCCCTAGTGATGGAGTTGGCCACTCCCTCTCTGCGCGCTCGCTCGCTCACTGAGGCCGGGCGACCAAAGGTCGCCCGACGCCCGGGCTTTGCCCGGGCGGCCTCAGTGAGCGAGCGAGCGCGCAGCTGCCTGCAGGaatacttttctaacatccatcatttc//TTCANNN
Clone 6: Cas9rev- ITR(-114bp)-In13-527(-3)
NNNTTCCCAGTTTGCTCCTGAATTCTCATCTACATAGTACCGCCTCCATTCTAGAGGCCGCAGGAACCCCTAGTGATGGAGTTGGCCACTCCCTCTCTGCGCGCTCGCTCGCTCACTGAGGCCGGGCGACCAAAGGTCGCCCGACGCCCGGGCTTTGCCCGGGCGGCCTCAGTGAGCGAGCGAGCGCGCAGCTGCCTGCAGGaatacttttctaacatccatcatttc//TTCANNN
Fig. S12P. Analysis of right junction of reverse insertion of AAV-Cas9 at Alb-Intron13-527. Double strikethrough indicates deleted nucleotides (-) and red lowercase indicates inserted nucleotides (+).

## Slide 40
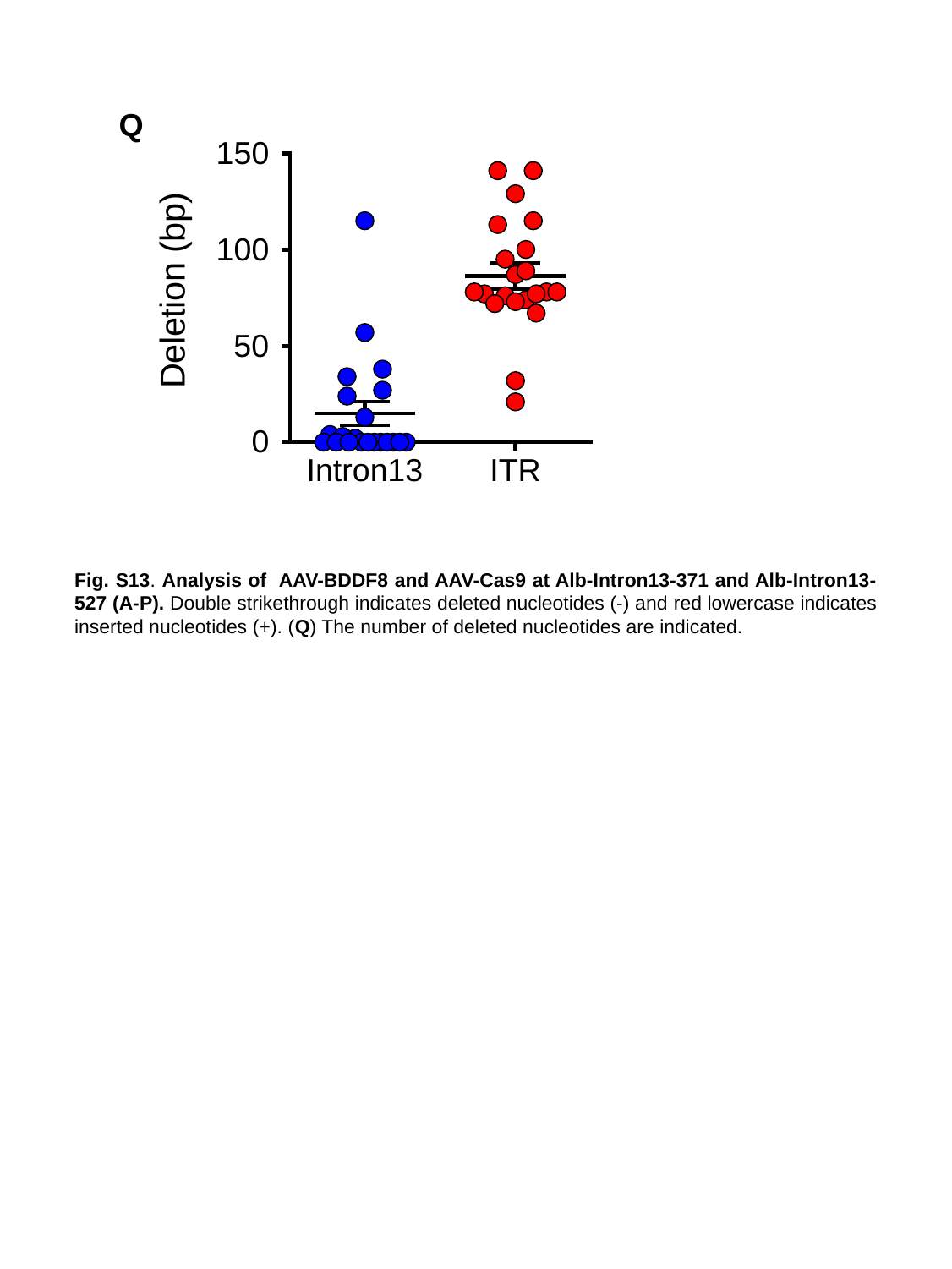

Q
Fig. S13. Analysis of AAV-BDDF8 and AAV-Cas9 at Alb-Intron13-371 and Alb-Intron13-527 (A-P). Double strikethrough indicates deleted nucleotides (-) and red lowercase indicates inserted nucleotides (+). (Q) The number of deleted nucleotides are indicated.

## Slide 41
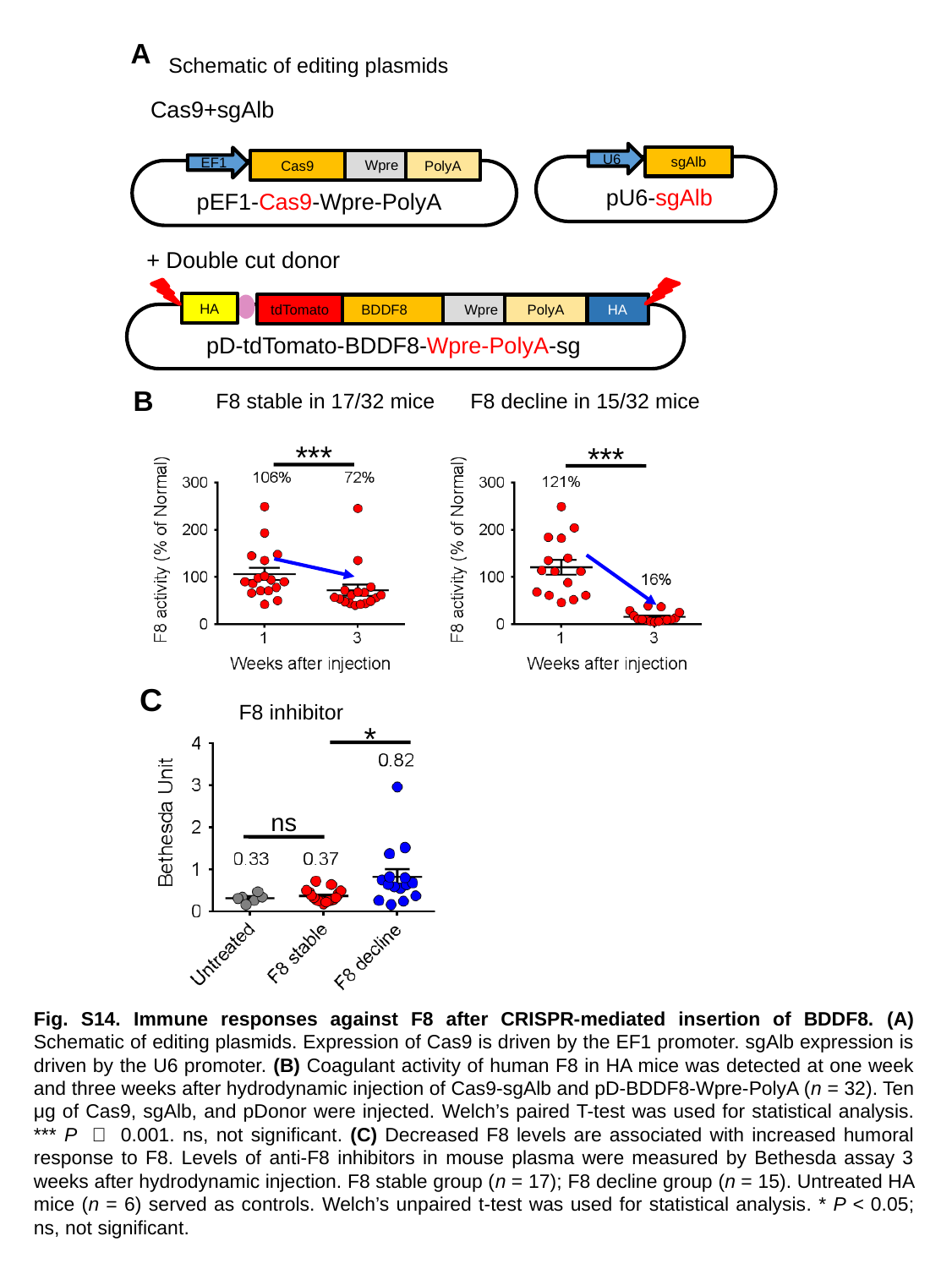

A
Schematic of editing plasmids
Cas9+sgAlb
U6
sgAlb
pU6-sgAlb
EF1
Wpre
PolyA
Cas9
pEF1-Cas9-Wpre-PolyA
+ Double cut donor
HA
Wpre
PolyA
BDDF8
HA
pD-tdTomato-BDDF8-Wpre-PolyA-sg
tdTomato
B
F8 stable in 17/32 mice
F8 decline in 15/32 mice
***
***
C
F8 inhibitor
*
ns
Fig. S14. Immune responses against F8 after CRISPR-mediated insertion of BDDF8. (A) Schematic of editing plasmids. Expression of Cas9 is driven by the EF1 promoter. sgAlb expression is driven by the U6 promoter. (B) Coagulant activity of human F8 in HA mice was detected at one week and three weeks after hydrodynamic injection of Cas9-sgAlb and pD-BDDF8-Wpre-PolyA (n = 32). Ten μg of Cas9, sgAlb, and pDonor were injected. Welch’s paired T-test was used for statistical analysis. *** P ＜ 0.001. ns, not significant. (C) Decreased F8 levels are associated with increased humoral response to F8. Levels of anti-F8 inhibitors in mouse plasma were measured by Bethesda assay 3 weeks after hydrodynamic injection. F8 stable group (n = 17); F8 decline group (n = 15). Untreated HA mice (n = 6) served as controls. Welch’s unpaired t-test was used for statistical analysis. * P < 0.05; ns, not significant.

## Slide 42
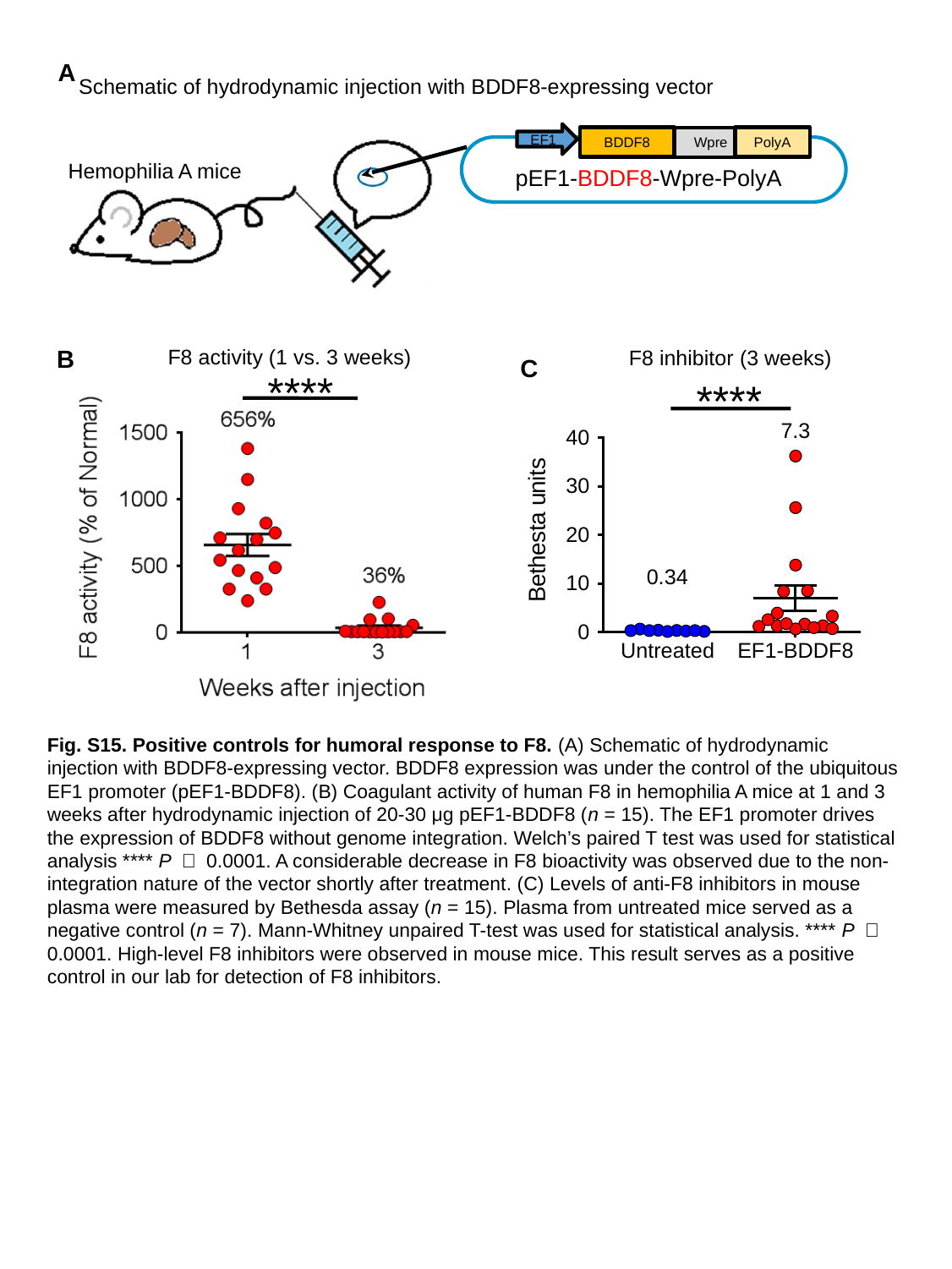

A
Schematic of hydrodynamic injection with BDDF8-expressing vector
EF1
PolyA
BDDF8
Wpre
pEF1-BDDF8-Wpre-PolyA
Hemophilia A mice
F8 activity (1 vs. 3 weeks)
B
F8 inhibitor (3 weeks)
C
****
****
Fig. S15. Positive controls for humoral response to F8. (A) Schematic of hydrodynamic injection with BDDF8-expressing vector. BDDF8 expression was under the control of the ubiquitous EF1 promoter (pEF1-BDDF8). (B) Coagulant activity of human F8 in hemophilia A mice at 1 and 3 weeks after hydrodynamic injection of 20-30 μg pEF1-BDDF8 (n = 15). The EF1 promoter drives the expression of BDDF8 without genome integration. Welch’s paired T test was used for statistical analysis **** P ＜ 0.0001. A considerable decrease in F8 bioactivity was observed due to the non-integration nature of the vector shortly after treatment. (C) Levels of anti-F8 inhibitors in mouse plasma were measured by Bethesda assay (n = 15). Plasma from untreated mice served as a negative control (n = 7). Mann-Whitney unpaired T-test was used for statistical analysis. **** P ＜ 0.0001. High-level F8 inhibitors were observed in mouse mice. This result serves as a positive control in our lab for detection of F8 inhibitors.

## Slide 43
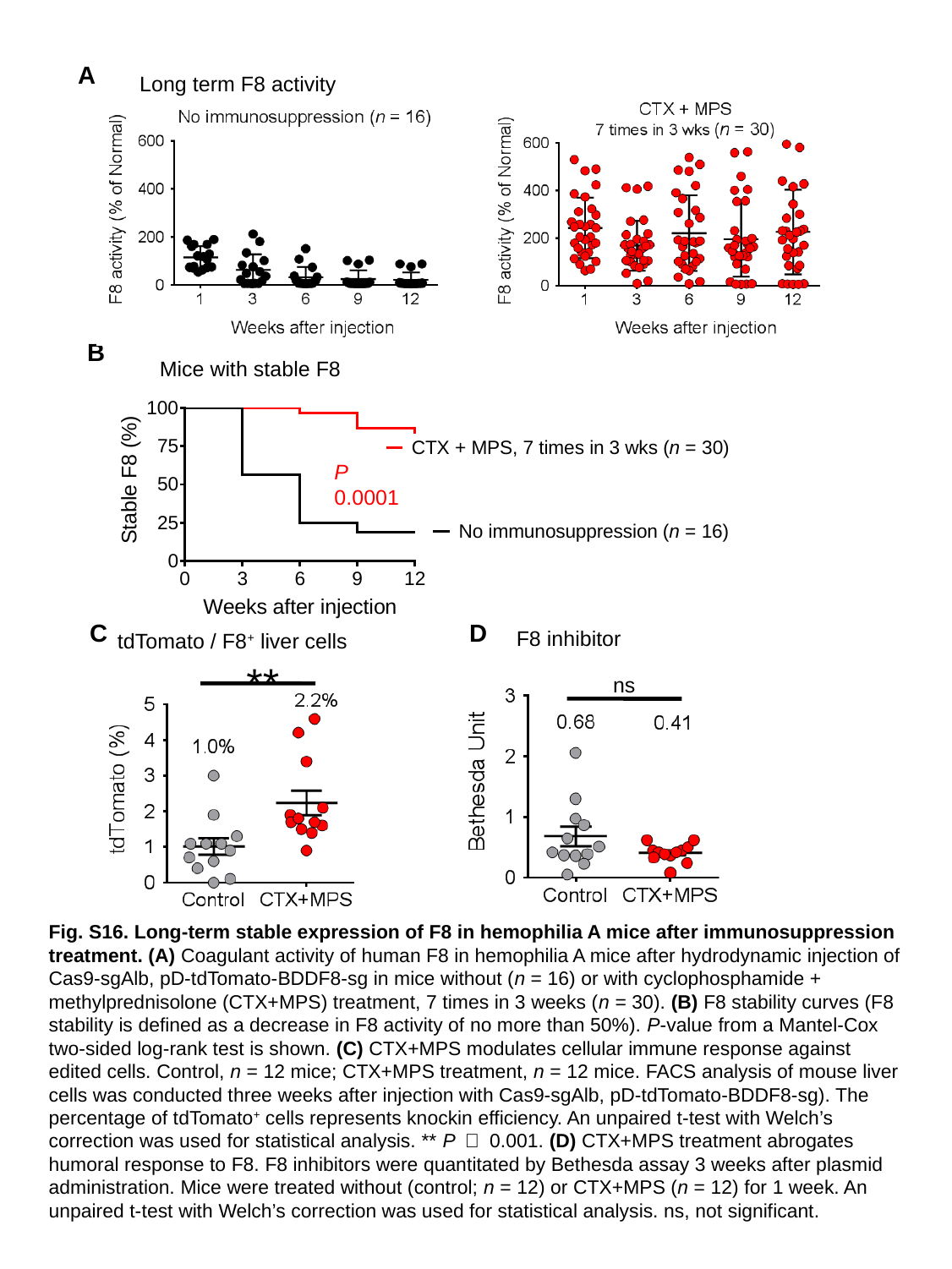

A
Long term F8 activity
B
Mice with stable F8
P ＜ 0.0001
C
tdTomato / F8+ liver cells
D
F8 inhibitor
ns
**
Fig. S16. Long-term stable expression of F8 in hemophilia A mice after immunosuppression treatment. (A) Coagulant activity of human F8 in hemophilia A mice after hydrodynamic injection of Cas9-sgAlb, pD-tdTomato-BDDF8-sg in mice without (n = 16) or with cyclophosphamide + methylprednisolone (CTX+MPS) treatment, 7 times in 3 weeks (n = 30). (B) F8 stability curves (F8 stability is defined as a decrease in F8 activity of no more than 50%). P-value from a Mantel-Cox two-sided log-rank test is shown. (C) CTX+MPS modulates cellular immune response against edited cells. Control, n = 12 mice; CTX+MPS treatment, n = 12 mice. FACS analysis of mouse liver cells was conducted three weeks after injection with Cas9-sgAlb, pD-tdTomato-BDDF8-sg). The percentage of tdTomato+ cells represents knockin efficiency. An unpaired t-test with Welch’s correction was used for statistical analysis. ** P ＜ 0.001. (D) CTX+MPS treatment abrogates humoral response to F8. F8 inhibitors were quantitated by Bethesda assay 3 weeks after plasmid administration. Mice were treated without (control; n = 12) or CTX+MPS (n = 12) for 1 week. An unpaired t-test with Welch’s correction was used for statistical analysis. ns, not significant.

## Slide 44
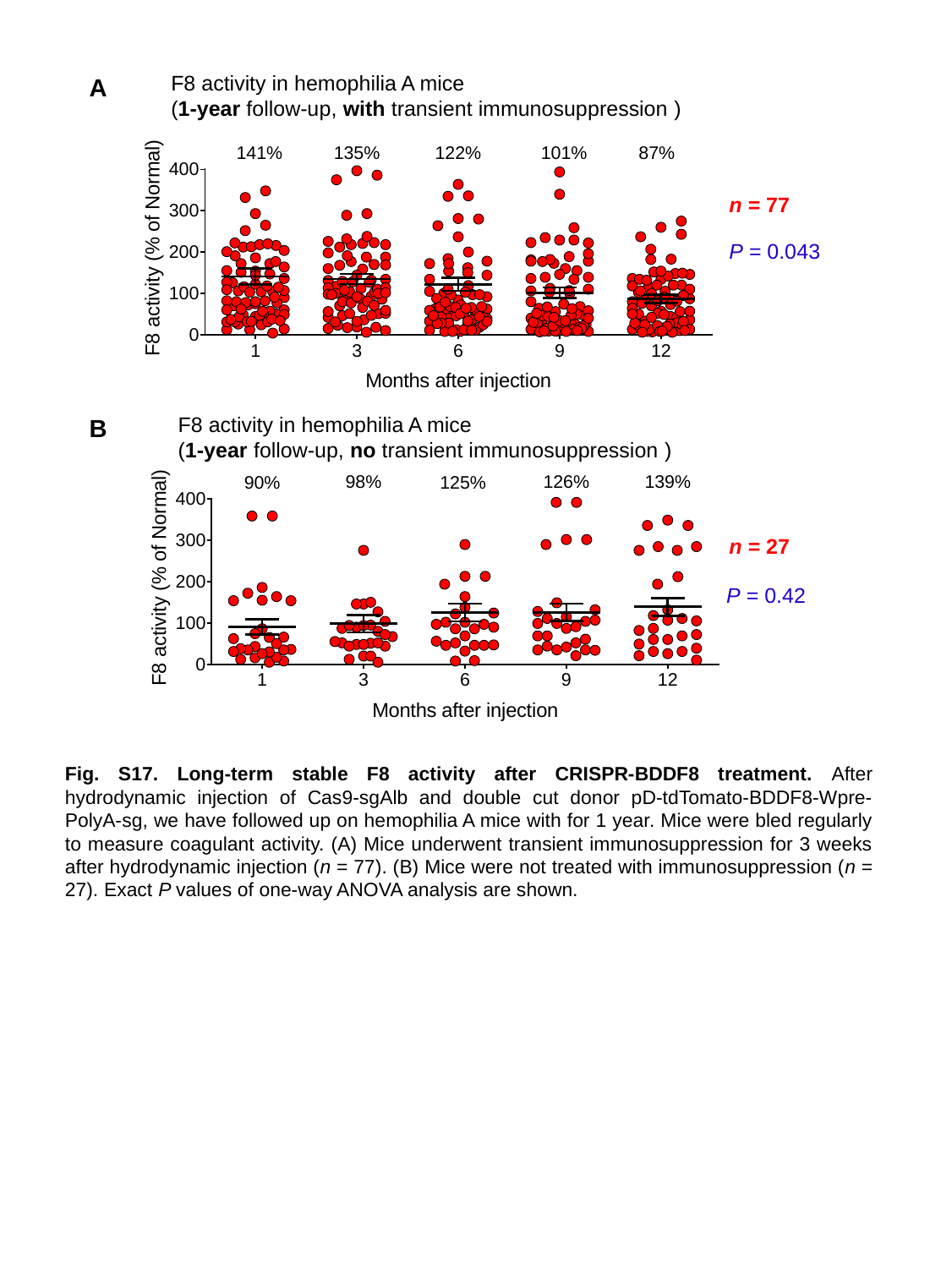

F8 activity in hemophilia A mice
(1-year follow-up, with transient immunosuppression )
A
n = 77
P = 0.043
F8 activity in hemophilia A mice
(1-year follow-up, no transient immunosuppression )
B
n = 27
P = 0.42
Fig. S17. Long-term stable F8 activity after CRISPR-BDDF8 treatment. After hydrodynamic injection of Cas9-sgAlb and double cut donor pD-tdTomato-BDDF8-Wpre-PolyA-sg, we have followed up on hemophilia A mice with for 1 year. Mice were bled regularly to measure coagulant activity. (A) Mice underwent transient immunosuppression for 3 weeks after hydrodynamic injection (n = 77). (B) Mice were not treated with immunosuppression (n = 27). Exact P values of one-way ANOVA analysis are shown.

## Slide 45
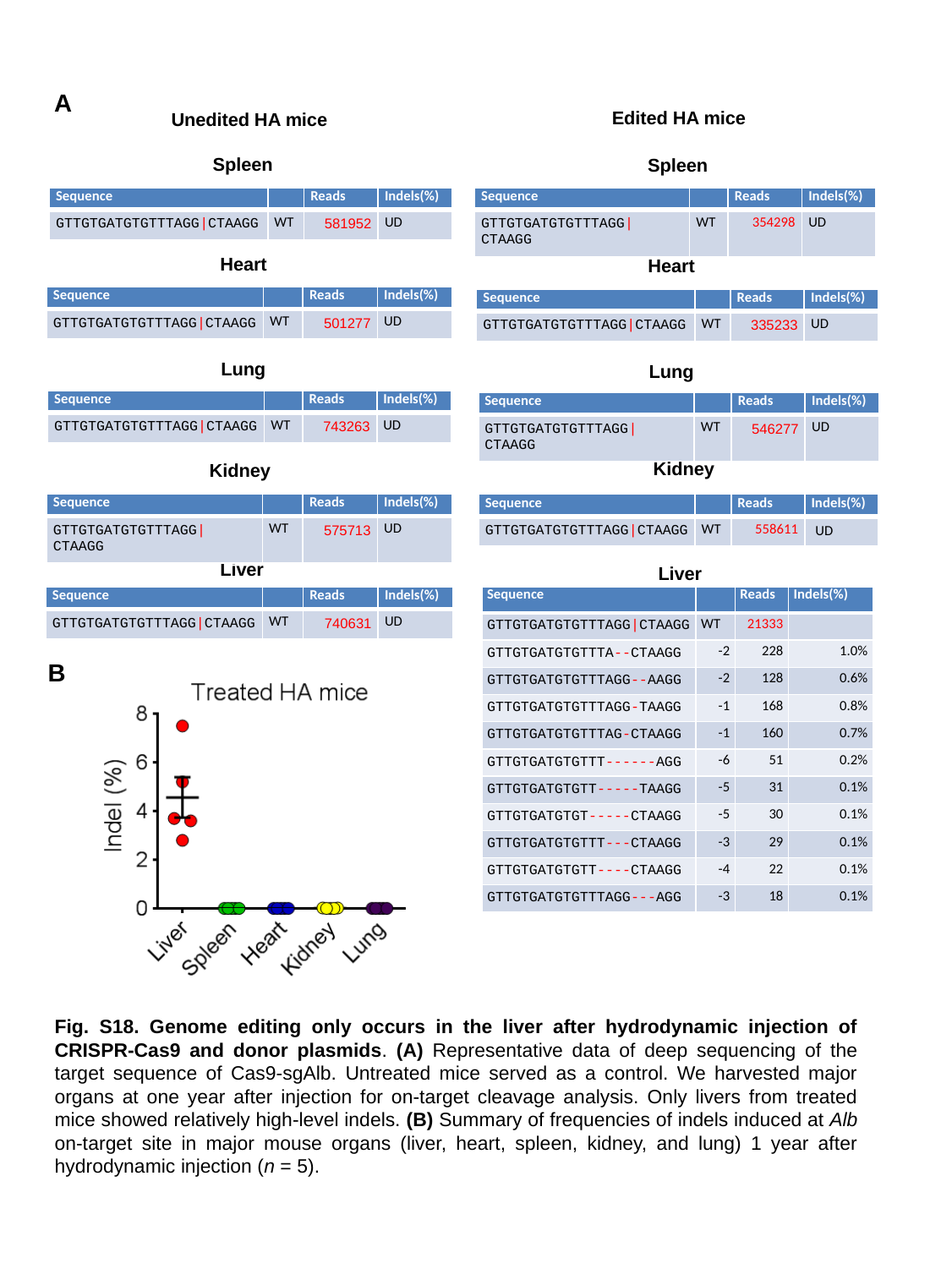

A
Edited HA mice
Unedited HA mice
Spleen
Spleen
| Sequence | | Reads | Indels(%) |
| --- | --- | --- | --- |
| GTTGTGATGTGTTTAGG|CTAAGG | WT | 581952 | UD |
| Sequence | | Reads | Indels(%) |
| --- | --- | --- | --- |
| GTTGTGATGTGTTTAGG|CTAAGG | WT | 354298 | UD |
Heart
Heart
| Sequence | | Reads | Indels(%) |
| --- | --- | --- | --- |
| GTTGTGATGTGTTTAGG|CTAAGG | WT | 501277 | UD |
| Sequence | | Reads | Indels(%) |
| --- | --- | --- | --- |
| GTTGTGATGTGTTTAGG|CTAAGG | WT | 335233 | UD |
Lung
Lung
| Sequence | | Reads | Indels(%) |
| --- | --- | --- | --- |
| GTTGTGATGTGTTTAGG|CTAAGG | WT | 743263 | UD |
| Sequence | | Reads | Indels(%) |
| --- | --- | --- | --- |
| GTTGTGATGTGTTTAGG|CTAAGG | WT | 546277 | UD |
Kidney
Kidney
| Sequence | | Reads | Indels(%) |
| --- | --- | --- | --- |
| GTTGTGATGTGTTTAGG|CTAAGG | WT | 575713 | UD |
| Sequence | | Reads | Indels(%) |
| --- | --- | --- | --- |
| GTTGTGATGTGTTTAGG|CTAAGG | WT | 558611 | UD |
Liver
Liver
| Sequence | | Reads | Indels(%) |
| --- | --- | --- | --- |
| GTTGTGATGTGTTTAGG|CTAAGG | WT | 21333 | |
| GTTGTGATGTGTTTA--CTAAGG | -2 | 228 | 1.0% |
| GTTGTGATGTGTTTAGG--AAGG | -2 | 128 | 0.6% |
| GTTGTGATGTGTTTAGG-TAAGG | -1 | 168 | 0.8% |
| GTTGTGATGTGTTTAG-CTAAGG | -1 | 160 | 0.7% |
| GTTGTGATGTGTTT------AGG | -6 | 51 | 0.2% |
| GTTGTGATGTGTT-----TAAGG | -5 | 31 | 0.1% |
| GTTGTGATGTGT-----CTAAGG | -5 | 30 | 0.1% |
| GTTGTGATGTGTTT---CTAAGG | -3 | 29 | 0.1% |
| GTTGTGATGTGTT----CTAAGG | -4 | 22 | 0.1% |
| GTTGTGATGTGTTTAGG---AGG | -3 | 18 | 0.1% |
| Sequence | | Reads | Indels(%) |
| --- | --- | --- | --- |
| GTTGTGATGTGTTTAGG|CTAAGG | WT | 740631 | UD |
B
Fig. S18. Genome editing only occurs in the liver after hydrodynamic injection of CRISPR-Cas9 and donor plasmids. (A) Representative data of deep sequencing of the target sequence of Cas9-sgAlb. Untreated mice served as a control. We harvested major organs at one year after injection for on-target cleavage analysis. Only livers from treated mice showed relatively high-level indels. (B) Summary of frequencies of indels induced at Alb on-target site in major mouse organs (liver, heart, spleen, kidney, and lung) 1 year after hydrodynamic injection (n = 5).

## Slide 46
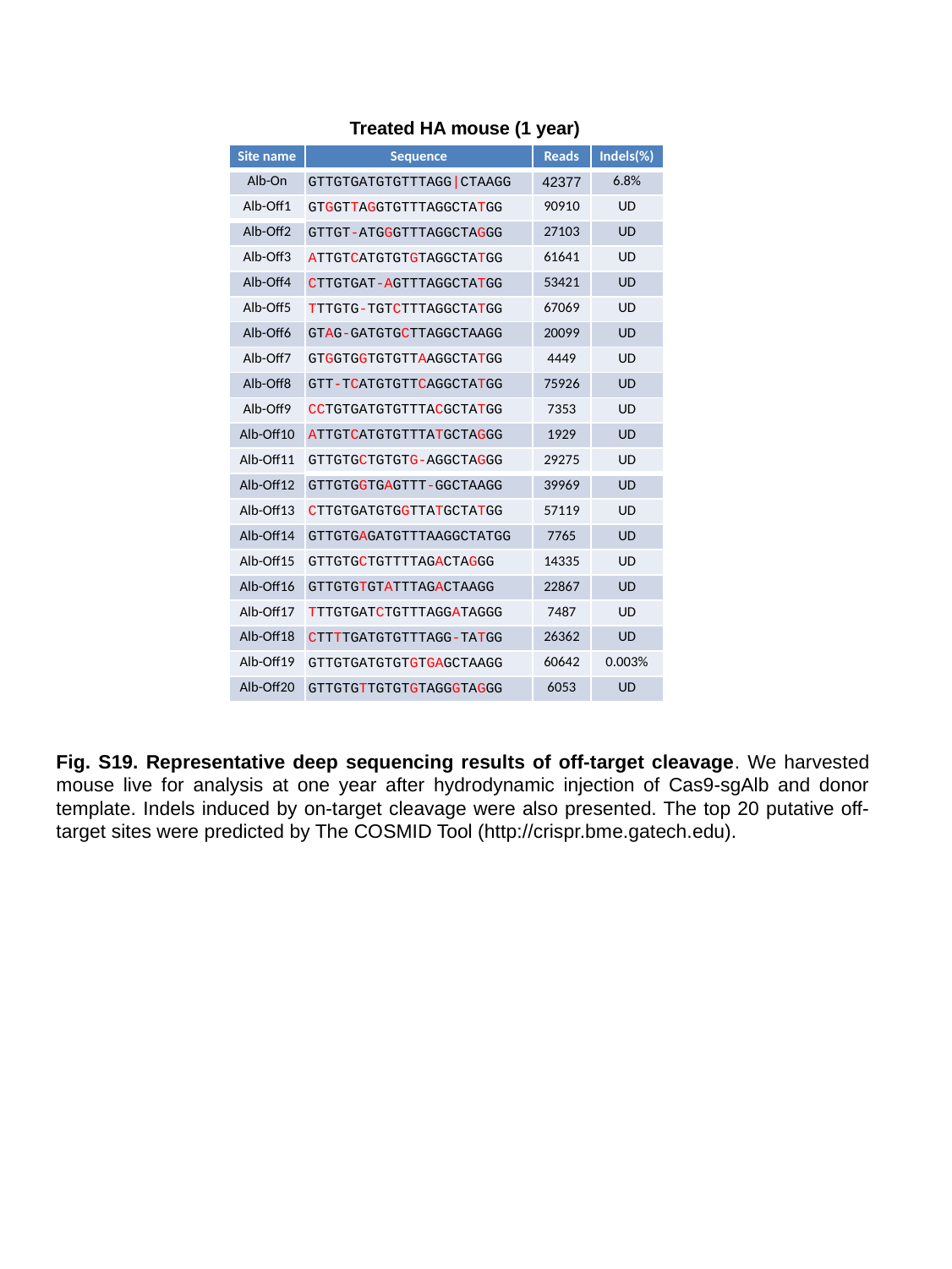

Treated HA mouse (1 year)
| Site name | Sequence | Reads | Indels(%) |
| --- | --- | --- | --- |
| Alb-On | GTTGTGATGTGTTTAGG|CTAAGG | 42377 | 6.8% |
| Alb-Off1 | GTGGTTAGGTGTTTAGGCTATGG | 90910 | UD |
| Alb-Off2 | GTTGT-ATGGGTTTAGGCTAGGG | 27103 | UD |
| Alb-Off3 | ATTGTCATGTGTGTAGGCTATGG | 61641 | UD |
| Alb-Off4 | CTTGTGAT-AGTTTAGGCTATGG | 53421 | UD |
| Alb-Off5 | TTTGTG-TGTCTTTAGGCTATGG | 67069 | UD |
| Alb-Off6 | GTAG-GATGTGCTTAGGCTAAGG | 20099 | UD |
| Alb-Off7 | GTGGTGGTGTGTTAAGGCTATGG | 4449 | UD |
| Alb-Off8 | GTT-TCATGTGTTCAGGCTATGG | 75926 | UD |
| Alb-Off9 | CCTGTGATGTGTTTACGCTATGG | 7353 | UD |
| Alb-Off10 | ATTGTCATGTGTTTATGCTAGGG | 1929 | UD |
| Alb-Off11 | GTTGTGCTGTGTG-AGGCTAGGG | 29275 | UD |
| Alb-Off12 | GTTGTGGTGAGTTT-GGCTAAGG | 39969 | UD |
| Alb-Off13 | CTTGTGATGTGGTTATGCTATGG | 57119 | UD |
| Alb-Off14 | GTTGTGAGATGTTTAAGGCTATGG | 7765 | UD |
| Alb-Off15 | GTTGTGCTGTTTTAGACTAGGG | 14335 | UD |
| Alb-Off16 | GTTGTGTGTATTTAGACTAAGG | 22867 | UD |
| Alb-Off17 | TTTGTGATCTGTTTAGGATAGGG | 7487 | UD |
| Alb-Off18 | CTTTTGATGTGTTTAGG-TATGG | 26362 | UD |
| Alb-Off19 | GTTGTGATGTGTGTGAGCTAAGG | 60642 | 0.003% |
| Alb-Off20 | GTTGTGTTGTGTGTAGGGTAGGG | 6053 | UD |
Fig. S19. Representative deep sequencing results of off-target cleavage. We harvested mouse live for analysis at one year after hydrodynamic injection of Cas9-sgAlb and donor template. Indels induced by on-target cleavage were also presented. The top 20 putative off-target sites were predicted by The COSMID Tool (http://crispr.bme.gatech.edu).

## Slide 47
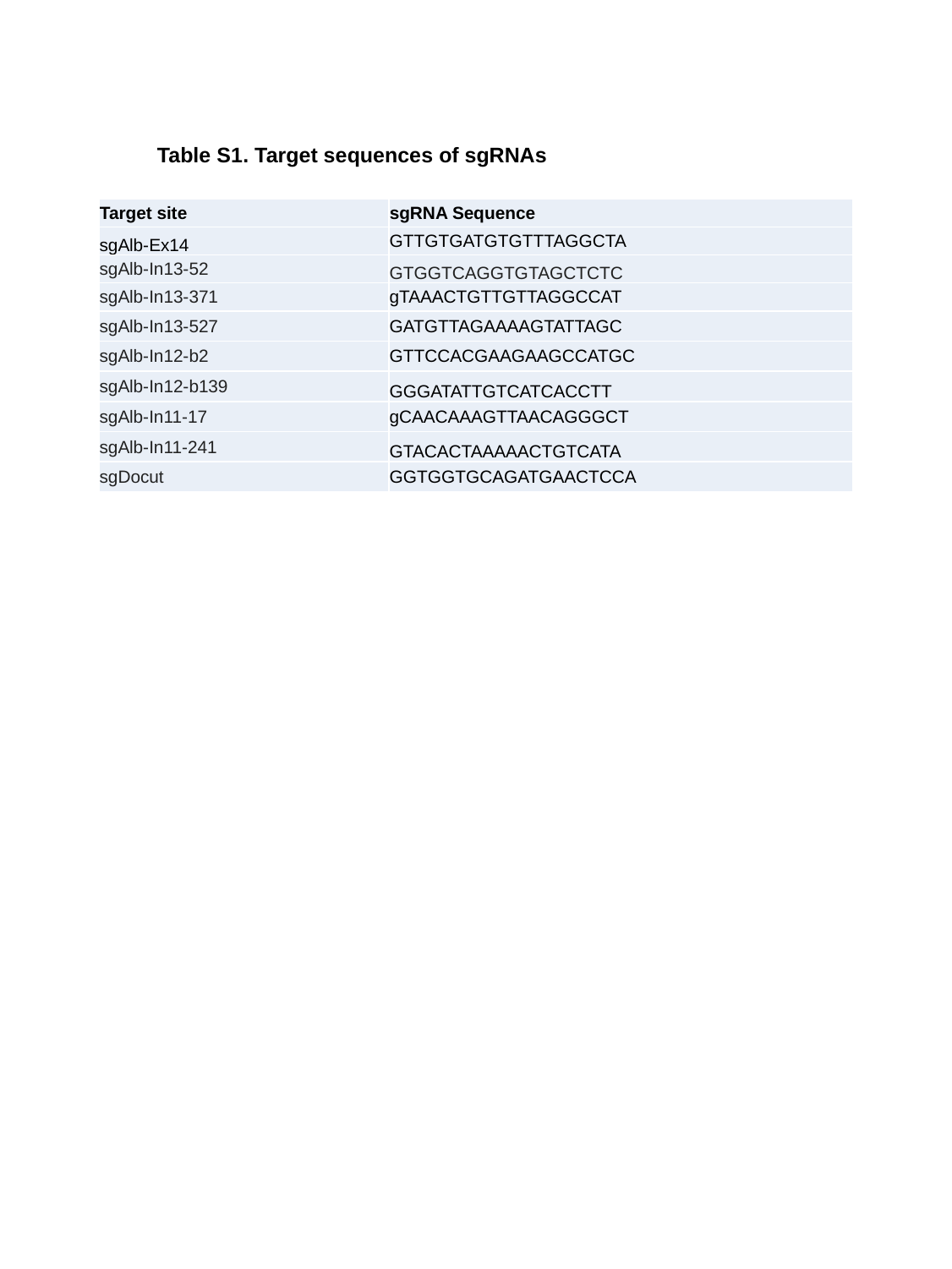

Table S1. Target sequences of sgRNAs
| Target site | sgRNA Sequence |
| --- | --- |
| sgAlb-Ex14 | GTTGTGATGTGTTTAGGCTA |
| sgAlb-In13-52 | GTGGTCAGGTGTAGCTCTC |
| sgAlb-In13-371 | gTAAACTGTTGTTAGGCCAT |
| sgAlb-In13-527 | GATGTTAGAAAAGTATTAGC |
| sgAlb-In12-b2 | GTTCCACGAAGAAGCCATGC |
| sgAlb-In12-b139 | GGGATATTGTCATCACCTT |
| sgAlb-In11-17 | gCAACAAAGTTAACAGGGCT |
| sgAlb-In11-241 | GTACACTAAAAACTGTCATA |
| sgDocut | GGTGGTGCAGATGAACTCCA |

## Slide 48
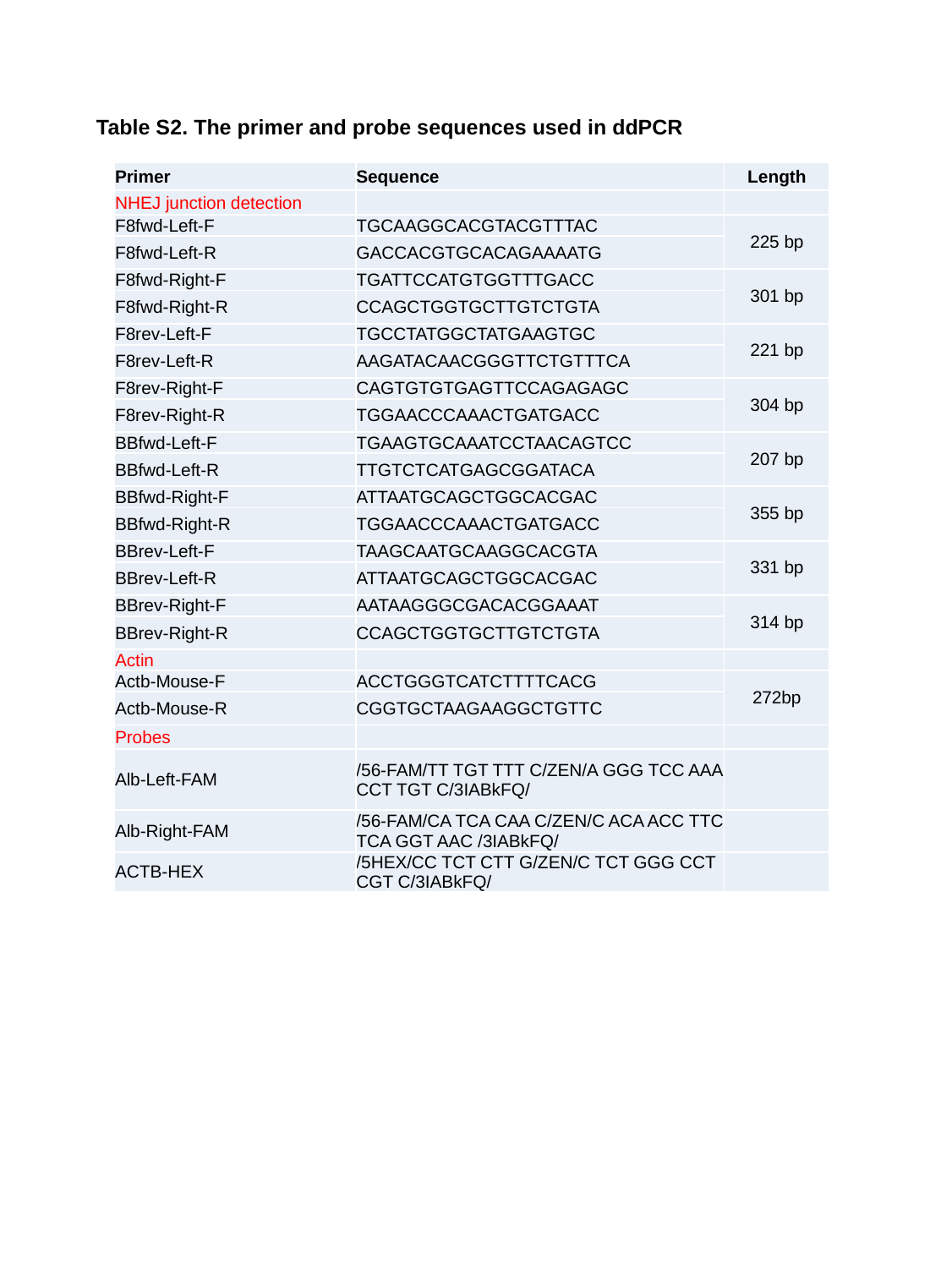

Table S2. The primer and probe sequences used in ddPCR
| Primer | Sequence | Length |
| --- | --- | --- |
| NHEJ junction detection | | |
| F8fwd-Left-F | TGCAAGGCACGTACGTTTAC | 225 bp |
| F8fwd-Left-R | GACCACGTGCACAGAAAATG | |
| F8fwd-Right-F | TGATTCCATGTGGTTTGACC | 301 bp |
| F8fwd-Right-R | CCAGCTGGTGCTTGTCTGTA | |
| F8rev-Left-F | TGCCTATGGCTATGAAGTGC | 221 bp |
| F8rev-Left-R | AAGATACAACGGGTTCTGTTTCA | |
| F8rev-Right-F | CAGTGTGTGAGTTCCAGAGAGC | 304 bp |
| F8rev-Right-R | TGGAACCCAAACTGATGACC | |
| BBfwd-Left-F | TGAAGTGCAAATCCTAACAGTCC | 207 bp |
| BBfwd-Left-R | TTGTCTCATGAGCGGATACA | |
| BBfwd-Right-F | ATTAATGCAGCTGGCACGAC | 355 bp |
| BBfwd-Right-R | TGGAACCCAAACTGATGACC | |
| BBrev-Left-F | TAAGCAATGCAAGGCACGTA | 331 bp |
| BBrev-Left-R | ATTAATGCAGCTGGCACGAC | |
| BBrev-Right-F | AATAAGGGCGACACGGAAAT | 314 bp |
| BBrev-Right-R | CCAGCTGGTGCTTGTCTGTA | |
| Actin | | |
| Actb-Mouse-F | ACCTGGGTCATCTTTTCACG | 272bp |
| Actb-Mouse-R | CGGTGCTAAGAAGGCTGTTC | |
| Probes | | |
| Alb-Left-FAM | /56-FAM/TT TGT TTT C/ZEN/A GGG TCC AAA CCT TGT C/3IABkFQ/ | |
| Alb-Right-FAM | /56-FAM/CA TCA CAA C/ZEN/C ACA ACC TTC TCA GGT AAC /3IABkFQ/ | |
| ACTB-HEX | /5HEX/CC TCT CTT G/ZEN/C TCT GGG CCT CGT C/3IABkFQ/ | |

## Slide 49
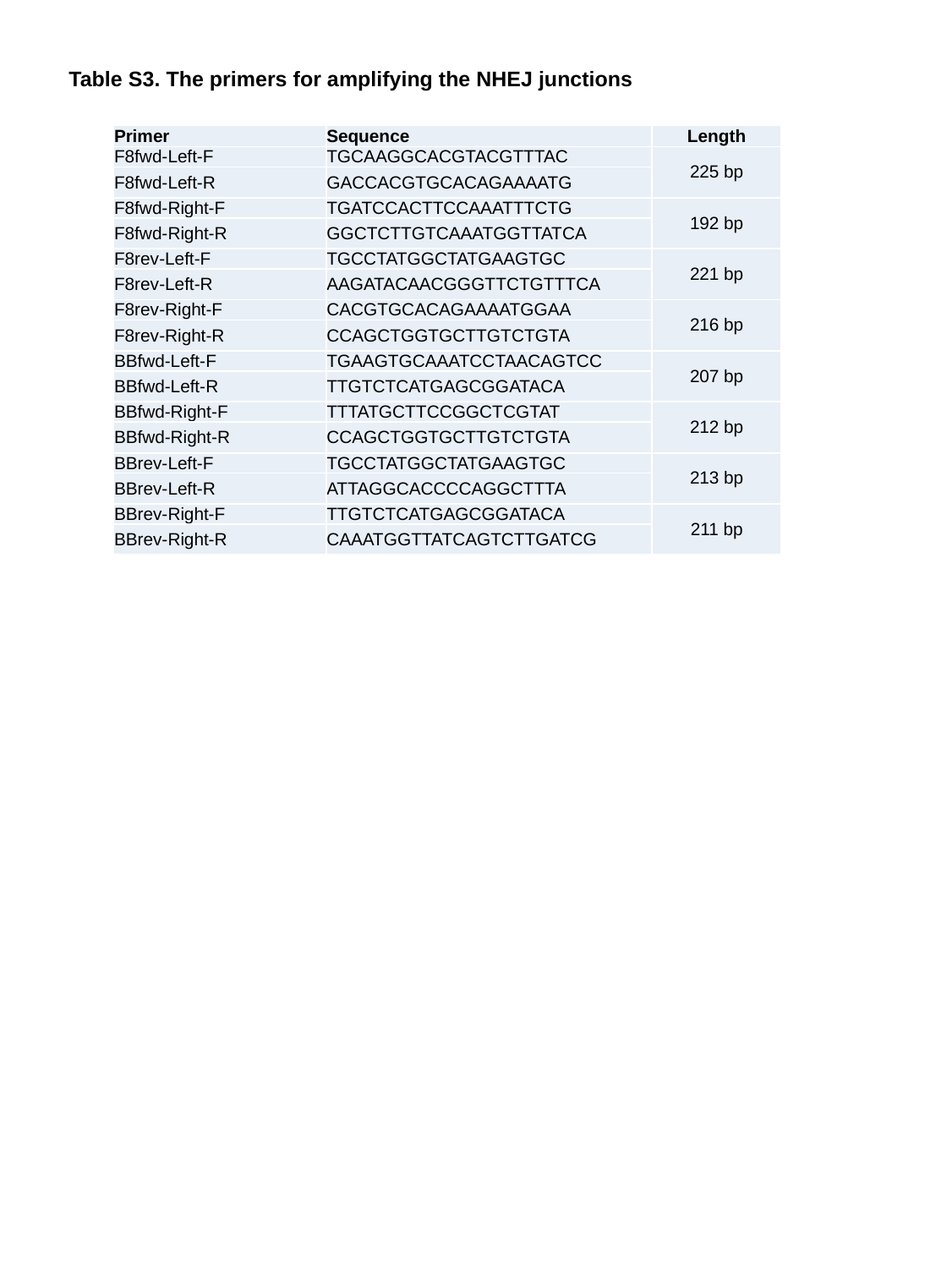

Table S3. The primers for amplifying the NHEJ junctions
| Primer | Sequence | Length |
| --- | --- | --- |
| F8fwd-Left-F | TGCAAGGCACGTACGTTTAC | 225 bp |
| F8fwd-Left-R | GACCACGTGCACAGAAAATG | |
| F8fwd-Right-F | TGATCCACTTCCAAATTTCTG | 192 bp |
| F8fwd-Right-R | GGCTCTTGTCAAATGGTTATCA | |
| F8rev-Left-F | TGCCTATGGCTATGAAGTGC | 221 bp |
| F8rev-Left-R | AAGATACAACGGGTTCTGTTTCA | |
| F8rev-Right-F | CACGTGCACAGAAAATGGAA | 216 bp |
| F8rev-Right-R | CCAGCTGGTGCTTGTCTGTA | |
| BBfwd-Left-F | TGAAGTGCAAATCCTAACAGTCC | 207 bp |
| BBfwd-Left-R | TTGTCTCATGAGCGGATACA | |
| BBfwd-Right-F | TTTATGCTTCCGGCTCGTAT | 212 bp |
| BBfwd-Right-R | CCAGCTGGTGCTTGTCTGTA | |
| BBrev-Left-F | TGCCTATGGCTATGAAGTGC | 213 bp |
| BBrev-Left-R | ATTAGGCACCCCAGGCTTTA | |
| BBrev-Right-F | TTGTCTCATGAGCGGATACA | 211 bp |
| BBrev-Right-R | CAAATGGTTATCAGTCTTGATCG | |

## Slide 50
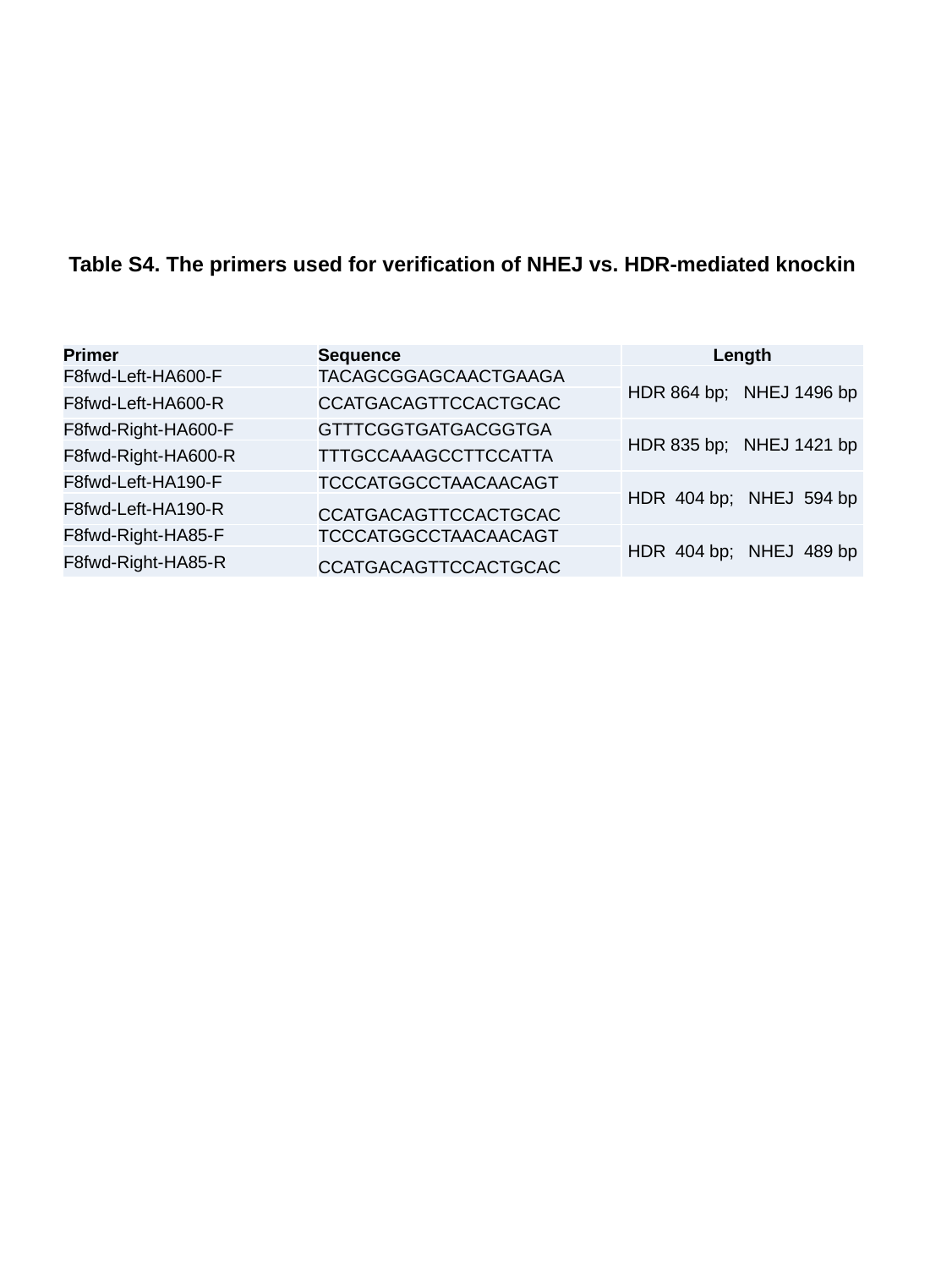

Table S4. The primers used for verification of NHEJ vs. HDR-mediated knockin
| Primer | Sequence | Length |
| --- | --- | --- |
| F8fwd-Left-HA600-F | TACAGCGGAGCAACTGAAGA | HDR 864 bp; NHEJ 1496 bp |
| F8fwd-Left-HA600-R | CCATGACAGTTCCACTGCAC | |
| F8fwd-Right-HA600-F | GTTTCGGTGATGACGGTGA | HDR 835 bp; NHEJ 1421 bp |
| F8fwd-Right-HA600-R | TTTGCCAAAGCCTTCCATTA | |
| F8fwd-Left-HA190-F | TCCCATGGCCTAACAACAGT | HDR 404 bp; NHEJ 594 bp |
| F8fwd-Left-HA190-R | CCATGACAGTTCCACTGCAC | |
| F8fwd-Right-HA85-F | TCCCATGGCCTAACAACAGT | HDR 404 bp; NHEJ 489 bp |
| F8fwd-Right-HA85-R | CCATGACAGTTCCACTGCAC | |

## Slide 51
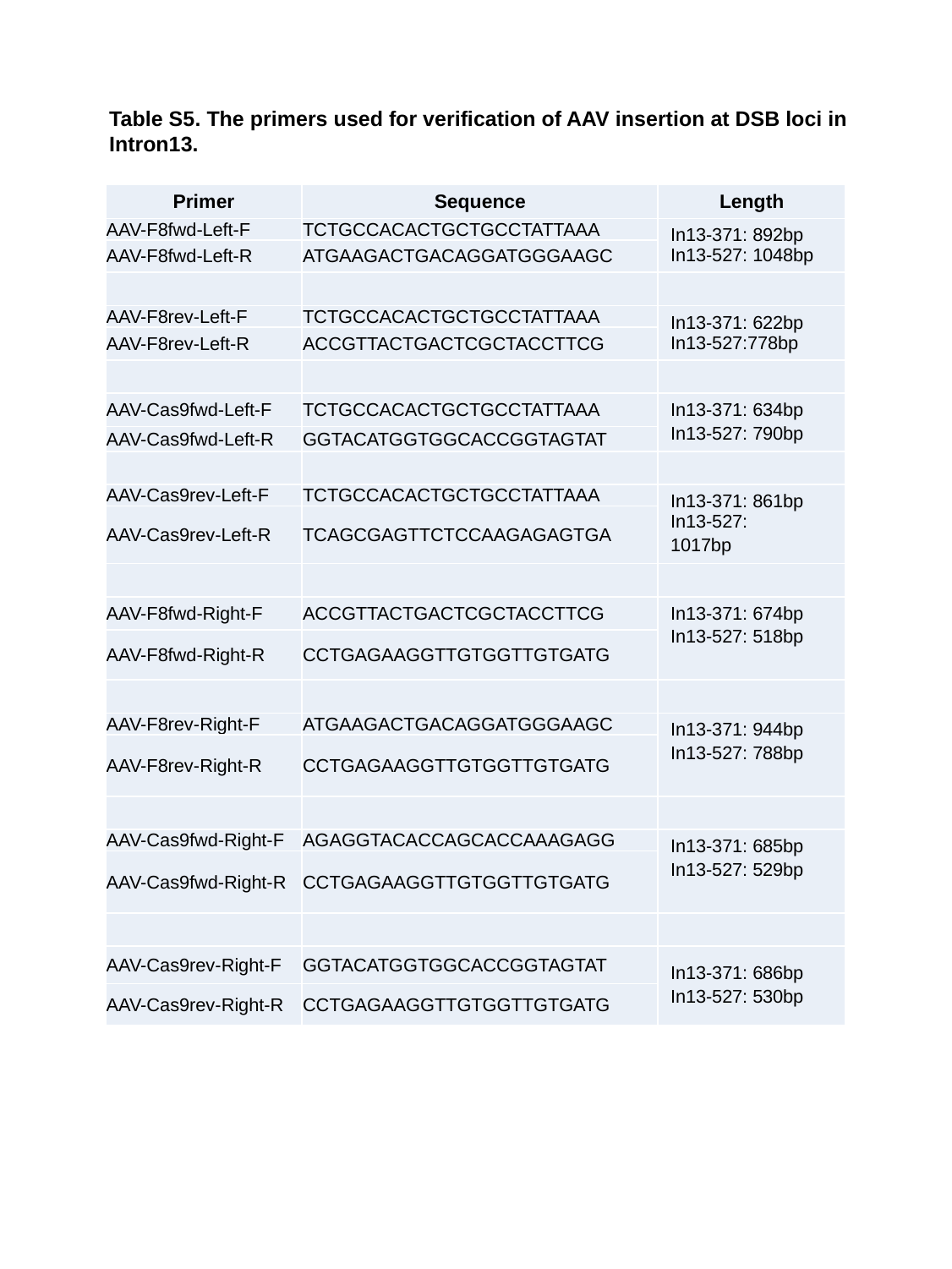

Table S5. The primers used for verification of AAV insertion at DSB loci in Intron13.
| Primer | Sequence | Length |
| --- | --- | --- |
| AAV-F8fwd-Left-F | TCTGCCACACTGCTGCCTATTAAA | In13-371: 892bp In13-527: 1048bp |
| AAV-F8fwd-Left-R | ATGAAGACTGACAGGATGGGAAGC | |
| | | |
| AAV-F8rev-Left-F | TCTGCCACACTGCTGCCTATTAAA | In13-371: 622bp In13-527:778bp |
| AAV-F8rev-Left-R | ACCGTTACTGACTCGCTACCTTCG | |
| | | |
| AAV-Cas9fwd-Left-F | TCTGCCACACTGCTGCCTATTAAA | In13-371: 634bp In13-527: 790bp |
| AAV-Cas9fwd-Left-R | GGTACATGGTGGCACCGGTAGTAT | |
| | | |
| AAV-Cas9rev-Left-F | TCTGCCACACTGCTGCCTATTAAA | In13-371: 861bp In13-527: 1017bp |
| AAV-Cas9rev-Left-R | TCAGCGAGTTCTCCAAGAGAGTGA | |
| | | |
| AAV-F8fwd-Right-F | ACCGTTACTGACTCGCTACCTTCG | In13-371: 674bp In13-527: 518bp |
| AAV-F8fwd-Right-R | CCTGAGAAGGTTGTGGTTGTGATG | |
| | | |
| AAV-F8rev-Right-F | ATGAAGACTGACAGGATGGGAAGC | In13-371: 944bp In13-527: 788bp |
| AAV-F8rev-Right-R | CCTGAGAAGGTTGTGGTTGTGATG | |
| | | |
| AAV-Cas9fwd-Right-F | AGAGGTACACCAGCACCAAAGAGG | In13-371: 685bp In13-527: 529bp |
| AAV-Cas9fwd-Right-R | CCTGAGAAGGTTGTGGTTGTGATG | |
| | | |
| AAV-Cas9rev-Right-F | GGTACATGGTGGCACCGGTAGTAT | In13-371: 686bp In13-527: 530bp |
| AAV-Cas9rev-Right-R | CCTGAGAAGGTTGTGGTTGTGATG | |

## Slide 52
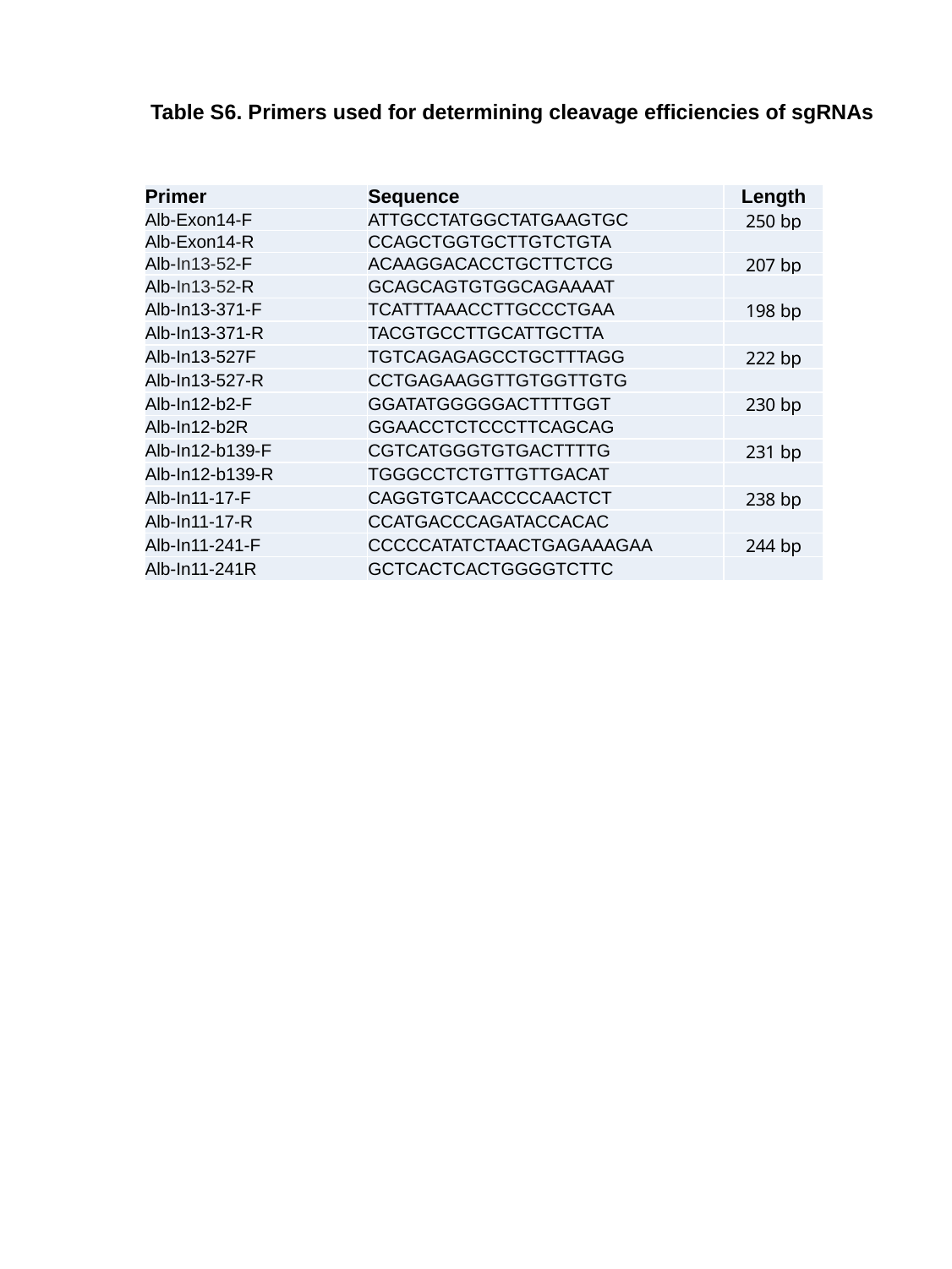

Table S6. Primers used for determining cleavage efficiencies of sgRNAs
| Primer | Sequence | Length |
| --- | --- | --- |
| Alb-Exon14-F | ATTGCCTATGGCTATGAAGTGC | 250 bp |
| Alb-Exon14-R | CCAGCTGGTGCTTGTCTGTA | |
| Alb-In13-52-F | ACAAGGACACCTGCTTCTCG | 207 bp |
| Alb-In13-52-R | GCAGCAGTGTGGCAGAAAAT | |
| Alb-In13-371-F | TCATTTAAACCTTGCCCTGAA | 198 bp |
| Alb-In13-371-R | TACGTGCCTTGCATTGCTTA | |
| Alb-In13-527F | TGTCAGAGAGCCTGCTTTAGG | 222 bp |
| Alb-In13-527-R | CCTGAGAAGGTTGTGGTTGTG | |
| Alb-In12-b2-F | GGATATGGGGGACTTTTGGT | 230 bp |
| Alb-In12-b2R | GGAACCTCTCCCTTCAGCAG | |
| Alb-In12-b139-F | CGTCATGGGTGTGACTTTTG | 231 bp |
| Alb-In12-b139-R | TGGGCCTCTGTTGTTGACAT | |
| Alb-In11-17-F | CAGGTGTCAACCCCAACTCT | 238 bp |
| Alb-In11-17-R | CCATGACCCAGATACCACAC | |
| Alb-In11-241-F | CCCCCATATCTAACTGAGAAAGAA | 244 bp |
| Alb-In11-241R | GCTCACTCACTGGGGTCTTC | |

## Slide 53
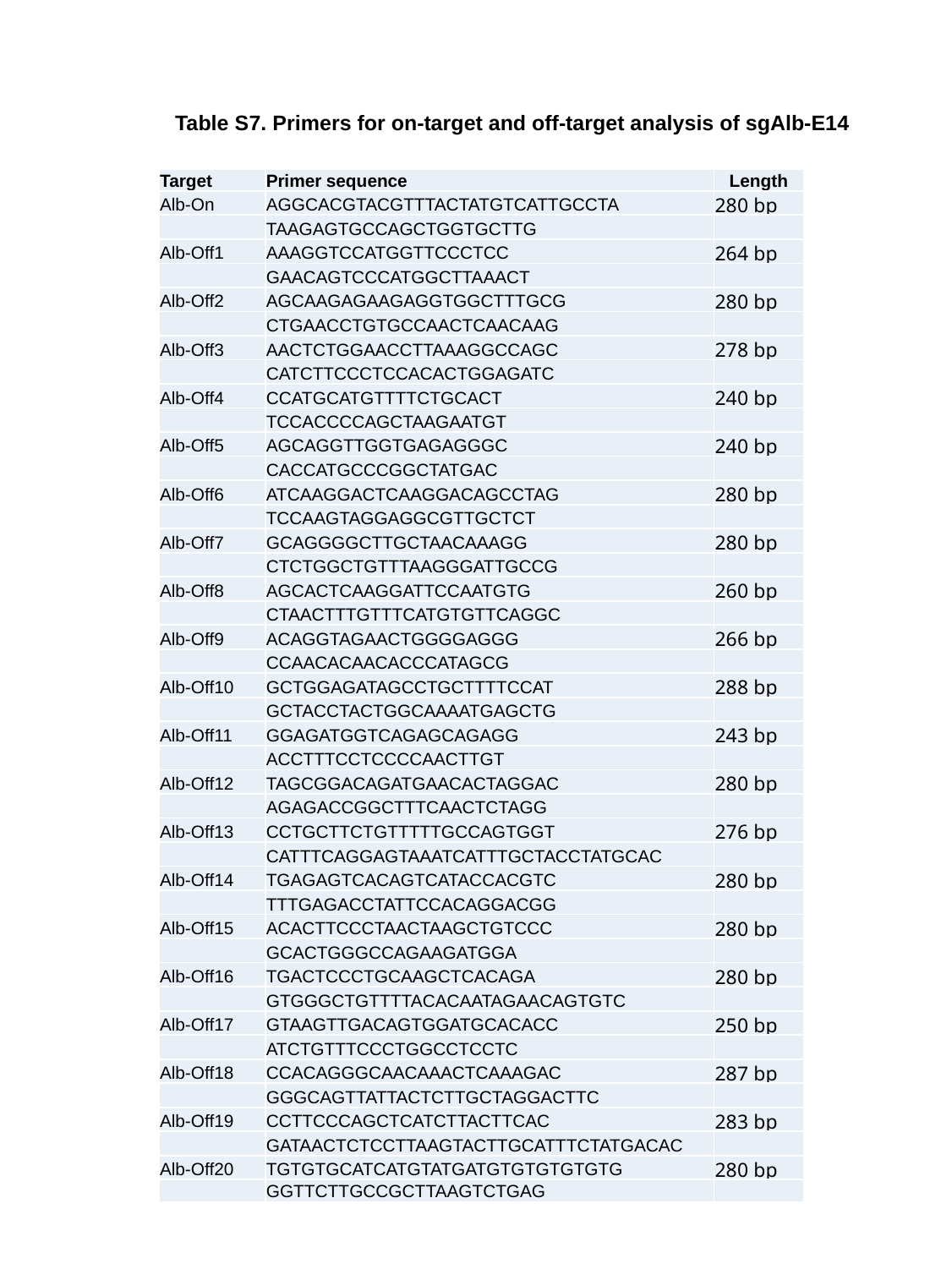

Table S7. Primers for on-target and off-target analysis of sgAlb-E14
| Target | Primer sequence | Length |
| --- | --- | --- |
| Alb-On | AGGCACGTACGTTTACTATGTCATTGCCTA | 280 bp |
| | TAAGAGTGCCAGCTGGTGCTTG | |
| Alb-Off1 | AAAGGTCCATGGTTCCCTCC | 264 bp |
| | GAACAGTCCCATGGCTTAAACT | |
| Alb-Off2 | AGCAAGAGAAGAGGTGGCTTTGCG | 280 bp |
| | CTGAACCTGTGCCAACTCAACAAG | |
| Alb-Off3 | AACTCTGGAACCTTAAAGGCCAGC | 278 bp |
| | CATCTTCCCTCCACACTGGAGATC | |
| Alb-Off4 | CCATGCATGTTTTCTGCACT | 240 bp |
| | TCCACCCCAGCTAAGAATGT | |
| Alb-Off5 | AGCAGGTTGGTGAGAGGGC | 240 bp |
| | CACCATGCCCGGCTATGAC | |
| Alb-Off6 | ATCAAGGACTCAAGGACAGCCTAG | 280 bp |
| | TCCAAGTAGGAGGCGTTGCTCT | |
| Alb-Off7 | GCAGGGGCTTGCTAACAAAGG | 280 bp |
| | CTCTGGCTGTTTAAGGGATTGCCG | |
| Alb-Off8 | AGCACTCAAGGATTCCAATGTG | 260 bp |
| | CTAACTTTGTTTCATGTGTTCAGGC | |
| Alb-Off9 | ACAGGTAGAACTGGGGAGGG | 266 bp |
| | CCAACACAACACCCATAGCG | |
| Alb-Off10 | GCTGGAGATAGCCTGCTTTTCCAT | 288 bp |
| | GCTACCTACTGGCAAAATGAGCTG | |
| Alb-Off11 | GGAGATGGTCAGAGCAGAGG | 243 bp |
| | ACCTTTCCTCCCCAACTTGT | |
| Alb-Off12 | TAGCGGACAGATGAACACTAGGAC | 280 bp |
| | AGAGACCGGCTTTCAACTCTAGG | |
| Alb-Off13 | CCTGCTTCTGTTTTTGCCAGTGGT | 276 bp |
| | CATTTCAGGAGTAAATCATTTGCTACCTATGCAC | |
| Alb-Off14 | TGAGAGTCACAGTCATACCACGTC | 280 bp |
| | TTTGAGACCTATTCCACAGGACGG | |
| Alb-Off15 | ACACTTCCCTAACTAAGCTGTCCC | 280 bp |
| | GCACTGGGCCAGAAGATGGA | |
| Alb-Off16 | TGACTCCCTGCAAGCTCACAGA | 280 bp |
| | GTGGGCTGTTTTACACAATAGAACAGTGTC | |
| Alb-Off17 | GTAAGTTGACAGTGGATGCACACC | 250 bp |
| | ATCTGTTTCCCTGGCCTCCTC | |
| Alb-Off18 | CCACAGGGCAACAAACTCAAAGAC | 287 bp |
| | GGGCAGTTATTACTCTTGCTAGGACTTC | |
| Alb-Off19 | CCTTCCCAGCTCATCTTACTTCAC | 283 bp |
| | GATAACTCTCCTTAAGTACTTGCATTTCTATGACAC | |
| Alb-Off20 | TGTGTGCATCATGTATGATGTGTGTGTGTG | 280 bp |
| | GGTTCTTGCCGCTTAAGTCTGAG | |
