# Supplementary material for: Curing hemophilia A by NHEJ-mediated ectopic F8 insertion in the mouse
Source: Genome Biol. 2019 Dec 16;20:276. doi: 10.1186/s13059-019-1907-9 (PMC6912951; doi:10.1186/s13059-019-1907-9)
Supplement: Supplementary file 4 — Additional file 4. Review history. [file 13059_2019_1907_MOESM4_ESM.docx]

Review History

**First round of review**

**Reviewer 1**

**Are you able to assess all statistics in the manuscript, including the appropriateness of statistical tests used?**

Yes, and I have assessed the statistics in my report.

**Comments to author:**

The authors treated Hemophilia A model mice by hydrodynamic injection of Cas9, gRNA and donors including their constructed double-cut donor, which has BDD8, two homology arms and two sgRNA target sites. They observed that the double-cut donor increases in vivo gene knock-in efficiency about 10-20 fold compared to conventional HDR donor in the liver. The detailed analyses revealed that NHEJ, not HDR, played a predominant role in donor integration. When no homology donor is used, they conclude that NHEJ-mediated insertion of BDD8 is a variable therapeutic strategy. For future therapeutic purposes, they also switched the delivery method to AAV and achieved a similar therapeutic effect. Life-long efficacy and safety were also examined using double-cut HDR/NHEJ donor (pD-tdTomato-BDDF8-sg). Overall, the experiments were well done and most of the results are supported. This is the first successful report of CRISPR mediated treatment of hemophilia A mice. I feel it is still necessary to clarify the following points before publication.

1.     As shown in Figure 2A and as the authors describe on page 9, NHEJ-mediated knock-in inserted an additional exon 14 at the Alb locus. This insertion should change the structure of the mRNA and protein as well (i.e. C terminal of Alb mRNA and protein now possesses two exon 14 repeats) since the left homology arm of the double-cut donor should include the splicing acceptor and exon 14. The authors should correct the mRNA and protein in Figure 2A and 4A. The authors should also move the sentence about exon 14 redundancy from page 9 to page 7.

2.     Another question is whether such aberrant Alb protein still possesses wild-type activity? Is there any unexpected effect by NHEJ-mediated knock-in? Furthermore, HDR-mediated knock-in in the 3'UTR of Alb is not likely to be transcribed because polyA is inserted in front of the 3'UTR. Will this affect the function of endogenous Alb? The authors should discuss these points as well.

3.     In figure 3, the authors showed that the double-cut donor was inserted via NHEJ in both forward and reverse directions. What is the direction of sgRNAs on the double-cut donor? If the direction of sgRNAs on the donor and target chromosome is opposite, it is possible that it is unidirectional NHEJ-mediated knock-in similar to the reported HITI method (Suzuki et al Nature 2016). To show the direction of the sgRNA on donor and target loci, the authors should show details of the sequence information of the sgRNA target in the figures.

4.     The authors demonstrated that transient immunosuppression can sustain therapeutic efficacy. Did the authors use immunosuppressants in the experiment of Figure 7 as well? If the data is a mixture of treated and non-treated mice, the data should be shown as individual panels.

Minor comments

5.     What is the difference between the double-cut donor system here, HMEJ (Yao X et al, Cell Res 2017) and MMEJ. Please include this in the main text.

6.     On page 5 and Figure 1D, the authors described that tdTomato+ cells represent the HDR-mediated knock-in efficiency. However, as the authors described later, most of the knock-in events should likely be mediated by NHEJ when they use the double-cut donor. The authors should simply use "knock-in efficiency" instead of "HDR efficiency" in this sentence.

7.     In Supplementary Figure 1A, the sequence doesn't align well.

8.     In figure 2A, homology arms between donor and Alb locus don't align well.

9.     Figure 2B has not been cited in the main text.

10.     The authors should show the sgAlb target site in Supplementary Figure 6. In this way, it is easy to understand why precise NHEJ is low at some junction sites. As I suggested above, HITI can avoid this as well as reverse integration. Please also discuss these points.

**Reviewer 2**

**Are you able to assess all statistics in the manuscript, including the appropriateness of statistical tests used?**

Yes, and I have assessed the statistics in my report.

**Comments to author:**

In this paper, the authors performed in vivo genome editing to cure F8 deficiency using two kinds of methods (hydrodynamic injection and AAV8-mediated method). They confirmed targeted knock-in in vivo, especially in liver tissue. In addition, they demonstrated long term rescue effect with functional recovery through AAV8-mediated-CRISPR/BDDF8 delivery study. The experimental designs were well organized and straightforward. The results are also very convincing. Moreover, an AAV-mediate delivery system has merit for in vivo genome editing due to their low immunogenicity.  
Here are some issues to improve this manuscript.
1)     Authors have to provide the whole sequences for BDDF8 used in this study.
2)     Is there any insertion of AAV-Cas9 sequence into the genome? It is necessary to check whether AAV-Cas9 vector was inserted into the host genome or not.
3)     Authors need to check the total albumin proteins in blood plasma in recipient HA mouse.
       It is known that FVIII cDNA has transcriptional repression sequences, so it is possible that insertion of FVIII cDNA and co-expression of FVIII and albumin in the same mRNA might be influenced the production of albumin in recipients.
4)     In Figure 4B, FVIII activities of HA190-130 and HA85-0 were above 300% compared to normal WT mice. Are there any problems in blood physiology caused by the high FVIII level?

**Authors Response**

**Point-by-point responses to the reviewers’ comments:**

Comment

*Response*

Reviewer #1
The authors treated Hemophilia A model mice by hydrodynamic injection of Cas9, gRNA and donors including their constructed double-cut donor, which has BDDF8, two homology arms and two sgRNA target sites. They observed that the double-cut donor increases in vivo gene knock-in efficiency about 10-20 fold compared to conventional HDR donor in the liver. The detailed analyses revealed that NHEJ, not HDR, played a predominant role in donor integration. When no homology donor is used, they conclude that NHEJ-mediated insertion of BDD8 is a variable therapeutic strategy. For future therapeutic purposes, they also switched the delivery method to AAV and achieved a similar therapeutic effect. Life-long efficacy and safety were also examined using the double-cut HDR/NHEJ donor (pD-tdTomato-BDDF8-sg). Overall, the experiments were well done and most of the results are supported. This is the first successful report of CRISPR mediated treatment of hemophilia A mice. I feel it is still necessary to clarify the following points before publication.

*RESPONSE: We appreciate this reviewer’s recognition that this is “the first successful report of CRISPR mediated treatment of hemophilia A mice.” We have also addressed all the other issues raised by reviewers.*

1. As shown in Figure 2A and as the authors describe on page 9, NHEJ-mediated knock-in inserted an additional exon 14 at the Alb locus. This insertion should change the structure of the mRNA and protein as well (i.e. C terminal of Alb mRNA and protein now possesses two exon 14 repeats) since the left homology arm of the double-cut donor should include the splicing acceptor and exon 14. The authors should correct the mRNA and protein in Figure 2A and 4A. The authors should also move the sentence about exon 14 redundancy from page 9 to page 7.

*RESPONSE: As suggested, we have moved the sentence about exon 14 redundancy from page 9 to page 7. We agree that the pattern of NHEJ-mediated knock-in insertion is complicated in our study. As such, we now showed the details of the schematic of genome editing at the Alb stop codon when NHEJ-mediated knock-in occurred in Supplementary Figure 4A. To enhance readability, we also showed the sgAlb recognition sites on pDonor and the genome.

The left-arm contains 6 bp of PAM sequence, 20 bp of exon 13, the whole intron 13, and 39 bp of exon 14 sequence. The right-arm contains 29 bp of exon 14 sequence, 549 bp of intron 14 and 6 bp of PAM sequence. “NHEJ insertion of pDonor is expected to create two identical introns 13, which may lead to two possible splice isoforms (Supplementary Figure 4A). To distinguish these possibilities, we designed primers to amplify the Alb-BDDF8 fusion transcript and observed only one band in liver gDNA samples from five independent mice.” “We speculate that the aberrant E14-E13 in-frame fusion exon was skipped for unknown reasons, likely because it is flanked by two identical introns.”

The full sequence of the expected RT-PCR product of the Alb-BDDF8 fusion gene was shown in Supplementary Figure 4B-C. Using primers targeting exon 11 and BDDF8 to amplify the RT-PCR product followed by Sanger sequencing indicates the formation of only the splice isoform 2 fusion transcript but not the splice isoform 1. We also showed the representative sequencing data in Supplementary Figure 4D to highlight the correct splicing of Alb exon 13 and exon 14, and the Exon14-E2A-BDDF4 junctions. As such, to prevent misunderstanding and miscommunication, we leave the Figure 2A unchanged but have deliberated the two possibilities in Supplementary Figure 4.*

2. Another question is whether such aberrant Alb protein still possesses wild-type activity? Is there any unexpected effect by NHEJ-mediated knock-in? Furthermore, HDR-mediated knock-in in the 3'UTR of Alb is not likely to be transcribed because polyA is inserted in front of the 3'UTR. Will this affect the function of endogenous Alb? The authors should discuss these points as well.

*RESPONSE: There are four types of NHEJ-mediated knock-in patterns: F8 forward, F8 reverse, Backbone forward, and Backbone reverse. We agree that in some cases, the aberrant Alb protein can be generated. These aberrant proteins may be degraded by proteasomes. To investigate the potential adverse effects, we followed up on treated mice for 2 years and did not observe any unexpected toxicity (Figure 7).

We now have shown the target sequence of sgAlb in Figures 1, 3, and S4. It shows that the expected cleavage site is 2 bp before the stop codon TAA. As such, the inserted BDDF8 is situated before 3’UTR. In our design, due to the presence of PolyA at the end of BDDF8, the Alb 3’UTR will not be transcribed. We noticed that the Alb 3’UTR has enhancer activity, and thus loss of this sequence in the fusion transcript may decrease the protein expression levels of Alb. However, this is not expected to affect the function of endogenous Alb.

Our knockin efficiency is relatively low (~5%); thus more than 95% of Alb gene in the liver will not be affected. To evaluate the potential side effects of knockin at the Alb locus, we evaluated total albumin proteins in blood plasma after a one-year treatment. We observed an ~5% decrease in Alb levels in treated mice relative to untreated mice, but the difference is not statistically significant. (Figure 7D).*

3. In Figure 3, the authors showed that the double-cut donor was inserted via NHEJ in both forward and reverse directions. What is the direction of sgRNAs on the double-cut donor? If the direction of sgRNAs on the donor and target chromosome is opposite, it is possible that it is unidirectional NHEJ-mediated knock-in similar to the reported HITI method (Suzuki et al Nature 2016). To show the direction of the sgRNA on donor and target loci, the authors should show details of the sequence information of the sgRNA target in the figures.

*RESPONSE: As requested, we have now shown the direction and sequences of sgRNAs on the double-cut donor and target site in Figure 3A. We understand the HITI design will promote unidirectional NHEJ-mediated knock-in. However, when we initiated this project, the Nature paper has not been published. In addition, we wished to guide HDR insertion using double cut donor instead of NHEJ. NHEJ-mediated insertion in the liver is a serendipitous finding. We believe that if we had used the HITI design, the therapeutic effects would be further increased due to increased forward insertion.*

4. The authors demonstrated that transient immunosuppression can sustain therapeutic efficacy. Did the authors use immunosuppressants in the experiment of Figure 7 as well? If the data is a mixture of treated and non-treated mice, the data should be shown as individual panels.

*RESPONSE: As requested, in Figure 7 and Supplementary Figure 17, we have now separated the data for the transient immunosuppression treated and non-treated mice.*

Minor comments

5. What is the difference between the double-cut donor system here, HMEJ (Yao X et al, Cell Res 2017) and MMEJ. Please include this in the main text.

*RESPONSE: In principle, our double-cut donor is identical to the HMEJ donor. Both are knock-in templates flanked by sgRNA-PAM sequences with different length of homology arms and is released after Cas9-sgRNA cleavage. We published our paper in Genome Biology in 2017 three months earlier than Dr. Yao’s paper. However, they used the term HMEJ (homology-mediated end joining) to describe this type of donor design. In our design, we use homology arms of 300-600 bp, whereas Dr. Yao’s HMEJ approach uses longer homology arms of 800 bp. The MMEJ (microhomology-mediated end-joining) is a similar approach, but using short homology arms of 5-20 bp. In our experience, MMEJ is much less efficient in guiding HDR editing than the previous approach. We have now discussed these differences.*

6. On page 5 and Figure 1D, the authors described that tdTomato+ cells represent the HDR-mediated knock-in efficiency. However, as the authors described later, most of the knock-in events should likely be mediated by NHEJ when they use the double-cut donor. The authors should use "knock-in efficiency" instead of "HDR efficiency" in this sentence.

*RESPONSE: We have now corrected the typos and use "knock-in efficiency" instead of "HDR efficiency."*

7. In Supplementary Figure 1A, the sequence does not align well.

*RESPONSE: We have re-aligned the sequence in Supplementary Figure 1A.*

8. In Figure 2A, homology arms between donor and Alb locus don't align well.

*RESPONSE: We have re-aligned the homology arms between donor and Alb locus in Figure 2A.*

9. Figure 2B has not been cited in the main text.

*RESPONSE: We have cited Figure 2B in the main text.*

10. The authors should show the sgAlb target site in Supplementary Figure 6. In this way, it is easy to understand why precise NHEJ is low at some junction sites. As I suggested above, HITI can avoid this as well as reverse integration. Please also discuss these points.

*RESPONSE: We have now shown the sgAlb target site (CCTTAG|CCTAAACACATCACAAC) in Supplementary Figure 6. The direction of sgRNAs on the double-cut donor and target site was also shown in Figure 3A.

We understand that the HITI design will promote unidirectional insertion by NHEJ. However, when we initiated this project, the Nature paper has not been published. Besides, we wished to guide HDR insertion using a double cut donor instead of NHEJ. NHEJ-mediated insertion in the liver is a serendipitous finding. We believe that if we had used the HITI design, we might have observed better therapeutic effects due to increased forward insertion.*


===
Reviewer #2:
In this paper, the authors performed in vivo genome editing to cure F8 deficiency using two kinds of methods (hydrodynamic injection and AAV8-mediated method). They confirmed targeted knock-in in vivo, especially in liver tissue. In addition, they demonstrated long term rescue effects with functional recovery through AAV8-mediated-CRISPR/BDDF8 delivery study. The experimental designs were well organized and straightforward. The results are also very convincing. Moreover, an AAV-mediate delivery system has merit for in vivo genome editing due to their low immunogenicity.

Here are some issues to improve this manuscript.
1) Authors have to provide the whole sequences for BDDF8 used in this study.

*RESPONSE: We have now provided the whole sequence of BDDF8 used in this study in Supplementary Figure 3F.*

2) Is there any insertion of AAV-Cas9 sequence into the genome? It is necessary to check whether AAV-Cas9 vector was inserted into the host genome or not.

*RESPONSE: We have designed primers to amplify the junction sequences, and found insertion of both AAV-F8 and AAV-Cas9 sequences in double-strand break sites (Supplementary Figure 12 and Supplementary Figure 13). We sequenced over 100 junctions and found that NHEJ-mediated insertion leads to an average of ~15 bp deletions in the genome cleavage site and ~80-bp deletion of AAV ITR.

An early report showed that (Chandler et al, J Clin Invest. 2015) genes known to have higher expression in the liver, such as albumin, were the most susceptible to AAV integrations. More recently, Hanlon et al. (Nature communication. 2019) observed high levels of AAV integration into Cas9-induced double-strand breaks in cultured mouse tissues. In another report (Nelson et al, Nature medicine. 2019), using Nextera-transposon-based library preparation method for unbiased sequencing, AAV integrations were observed in all treated mice.

Although AAV-mediated expression of Cas9 or integration of Cas9 did not lead to significant genome-wide genotoxicity, decreasing AAV-Cas9 integration is clinically beneficial. Toward that goal, the use of a self-deleting AAV-CRISPR system may be able to effectively remove Cas9 protein in mouse liver while retaining efficient in vivo editing of endogenous targets (Molecular Therapy: Methods & Clinical Development. 2019).

[1] Chandler R J, LaFave M C, Varshney G K, et al. Vector design influences hepatic genotoxicity after adeno-associated virus gene therapy[J]. The Journal of clinical investigation, 2015, 125(2): 870-880.

[2] Hanlon K S, Kleinstiver B P, Garcia S P, et al. High levels of AAV vector integration into CRISPR-induced DNA breaks[J]. Nature communications, 2019, 10(1): 1-11.

[3] Nelson C E, Wu Y, Gemberling M P, et al. Long-term evaluation of AAV-CRISPR genome editing for Duchenne muscular dystrophy[J]. Nature medicine, 2019, 25(3): 427.

[4] Li A, Lee C M, Hurley A E, et al. A self-deleting AAV-CRISPR system for in vivo genome editing[J]. Molecular Therapy-Methods & Clinical Development, 2019, 12: 111-122.*

3) Authors need to check the total albumin proteins in blood plasma in recipient HA mouse. It is known that FVIII cDNA has transcriptional repression sequences, so it is possible that insertion of FVIII cDNA and co-expression of FVIII and albumin in the same mRNA might be.

*RESPONSE: As requested, we have now evaluated total albumin proteins in blood plasma after one-year treatment. We observed an ~5% decrease in Alb levels in treated mice relative to untreated mice, but the difference was not statistically significant (Figure 7D). We agree that integration of BDDF8 at Alb will lead to decreased expression of Alb， and thus will influence the production of albumin in recipients. However, our knockin efficiency is relatively low (~5%); thus more than 95% of Alb gene in the liver will not be affected.*

4) In Figure 4B, FVIII activities of HA190-130 and HA85-0 were above 300% compared to normal WT mice. Are there any problems in blood physiology caused by the high FVIII level?

*RESPONSE: We did not observe any apparent problems in blood physiology caused by the high-level F8 activity. High-level F8 or F9 levels have also been previously reported after gene therapy, but we did not notice any report of potential adverse effects associated with superphysiological levels of coagulation factors in the mouse. Of course, in clinical gene therapies, the F8 levels will be closely monitored.*

**Second round of review**

**Reviewer 1**

The authors have addressed all my concerns and I feel this version is suitable for publication.

**Reviewer 2**

There isn't any comment.
